# Sequential Homo‐ and Hetero‐Coupling of Isocyanide and Carbon Monoxide Mediated by Acyclic Imino(silyl)Silylene

**DOI:** 10.1002/anie.9929509

**Published:** 2026-02-24

**Authors:** Huaiyuan Zhu, Arseni Kostenko, Xufang Liu, Shigeyoshi Inoue

**Affiliations:** ^1^ TUM School of Natural Sciences Department of Chemistry Institute of Silicon Chemistry and Catalysis Research Center Technische Universität München (TUM) Garching bei München Germany

**Keywords:** carbon monoxide, coupling reaction, isocyanide, silaazacycle, silylene

## Abstract

Carbon monoxide (CO) and its isoelectronic analogues, isocyanides (RNC), have been extensively employed as model substrates to elucidate elementary steps of the Fischer–Tropsch (F–T) process, particularly those governing C–C bond construction. Building on this concept, we demonstrate carbon chain growth mediated by an acyclic imino(hypersilyl)silylene through sequential reactions with CO and isocyanides, ultimately affording well‐defined C_6_ frameworks composed of four isocyanide and two CO units. A proposed mechanism for the homo‐ and hetero‐coupling processes, derived from experimental observations and corroborated by quantum chemical calculations, reveals that the silylene functions as an initiator, inducing the stepwise homocoupling of RNC through consecutive formation of electrophilic sites, as well as promoting heterocoupling reactions with CO.

## Graphical abstract

We demonstrate the sequential reactions between an imino(hypersilyl)silylene and aryl isocyanide or carbon monoxide as a molecular‐level model of the Fischer–Tropsch process, providing systematic and detailed insight into C–C bond formation during the homologation of C1 building blocks.

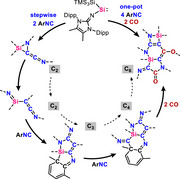

## 1 Introduction

**FIGURE 1:**
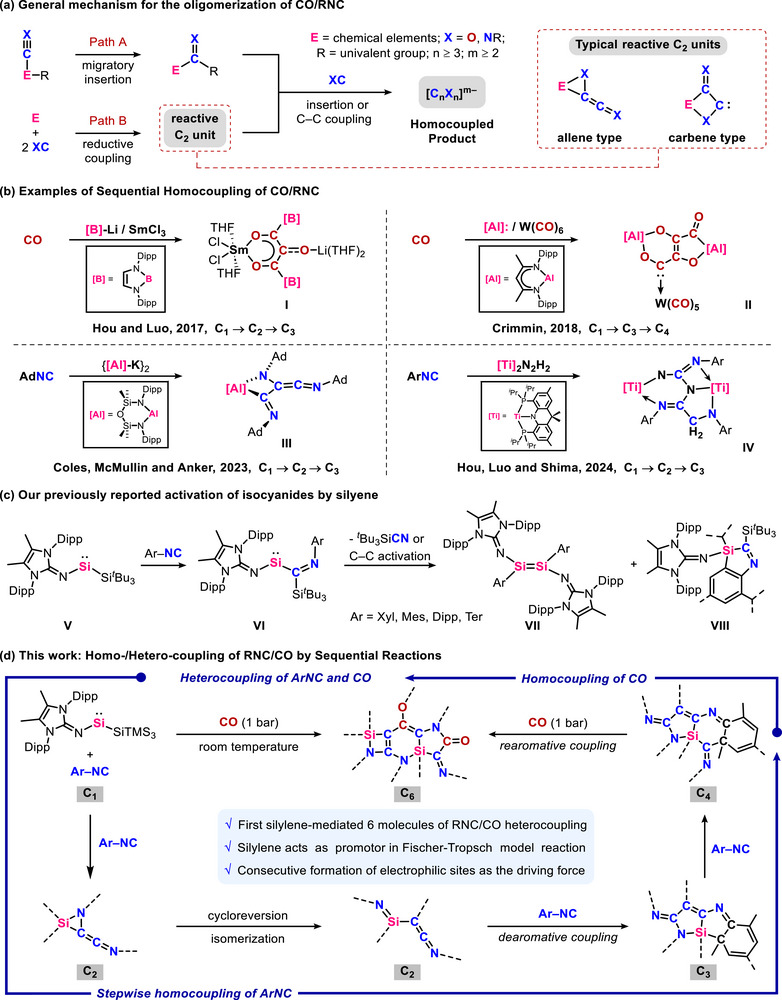
(a) General mechanism for the oligomerization of CO/RNC; (b) Examples of sequential homocoupling of CO/RNC; (c) Our previously reported isocyanide activation by imino(supersilyl)silylene **V**; (d) Present work.

Homocoupling of simple unsaturated building blocks to construct complex molecules is one of the cornerstones of organic synthesis. A pivotal challenge in this area has been the conversion of carbon monoxide (CO) into hydrocarbon fuels, relevant to the industrial Fischer−Tropsch (F–T) process [1, 2]. Even though F–T process has been widely used for nearly a century, its fundamental steps of C–C bond formation remain elusive, and the formation of hydrocarbons is generally nonselective due to the heterogeneous catalysis [3, 4]. To gain a deeper understanding of the reaction mechanism and thereby develop more efficient and selective catalysts, the homogeneous complexes, leveraging their tunable structures and relatively facile characterization, have been utilized to model the reductive oligomerization of CO as well as its isoelectronic analogue, isocyanide (RNC), over the past decades [5, 6, 7, 8, 9]. To date, investigations have been focused on the reactions of transition metal or main group complexes with CO/RNC to form C_2_ [10, 11, 12, 13, 14, 15, 16, 17, 18, 19], C_3_ [20, 21, 22, 23, 24]_,_ and C_4_ [25, 26, 27, 28, 29] fragments or even uncommon higher‐order homologs C_5_ and C_6_ units [30, 31, 32, 33, 34, 35, 36, 37]. These studies revealed two general mechanisms of homocoupling of CO/RNC. One approach is the migratory insertion of CO/RNC into element–element bond to yield acyl/iminoacyl‐substituted complexes, followed by C–C coupling or multiple insertion with additional CO/RNC to afford oligomers (Path A, Figure 1a) [5, 23]. Another widely adopted approach involves low‐valent complexes, such as aluminum(I) [23, 36, 37], titanium(III) [24], magnesium(I) [22], and silylene [20], to promote C–C reductive coupling of CO/RNC without prior migratory insertion. This results in the formation of O/N‐substituted C_2_ units, which serve as reactive intermediates for the further coupling with additional CO/RNC (Path B, Figure 1a). Nevertheless, systems enabling controlled reductive oligomerization of CO/RNC through sequential reactions from isolable C_1_ to C*_n_* units (*n* ≥ 3), which may offer valuable insights into the underlying mechanism, are seldom reported. In 2017, Hou et al. developed bimetallic boryl lithium/samarium [21] system, which enables stepwise chain growth of CO to form C_3_ unit **I**. Similar metal‐metal cooperative, sequential CO homocoupling to generate a C_4_ framework **II** was reported by Crimmin group in 2018 using Al(I)/metal carbonyls system (Figure 1b) [27, 28]. However, progressive oligomerization of CO results in depletion of active sites and concomitant formation of increasingly strong metal–oxygen bonds within these bimetallic systems. This phenomenon likely accounts for the inability of **I** and **II** to undergo further CO coupling. Recently, stepwise homologations of RNC were also achieved by Coles et al. and Hou et al. using an aluminyl anion [23] and a dinitrogen‐bridged dititanium dihydride complex [24], respectively, ultimately affording C_3_ units **III** and **IV** (Figure 1b). The suppression of further RNC coupling at **III** and **IV** can be rationalized by the combined effects of active‐site attrition during the homocoupling process and steric hindrance imposed by the RNC frameworks. Despite these advances, the formation of higher‐order C_6_ units by sequential reactions has yet to be reported.

Silylenes (R_2_Si:), the heavier analogues of carbenes, have garnered significant attention over the past several decades and continue to be a focal point of contemporary research due to their distinctive electronic structures, remarkable reactivity profiles, and promising potential in catalysis [38, 39]. The stoichiometric coupling of silylenes with CO or RNC to afford silicon carbonyl or silylene isocyanide complexes has been well documented [17, 40, 41, 42, 43, 44, 45]. In contrast, silylenes‐mediated homo‐ or hetero‐coupling of CO and RNC to form silacycles are comparatively rare [15, 16, 20, 46]. Our group has been focusing on the study of two‐coordinate acyclic silylenes stabilized by a series of *N*‐heterocyclic imines (NHI) and bulky silyl groups, with emphasis on the substituent effect on their remarkable reactivity toward activation of small molecules and inert bonds. That is, our studies systematically revealed the synthesis and isolation of Si(II) species from diiminodisilene [47, 48] (dimer of iminosilylene) to transient iminosilylene [49, 50] and ultimately to isolable imino(supersilyl)silylene **V** with pronounced *σ*‐donor and *π*‐acceptor abilities [51]. Leveraging the unique ambiphilic properties of **V**, its reaction with aryl isocyanides selectively formed an isolable migratory‐inserted (imino‐*κ* ^1^N)(*η* ^1^‐iminoacyl‐*κ* ^1^C)silylene intermediate **VI**. This intermediate serves as a branching point for divergent reactivity, delivering either diaryldiiminodisilenes **VII** via Ar–NC bond cleavage or benzazasilole **VIII** via intramolecular C–C bond activation (Figure 1c) [52]. Building on previous studies of the silyl effect in silylsilylenes, we aimed to introduce –SiTMS_3_ (hypersilyl) substituent for the synthesis of a novel iminosilylene [47, 49, 50, 51, 53, 54, 55]. The idea was to explore previously undiscovered reaction pathways involving the migration of either the hypersilyl or the TMS fragments. Such migrations routinely occur in organosilicon compounds, and here they are expected to give rise to unique reactive intermediates, in which subsequent C–C coupling reactions of isocyanides and CO could lead to the formation of novel silicon‐containing products [50, 53, 55, 56].

In this contribution, we present a comprehensive study into the mechanism underlying homo‐ and hetero‐coupling reactions involving isocyanides and CO mediated by an isolable acyclic imino(hypersilyl)silylene (Figure 1d). This study demonstrates carbon chain growth from C_1_ → C_2_ → C_3_ → C_4_ → C_6_ units through sequential reactions, as supported by experimental and computational investigations. In this reaction, which mimics the F–T process, an acyclic silylene acts as an initiator, triggering the homocoupling of RNC to generate a C_2_ reactive intermediate. This intermediate can subsequently undergo sequential insertion of one or two additional RNC molecules, affording dearomative silacycles. Remarkably, these dearomative silaazacycles are capable of regenerating the reactive intermediates through a rearomatization process, thereby enabling subsequent heterocoupling with CO in an uninterrupted cascade sequence. This transformation ultimately furnishes silaazacycles bearing both imino and acyl functionalities.

## 2 Results and Discussion

### 2.1 Homocoupling of Aryl Isocyanides

**FIGURE 2:**
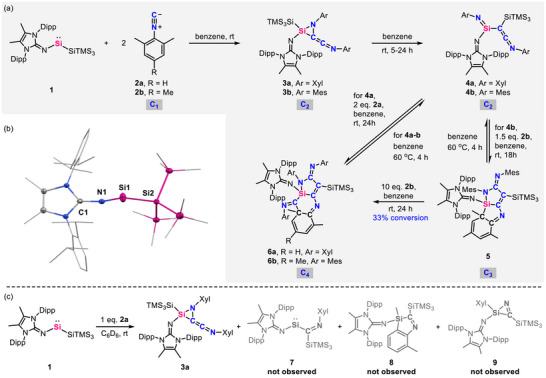
(a) Sequential reactions of **1** and **2**; (b) Molecular structures of **1** [58]. Ellipsoids set at 50% probability to compensate for advanced thermal motion. Hydrogen atoms are omitted for clarity; (c) Stoichiometric reaction of **1** with **2a**.

Similarly to our previous report on the synthesis of **V** [51], imino(hypersilyl)silylene **1** was prepared by debromination of methylated backbone *N*‐heterocyclic iminosilicon tribromide with two equivalents of the KSiTMS_3_ and isolated as intensely blue powder in 65% yield (Figures 2a and S1.1–S1.3). The ^29^Si NMR displays a signal at 405.7 ppm for the central silicon (Figure S1.3), which is upfield shifted than previously reported **V** (454.0 ppm) due to the stronger σ‐donating character of the hypersilyl group [57]. The molecular structure of **1** was determined by single crystal x‐ray diffraction (SC‐XRD) analysis (Figure 2b and Table S1). The Si1–N1 distance of 1.656(2) Å and the N1–Si1–Si2 angle of 105.73(6)° are very close to those of **V** (1.665(2) Å and 106.15(7)°) [51].

**FIGURE 3:**
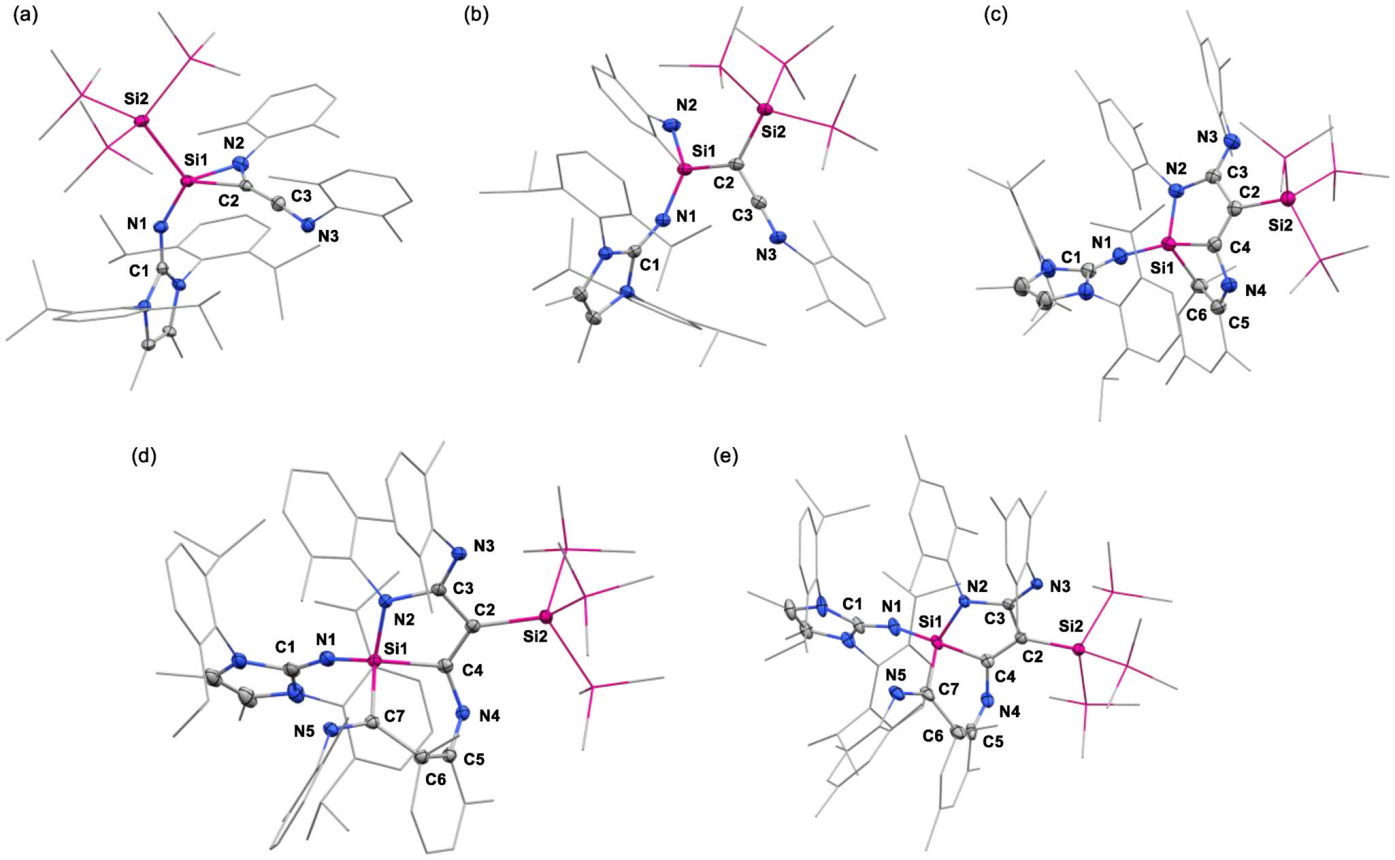
Molecular structures of **3a** (a), **4a** (b), **5** (c), **6a** (d), and **6b** (e) [58]. Ellipsoids set at 50% probability to compensate for advanced thermal motion. Hydrogen atoms are omitted for clarity.

To investigate the capability of **1** to mediate isocyanide homocoupling and to elucidate the silyl effect in comparison with **V** (Figure 1c), **1** was treated with two equivalents of xylyl isocyanide (**2a**) in benzene at room temperature, resulting in a rapid color change from blue to brown within seconds (Figures 2a and S2.1–S2.5). After crystallization from pentane solution at ‐30°C, compound **3a**, a 1,2‐azasiliridine with an exocyclic ketenimine moiety, was isolated as red crystals in high yield. The ^29^Si NMR spectrum of **3a** features a singlet at ‐120.7 ppm for the central silicon (Figure S2.3). In the ^13^C NMR spectrum, the signals appearing at 73.9 and 193.6 ppm correspond to the carbon from the ketenimine moiety (Figure S2.2). The subsequent crystallographic study unambiguously confirmed the molecular structure of **3a**, as shown in Figure 3a and Table S1. The C2–C3 distance (1.284(5) Å), C3–N3 distance (1.245(5) Å), and the C2–C3–N3 angle (173.7(5)°) in the keteimine fragment are similar to known exocyclic ketenimine [37]. The formation of **3a** proceeds through the homologation of two equivalents of **2a** mediated by **1**, in which *N*‐substituted C_2_ units are always proposed as reactive intermediates for the further coupling with additional isocyanides (Figure 1b) [23, 24, 37].

Interestingly, leaving **3a** at room temperature in benzene solution led to the appearance of a different species, detected by the ^29^Si NMR. Full consumption of **3a** was achieved within 24 h, and the 1‐silaimino‐2‐ketenimine **4a** was isolated quantitatively (Figures 2a and S4.1–S4.3). In the ^29^Si NMR spectrum, the central silicon is observed at ‐36.2 ppm (Figure S4.3), which is downfield shifted in comparison to **3a** and lies in the range of known silaimines (‐2.5 to ‐56.8 ppm) [59, 60]. The ^13^C NMR spectrum displays signals at 19.1 and 165.9 ppm, assigned to the ketenimine unit (Figure S4.2). These values are upfield shifted compared to **3a** and other main‐group‐element‐substituted ketenimines [18, 37], probably caused by the electron‐donating silaimino group, in line with the result of downfield shifted silicon. The molecular structure of **4a** was determined by SC‐XRD analysis, revealing adjacent silaimine and keteimine units formed by ring opening of SiCN ring and subsequent hypersilyl migration (Figure 3b and Table S1). It is worth mentioning that the formation of **4a** clearly demonstrates a silyl effect between **1** and **V**. The hypersilyl group readily undergoes migration from the silicon center, thereby generating a new electronic site that enables access to new reactivity. In addition, the reaction of **1** with one equivalent of **2a** at either room temperature or ‐30°C consistently gave to **3a** along with a minor fraction of unidentified products (Figure 2c). Notably, these undefined products do not correspond to migratory‐inserted, intramolecular C–C activation or C–N cleavage products (**7**‐**9**) as evidenced by ^1^H and ^29^Si NMR spectra (Figures S2.4–S2.5).

Although the analogues of **3a** are commonly proposed as reactive intermediates for the subsequent coupling with additional isocyanides (Figure 1b) [23, 24, 37], no reaction was originally observed between **3a** and **2a** in our case. Gratifyingly, stirring a benzene solution of **4a** and with two additional equivalents of **2a** led to the color change from yellow to orange with the formation of silaazatricycle **6a** within 24 h at room temperature (Figures 2a and S7.1–S7.4). In the ^29^Si NMR spectrum, the central silicon exhibits a singlet at ‐13.5 ppm (Figure S7.4). Subsequent SC‐XRD analysis revealed the molecular structure of **6a** with a dearomative tricyclic organosilicon core formed by the homologation of **4a** and another two equivalents of **2a** (Figure 3d and Table S1), which clearly evidenced **4a** as the key intermediate responsible for the further coupling of isocyanides from C_2_ to C_4_ unit. Notably, heating a benzene solution of **6a** to 60°C for 4 h, quantitatively formed **4a** and **2a** (Figure 2a). Leaving the reaction mixture at room temperature resulted again in the slow conversion to **6a**. The results clearly show that there is a **4a** + **2a ⇆ 6a** equilibrium.

To gain further insight into the mechanism of C_2_ to C_4_ chain growth, the reaction of **1** with slightly bulkier mesityl isocyanide (**2b**) was performed. Treating **1** with two equivalents of MesNC (**2b)** in benzene at room temperature led to the C_2_ homologated product **3b** as the major product (Figures 2a and S3.1–S3.2). Similarly, leaving **3b** in benzene at room temperature for 5 h resulted in the ring opening and silyl migration of **3b** to yield **4b** (Figures 2a and S5.1–S5.3). The ^29^Si NMR spectra of **3b** (‐121.3 ppm, Figure S3.2) and **4b** (‐36.7 ppm, Figure S5.3) feature almost the same chemical shift as **3a** (‐120.7 ppm, Figure S2.3) and **4a** (‐36.2 ppm, Figure S4.3). Surprisingly, stirring **4b** and 1.5 equivalents of **2b** in benzene afforded the C_3_ homologated silatricycle **5** (Figures 2a and S6.1–S6.4). Moreover, treatment of **5** with excess **2b** (10 eq.) in C_6_D_6_ resulted in the formation of minor amounts of the C_4_ homologated silatricycle **6b**, in addition to unreacted starting materials (33% conversion of **5**, Figures 2a and S8.1), indicating the pivotal intermediacy of the C_3_ product **5** in the C_2_ to C_4_ chain growth. Although the purification of **6b** failed due to the similar solubility of **5** and **6b**, the molecular structures of **5** and **6b** were undoubtedly determined by SC‐XRD analysis, revealing the C_3_ and C_4_ homologated dearomative silatricycles (Figure 3c,e and Table S1). As with **6a**, heating a benzene solution of **5** or **6b** to 60°C for 4 h, quantitatively formed **4b** and **2b** (Figure 2a). These results clearly indicate the existence of an equilibrium between **4**, **5,** and **6**.

**FIGURE 4:**
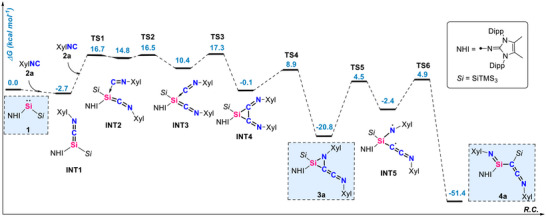
Free energy reaction coordinate diagram for the proposed mechanism of the formation of **3a** and **4a** from 1 in the presence of XylNC **2a** at the (SMD = Benzene)PW6B95‐D4/def2‐QZVPP//r^2^SCAN‐3c level of theory. Experimentally characterized compounds are highlighted in blue boxes.

To understand the capability of the silylene to act as a mediator in the stepwise homocoupling of isocyanide and to compare it with the previously reported reactivity, quantum chemical calculations were carried out using ORCA software (Table S2) [61]. The calculation shows that the formation of the C_2_ species **3a**, where the silylene consecutively uptakes two molecules of the isocyanide, enabling the C–C coupling, is exergonic by 20.8 kcal mol^−1^ (Figure 4). This proceeds via the initial formation of allenic silaketenimine **INT1**. This step is barrierless and exergonic by 2.7 kcal mol^−1^, similar to our previous report [52]. Additional coordination of an XylNC via **TS1**, followed by isomerization via **TS2,** allows for the first C–C coupling event to take place via the **TS3** at 17.3 kcal mol^−1^. This results in intermediate **INT4**, which can isomerize to the isolated compound **3a**. Although the previously described migratory insertion could also occur [52], the relatively thermoneutral energy difference between **INT1** (‐2.7 kcal mol^−1^, Figure 4) and migratory‐inserted product **7** (‐4.6 kcal mol^−1^, Figure S11) suggests the existence of an equilibrium between **1**, **INT1,** and **7**. In addition, the higher kinetic barriers associated with subsequent intramolecular C–C activation (by 4.4 kcal mol^−1^) or C–N cleavage (by 7.7 kcal mol^−1^) in comparison to the formation of **3a**, disfavor the generation and isolation of **8** and **9**. This outcome aligns well with our experimental observations (Figures 2c, 4 and S11). The isolation of **3a** is made possible by the relatively high barrier for the **3a → 4a** transformation. For this process to take place, the endocyclic C–N bond must cleave, forming a singlet diradical intermediate **INT5**. Then, the hypersily migration from the central Si atom to the geminal carbon forms the isolated compound **4a** in a highly exergonic fashion, which implies the regeneration of **1** is both thermodynamically and kinetically unfavorable via retro‐silyl migration.

**FIGURE 5:**
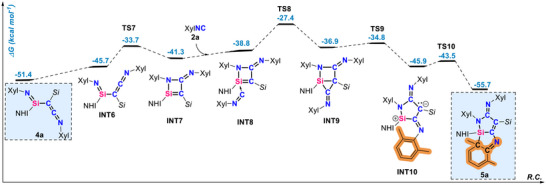
Free energy reaction coordinate diagram for the proposed mechanism of the formation of **5a** from **4a** in the presence of XylNC **2a** at the (SMD = Benzene)PW6B95‐D4/def2‐QZVPP//r^2^SCAN‐3c level of theory. Experimentally characterized compounds are highlighted in blue boxes.

We considered that the Si–N double bond in **4a** could potentially serve as an electrophilic site for the complexation of additional isocyanide molecules, similarly to the silaketenimine intermediate, which would lead to the formation of **5a**. However, we could not find a feasible route for such a scenario. Instead, **4a** has a slightly energetically less favorable rotamer **INT6**, which can undergo an intramolecular cyclization forming **INT7** (Figure 5). This species features a four‐membered Si–N–C–C ring, with an endocyclic silene (Si═C) fragment, that acts as an electrophilic site for complexation with an additional equivalent of isocyanide. Similar reactivity of silenes toward RNC and CO has been previously reported [16, 17, 62]. Upon the coordination intermediate **INT8** forms, followed by a ring expansion via incorporation of the XylNC, resulting in the five‐membered ring zwitterionic species **INT10**. Details regarding the electronic structure of **INT10** are given in Figure S12. The electrophilic silylium center in **INT10**, in the absence of an external nucleophile, reacts with the aromatic C–C bond of the aryl substituent, dearomatizing it, and forming the compound **5a**. This species can be viewed as a masked zwitterionic silylium **INT10**, which can form in the **INT10** ⇆ **5a** equilibrium, with a low barrier.

**FIGURE 6:**
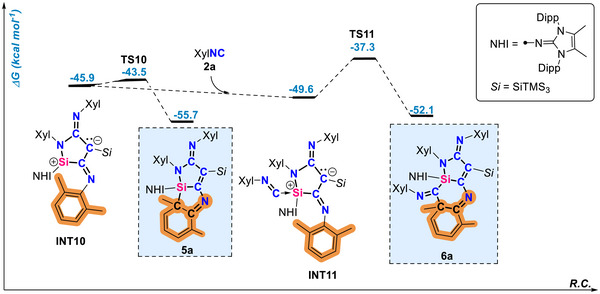
Free energy reaction coordinate diagram for **INT10** ⇆ **5a** ⇆ **INT11** ⇆ **6a** equilibrium in the presence of XylNC **2a** at the (SMD = Benzene)PW6B95‐D4/def2‐QZVPP//r^2^SCAN‐3c level of theory. Experimentally characterized compounds are highlighted in blue boxes.

Intermediate **INT10** plays an important role as it is capable of incorporating additional XylNC as well as the isoelectronic CO as will be shown later. XylNC coordinates to the silylium site of **INT10** forming intermediate **INT11**, which via a barrier of only 12.3 kcal mol^−1^ forms the product **6a**, by dearomatization of the xylyl (Figure 6). The low barriers connecting compounds **4**, **5** and **6** ensure that these species and the intermediates along the reaction pathway are at room temperature equilibrium in the presence of isocyanide.

Overall, imino(hypersilyl)silylene **1** functions as a trigger in the stepwise carbon chain growth from C_1_ → C_2_ → C_3_ → C_4_ frameworks through the consecutive formation of electrophilic intermediates, as supported by experimental evidence and DFT calculations. Distinct kinetic barriers govern the C_2_ → C_3_ → C_4_ equilibria. The barrier for the reverse conversion of C_3_ to C_2_ (i.e. **TS8**) is 28.3 kcal mol^−1^ and this step is endergonic by 4.3 kcal mol^−1^. Thus, once **5a** is formed, significant heating would be required to drive the reverse reaction and regenerate **4a**. In contrast, reaction of C_3_ with an additional nucleophile to form the C_4_ compound **6a** proceeds with a substantially lower barrier of 18.4 kcal mol^−^^1^ (**TS11**). The overall C_3_ → C_4_ transformation is slightly endergonic and therefore, an excess of RNC was required to drive the equilibrium toward formation and isolation of **6a**. Thes transformations represents rare examples of controlled homocoupling of unsaturated C1 feedstocks. In addition, the presented mechanistic study may provide insights into the relevant F‐T process and guide the development of more efficient catalysts based on main‐group elements.

### 2.2 Heterocoupling of Aryl Isocyanides and CO

**FIGURE 7:**
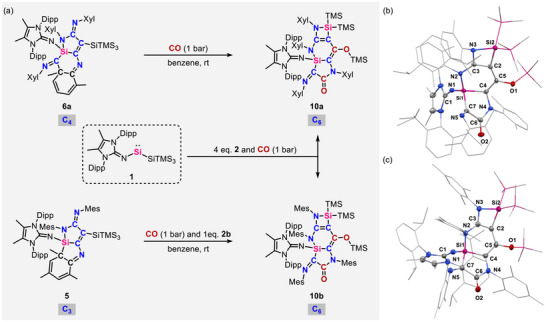
(a) Homocoupling of CO by **6a** and **5** and one pot reaction of heterocoupling of aryl isocyanides and CO mediated by **1**; Molecular structures of **10a** (b) and **10b** (c) [58]. Ellipsoids set at 50% probability to compensate for advanced thermal motion. Hydrogen atoms are omitted for clarity.

Alongside CO and RNC homocoupling, interest in the development of CO/RNC heterocoupling processes has also grown in recent years as the cross‐coupled products contain oxygen and nitrogen functionalities in the carbon framework, which is commonly found in natural products [63, 64]. However, the heterocoupling reactions of CO and RNC are rare, and most of the examples are limited to a single CO and RNC unit, restricting further reactivity and expansion of functionalized carbon framework [16, 63, 64, 65, 66]. The unusual type of RNC homologation by **1** afforded dearomative products **5** and **6**, promoting us to explore their potential for rearomatization and cross‐coupling with CO. Thus, exposing a benzene solution of **6a** to CO (1 bar) at room temperature resulted in the swift color change from orange to deep brown. The hetero‐coupled product **10a** can be isolated as brown crystals by recrystallization from pentane solution at −30°C (Figures 7a and S9.1–S9.3). The ^29^Si NMR spectrum of **10a** features four signals at 13.9, 10.3, −9.9, and −11.7 ppm, indicating the splitting of the hypersilyl group (Figure S9.3). Subsequent SC‐XRD analysis revealed the molecular structure of a new rearomative silaazatricycle **10a**, which was formed by the homologation of CO by **6a** and simultaneous TMS migration from the hypersilyl group to the CO moiety (Figure 7b and Table S1). Following the successful CO homologation by **6a**, the reaction of **5** with CO was subsequently examined. Thus, we found that exposing a benzene suspension of **5** to CO (1 bar) in the presence of another equivalent of **2b** at room temperature, gave rise to the selective formation of hetero‐coupled product **10b**, characterized by SC‐XRD analysis (Figures 7a,c and S10.1 and Table S1). Notably, silaazatricycles **10** can be synthesized via one‐pot reaction by combining **1** and four equivalents of **2a** or **2b** under CO atmosphere (1 bar). It also represents a rare example of multiple heterocoupling between CO and isocyanides, which provides a new strategy in the synthesis of both imino‐ and acyl‐functional molecules. However, attempts to functionalize **10** with halide reagents, including bromoform, silicon halides, and boron halides, were unsuccessful, resulting either in no observable reaction or in the formation of ill‐defined mixtures.

**FIGURE 8:**
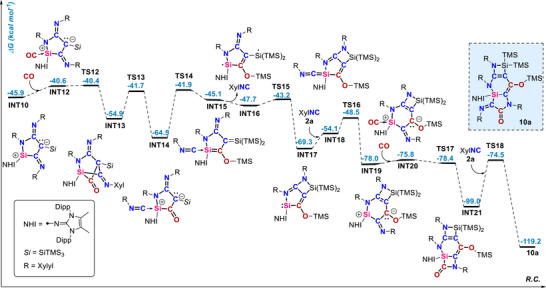
Free energy reaction coordinate diagram for XylNC/CO heterocoupling to form **10a** at the (SMD = Benzene)PW6B95‐D4/def2‐QZVPP//r^2^SCAN‐3c level of theory. Experimentally characterized compounds are highlighted in blue boxes.

The proposed mechanism for the formation of **10a** is presented in Figure 8. The previously discussed key intermediate **INT10**, which can form in the **5** ⇆ **INT10** equilibrium, upon the release of an isocyanide molecule, can be coordinated by a CO molecule, forming the silylium‐CO complex **INT12**, analogues to the isocyanide congener **INT11** (shown in Figure 6). In this case, however, no xylyl dearomatization occurs and the CO analogue of **6a** is not formed. Instead, **INT14** is generated in two steps. **INT14** is analogous to **INT12**, but with the positions of the CO and isocyanide units interchanged—the CO is now incorporated into the five‐membered heterocycle, whereas the isocyanide is coordinated to silylium center. **INT14** features a silyl group β to carbonyl which allows for a homo‐Brook rearrangement, i.e., a 1,3‐silyl shift from C to O, and the subsequent Si–N coupling forming **INT17** (via open shell **INT16** and **TS15**; details regarding the electronic structure of **INT17** are given in Figure S13). These steps again illustrate the utility of the hypersilyl substituent in enabling divergent reactivity pathways. In this case, the hypersilyl provides the TMS unit for an intramolecular migration, effectively sealing the CO unit within the heterocyclic framework. The previously released isocyanide can now be reincorporated into the heterocycle, forming the six‐membered zwitterionic **INT19** with a silylium ion center (details regarding the electronic structure of **INT19** are given in Figure S14). In the presence of an additional CO molecule, **INT19** is then converted into the transient silylium‐CO complex **INT20**. In **INT20,** the Si–CO bond is much weaker than that in the previously reported bis(silyl)silicon carbonyl complex [43, 44, 45]. This is evidenced by the endergonicity of the CO complexation to **INT19** (2.2 kcal mol^−1^). However, the formation of **INT20** allows for its rearrangement to **INT21**‐ a tricyclic species, which in the presence of the isocyanide present in the reaction mixture, forms the final product **10a**. The whole process of heterocoupling is landmarked by the three steps in which the two CO molecules and one isocyanide molecule are incorporated. The first CO uptake proceeds through interchange of CO and isocyanide from reactive zwitterionic silylium ion, affording a zwitterionic silylium‐isocyanide complex. The second CO incorporation occurs through dissociation and reincorporation of isocyanide, promoting intramolecular cyclization via a silylium–CO adduct. Finally, C–C cross‐coupling is accomplished by insertion of isocyanide into Si–C bond. Notably, while each of these steps has been independently established [17, 43, 44, 45, 62], our findings uncover their unique convergence within a single sequence proceeding in a cascade fashion.

## 3 Conclusion

In summary, we report the synthesis of a novel acyclic silylene featuring a methylated N‐heterocyclic imine backbone and a hypersilyl group. Notably, a subtle structural modification—replacing the supersilyl substituent with a hypersilyl group—induces a profound shift in the electronic and steric landscape of the silylene center, thereby unlocking an entirely new manifold of reactivity. That is, these unique properties allow silylene to act as an efficient initiator for the coupling of isocyanides and CO, affording silaazatricycles bearing both acyl and imino functional groups via stepwise carbon chain growth from C_1_ → C_2_ → C_3_ → C_4_ → C_6_ units. Complementary experimental studies and quantum chemical calculations unveil a mechanistic pathway involving the consecutive generation of electrophilic centers through silyl migration, de‐ and re‐aromatization processes, which drives a cascade homo‐ and hetero‐coupling of the isocyanides and CO. This silylene‐mediated mechanistic study of homo‐ and hetero‐coupling reactions between isocyanides CO provides deeper insight into processes relevant to the F–T reaction and may help guide the development of more efficient and selective catalytic systems based on earth‐abundant elements. In addition, the generation of silaazacycles with diverse functional groups highlights a promising synthetic strategy for accessing silicon‐based bioactive frameworks from simple silylene and readily available unsaturated C1 feedstocks.

## Conflicts of Interest

The authors declare no conflict of interest.

## Supporting information




**Supporting File 1**: anie71544‐sup‐0001‐SuppMat.pdf.

## Data Availability

The data that support the findings of this study are available in the Supporting Information of this article.

## References

[1] B. H. Davis , Advances in Fischer‐Tropsch Synthesis, Catalysts, and Catalysis, (CRC Press), 2009.

[2] M. L. Occelli , Fischer‐Tropsch Synthesis, Catalysts, and Catalysis, (CRC Press), 2016.

[3] J. A. Labinger , “Approaches to Homogeneously Catalyzed CO Hydrogenation: A Personal Retrospective,” Journal of Organometallic Chemistry 847 (2017): 4–12, 10.1016/j.jorganchem.2017.02.007.

[4] J. van de Loosdrecht , F. G. Botes , and I. M. Ciobica , Comprehensive Inorganic Chemistry II, ed. J. Reedijk and K. Poeppelmeier (Elsevier, 2013), 525–557.

[5] C. R. Cahoon and C. W. Bielawski , “Metal‐promoted C1 Polymerizations,” Coordination Chemistry Reviews 374 (2018): 261–278, 10.1016/j.ccr.2018.06.017.

[6] S. Fujimori and S. Inoue , “Main Group Carbonyl Complexes,” Communications Chemistry 3 (2020): 175, 10.1038/s42004-020-00423-9. 36703371 PMC9814907

[7] R. Y. Kong and M. R. Crimmin , “Cooperative Strategies for CO Homologation,” Dalton Transactions 49 (2020): 16587–16597, 10.1039/D0DT01564D. 32530017

[8] S. Fujimori and S. Inoue , “Carbon Monoxide in Main‐Group Chemistry,” Journal of the American Chemical Society 144 (2022): 2034–2050, 10.1021/jacs.1c13152. 35068141

[9] S. Mukhopadhyay , A. G. Patro , R. S. Vadavi , and S. Nembenna , “Coordination Chemistry of Main Group Metals With Organic Isocyanides,” European Journal of Inorganic Chemistry 2022 (2022): e202200469, 10.1002/ejic.202200469.

[10] T. Shima and Z. Hou , “Hydrogenation of Carbon Monoxide by Tetranuclear Rare Earth Metal Polyhydrido Complexes. Selective Formation of Ethylene and Isolation of Well‐Defined Polyoxo Rare Earth Metal Clusters,” Journal of the American Chemical Society 128 (2006): 8124–8125, 10.1021/ja062348l. 16787062

[11] S. Li , J. Cheng , Y. Chen , M. Nishiura , and Z. Hou , “Rare Earth Metal Boryl Complexes: Synthesis, Structure, and Insertion of a Carbodiimide and Carbon Monoxide,” Angewandte Chemie International Edition 50 (2011): 6360–6363, 10.1002/anie.201101107. 21574226

[12] R. Lalrempuia , C. E. Kefalidis , S. J. Bonyhady , et al., “Activation of CO by Hydrogenated Magnesium(I) Dimers: Sterically Controlled Formation of Ethenediolate and Cyclopropanetriolate Complexes,” Journal of the American Chemical Society 137 (2015): 8944–8947, 10.1021/jacs.5b06439. 26135846

[13] M. Majumdar , I. Omlor , C. B. Yildiz , A. Azizoglu , V. Huch , and D. Scheschkewitz , “Reductive Cleavage of Carbon Monoxide by a Disilenide,” Angewandte Chemie International Edition 54 (2015): 8746–8750, 10.1002/anie.201503455. 26088688

[14] B. Wang , X. Kang , M. Nishiura , Y. Luo , and Z. Hou , “Isolation, Structure, and Reactivity of a Scandium Boryl Oxycarbene Complex,” Chemical Science 7 (2016): 803–809, 10.1039/C5SC03138A. 28966773 PMC5580044

[15] A. V. Protchenko , P. Vasko , D. C. H. Do , et al., “Reduction of Carbon Oxides by an Acyclic Silylene: Reductive Coupling of CO,” Angewandte Chemie International Edition 58 (2019): 1808–1812, 10.1002/anie.201812675. 30537262

[16] Y. Wang , A. Kostenko , T. J. Hadlington , M.‐P. Luecke , S. Yao , and M. Driess , “Silicon‐Mediated Selective Homo‐ and Heterocoupling of Carbon Monoxide,” Journal of the American Chemical Society 141 (2019): 626–634, 10.1021/jacs.8b11899. 30516372

[17] L. Zhu , J. Zhang , H. Yang , and C. Cui , “Synthesis of Silaketenimine Anion and Its Coupling With Isocyanide,” Journal of the American Chemical Society 141 (2019): 19600–19604, 10.1021/jacs.9b11913. 31800229

[18] Z. Güven , L. Denker , D. Wullschläger , J. Pablo Martínez , B. Trzaskowski , and R. Frank , “Reductive Al−B σ‐Bond Formation in Alumaboranes: Facile Scission of Polar Multiple Bonds,” Angewandte Chemie International Edition 61 (2022): e202209502. 35947518 10.1002/anie.202209502 PMC9826004

[19] W. Yang , A. J. P. White , and M. R. Crimmin , “Deoxygenative Coupling of CO With a Tetrametallic Magnesium Hydride Complex,” Angewandte Chemie International Edition 63 (2024): e202319626, 10.1002/anie.202319626. 38348749 PMC11497278

[20] Y. Xiong , S. Yao , and M. Driess , “Striking Reactivity of a Stable, Zwitterionic Silylene Toward Substituted Diazomethanes, Azides, and Isocyanides,” Chemistry–A European Journal 15 (2009): 8542–8547, 10.1002/chem.200901337. 19610003

[21] B. Wang , G. Luo , M. Nishiura , Y. Luo , and Z. Hou , “Cooperative Trimerization of Carbon Monoxide by Lithium and Samarium Boryls,” Journal of the American Chemical Society 139 (2017): 16967–16973, 10.1021/jacs.7b10108. 29083923

[22] K. Yuvaraj , I. Douair , A. Paparo , L. Maron , and C. Jones , “Reductive Trimerization of CO to the Deltate Dianion Using Activated Magnesium(I) Compounds,” Journal of the American Chemical Society 141 (2019): 8764–8768, 10.1021/jacs.9b04085. 31096751

[23] M. J. Evans , M. D. Anker , C. L. McMullin , and M. P. Coles , “Controlled Reductive C–C Coupling of Isocyanides Promoted by an Aluminyl Anion,” Chemical Science 14 (2023): 6278–6288, 10.1039/D3SC01387A. 37325153 PMC10266456

[24] Q. Zhuo , J. Yang , X. Zhou , T. Shima , Y. Luo , and Z. Hou , “Dinitrogen Cleavage and Multicoupling With Isocyanides in a Dititanium Dihydride Framework,” Journal of the American Chemical Society 146 (2024): 10984–10992, 10.1021/jacs.4c02905. 38578866

[25] O. T. Summerscales , F. G. N. Cloke , P. B. Hitchcock , J. C. Green , and N. Hazari , “Reductive Cyclotetramerization of CO to Squarate by a U(III) Complex: The X‐ray Crystal Structure of [(U (η‐C_8_H_6_{Si^i^Pr_3_‐1,4}_2_)(η‐C_5_Me_4_H)]_2_(μ‐η^2^: η^2^‐C_4_O_4_),” Journal of the American Chemical Society 128 (2006): 9602–9603, 10.1021/ja063222r. 16866493

[26] H. Braunschweig , T. Dellermann , R. D. Dewhurst , et al., “Metal‐Free Binding and Coupling of Carbon Monoxide at a Boron–Boron Triple Bond,” Nature Chemistry 5 (2013): 1025–1028, 10.1038/nchem.1778. 24256866

[27] R. Y. Kong and M. R. Crimmin , “Carbon Chain Growth by Sequential Reactions of CO and CO_2_ With [W(CO)_6_] and an Aluminum(I) Reductant,” Journal of the American Chemical Society 140 (2018): 13614–13617, 10.1021/jacs.8b09761. 30351139

[28] R. Y. Kong , M. Batuecas , and M. R. Crimmin , “Reactions of Aluminium(i) With Transition Metal Carbonyls: Scope, Mechanism, and Selectivity of CO Homologation,” Chemical Science 12 (2021): 14845–14854, 10.1039/D1SC04940B. 34820100 PMC8597845

[29] K. Gour , D. Pramanik , S. Dash , et al., “Germylene Mediated Reductive C–C and C‒N Coupling of an Isocyanide and Its Device Application,” Angewandte Chemie International Edition 64 (2025): e202417052. 39365021 10.1002/anie.202417052

[30] W. J. Evans , J. W. Grate , L. A. Hughes , H. Zhang , and J. L. Atwood , “Reductive Homologation of Carbon Monoxide to a Ketenecarboxylate by a Low‐Valent Organolanthanide Complex: Synthesis and X‐Ray Crystal Structure of [(C_5_Me_5_)_4_Sm_2_(O_2_CCCO)(THF)]_2_ ,” Journal of the American Chemical Society 107 (1985): 3728–3730, 10.1021/ja00298a060.

[31] T. Watanabe , Y. Ishida , T. Matsuo , and H. Kawaguchi , “Reductive Coupling of Six Carbon Monoxides by a Ditantalum Hydride Complex,” Journal of the American Chemical Society 131 (2009): 3474–3475, 10.1021/ja9007276. 19243097

[32] J. Shen , G. P. A. Yap , and K. H. Theopold , “Chromium Mediated Reductive Coupling of Isonitrile Forms Unusual Heterocycles,” Journal of the American Chemical Society 136 (2014): 3382–3384, 10.1021/ja501291p. 24552240

[33] B. M. Kriegel , R. G. Bergman , and J. Arnold , “Nitrene Metathesis and Catalytic Nitrene Transfer Promoted by Niobium Bis(imido) Complexes,” Journal of the American Chemical Society 138 (2016): 52–55, 10.1021/jacs.5b11287. 26698833

[34] A. Paparo , K. Yuvaraj , A. J. R. Matthews , I. Douair , L. Maron , and C. Jones , “Reductive Hexamerization of CO Involving Cooperativity Between Magnesium(I) Reductants and [Mo(CO)_6_ ]: Synthesis of Well‐Defined Magnesium Benzenehexolate Complexes**,” Angewandte Chemie International Edition 60 (2021): 630–634, 10.1002/anie.202009523. 32969564

[35] M. J. Evans , M. G. Gardiner , M. D. Anker , and M. P. Coles , “Extending Chain Growth Beyond C_1_ → C_4_ in CO Homologation: Aluminyl Promoted Formation of the [C_5_O_5_]^5−^ Ligand,” Chemical Communications 58 (2022): 5833–5836, 10.1039/D2CC01554D. 35452064

[36] A. Heilmann , M. M. D. Roy , A. E. Crumpton , et al., “Coordination and Homologation of CO at Al(I): Mechanism and Chain Growth, Branching, Isomerization, and Reduction,” Journal of the American Chemical Society 144 (2022): 12942–12953, 10.1021/jacs.2c05228. 35786888 PMC9348839

[37] C. Zhang , F. Dankert , Z. Jiang , B. Wang , D. Munz , and J. Chu , “Evidence for Carbene Intermediates in Isocyanide Homologation by Aluminium(I),” Angewandte Chemie International Edition 62 (2023): e202307352, 10.1002/anie.202307352. 37319123

[38] S. Fujimori and S. Inoue , “Small Molecule Activation by Two‐Coordinate Acyclic Silylenes,” European Journal of Inorganic Chemistry 2020 (2020): 3131–3142, 10.1002/ejic.202000479. 32999589 PMC7507849

[39] T. J. Hadlington , “Heavier Tetrylene‐ and Tetrylyne‐Transition Metal Chemistry: It's no Carbon Copy,” Chemical Society Reviews 53 (2024): 9738–9831, 10.1039/D3CS00226H. 39230570 PMC11373607

[40] N. Takeda , H. Suzuki , N. Tokitoh , R. Okazaki , and S. Nagase , “Reaction of a Sterically Hindered Silylene With Isocyanides: The First Stable Silylene−Lewis Base Complexes,” Journal of the American Chemical Society 119 (1997): 1456–1457, 10.1021/ja963092u.

[41] T. Abe , T. Iwamoto , C. Kabuto , and M. Kira , “Synthesis, Structure, and Bonding of Stable Dialkylsilaketenimines,” Journal of the American Chemical Society 128 (2006): 4228–4229, 10.1021/ja057917o. 16568988

[42] K. Takeuchi , M. Ichinohe , and A. Sekiguchi , “Reactivity of the Disilyne RSi≡SiR (R = Si^i^Pr[CH(SiMe_3_)_2_]_2_) Toward Silylcyanide: Two Pathways to Form the Bis‐Adduct [RSiSiR(CNSiMe_3_)_2_] With Some Silaketenimine Character and a 1,4‐Diaza‐2,3‐disilabenzene Analogue,” Journal of the American Chemical Society 130 (2008): 16848–16849, 10.1021/ja807974a. 19053404

[43] C. Ganesamoorthy , J. Schoening , C. Wölper , L. Song , P. R. Schreiner , and S. Schulz , “A Silicon–Carbonyl Complex Stable at Room Temperature,” Nature Chemistry 12 (2020): 608–614, 10.1038/s41557-020-0456-x. 32313239

[44] D. Reiter , R. Holzner , A. Porzelt , P. Frisch , and S. Inoue , “Silylated Silicon–Carbonyl Complexes as Mimics of Ubiquitous Transition‐Metal Carbonyls,” Nature Chemistry 12 (2020): 1131–1135, 10.1038/s41557-020-00555-4. 33071286

[45] Y. Ding , J. Zhang , Y. Li , and C. Cui , “Disilicon Dicarbonyl Complex: Synthesis and Protonation of CO With O–H Bond,” Journal of the American Chemical Society 144 (2022): 20566–20570, 10.1021/jacs.2c10599. 36342481

[46] Y. Xiong , S. Yao , T. Szilvási , A. Ruzicka , and M. Driess , “Homocoupling of CO and Isocyanide Mediated by a C,C′‐bis(silylenyl)‐substituted Ortho‐Carborane,” Chemical Communications 56 (2020): 747–750, 10.1039/C9CC08680C. 31845675

[47] D. Wendel , T. Szilvási , C. Jandl , S. Inoue , and B. Rieger , “Twist of a Silicon–Silicon Double Bond: Selective Anti ‐Addition of Hydrogen to an Iminodisilene,” Journal of the American Chemical Society 139 (2017): 9156–9159, 10.1021/jacs.7b05335. 28640616

[48] F. J. Kiefer , A. Kostenko , R. Holzner , and S. Inoue , “Reversible CO Insertion Into the Si = Si Double Bond Enables a Disila‐Bislactone Formation via Subsequent CO_2_ Addition,” Journal of the American Chemical Society 147 (2025): 26663–26673, 10.1021/jacs.5c07040. 40685932 PMC12314906

[49] D. Wendel , A. Porzelt , F. A. D. Herz , et al., “From Si(II) to Si(IV) and Back: Reversible Intramolecular Carbon–Carbon Bond Activation by an Acyclic Iminosilylene,” Journal of the American Chemical Society 139 (2017): 8134–8137, 10.1021/jacs.7b05136. 28587448

[50] D. Wendel , D. Reiter , A. Porzelt , P. J. Altmann , S. Inoue , and B. Rieger , “Silicon and Oxygen's Bond of Affection: An Acyclic Three‐Coordinate Silanone and Its Transformation to an Iminosiloxysilylene,” Journal of the American Chemical Society 139 (2017): 17193–17198, 10.1021/jacs.7b10634. 29098861

[51] H. Zhu , A. Kostenko , D. Franz , F. Hanusch , and S. Inoue , “Room Temperature Intermolecular Dearomatization of Arenes by an Acyclic Iminosilylene,” Journal of the American Chemical Society 145 (2023): 1011–1021, 10.1021/jacs.2c10467. 36597967

[52] H. Zhu , A. Kostenko , J. A. Kelly , and S. Inoue , “Substituent Exchange Between an Imino(silyl)Silylene and Aryl Isocyanides,” Chemistry 10 (2024): 1213–1224, 10.1016/j.chempr.2024.01.007.

[53] D. Reiter , R. Holzner , A. Porzelt , P. J. Altmann , P. Frisch , and S. Inoue , “Disilene–Silylene Interconversion: A Synthetically Accessible Acyclic Bis(silyl)Silylene,” Journal of the American Chemical Society 141 (2019): 13536–13546, 10.1021/jacs.9b05318. 31352777

[54] T. Eisner , A. Kostenko , F. Hanusch , and S. Inoue , “Room‐Temperature‐Observable Interconversion Between Si(IV) and Si(II) via Reversible Intramolecular Insertion Into an Aromatic C−C Bond,” Chemistry–A European Journal 28 (2022): e202202330, 10.1002/chem.202202330. 36098491 PMC10092829

[55] M. E. Doleschal , A. Kostenko , J. Y. Liu , and S. Inoue , “Isolation of a NHC‐Stabilized Heavier Nitrile and Its Conversion Into an Isonitrile Analogue,” Nature Chemistry 16 (2024): 2009–2016, 10.1038/s41557-024-01618-6. PMC11611736 39256544

[56] M. M. D. Roy , M. J. Ferguson , R. McDonald , Y. Zhou , and E. Rivard , “A Vinyl Silylsilylene and Its Activation of Strong Homo‐ and Heteroatomic Bonds,” Chemical Science 10 (2019): 6476–6481, 10.1039/C9SC01192G. 31341599 PMC6610552

[57] K. Gour , M. K. Bisai , and S. S. Sen , “The Hypersilyl Substituent in Heavier Low‐Valent Group 14 Chemistry,” European Journal of Inorganic Chemistry 2022 (2022): e202200071, 10.1002/ejic.202200071.

[58] Deposition numbers 2498260 (for **1**), 2498261 (for **3a**), 2498262 (for **4a**), 2498263 (for **5**), 2498264 (for **6a**), 2498265 (for **6b**), 2498266 (for **10a**), and 2498267 (for **10b**) contain the supplementary crystallographic data for this paper. These data are provided free of charge by the joint Cambridge Crystallographic Data Centre and Fachinformationszentrum Karlsruhe Access Structures service.

[59] N. Wiberg , K. Schurz , and G. Fischer , “Isolation of the Stable Silaketimine t Bu_2_ Si═N─Si T Bu_3_ ,” Angewandte Chemie (International ed in English) 24 (1985): 1053–1054, 10.1002/anie.198510531.

[60] R. S. Ghadwal , H. W. Roesky , K. Pröpper , B. Dittrich , S. Klein , and G. Frenking , “A Dimer of Silaisonitrile With Two‐Coordinate Silicon Atoms,” Angewandte Chemie International Edition 50 (2011): 5374–5378, 10.1002/anie.201101320. 21574229

[61] F. Neese , “Software Update: The ORCA Program System—Version 5.0,” WIREs Computational Molecular Science 12 (2022): e1606, 10.1002/wcms.1606.

[62] Y. Kratish , D. Pinchuk , A. Kaushansky , et al., “The Reactions of Carbon Monoxide With Silyl and Silenyl Lithium—Synthesis and Isolation of the First Stable Tetra‐Silyl Di‐Ketyl Biradical and 1‐Silaallenolate Lithium,” Angewandte Chemie International Edition 58 (2019): 18849–18853, 10.1002/anie.201910336. 31591792

[63] R. N. Vrtis and S. J. Lippard , “Reductive Coupling of Carbon Monoxide and Alkyl Isocyanide Ligands in Early Transition Metal Complexes: A Review,” Israel Journal of Chemistry 30 (1990): 331–341, 10.1002/ijch.199000035.

[64] W. Yang , A. J. P. White , and M. R. Crimmin , “Cross‐coupling of CO and an Isocyanide Mediated by a Tetrameric Magnesium Hydride Cluster,” Chemical Science 15 (2024): 11807–11813, 10.1039/D4SC02638A. 39092134 PMC11290425

[65] E. M. Carnahan and S. J. Lippard , “Formation of Highly Functionalized Metal‐Bound Acetylenes by Reductive Coupling of Carbon Monoxide and Methyl Isocyanide Ligands,” Journal of the American Chemical Society 114 (1992): 4166–4174, 10.1021/ja00037a019.

[66] N. S. Radu , M. P. Engeler , C. P. Gerlach , T. D. Tilley , and A. L. Rheingold , “Isolation of the First d_0_ Metalloxy Ketene Complexes via "Double Insertion" of Carbon Monoxide Into Thorium‐Silicon Bonds,” Journal of the American Chemical Society 117 (1995): 3621–3622, 10.1021/ja00117a035.

