# Supplementary material for: Sequential Homo‐ and Hetero‐Coupling of Isocyanide and Carbon Monoxide Mediated by Acyclic Imino(silyl)Silylene
Source: Angew Chem Int Ed Engl. 2026 Feb 24;65(15):e9929509. doi: 10.1002/anie.9929509 (PMC13053907; doi:10.1002/anie.9929509)
Supplement: Supplementary file 1 — Supporting File 1: anie71544‐sup‐0001‐SuppMat.pdf. [file ANIE-65-e9929509-s001.pdf]

Sequential Homo- and Hetero-Coupling of Isocyanide and Carbon Monoxide Mediated by Acyclic Imino(silyl)silylene

Huaiyuan Zhu, Arseni Kostenko, Xufang Liu and Shigeyoshi Inoue*

Abstract: Carbon monoxide (CO) and its isoelectronic analogues, isocyanides (RNC), have been extensively employed as model substrates to elucidate elementary steps of the Fischer–Tropsch (F–T) process, particularly those governing C–C bond construction. Building on this concept, we demonstrate carbon chain growth mediated by an acyclic imino(hypersilyl)silylene through sequential reactions with CO and isocyanides, ultimately affording well-defined C6 frameworks composed of four isocyanide and two CO units. A proposed mechanism for the homo- and hetero-coupling processes, derived from experimental observations and corroborated by quantum chemical calculations, reveals that the silylene functions as an initiator, inducing the stepwise homocoupling of RNC through consecutive formation of electrophilic sites, as well as promoting heterocoupling reactions with CO.

Contents

1. Experimental Procedures 3

1.1 General Methods and Instrumentation 3

1.2 Synthesis and Characterization 4

1.2.1 Synthesis of **1** 4

1.2.2 Synthesis of **3a** 7

1.2.3 Synthesis of **3b** 11

1.2.4 Synthesis of **4a** 13

1.2.5 Synthesis of **4b** 16

1.2.6 Synthesis of **5** 19

1.2.7 Synthesis of **6a** 22

1.2.8 Synthesis of **6b** 25

1.2.9 Synthesis of **10a** 26

1.2.10 Synthesis of **10b** 30

2. Single Crystal X-Ray Structure Determination 32

3. Computational Details 36

4. Appendix: Cartesian coordinates of the optimized geometries 42

5. References 101

1. Experimental Procedures

1.1 General Methods and Instrumentation

All experiments and manipulations were carried out under argon atmosphere using standard Schlenk or glovebox techniques. The glassware was heat-dried under vacuum prior to use. All glass junctions were coated with PTFE-based grease Merkel Triboflon III. For stirring, PTFE-coated magnetic stirrer bars were used or glass-coated ones if stated. Liquid phases were transferred using standard PE/PP syringes equipped with stainless steel cannula or directly canted from vessel to vessel if not stated otherwise. Solvents were dried by standard methods (withdrawal from MBraun Solvent Purification System and storage over molecular sieves (3 Å), or distilled from sodium/ benzophenone or CaH_2 under argon atmosphere and degassed via freeze-pump-thaw cycling). All chemicals were purchased from commercial suppliers and used as received if not stated otherwise. Deuterated benzene (C_6D_6), THF-D_8 and Tol-D_8 were obtained from Deutero Deutschland GmbH and were dried over 3 Å molecular sieves. All NMR samples were prepared under argon in J. Young PTFE tubes. Carbon monoxide (5.0) were purchased from Westfalen AG and used as received. NMR spectra were recorded on a Bruker AV400US, DRX400, AVHD300 or AV500cr at ambient temperature (300 K) if not stated otherwise. ^{1}H and ^{13}C NMR spectra were calibrated against the residual proton and natural abundance carbon resonances of the respective deuterated solvent as internal standard. Elemental analyses (EA) were conducted with a EURO EA (HEKA tech) instrument equipped with a CHNS combustion analyzer. Liquid Injection Field Desorption Ionization Mass Spectrometry (LIFDI-MS) was measured directly from an inert atmosphere glovebox with a Thermo Fisher Scientific Exactive Plus Orbitrap equipped with an ion source from Linden CMS. **1** and MesNC were synthesized according to procedures described in literature.[S1-2]

1.2 Synthesis and Characterization

1.2.1 Synthesis of 1

A solution of $KSiTMS_3$ (1.64 g, 5.7 mmol) in toluene (30 mL) was added to $^{Me}IPrNSiBr_3$ (2.0 g, 2.8 mmol) in toluene (60 mL) at room temperature. The color changed to blue rapidly. The resulting mixture was stirred for 1 h, then all volatiles were removed *in vacuum*. The obtained residue was extracted with pentane (3 × 20 mL). Pentane was removed from the filtrate *in vacuum*, the resulting blue solid was washed with cold hexamethyldisiloxane (3 × 10 mL). The remaining solid was dried *in vacuum* to yield silylene **1** as intense blue powder (1.11 g, 59%). Crystal suitable for single crystal X-ray diffraction analysis was obtained by storing saturated pentane solution at –30 °C for 2 days.

^{1}H NMR (400.1 MHz, C_6D_6): δ [ppm] 7.20-7.24 (m, 2H, *p*-C*H*-Dipp), 7.11-7.13 (m, 4H, *m*-C*H*-Dipp), 3.11 (sept, *J* = 6.8 Hz, 4H, C*H*(CH_3)$_2$), 1.55 (s, 6H, NCC*H*$_3$), 1.39 (d, *J* = 6.8 Hz, 12H, CH(C*H*$_3$)$_2$), 1.12 (d, *J* = 6.8 Hz, 12H, CH(C*H*$_3$)$_2$), 0.29 (s, 27H, Si(C*H*$_3$)$_3$).

^{13}C{^{1}H} NMR (100.6 MHz, C_6D_6): δ [ppm] 155.4 (N*C*N), 147.8 (Ar*C*), 130.9 (Ar*C*), 129.6 (Ar*C*), 123.8 (Ar*C*), 118.3 (N*C*CH_3), 28.8 (*C*H(CH_3)$_2$), 24.5 (CH(*C*H$_3$)$_2$), 23.6 (CH(*C*H$_3$)$_2$), 9.5 (NC*C*H$_3$), 3.5 (Si(*C*H$_3$)$_3$).

^{29}Si{^{1}H} NMR (79.5 MHz, C_6D_6): δ [ppm] –117.1 (*Si*TMS$_3$), –9.5 (*Si*Me$_3$), 405.7 (*central Si*).

Elemental Analysis (%): Calcd: C 64.41, H 9.56, N 5.95; Found: C 63.08, H 9.05, N 5.91.

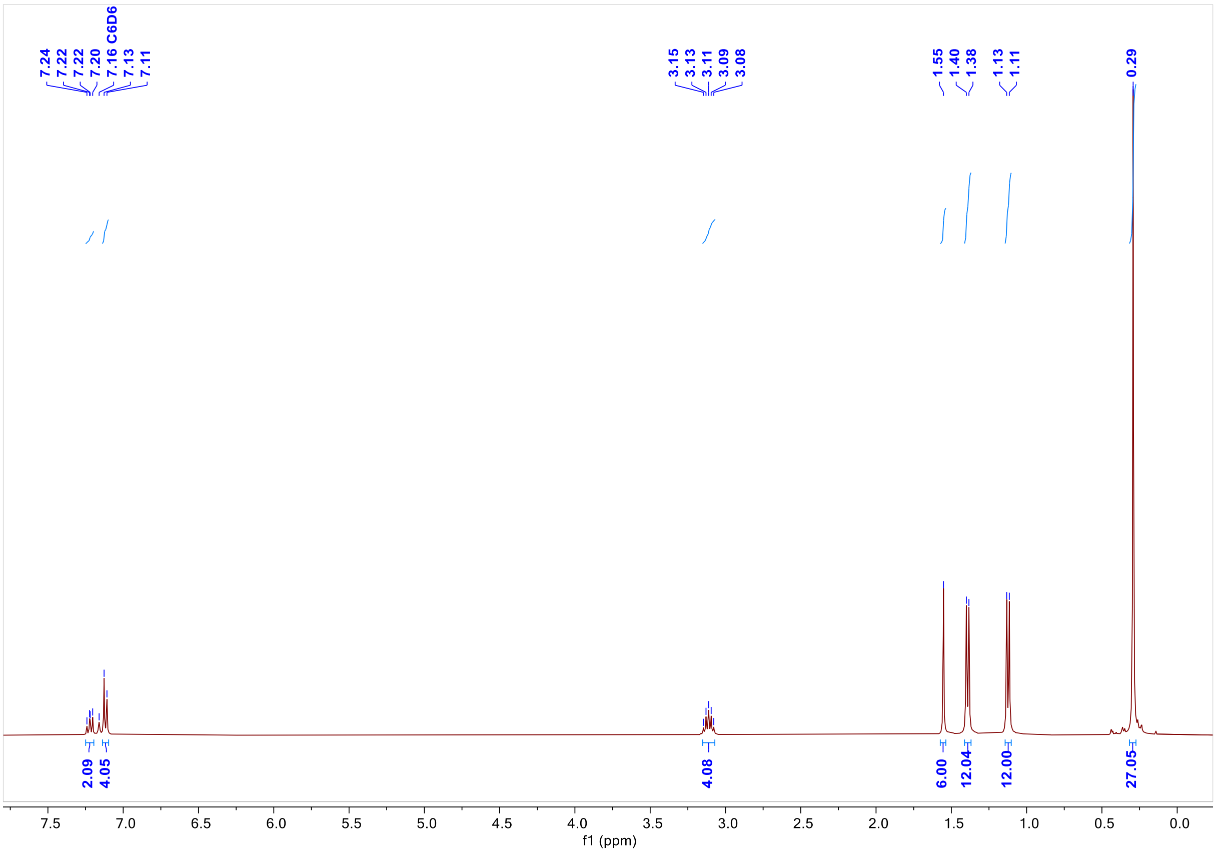

Figure S1.1. ^{1}H NMR spectrum of **1** in C_6D_6 at 300K.

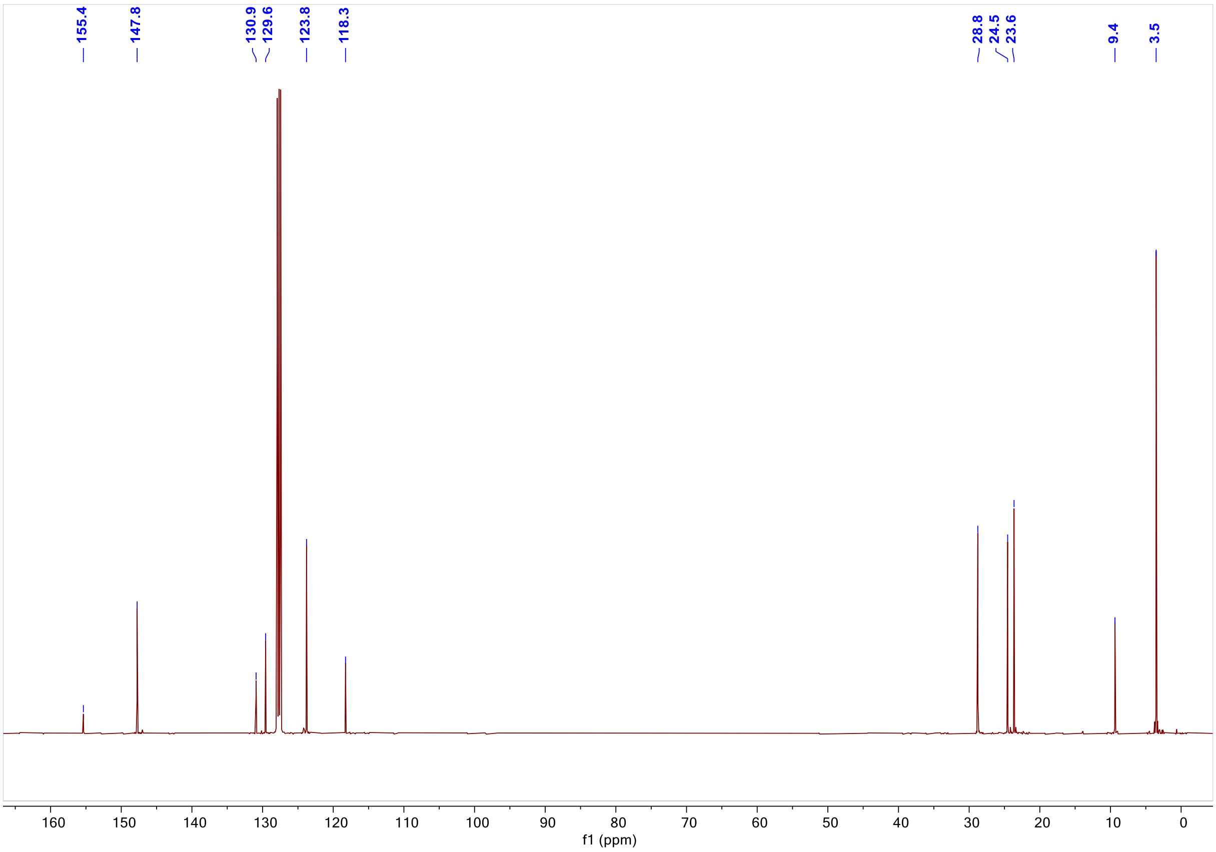

Figure S1.2. $^{13}C\{^{1}H\}$ NMR spectrum of **1** in C_6D_6 at 300K.

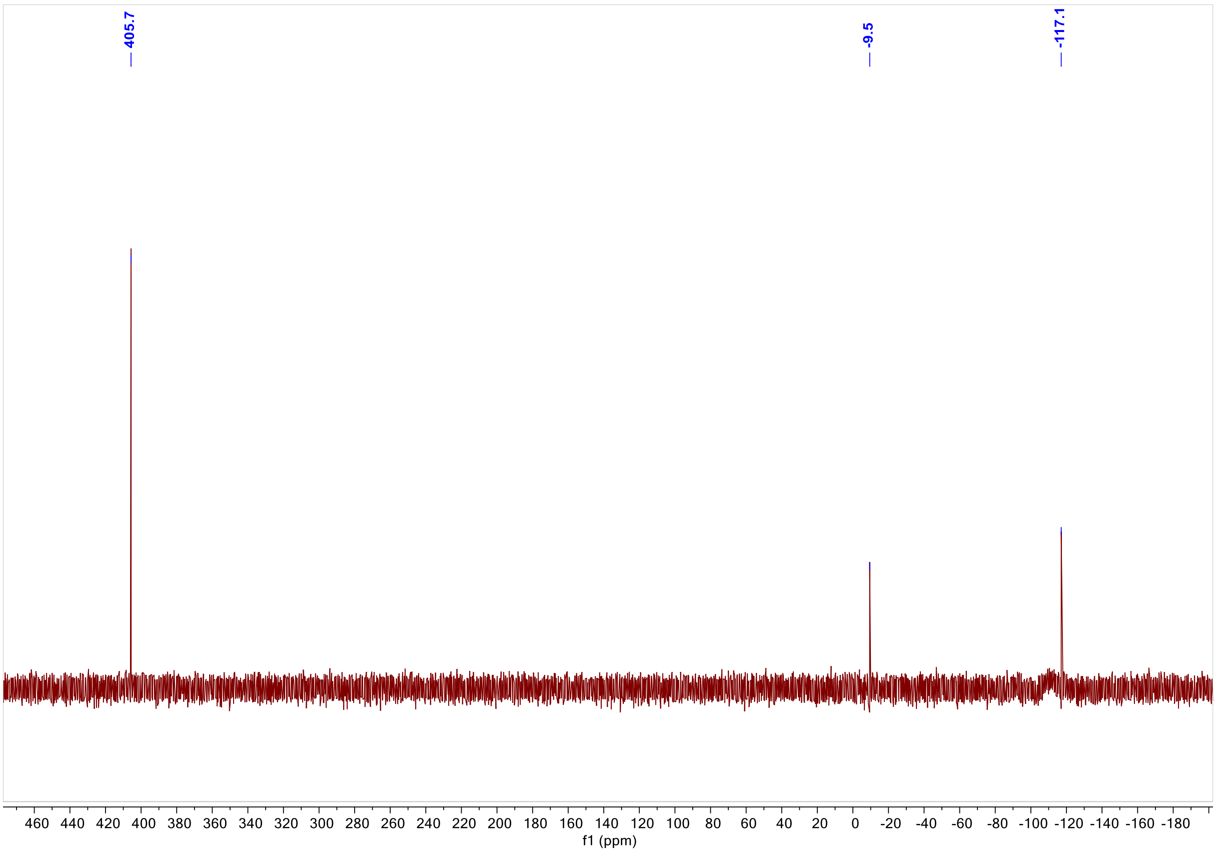

Figure S1.3. $^{29}Si\{^1H\}$ NMR spectrum of **1** in C_6D_6 at 300K.

1.2.2 Synthesis of 3a

Silylene **1** (106.0 mg, 0.15 mmol) and **2a** (39.4 mg, 0.30 mmol) were combined in benzene (3 mL) at room temperature. The color of mixture turned to deep green then to orange rapidly. After stirring at room temperature for 5 minutes, all volatiles were removed *in vacuum*, the oily residue was recrystallized from pentane at –30 °C for 2 days. The red crystals were separated by removing top orange solution, washed with pentane (3 × 1 mL) and dried *in vacuum* to yield **3a** (83.1 mg, 57%) as a red powder.

^{1}H NMR (400.1 MHz, C_6D_6): δ [ppm] 7.13-7.20 (m, 6H, Ar-*H*, overlapping with C_6D_6), 6.94-7.08 (m, 5H, Ar-*H*), 6.83-6.84 (m, 2H, Ar-*H*), 6.69-6.72 (m, 1H, Ar-*H*), 3.09 (sept, *J* = 6.4 Hz, 2H, C*H*(CH_3)$_2$), 3.07 (sept, *J* = 6.4 Hz, 2H, C*H*(CH_3)$_2$), 2.67 (s, 6H, ArC*H*$_3$), 2.19 (s, 6H, ArC*H*$_3$), 1.36 (d, *J* = 6.4 Hz, 6H, CH(C*H*$_3$)$_2$), 1.34 (d, *J* = 6.4 Hz, 6H, CH(C*H*$_3$)$_2$), 1.28 (s, 6H, NCC*H*$_3$), 1.09 (d, *J* = 6.8 Hz, 6H, CH(C*H*$_3$)$_2$), 1.08 (d, *J* = 6.8 Hz, 6H, CH(C*H*$_3$)$_2$), 0.17 (s, 27H, Si(C*H*$_3$)$_3$).

^{13}C{^{1}H} NMR (100.6 MHz, C_6D_6): δ [ppm] 193.6(C*C*N), 146.7 (N*C*N), 144.5 (Ar*C*),143.7 (Ar*C*), 142.8 (Ar*C*), 132.7 (Ar*C*), 131.2 (Ar*C*), 129.6 (Ar*C*), 129.0 (Ar*C*), 128.5 (Ar*C*), 124.8 (Ar*C*), 124.4 (Ar*C*), 123.9 (Ar*C*), 118.2 (N*C*CH$_3$), 118.1 (Ar*C*), 73.9 (*C*CN), 28.8 (*C*H(CH_3)$_2$), 28.6 (*C*H(CH_3)$_2$), 24.6 (CH(*C*H$_3$)$_2$), 24.3 (CH(*C*H$_3$)$_2$), 22.8 (CH(*C*H$_3$)$_2$), 19.8 (C*C*H$_3$), 19.8 (Ar*C*H$_3$), 10.0 (NC*C*H$_3$), 3.0 (Si(*C*H$_3$)$_3$).

^{29}Si{^{1}H} NMR (79.5 MHz, C_6D_6): δ [ppm] –9.9 (*Si*Me$_3$), –98.3 (*Si*TMS$_3$), –120.7 (*central Si*).

Elemental Analysis (%): Calcd: C 69.43, H 8.84, N 7.23; Found: C 68.36, H 8.72, N 7.33.

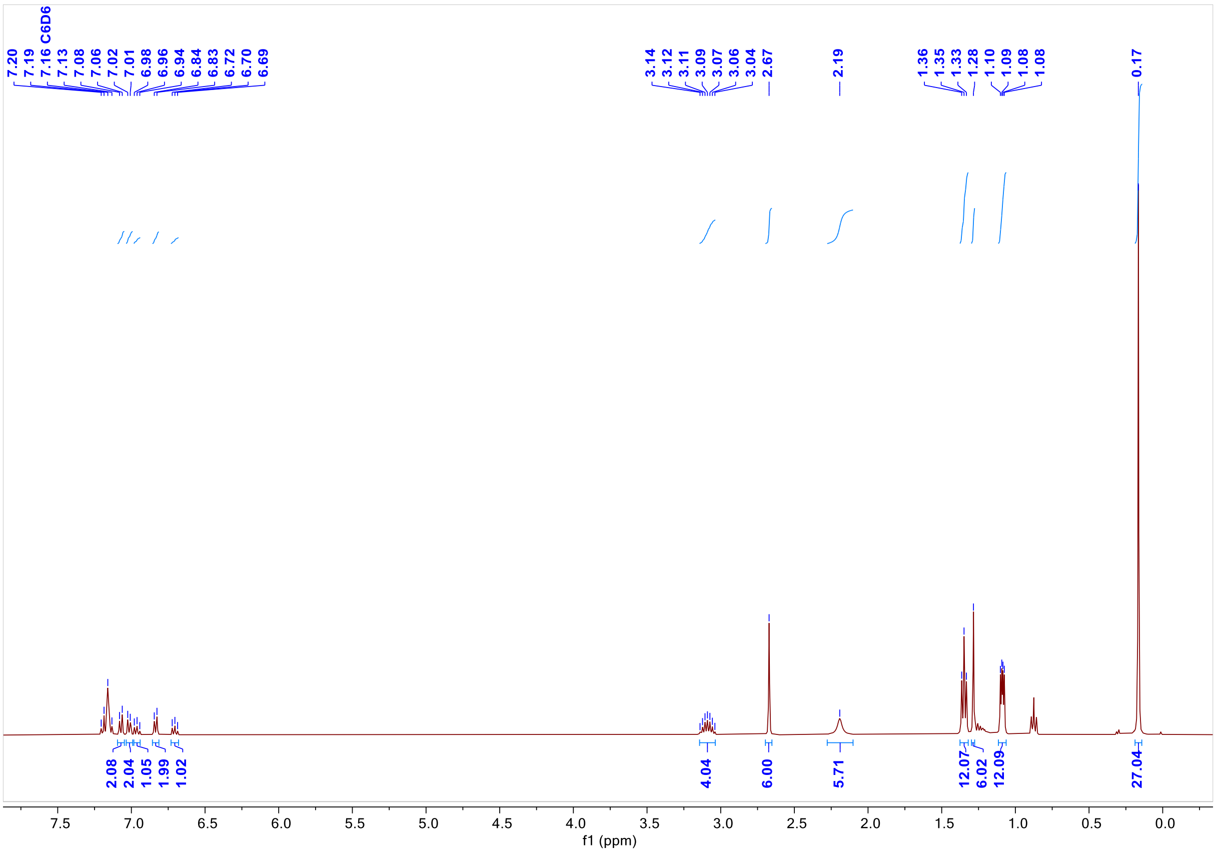

Figure S2.1. ^{1}H NMR spectrum of **3a** in C_6D_6 at 300K.

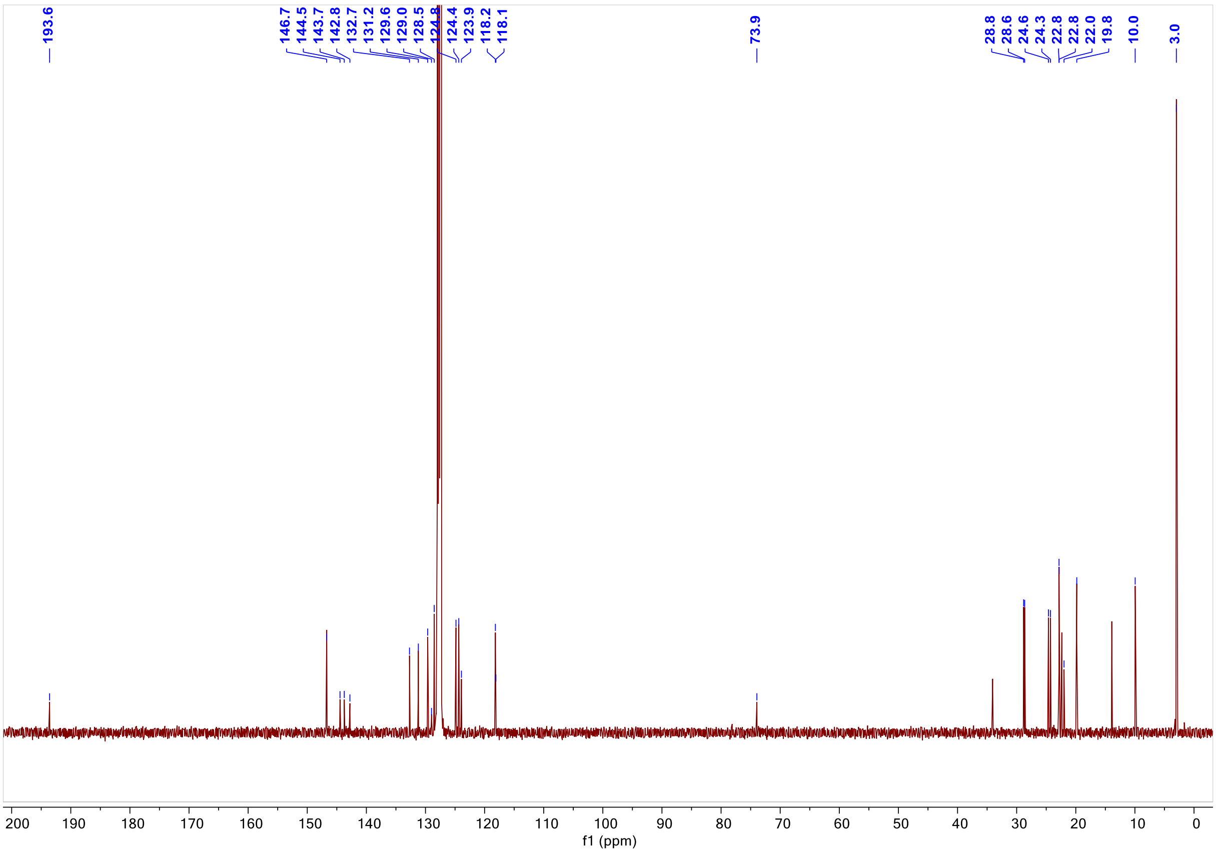

Figure S2.2. ^{13}C{^{1}H} NMR spectrum of **3a** in C_6D_6 at 300K.

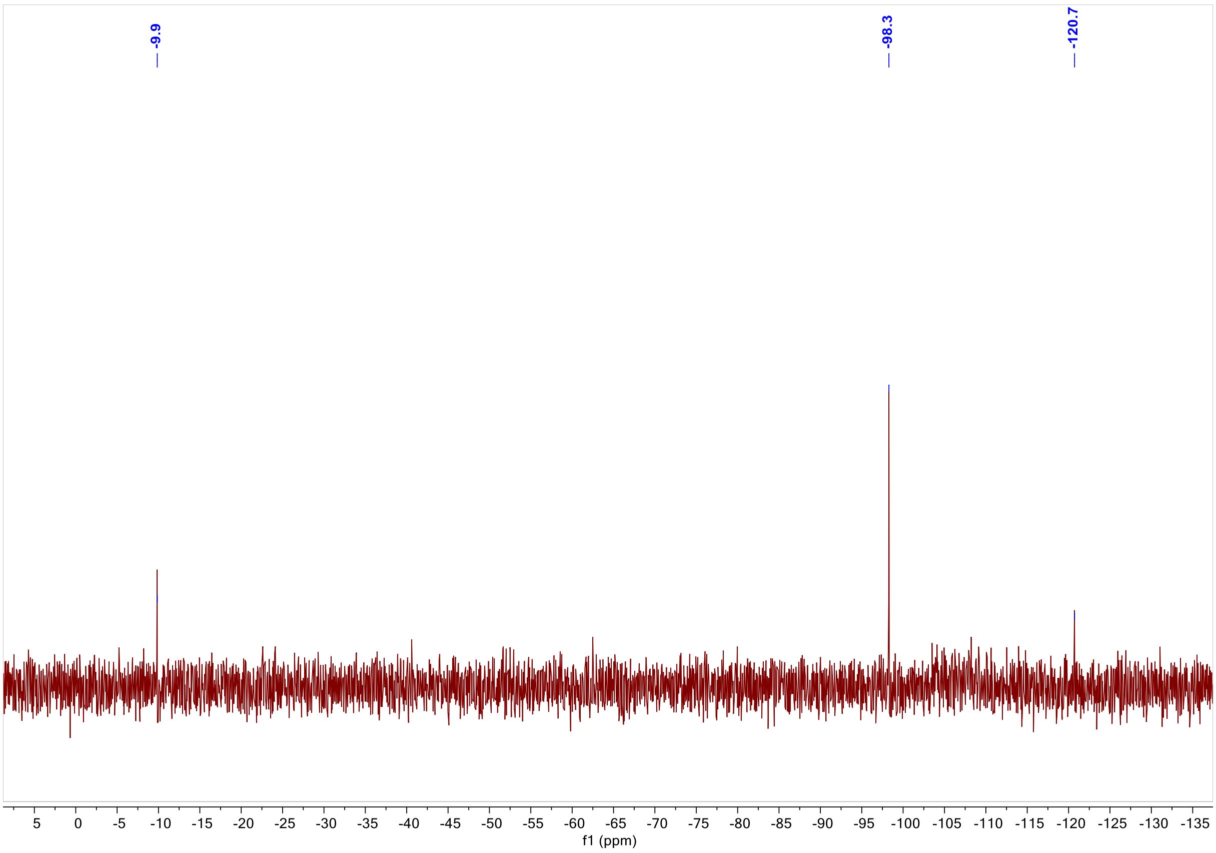

Figure S2.3. $^{29}Si\{^{1}H\}$ NMR spectrum of **3a** in C_6D_6 at 300K.

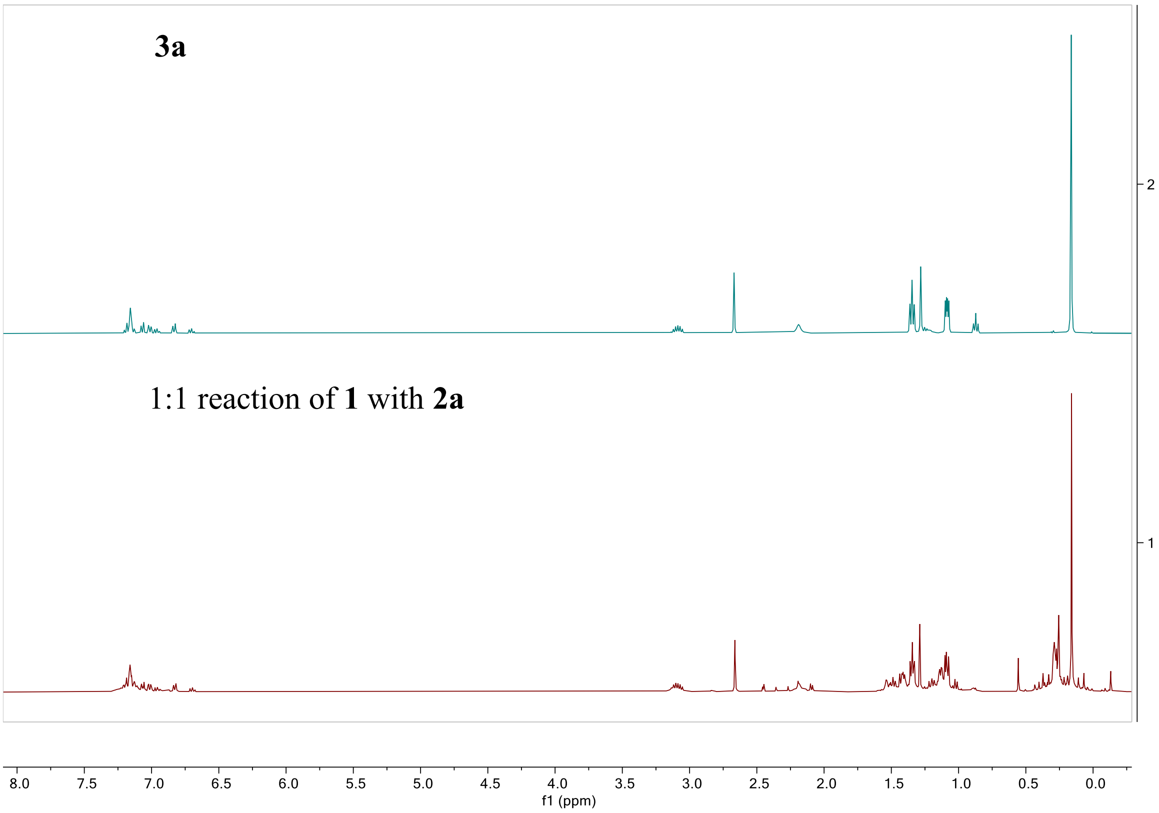

Figure S2.4. Stacked ^{1}H NMR spectrum of **3a** and the stoichiometric reaction of **1** with **2a** in C_6D_6 at 300K.

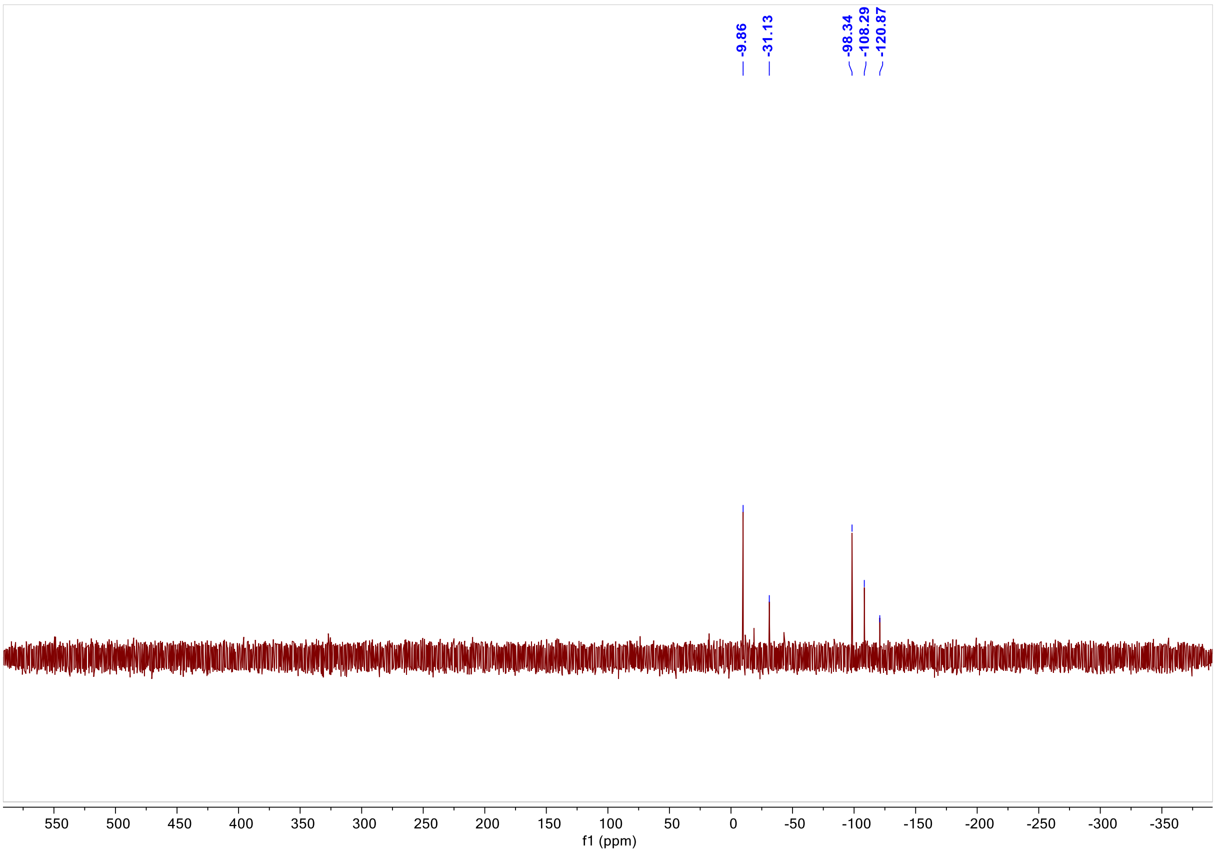

Figure S2.5. $^{29}Si\{^{1}H\}$ NMR spectrum of the stoichiometric reaction of **1** with **2a** in C_6D_6 at 300K.

1.2.3 Synthesis of 3b

Silylene **1** (53 mg, 0.075 mmol) and **2b** (21.8 mg, 0.15 mmol) were combined in C_6D_6 (0.5 mL) at room temperature. The color of mixture turned to deep green then to orange rapidly. This orange solution was subsequently monitored by ^{1}H NMR and ^{29}Si NMR spectra. The NMR parameters closely resemble those of **3a**, particularly the ^{29}Si NMR chemical shift, which strongly indicates the formation of **3b** as the predominant product. However, attempts to further purify **3b** were unsuccessful due to its rapid transformation into **4b** within a short time period, even at –30 °C.

^{1}H NMR (400.1 MHz, C_6D_6): δ [ppm] 7.16-7.22 (m, 6H, Ar-*H*, overlapping with C_6D_6), 6.88 (s, 2H, Ar-*H*), 6.85 (s, 2H, Ar-*H*),6.69-6.72 (m, 1H, Ar-*H*), 3.12 (sept, *J* = 6.4 Hz, 2H, C*H*(CH_3)$_2$), 3.10 (sept, *J* = 6.4 Hz, 2H, C*H*(CH_3)$_2$), 2.70 (s, 6H, ArC*H*$_3$), 2.22 (s, 6H, ArC*H*$_3$), 2.19 (s, 6H, ArC*H*$_3$), 1.38 (d, *J* = 6.4 Hz, 6H, CH(C*H*$_3$)$_2$), 1.37 (d, *J* = 6.4 Hz, 6H, CH(C*H*$_3$)$_2$), 1.29 (s, 6H, NCC*H*$_3$), 1.11 (d, *J* = 6.8 Hz, 6H, CH(C*H*$_3$)$_2$), 1.10 (d, *J* = 6.8 Hz, 6H, CH(C*H*$_3$)$_2$), 0.17 (s, 27H, Si(C*H*$_3$)$_3$).

^{29}Si{^{1}H} NMR (79.5 MHz, C_6D_6): δ [ppm] –9.8 (*Si*Me$_3$), –98.3 (*Si*TMS$_3$), –121.3 (*central Si*).

LIFDI-MS: Calcd: 995.5964; Found: 995.6025.

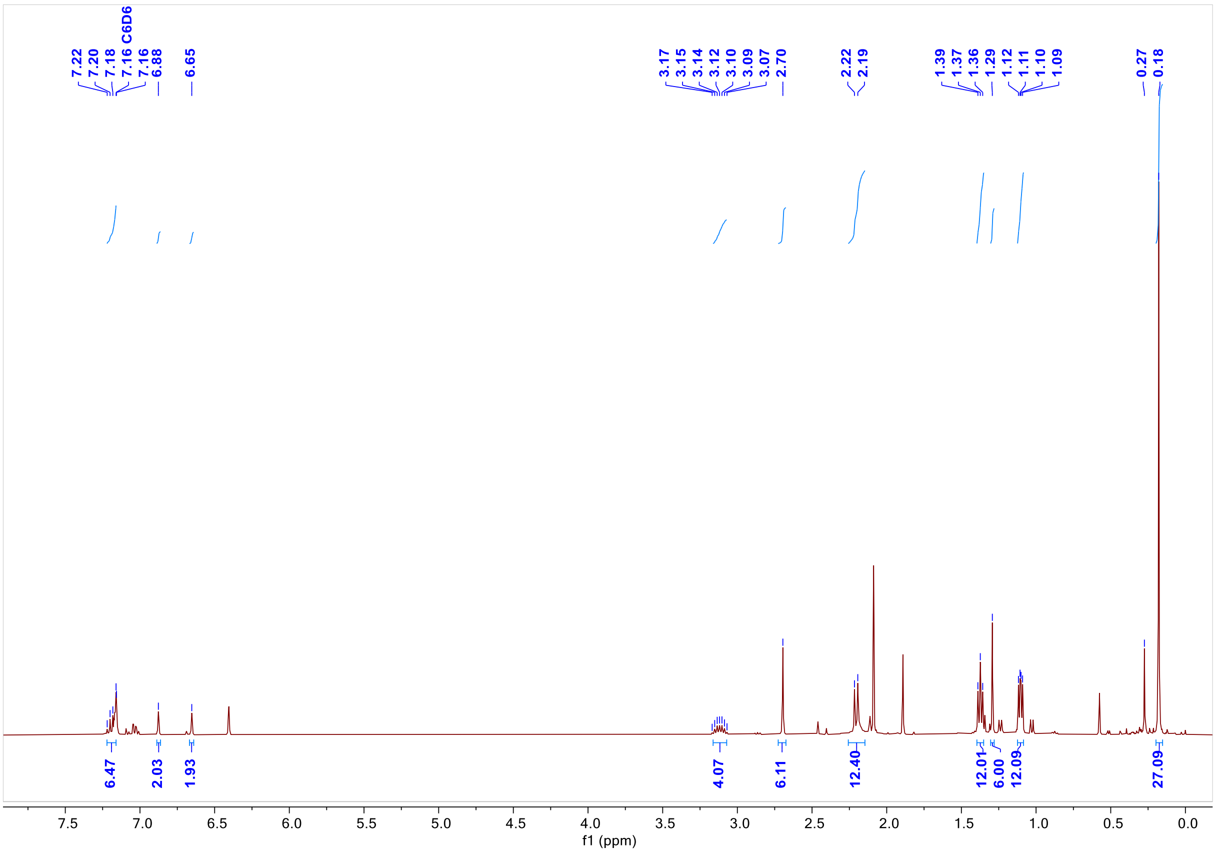

Figure S3.1. ^{1}H NMR spectrum of **3b** in C_6D_6 at 300K.

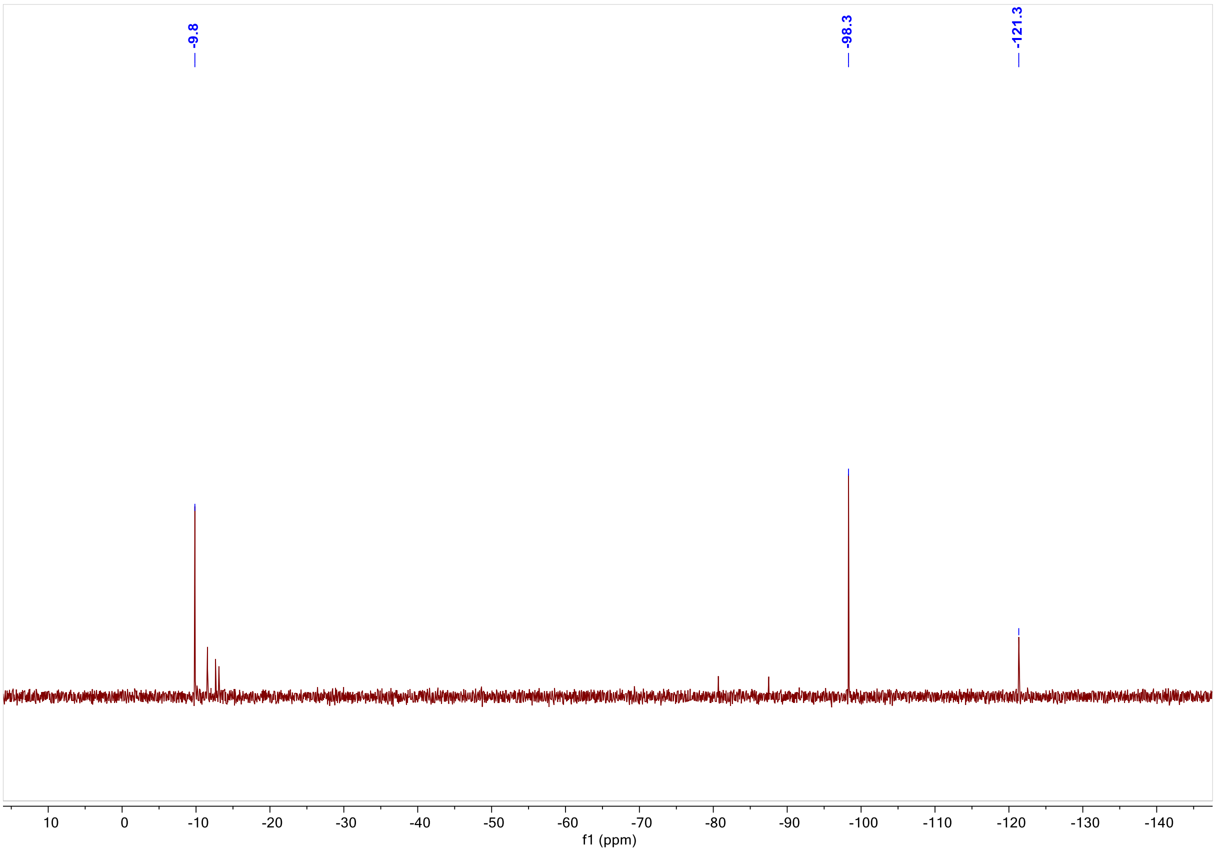

Figure S3.2. $^{29}Si\{^1H\}$ NMR spectrum of **3b** in C_6D_6 at 300K.

1.2.4 Synthesis of 4a

A benzene solution of **3a** (50 mg, 0.052 mmol) was stirred for 24 h at room temperature. The color of mixture turned to bright yellow from red gradually. All volatiles were removed *in vacuum* to yield **4a** as yellow powder quantitatively. Crystals **4a** suitable for single crystal X-ray diffraction analysis were obtained by storing saturated pentane solution at –30 °C for 2 days.

^{1}H NMR (400.1 MHz, C_6D_6): δ [ppm] 7.20-7.22 (m, 2H, Ar*H*), 7.11-7.16 (m, 2H, Ar*H*, overlapping with C_6D_6), 7.04-7.06 (m, 4H, Ar*H*), 6.85-6.92 (m, 3H, Ar*H*), 6.77-6.81 (m, 1H, Ar*H*), 2.84 (sept, *J* = 6.8 Hz, 4H, C*H*(CH_3)$_2$), 2.45 (s, 6H, ArC*H*$_3$), 2.11 (s, 6H, ArC*H*$_3$), 1.33 (s, 6H, NCC*H*$_3$), 1.21 (d, *J* = 6.8 Hz, 12H, CH(C*H*$_3$)$_2$), 1.02 (d, *J* = 6.8 Hz, 12H, CH(C*H*$_3$)$_2$), 0.26 (s, 27H, Si(C*H*$_3$)$_3$).

^{13}C{^{1}H} NMR (100.6 MHz, C_6D_6): δ [ppm] 165.9(C*C*N), 149.9 (N*C*N), 146.9 (Ar*C*),141.5 (Ar*C*), 135.8 (Ar*C*), 135.4 (Ar*C*), 131.2 (Ar*C*), 129.8 (Ar*C*), 128.6 (Ar*C*), 127.2 (Ar*C*), 126.8 (Ar*C*), 126.0 (Ar*C*), 124.4 (Ar*C*), 117.2 (N*C*CH$_3$), 114.7(Ar*C*), 28.8 (*C*H(CH_3)$_2$), 23.9 (CH(*C*H$_3$)$_2$), 23.0 (CH(*C*H$_3$)$_2$), 21.5 (Ar*C*H$_3$), 20.4 (Ar*C*H$_3$), 19.1(*C*CN), 9.5 (NC*C*H$_3$), 1.7 (Si(*C*H$_3$)$_3$).

^{29}Si{^{1}H} NMR (79.5 MHz, C_6D_6): δ [ppm] –12.0 (*Si*Me$_3$), –36.2 (*Si*=N), –73.1 (*Si*TMS$_3$).

Elemental Analysis (%): Calcd: C 69.43, H 8.84, N 7.23; Found: C 69.01, H 8.78, N 7.14.

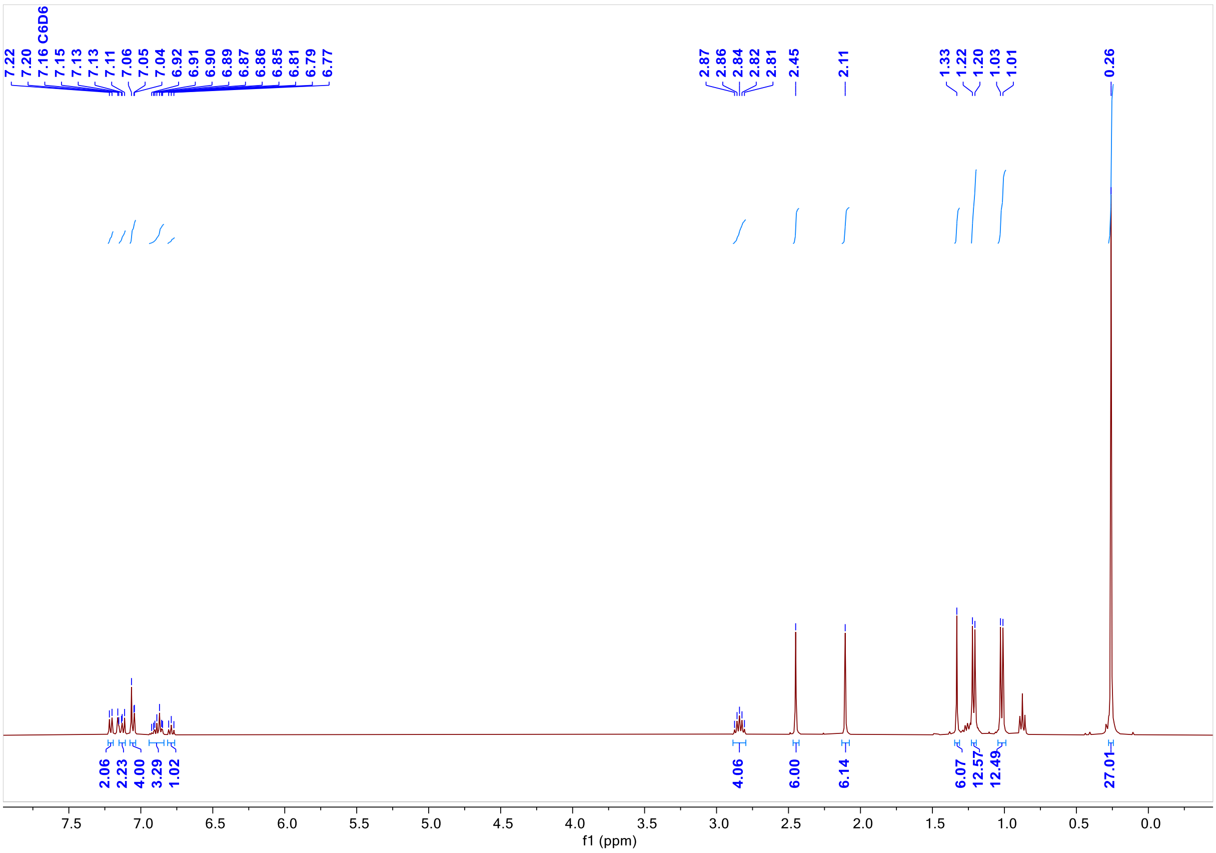

Figure S4.1. ^{1}H NMR spectrum of **4a** in C_6D_6 at 300K.

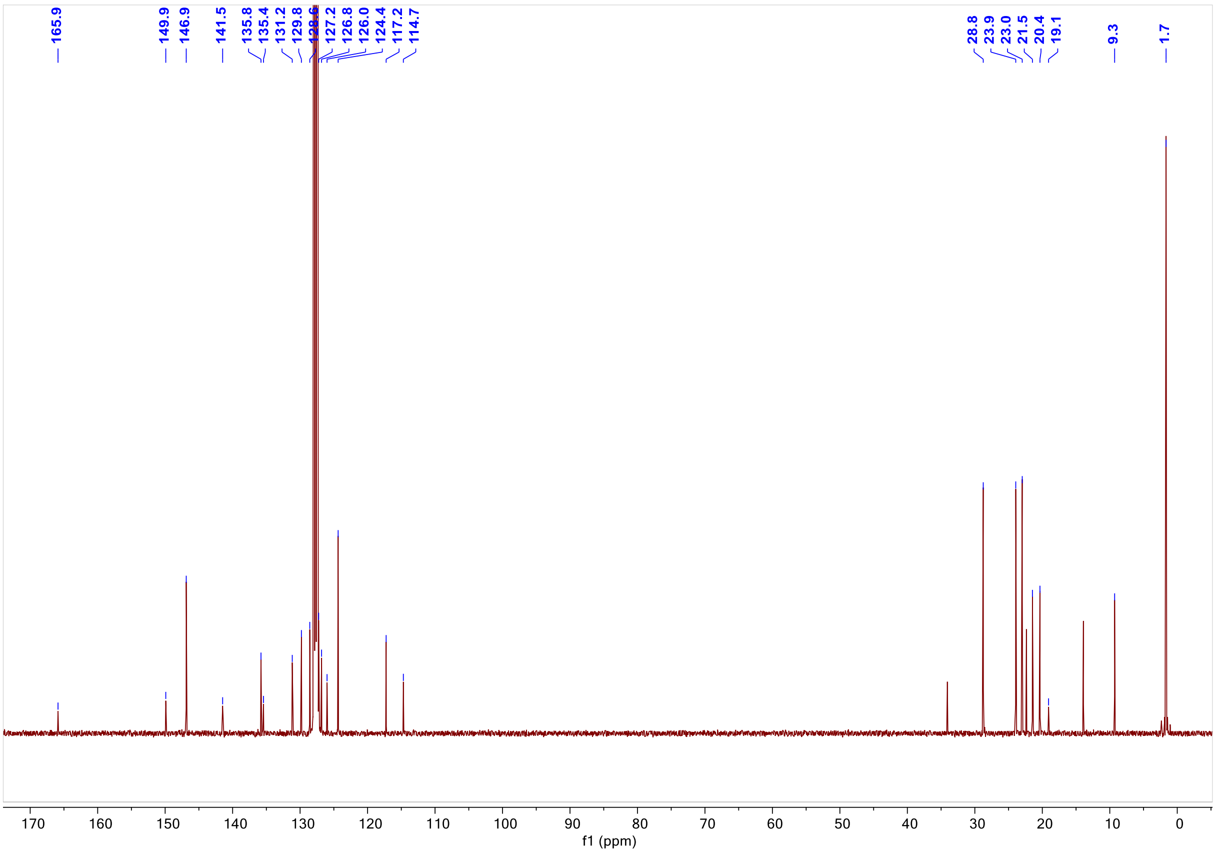

Figure S4.2. $^{13}C\{^{1}H\}$ NMR spectrum of **4a** in C_6D_6 at 300K.

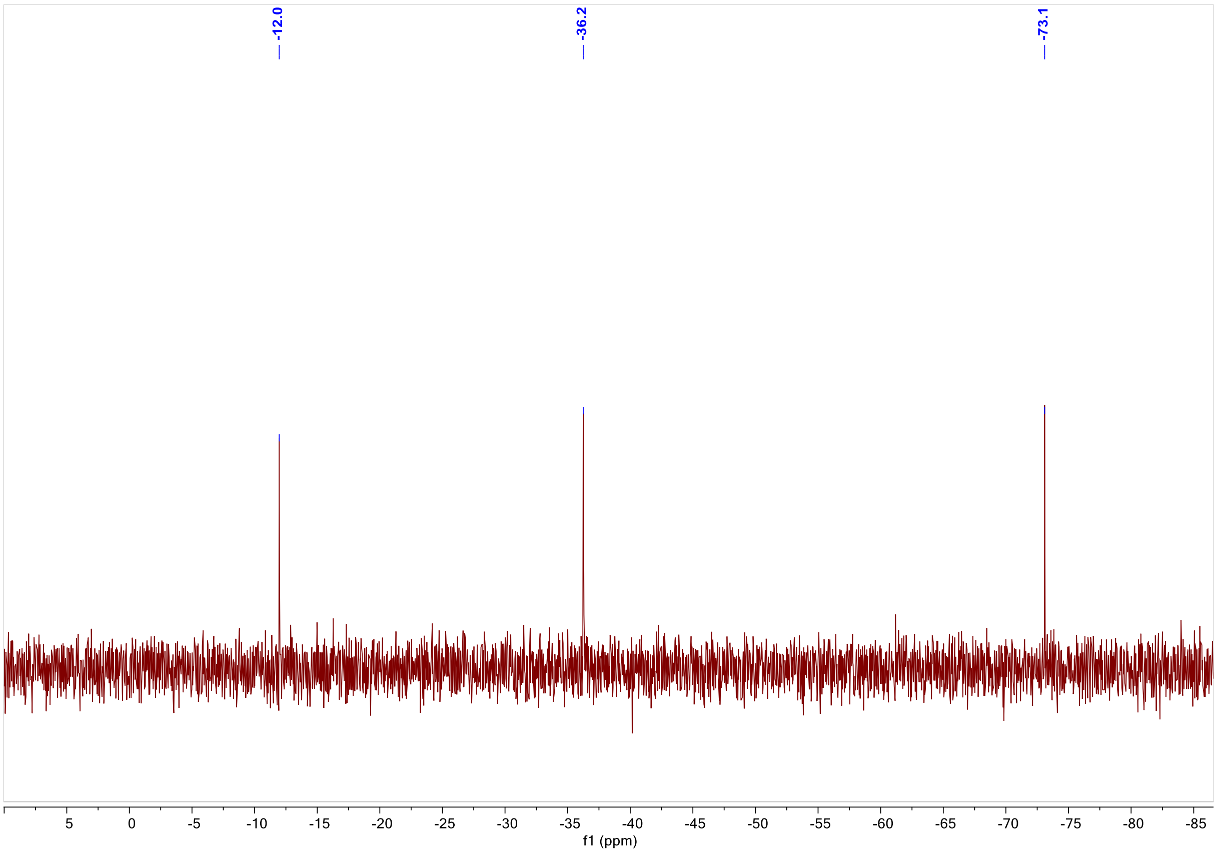

Figure S4.3. $^{29}Si\{^1H\}$ NMR spectrum of **4a** in C_6D_6 at 300K.

1.2.5 Synthesis of 4b

Silylene **1** (106.0 mg, 0.15 mmol) and **2b** (43.5 mg, 0.30 mmol) were combined in benzene (3 mL) at room temperature. The color of mixture changed to deep green then to orange rapidly from blue within 5 minutes. The orange solution was allowed to stir for 6 h at room temperature until the color turning yellow. All volatiles were removed *in vacuum*, the oily residue was recrystallized from pentane at –30 °C for 3 days. The yellow crystals were separated by removing top light orange solution, washed with cold pentane (3 × 1 mL) and dried *in vacuum* to yield **4b** (95.8 mg, 64%) as a yellow powder.

^{1}H NMR (400.1 MHz, C_6D_6): δ [ppm] 7.14-7.18 (m, 2H, Ar*H*, overlapping with C_6D_6), 7.07-7.09 (m, 4H, Ar*H*), 7.01(s, 2H, Ar*H*), 6.99(s, 2H, Ar*H*), 2.86 (sept, *J* = 6.8 Hz, 4H, C*H*(CH_3)$_2$), 2.46 (s, 6H, CCH_3), 2.40 (s, 3H, CCH_3), 2.11 (s, 3H, CCH_3), 1.34 (s, 6H, NCCH_3), 1.24 (d, *J* = 6.8 Hz, 12H, CH(CH_3)$_2$), 1.02 (d, *J* = 6.8 Hz, 12H, CH(CH_3)$_2$), 0.27 (s, 27H, C(CH_3)$_3$).

^{13}C{^{1}H} NMR (100.6 MHz, C_6D_6): δ [ppm] 165.8(C*C*N), 147.4 (N*C*N), 147.0 (Ar*C*),141.3 (Ar*C*), 135.5 (Ar*C*), 135.4 (Ar*C*), 132.8 (Ar*C*), 131.2 (Ar*C*), 129.8 (Ar*C*), 129.4 (Ar*C*), 128.0 (Ar*C*), 126.5 (Ar*C*), 124.3 (Ar*C*), 122.3 (Ar*C*), 117.2 (N*C*CH_3), 28.8 (*C*H(CH_3)$_2$), 23.9 (CH(CH_3)$_2$), 23.0 (CH(CH_3)$_2$), 21.4(ArCH_3), 20.9(ArCH_3), 20.6(ArCH_3), 20.3(ArCH_3), 18.9(*C*CN), 9.3 (NCCH_3), 1.7 (Si(CH_3)$_3$).

^{29}Si{^{1}H} NMR (79.5 MHz, C_6D_6): δ [ppm] –12.0 (*Si*Me$_3$), –36.7 (*Si*=N), –73.2 (*Si*TMS$_3$).

Elemental Analysis (%): Calcd: C 69.89, H 9.00, N 7.0; Found: C 69.23, H 9.02, N 7.05.

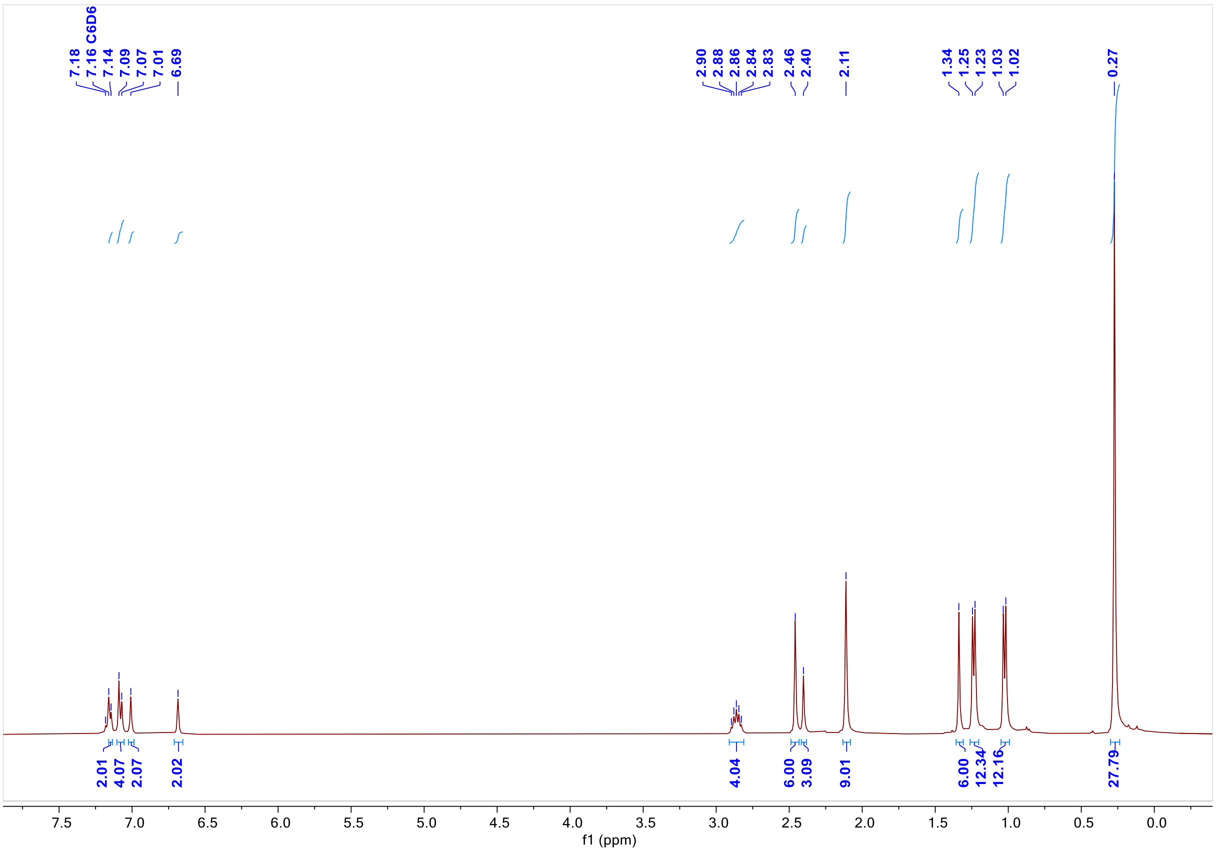

Figure S5.1. ^{1}H NMR spectrum of **4b** in C_6D_6 at 300K.

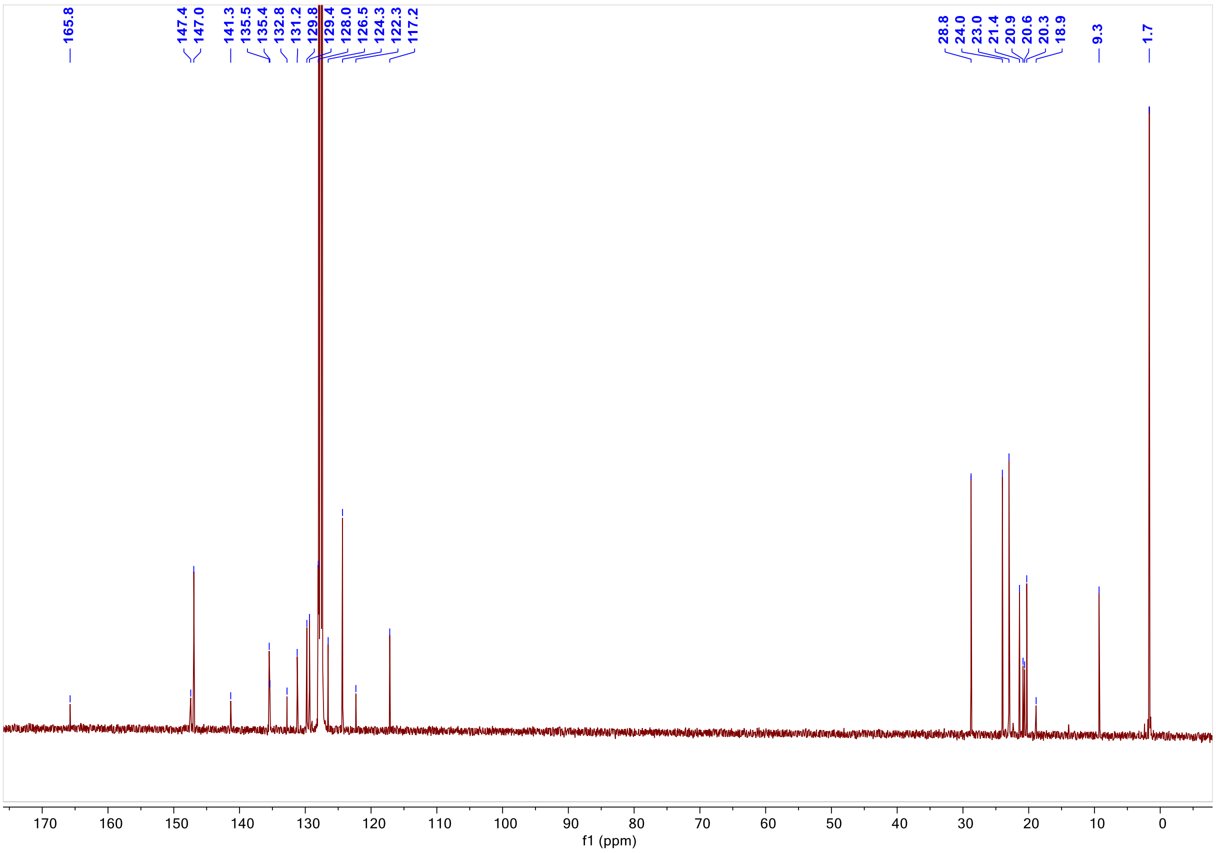

Figure S5.2. ^{13}C{^{1}H} NMR spectrum of **4b** in C_6D_6 at 300K.

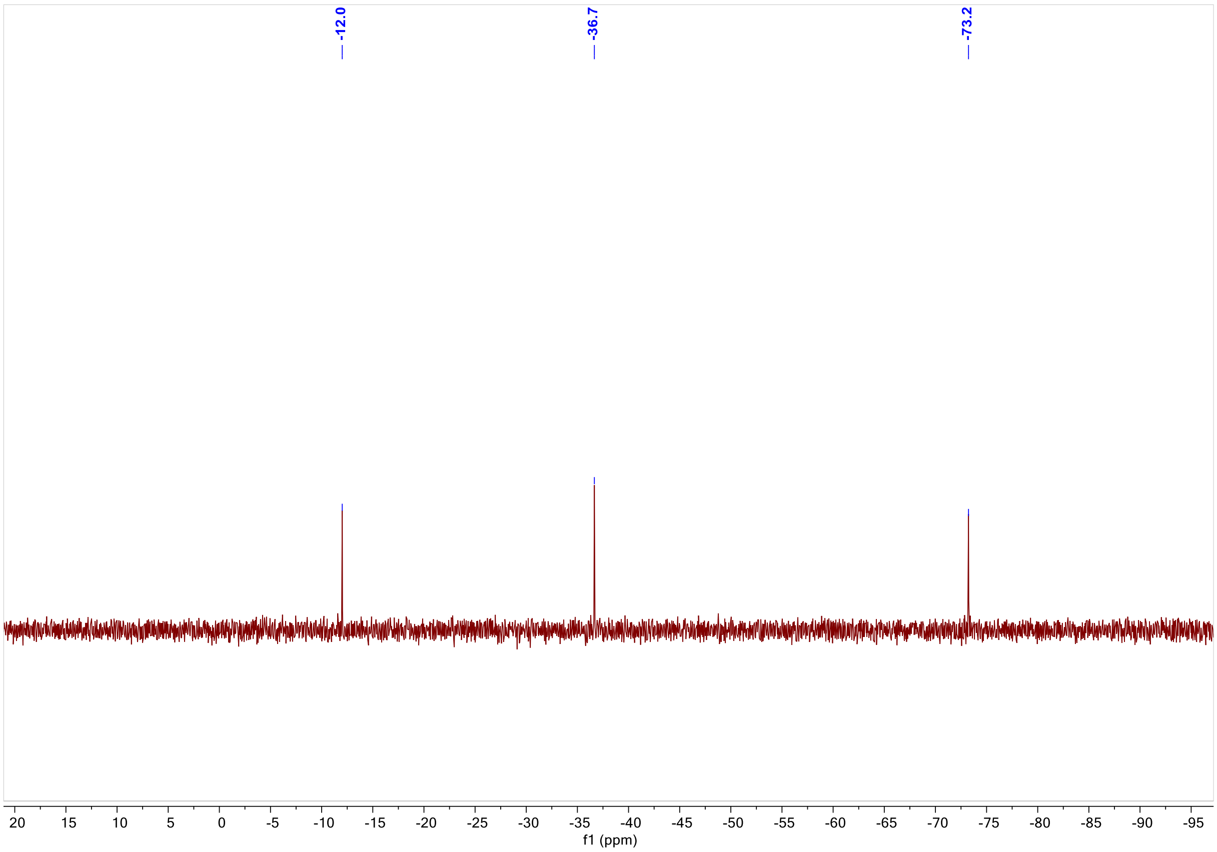

Figure S5.3. $^{29}Si\{^{1}H\}$ NMR spectrum of **4b** in C_6D_6 at 300K.

1.2.6 Synthesis of 5

4b (100 mg, 0.1 mmol) and **2b** (21.8 mg, 0.15 mmol) were combined in benzene (2 mL) at room temperature. The color of mixture changed to bright orange with some orange precipitate forming after stirring for 18 h at room temperature. All volatiles were removed *in vacuum*, the residue was washed with pentane (3 × 1 mL) and dried *in vacuum* to yield **5** (115.5 mg, 95%) as an orange powder. Crystals **5** suitable for single crystal X-ray diffraction analysis were obtained by slow evaporation of saturated benzene solution at room temperature for 2 days.

Note: Due to the pronounced intermolecular interactions involving hydrogen atoms in this system, the room temperature ^{1}H NMR and ^{13}C NMR spectra consistently exhibit extensive signal broadening, preventing the unambiguous assignment of individual proton or carbon resonances. The low-temperature (–78 °C) measurements failed to enhance spectral resolution.

^{29}Si{^{1}H} NMR (79.5 MHz, Tol-D$_8$): δ [ppm] –11.5 (*Si*Me$_3$), –12.6 (*central Si*), –87.5 (*Si*TMS$_3$).

Elemental Analysis (%): Calcd: C 71.52, H 8.83, N 7.36; Found: C 71.74, H 9.01, N 7.49.

LIFDI-MS: Calcd: 1140.6856; Found: 1140.6886.

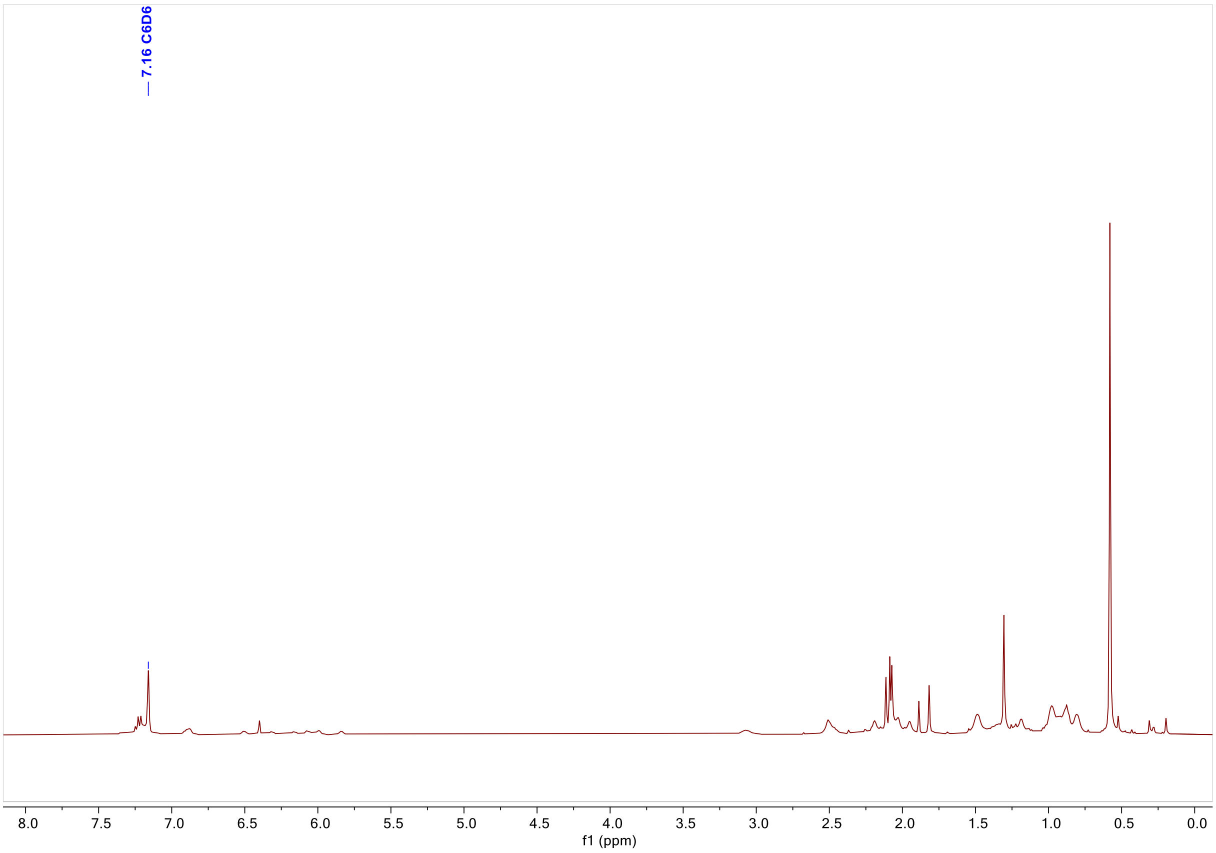

Figure S6.1. ^{1}H NMR spectrum of **5** in C_6D_6 at 300K.

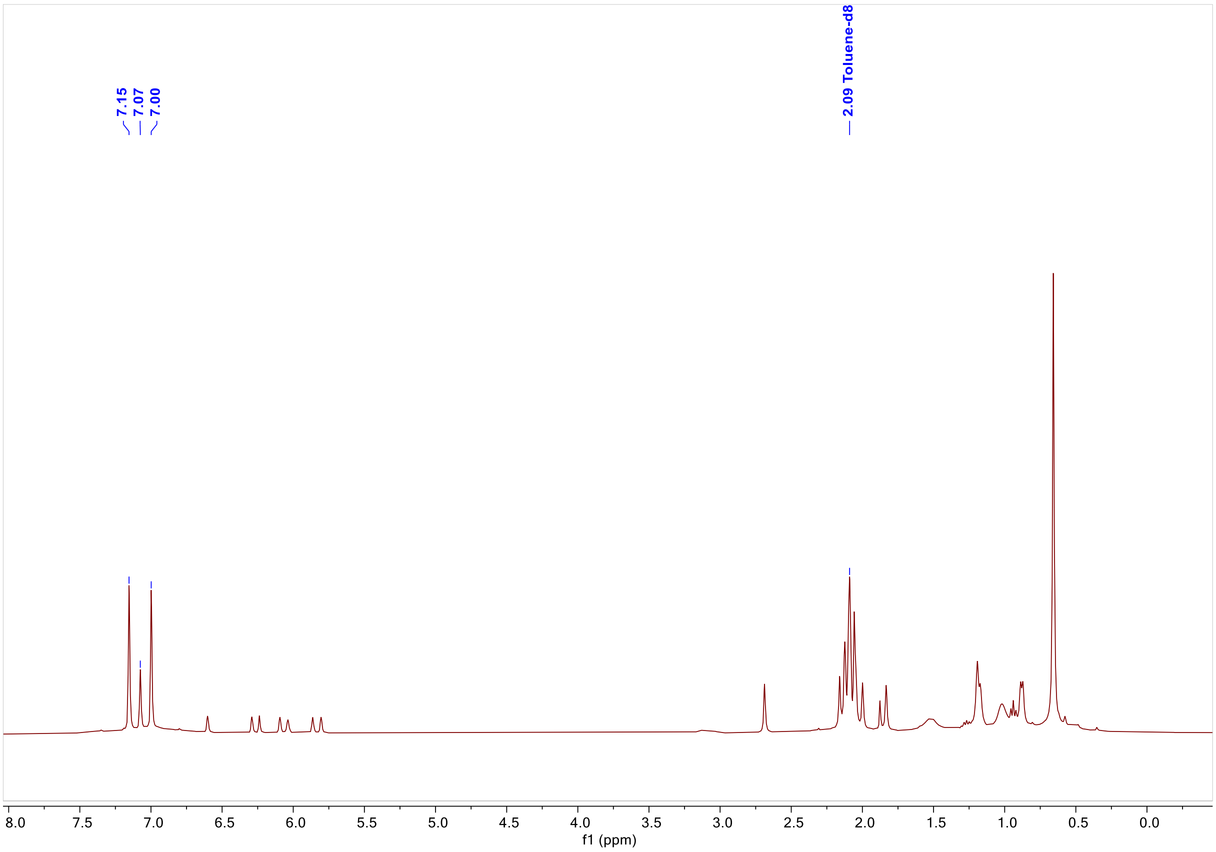

Figure S6.2. ^{1}H NMR spectrum of **5** in Tol-D_8 at 200K.

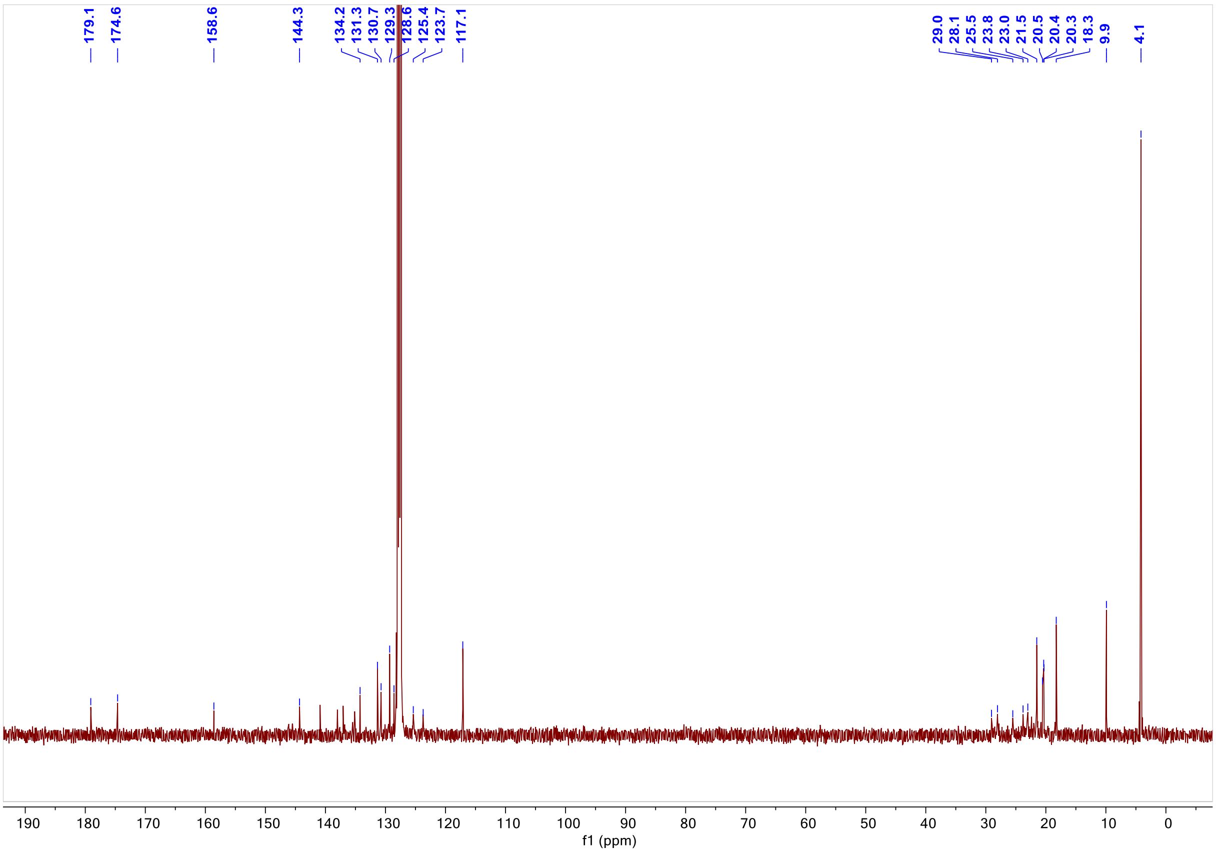

Figure S6.3. $^{13}C\{^{1}H\}$ NMR spectrum of **5** in C_6D_6 at 300K.

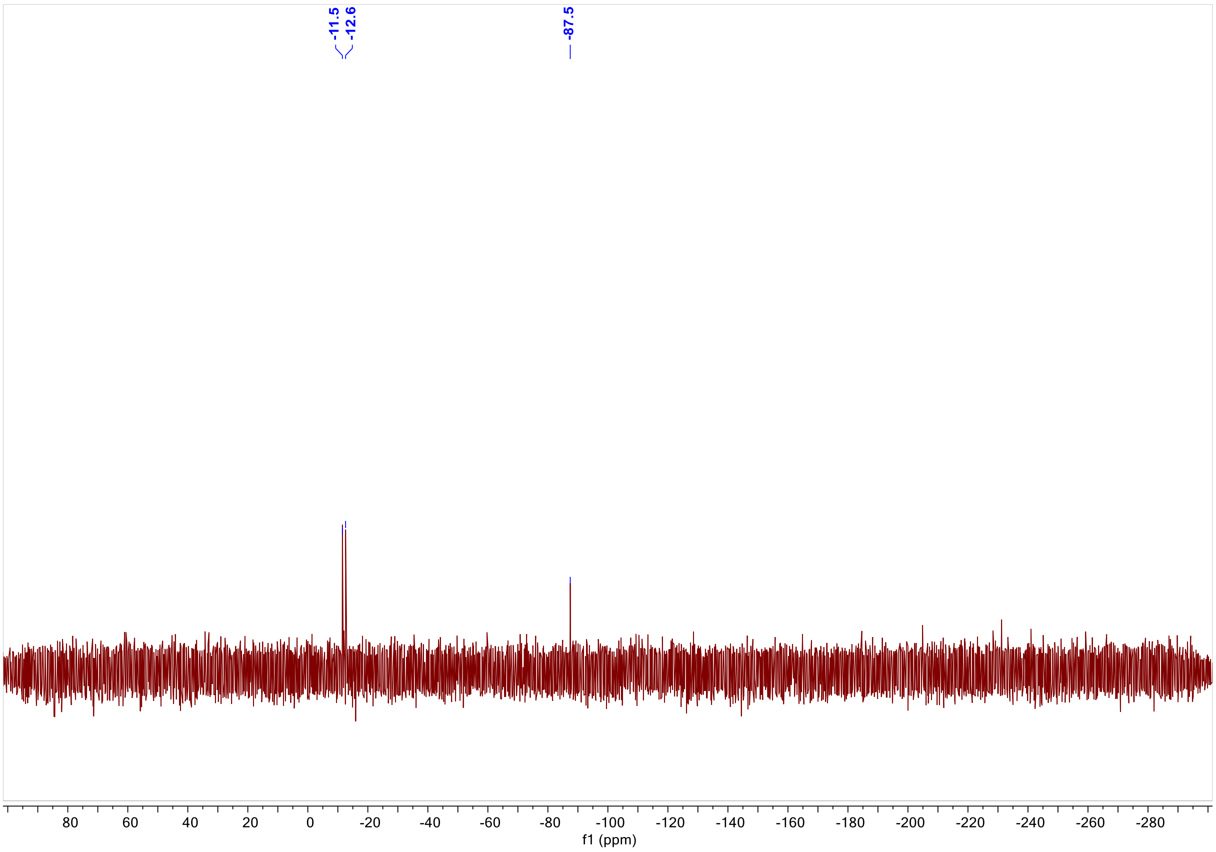

Figure S6.4. $^{29}Si\{^{1}H\}$ NMR spectrum of **5** in C_6D_6 at 300K.

1.2.7 Synthesis of 6a

4a (49 mg, 0.05 mmol) and **2a** (13.1 mg, 0.1 mmol) were combined in benzene (3 mL) at room temperature. The color of mixture turned to orange from yellow after stirring for 16 h. All volatiles were removed in vacuum, the residue was washed with pentane and dried in vacuum to yield **6a** (56 mg, 91%) as orange powder. Crystals **6a** suitable for single crystal X-ray diffraction analysis were obtained by storing a mixed toluene and pentane solution at –30 °C for 1 week.

Note: Due to the pronounced intermolecular interactions involving hydrogen atoms in this system, the room temperature ^{1}H NMR and ^{13}C NMR spectra consistently exhibit extensive signal broadening, preventing the unambiguous assignment of individual proton or carbon resonances. Surprisingly, the low-temperature (–50 °C) measurement for ^{1}H NMR could enhance spectral resolution.

^{1}H NMR (400.1 MHz, Tol-D$_8$): δ [ppm] 6.25-7.25 (m, 12H, Ar*H*), 6.21 (d, *J* = 7.2 Hz, 1H, CH_3CC*H*$CHCHCCH_3$), 6.09 (d, *J* = 6.0 Hz, 1H, $CH_3CCHCHC$*H*CCH_3), 5.88 (dd, *J* = 6.4 Hz, 1H, CH_3CCHC*H*$CHCCH_3$), 3.02 (sept, *J* = 6.8 Hz, 2H, C*H*$(CH_3)_2$), 2.64 (s, 3H, ArC*H*$_3$), 2.41 (sept, *J* = 6.4 Hz, 2H, C*H*$(CH_3)_2$), 2.08 (s, 3H, ArC*H*$_3$, overlapping with Tol-D$_8$), 2.02 (s, 3H, ArC*H*$_3$), 1.93 (s, 3H, ArC*H*$_3$), 1.39 (d, *J* = 4.2 Hz, 6H, CH(C*H*$_3$)$_2$), 1.21 (s, 6H, NCC*H*$_3$), 1.16 (s, 3H, $CH_3CCHCHCHCC$*H*$_3$), 0.94 (d, *J* = 6.4 Hz, 3H, CH(C*H*$_3$)$_2$), 0.87 (d, *J* = 6.4 Hz, 6H, CH(C*H*$_3$)$_2$), 0.69 (d, *J* = 4.2 Hz, 6H, CH(C*H*$_3$)$_2$), 0.60 (s, 27H, Si(C*H*$_3$)$_3$), 0.29 (d, *J* = 6.8 Hz, 3H, C*H*$_3CCHCHCHCCH_3$).

^{13}C{^{1}H} NMR (100.6 MHz, Tol-D$_8$): δ [ppm] 179.1 (*C*=NXyl), 175.4 (*C*=NXyl), 158.5 (*C*=N), 146.3 (N*C*N), 146.3 (Ar*C*), 141.4 (Ar*C*), 139.8 (Ar*C*), 135.3 (Ar*C*), 131.7 (Ar*C*), 129.7 (Ar*C*), 123.5, 121.1, 120.9, 117.6 (N*C*CH_3), 10.2 (NC*C*H_3), 4.4 (Si(*C*H_3)$_3$).

^{29}Si{^{1}H} NMR (79.5 MHz, Tol-D$_8$): δ [ppm] –11.5 (*Si*Me$_3$), –13.4 (*central Si*), –87.2 (*Si*TMS$_3$).

Elemental Analysis (%): Calcd: C 72.20, H 8.43, N 7.96; Found: C 72.02, H 8.52, N 7.43.

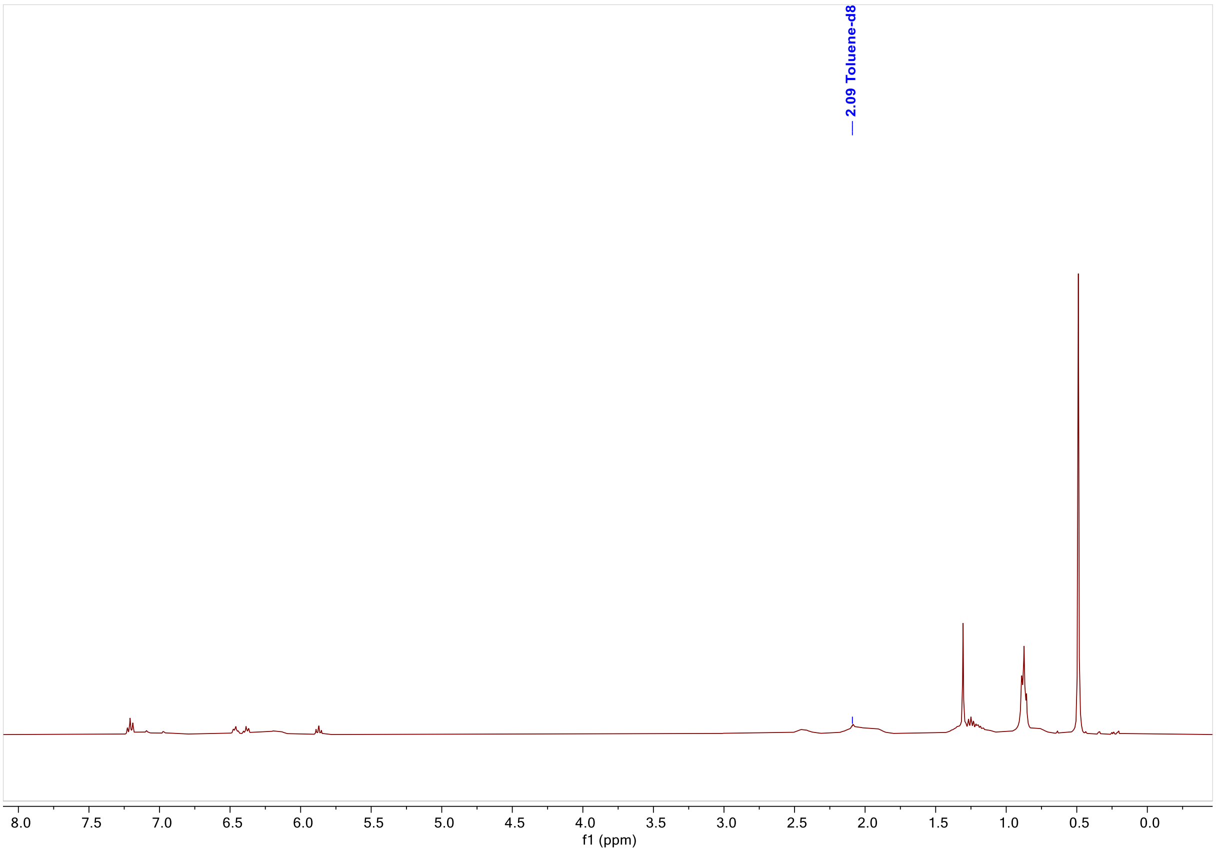

Figure S7.1. ^{1}H NMR spectrum of **6a** in Tol-D$_8$ at 300K.

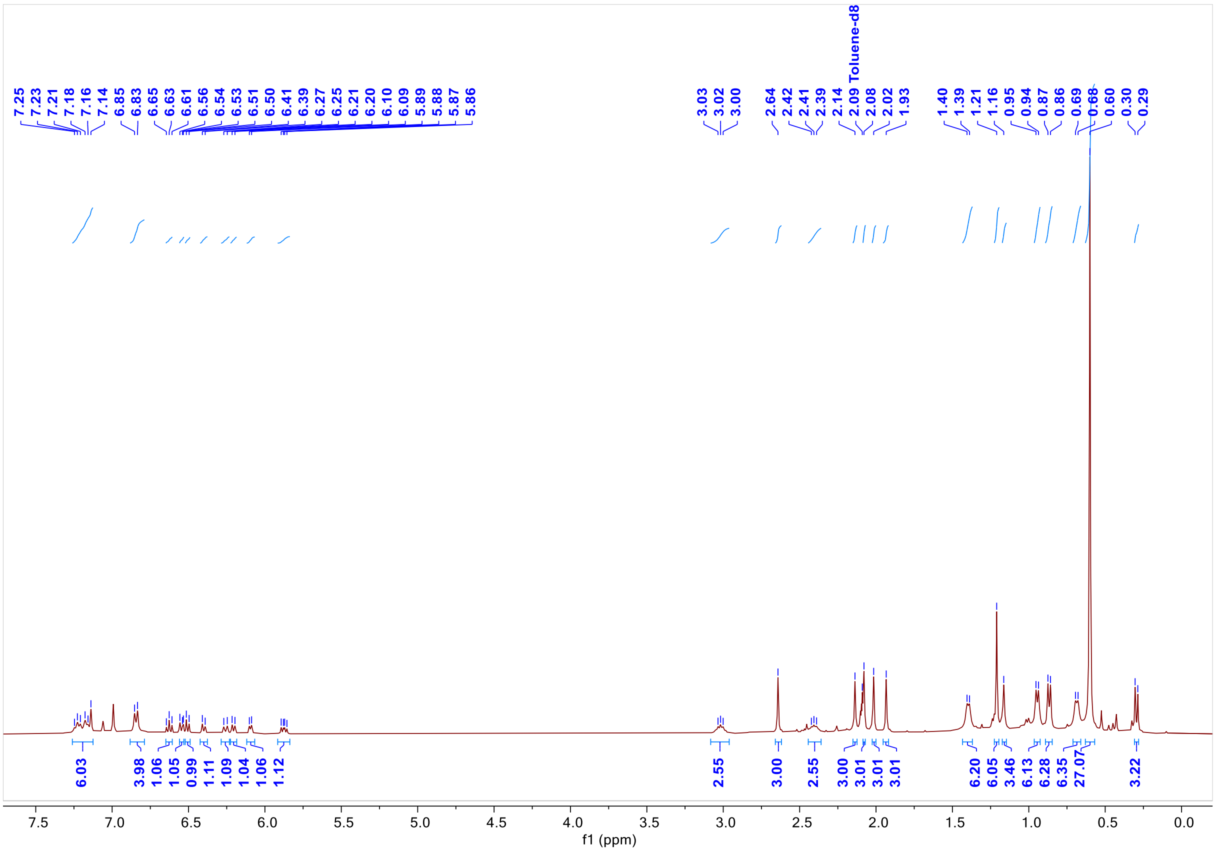

Figure S7.2. ^{1}H NMR spectrum of **6a** in Tol-D$_8$ at 233K.

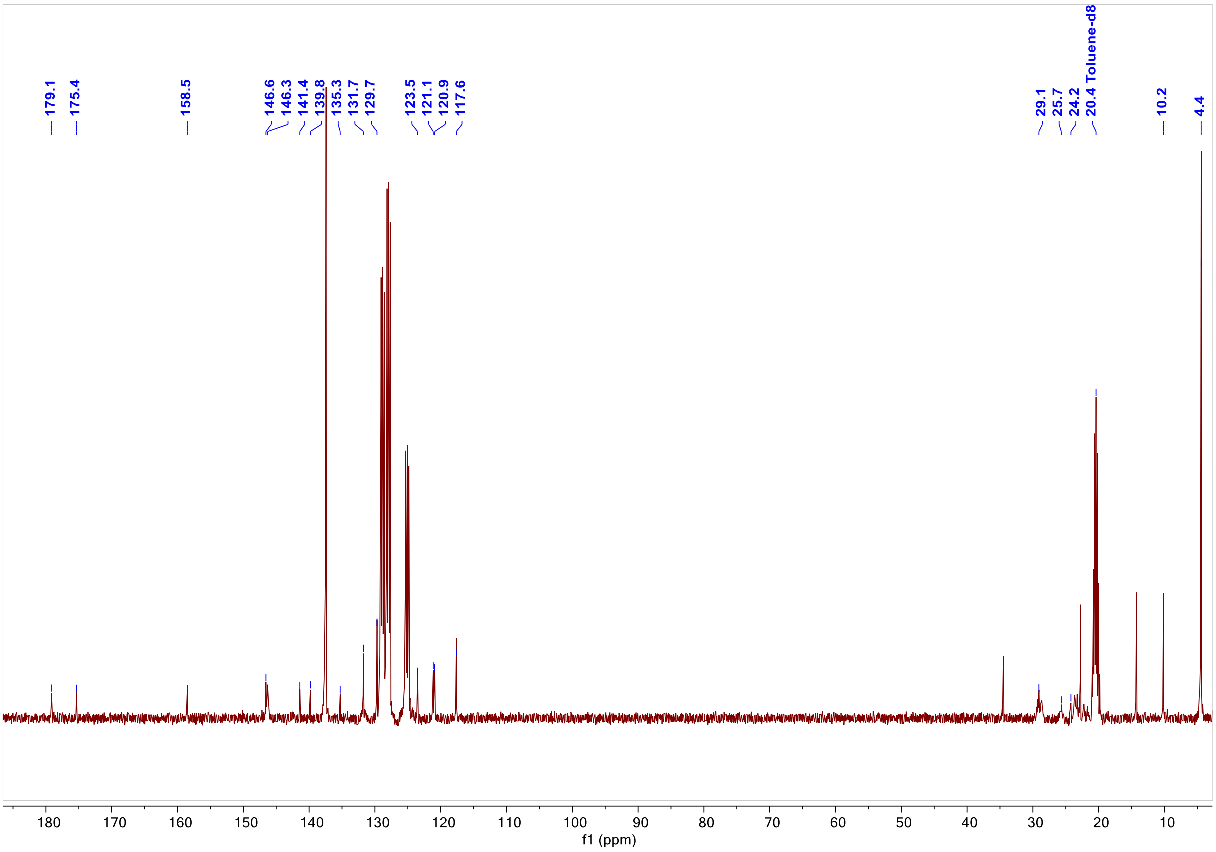

Figure S7.3. $^{13}C\{^{1}H\}$ NMR spectrum of **6a** in Tol-D_8 at 300K.

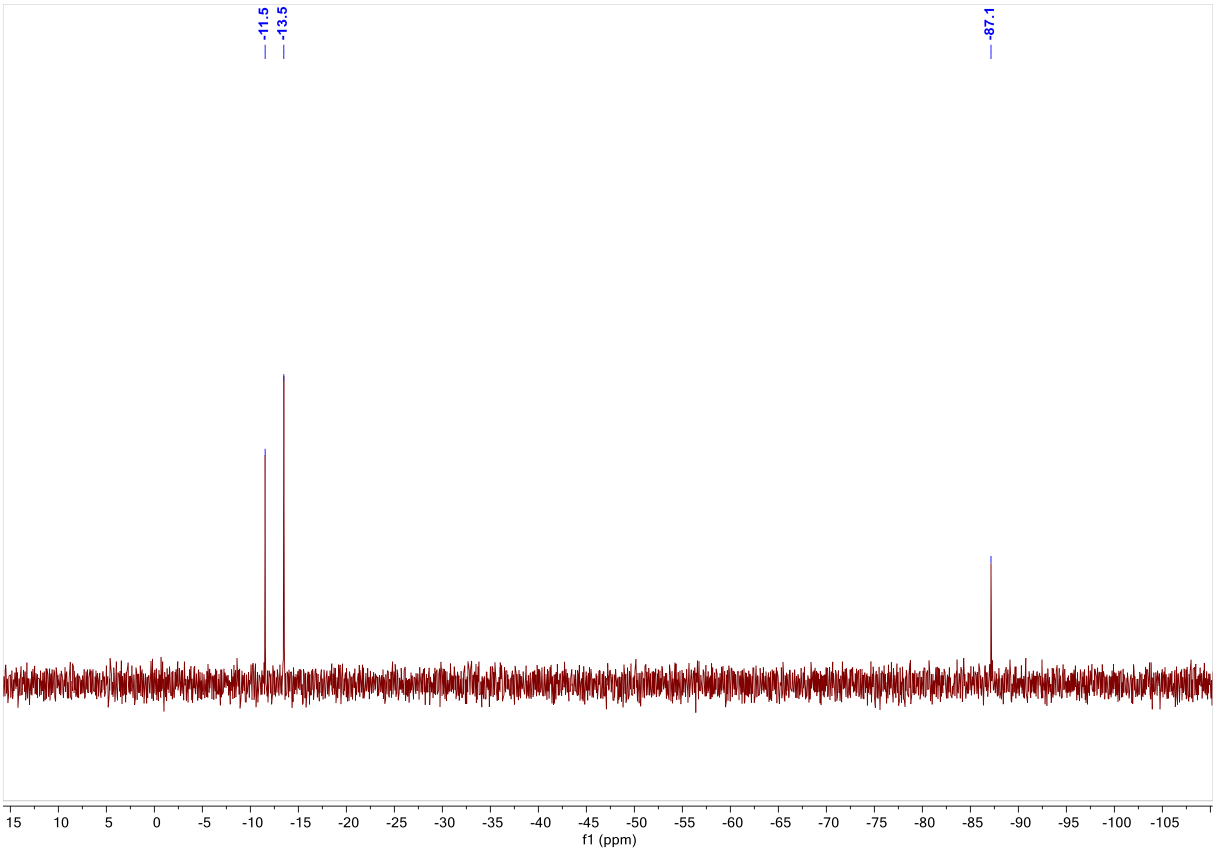

Figure S7.4. $^{29}Si\{^{1}H\}$ NMR spectrum of **6a** in Tol-D_8 at 300K.

1.2.8 Synthesis of 6b

A benzene solution of **5** (5 mg, 0.0044 mmol) and MesNC (6.4 mg, 0.044 mmol) was allowed to stir at room temperature. The reaction was monitored by ^{1}H NMR spectrum until the 33% conversion of **5** after 2 days. All volatiles were removed *in vacuum*, the residue was washed with pentane (3 × 2 mL). Due to the similar solubility and same color of **5** and **6b**, the purification of **6b** was failed. However, mixed crystals of **5** and **6b** suitable for single crystal X-ray diffraction analysis could be obtained by evaporation of a mixed benzene and pentane solution at room temperature for 24 h.

LIFDI-MS: Calcd (-$SiMe_3$): 1212.7274; Found: 14212.7175.

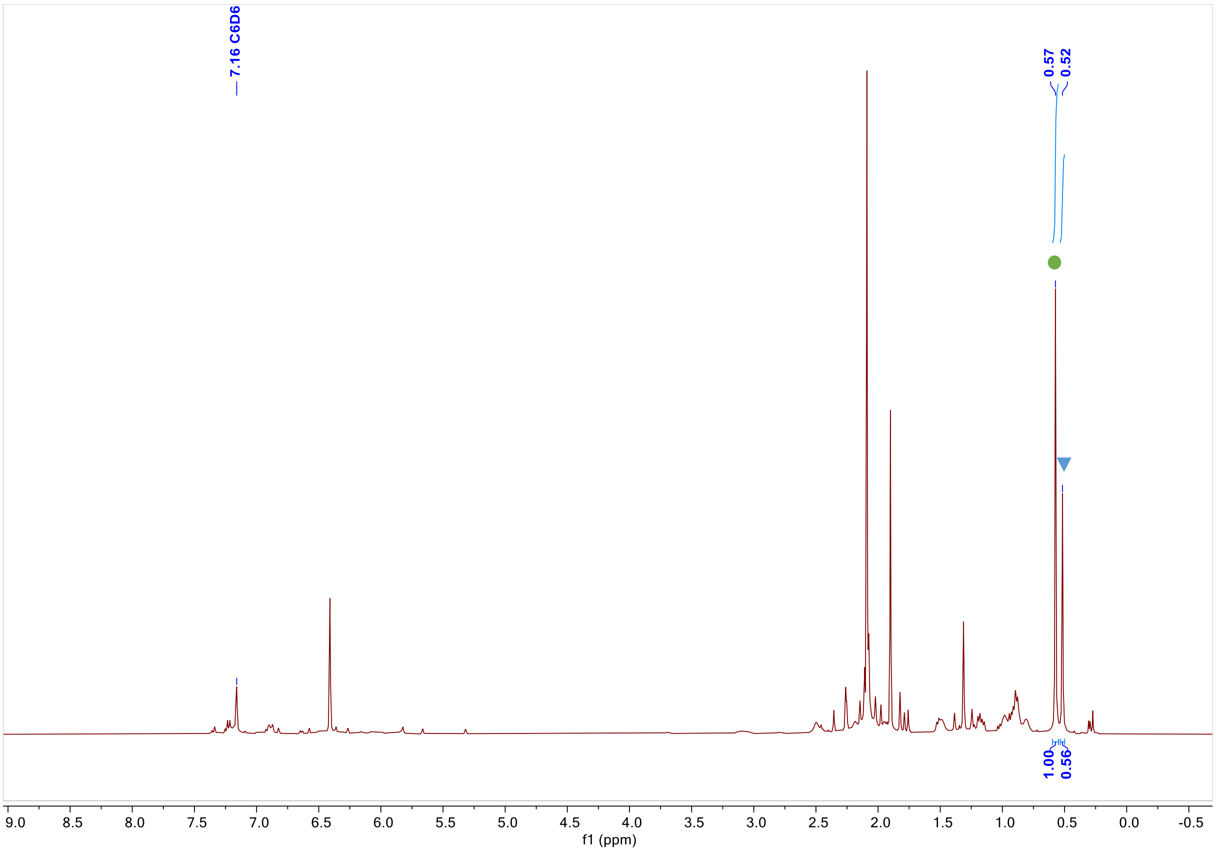

Figure S8.1. ^{1}H NMR spectrum of stoichiometric reaction of **5** with **2b** in C_6D_6 at 300K (●: **5**; ▼: **6b**).

1.2.9 Synthesis of 10a

A solution of **6a** (64.4 mg, 0.05 mmol) in benzene was degassed and subsequently exposed to CO (1 bar) under vigorous stirring at room temperature. The color of mixture turned to brown rapidly. All volatiles were removed from brown solution after stirring for 16 h at room temperature, the residue was recrystallized from a mixed toluene and pentane solution at –30 °C to yield **10a** (58.6 mg, 87%) as brown crystals.

Note: Due to the pronounced intermolecular interactions involving hydrogen atoms in this system, the room temperature ^{1}H NMR and ^{13}C NMR spectra consistently exhibit extensive signal broadening, preventing the unambiguous assignment of individual proton or carbon resonances. The low-temperature (–78 °C) measurements failed to enhance spectral resolution.

^{29}Si{^{1}H} NMR (79.5 MHz, C_6D_6): δ [ppm] 13.9 (*Si*Me_3), 10.3 (*Si*Me_3), –9.9 (*central Si*), –11.7 (*Si*TMS_2).

Elemental Analysis (%): Calcd: C 70.92, H 8.07, N 7.62; Found: C 71.13, H 7.99, N 7.64.

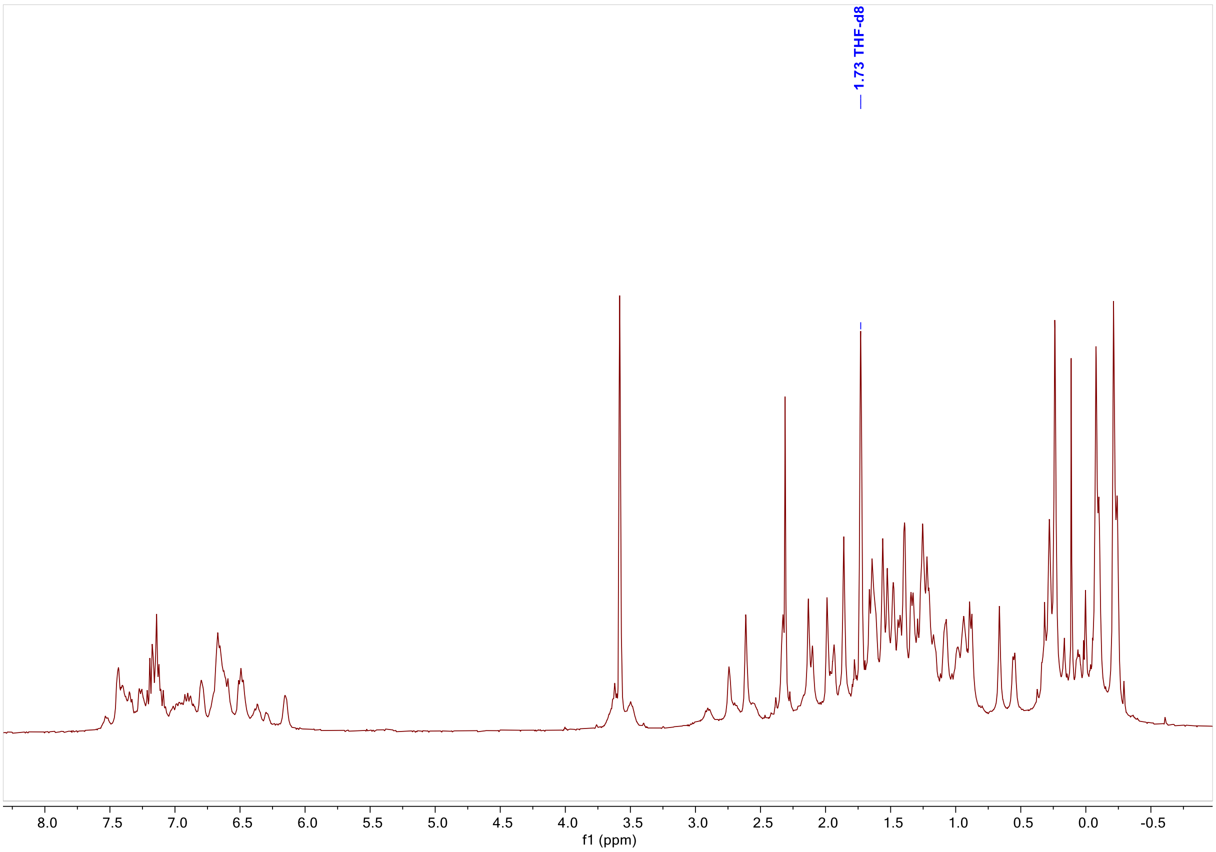

Figure S9.1. ^{1}H NMR spectrum of **10a** in THF-D_8 at 300K.

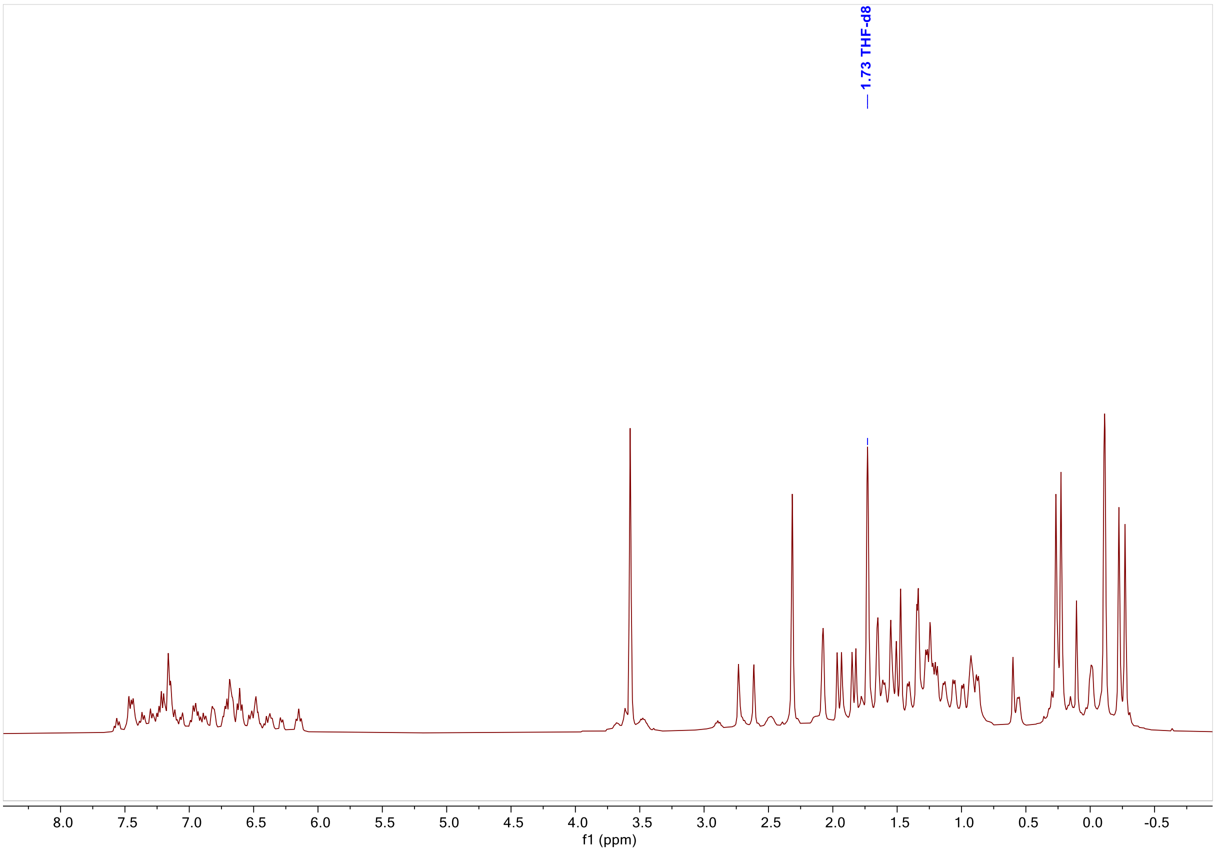

Figure S9.2. ^{1}H NMR spectrum of **10a** in THF-D_8 at 200K.

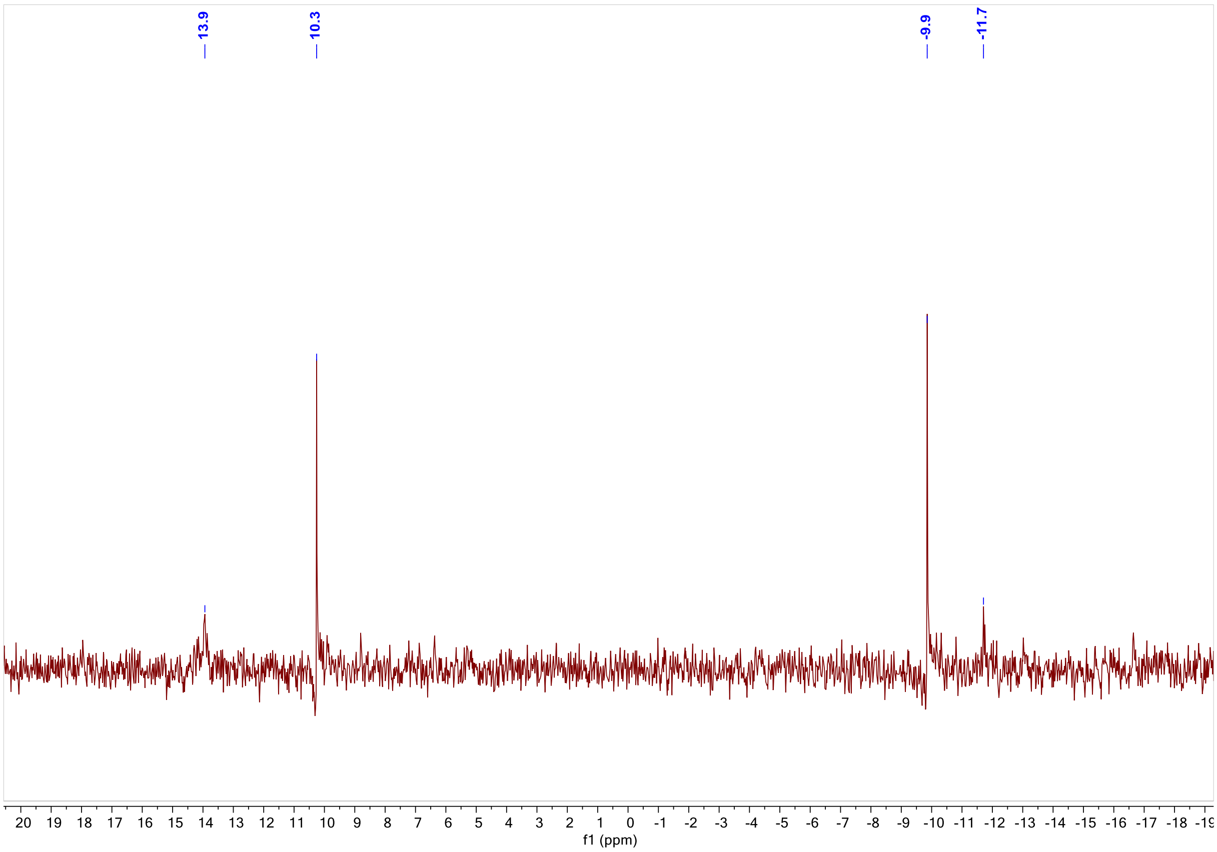

Figure S9.3. $^{29}Si\{^{1}H\}$ NMR spectrum of **10a** in THF-D$_8$ at 300K.

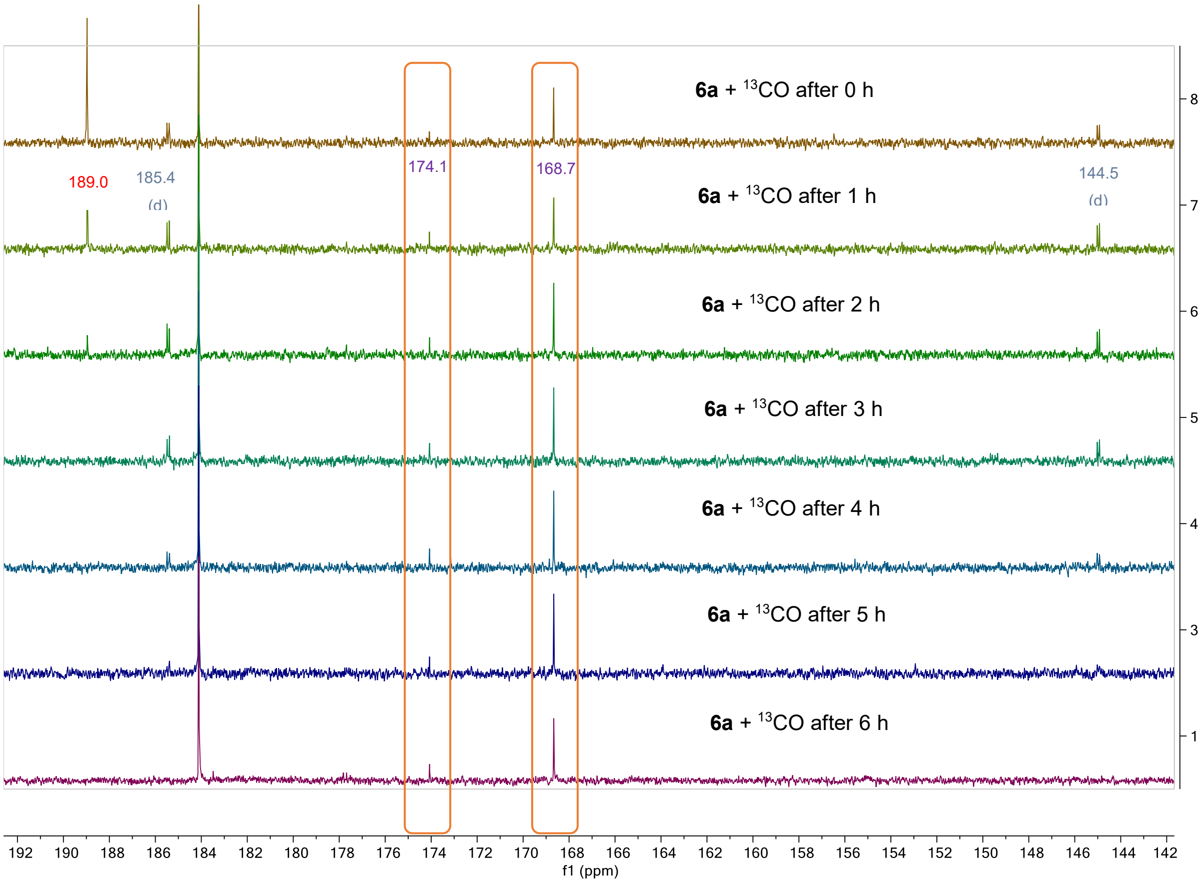

Figure S9.4. Stacked ^{13}C NMR spectrum (C_6D_6) for the reaction of **6a** with ^{13}CO gas (1 bar).

To observe the potential intermediate during the heterocoupling of CO and XylNC (**2a**), we performed the reaction of **6a** with ^{13}CO at 0 °C and measured the ^{13}C NMR every one hour, as shown the stacked ^{13}C NMR in Figure R1. In the first spectrum (**6a** + ^{13}CO after 0 h), five signals could be observed. We suggest that signals observed at 174.1 and 168.7 ppm assigning to ^{13}CO moieties in **10a**, signal observed at 189.0 ppm assigning to **INT14** and signals observed at 185.4 and 144.5 ppm (d, $J_{C\text{–}C}$ = 10.0 Hz) assigning to **INT21**. The **INT14** was totally consumed within 3 hours and this reaction was completed within 6 hours. The much quicker consumption of **INT14** than **INT21** can be explained by the higher kinetic barriers for **INT21** → **10a** transformation (24.5 kcal mol^{-1}) than **INT14** → **INT15** transformation (22.6 kcal mol^{-1}).

1.2.10 Synthesis of 10b

A benzene suspension of **5** (57.1 mg, 0.05 mmol) and **2b** (7.3 mg, 0.05 mmol) was degassed and subsequently exposed to CO (1 bar) under vigorous stirring at room temperature. After stirring at room temperature for 5 h, the color of suspension turned to deep brown with the formation of brown precipitates. All volatiles were removed from brown suspension, the residue was washed with pentane (3 × 2 mL) and dried *in vacuum* to yield **10b** (55.7 mg, 83%) as a brown powder. Crystals of **10b** suitable for single crystal X-ray diffraction analysis were obtained by storing a mixed toluene and pentane solution at –30 °C for 1 week.

Note: Due to the pronounced intermolecular interactions involving hydrogen atoms in this system, the room temperature ^{1}H NMR and ^{13}C NMR spectra consistently exhibit extensive signal broadening, preventing the unambiguous assignment of individual proton or carbon resonances. The ^{29}Si NMR signals were not detected due to the poor solubility in organic solvents.

Elemental Analysis (%): Calcd: C 71.53, H 8.33, N 7.30; Found: C 71.67, H 8.67, N 7.05.

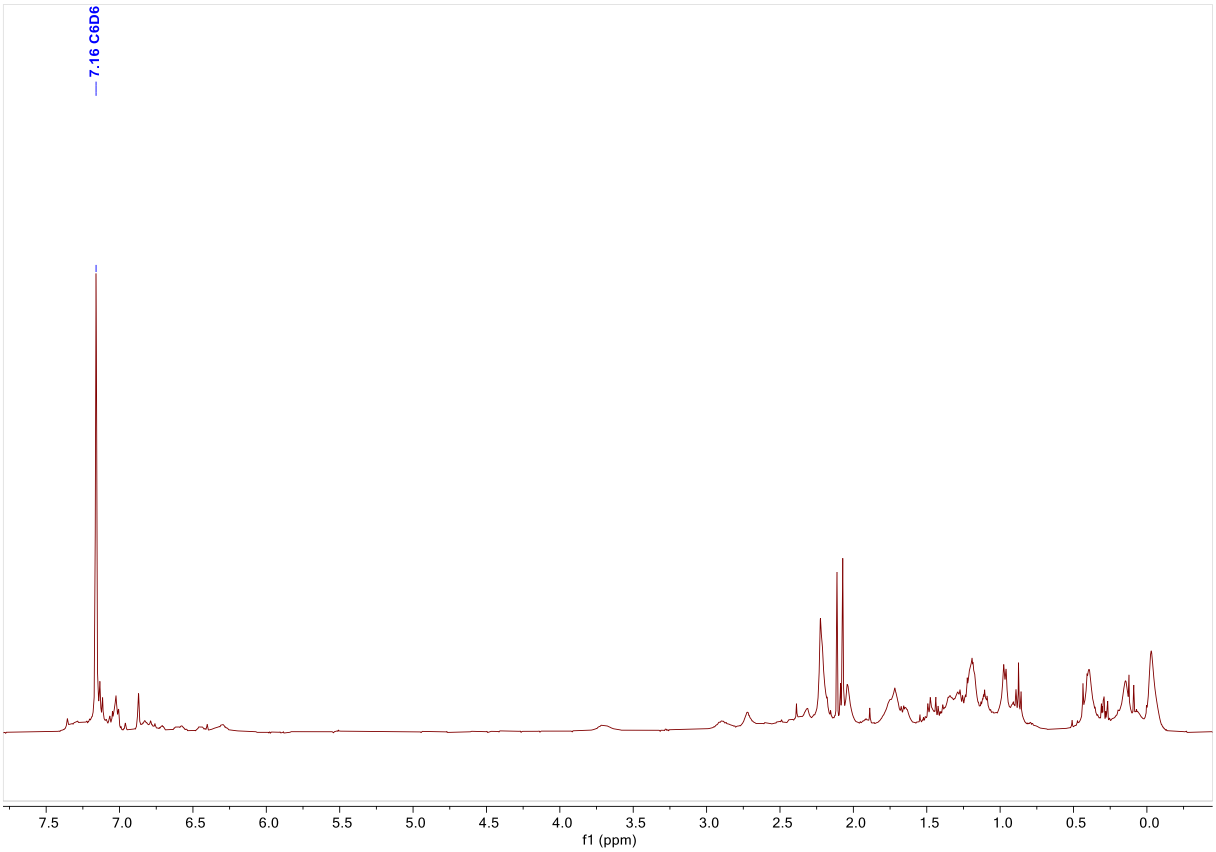

Figure S10.1. ^{1}H NMR spectrum of **10b** in C_6D_6 at 300K.

2. Single Crystal X-Ray Structure Determination

Single crystal diffraction data were recorded on a Bruker Photon D8 Venture DUO IMS system equipped with a Helios optic monochromator and a Mo IMS microsource (λ = 0.71073 Å) The data collection was performed, using the APEX IV software package[S3] on single crystals coated with Fomblin®Y as perfluorinated ether. The single crystals were picked on a micro sampler, transferred to the diffractometer, and measured frozen under a stream of cold nitrogen (100 K). A matrix scan was used to determine the initial lattice parameters. Reflections were merged and corrected for Lorenz and polarization effects, scan speed, and background using SAINT.[S4] Absorption corrections, including odd and even ordered spherical harmonics were performed using SADABS.[S4] Space group assignments were based upon systematic absences, E statistics, and successful refinement of the structures. Structures were solved by direct methods with the aid of successive difference Fourier maps and were refined against all data using the APEX IV software in conjunction with SHELXL-2014[S5] and SHELXLE.[S6] H atoms were placed in calculated positions and refined using a riding model, with methylene and aromatic C–H distances of 0.99 and 0.95 Å, respectively, and Uiso(H) = 1.2·Ueq(C). Non-hydrogen atoms were refined with anisotropic displacement parameters. Full-matrix least-squares refinements were carried out by minimizing Σw(Fo2-Fc2)2 with the SHELXL weighting scheme.[S7] Neutral atom scattering factors for all atoms and anomalous dispersion corrections for the non-hydrogen atoms were taken from International Tables for Crystallography.[S8] The images of the crystal structures were generated by Mercury.[S9] The CCDC numbers 2498260 to 2498267 contain the supplementary crystallographic data for the structures of **1** to **10**. These data can be obtained free of charge from the Cambridge Crystallographic Data Centre via https://www.ccdc.cam.ac.uk/structures/.

Table S1. Crystallographic details

	**compound_1**	**compound_3a**	**compound_4a**
**CCDC-Number**	2498260	2498261	2498262
Chemical formula	$C_{38}H_{67}N_3Si_5$	$[C_{56}H_{85}N_5Si_5]\cdot C_5H_{12}$	$C_{56}H_{85}N_5Si_5$
M_r	706.39	1040.88	968.73
Crystal system, space group	Monoclinic, $P2_1/n$	Monoclinic, $C2/c$	Triclinic, $P\bar{1}$
Temperature (K)	100	100	100
a (Å), α(°)<br>*b (Å)*, β(°)<br>*c (Å)*, γ(°)	10.8364(5), 90<br>18.4047(9), 100.970(2)<br>22.8426(11), 90	45.931(3), 90<br>12.2170(8), 98.128(2)<br>22.4576(13), 90	12.5246(10), 87.345(3)<br>13.6422(12), 84.846(3)<br>18.6662(17), 84.250(3)
V (Å^3)	4472.5(4)	12475.2(14)	3158.2(5)
Z	4	8	2
F(000)	1544	4544	1052
D_x (g/cm^3)	1.049	1.108	1.019
Radiation type	Mo $K\alpha$	Mo $K\alpha$	Mo $K\alpha$
μ (mm^{-1})	0.187	0.155	0.149
θ range (°) for cell meas.	2.25–25.33	2.47–25.67	2.61–25.70
Crystal size (mm)	0.297 × 0.236 × 0.198	0.215 × 0.189 × 0.121	0.247 × 0.221 × 0.165
Diffractometer	Bruker Photon CMOS	Bruker Photon CMOS	Bruker Photon CMOS
Radiation source	TXS rotating anode	TXS rotating anode	TXS rotating anode
Monochromator	Helios optic	Helios optic	Helios optic
Absorption correction	Multi-scan	Multi-scan	Multi-scan
T_{min}, T_{max}	0.709, 0.745	0.694, 0.745	0.717, 0.745
θ_{max} (°)	25.349	25.753	25.682
Range of *h*, *k*, *l*	h = -13→13, k = -22→22, l = -27→27	h = -56→55, k = -14→14, l = -27→27	h = -15→15, k = -16→16, l = -22→22
Refinement method	Full-matrix least-squares on F^2	Full-matrix least-squares on F^2	Full-matrix least-squares on F^2
Data/restraints/ parameters	8181/0/434	11871/0/665	11998/0/618
Goodness-of-fit on F^2	1.094	1.198	1.070
Final *R* indices (I>2σ(I))	R_1 = 0.043, wR_2 = 0.1142	R_1 = 0.0867, wR_2 = 0.2070	R_1 = 0.0470, wR_2 = 0.1344
$\Delta\rho_{max}$, $\Delta\rho_{min}$ (e Å^{-3})	0.972, -0.428	1.237, -0.704	0.929, -0.628

	compound_5	compound_6a	compound_6b
**CCDC-Number**	2498263	2498264	2498265
Chemical formula	$C_{68}H_{100}N_6Si_5$	$C_{74}H_{103}N_7Si_5$	$[C_{78}H_{111}N_7Si_5]\cdot C_3H_3$
M_r	1141.98	1231.08	1326.24
Crystal system, space group	Monoclinic, $P2_1/n$	Triclinic, $P\bar{1}$	Monoclinic, $P2_1$/c
Temperature (K)	100	100	100
a (Å), α(°)<br>*b (Å)*, β(°)<br>*c (Å)*, γ(°)	13.2571(8), 90<br>22.2525(10), 92.424(2)<br>23.0561(13), 90	12.2847(6), 100.484(2)<br>15.4680(8), 96.298(2)<br>21.7838(10), 94.326(2)	23.4128(7), 90<br>16.3814(5), 115.5730(10)<br>22.5530(6), 90
V (Å^3)	6795.5(6)	4026.4(3)	7802.5(4)
Z	4	2	4
F(000)	2480	1332	2876
D_x (g/cm^3)	1.116	1.015	1.129
Radiation type	Mo $K\alpha$	Mo $K\alpha$	Mo $K\alpha$
μ (mm^{-1})	0.148	0.129	0.138
θ range (°) for cell meas.	2.55–27.47	2.25–25.72	2.49–24.56
Crystal size (mm)	0.256 × 0.227 × 0.158	0.05 × 0.04 × 0.04	0.139× 0.078 × 0.015
Diffractometer	Bruker Photon CMOS	Bruker Photon CMOS	Bruker Photon CMOS
Radiation source	TXS rotating anode	TXS rotating anode	TXS rotating anode
Monochromator	Helios optic	Helios optic	Helios optic
Absorption correction	Multi-scan	Multi-scan	Multi-scan
T_{min}, T_{max}	0.690, 0.745	0.721, 0.745	0.683, 0.745
θ_{max} (°)	27.506	25.350	25.661
Range of *h*, *k*, *l*	h = -17→17, k = -28→28, l = -29→29	h = -14→14, k = -18→18, l = -26→26	h = -28→28, k = -19→19, l = -27→26
Refinement method	Full-matrix least-squares on F^2	Full-matrix least-squares on F^2	Full-matrix least-squares on F^2
Data/restraints/ parameters	15590/0/740	14753/0/802	14728/489/1099
Goodness-of-fit on F^2	1.052	1.036	1.036
Final *R* indices (I>2σ(I))	R_1 = 0.0416, wR_2 = 0.1206	R_1 = 0.0380, wR_2 = 0.0898	R_1 = 0.0644, wR_2 = 0.1709
$\Delta\rho_{max}$, $\Delta\rho_{min}$ (e Å^{-3})	0.360, -0. 276	0. 312, -0. 246	0.816, -0.426

	compound_10a	compound_10b
**CCDC-Number**	2498266	2498267
Chemical formula	$C_{76}H_{103}N_7Si_5O_2$	$C_{80}H_{111}N_7Si_5O_2$
M_r	1287.10	1343.20
Crystal system, space group	Monoclinic, $P2_1/c$	Orthorhombic, *Pbca*
Temperature (K)	100	100
a (Å), α(°)<br>*b (Å)*, β(°)<br>*c (Å)*, γ(°)	13.089(5), 90<br>22.136(8), 92.605(11)<br>54.085(17), 90	26.3004(11), 90<br>22.6678(8), 90<br>26.6966(10), 90
V ($Å^3$)	15654(9)	15915.8(11)
Z	8	8
F(000)	5552	5808
D_x (g/cm^3)	1.092	1.121
Radiation type	Mo $K\alpha$	Mo $K\alpha$
μ (mm^{-1})	0.137	0.138
θ range (°) for cell meas.	2.31–24.74	2.35–25.46
Crystal size (mm)	0.05 × 0.01 × 0.01	0.329 × 0.136 × 0.131
Diffractometer	Bruker Photon CMOS	Bruker Photon CMOS
Radiation source	TXS rotating anode	TXS rotating anode
Monochromator	Helios optic	Helios optic
Absorption correction	Multi-scan	Multi-scan
T_{min}, T_{max}	0.686, 0.745	0.662, 0.745
θ_{max} (°)	26.029	25.692
Range of *h*, *k*, *l*	h = -16→15, k = -26→27, l = -65→66	h = -27→32, k = -27→26, l = -32→23
Refinement method	Full-matrix least-squares on F^2	Full-matrix least-squares on F^2
Data/restraints/ parameters	29535/19/1707	15091/0/878
Goodness-of-fit on F^2	1.069	1.015
Final *R* indices (I>2σ(I))	R_1 = 0.1051, wR_2 = 0.2217	R_1 = 0.0546, wR_2 = 0.1667
$\Delta\rho_{max}$, $\Delta\rho_{min}$ (e Å^{-3})	0. 914, -0.528	0.390, -0.295

3. Computational Details

Calculations were carried out using ORCA software.[S10]

Geometry optimizations were carried out using the r^{2}SCAN-3c composite method, [S11] utilizing the regularized and restored SCAN functional,[S12,S13] geometrical counterpoise correction gCP,[S14] the atom-pairwise dispersion correction based on tight binding partial charges (D4),[15-17] the def2-mTZVPP basis set and def2-mTZVPP/J auxiliary basis set.[S11]

The optimized geometries were verified as minima or transition states by analytical frequency calculations. The transition states were additionally verified by IRC calculations. Single point calculations of the optimized geometries were carried out at the r^{2}SCAN-3c level in benzene using the SMD solvation module[S18] to obtain electrostatic contribution and the cavity term to account for the solvent effects. To get more accurate electronic energies for the mechanistic investigations, single point calculations of the r^{2}SCAN-3c optimized geometries were carried using the PW6B95 [S19] functional, with D4 dispersion correction, the def2-QZVPP[S20] basis set and def2/J[S21] auxiliary basis set. The level at which the free energies were obtained is denoted as (SMD=Benzene)PW6B95-D4/def2-QZVPP//r^{2}SCAN-3c. Singlet diradical species were treated using broken-symmetry DFT. Spin symmetry breaking was enforced using the ORCA feature “BrokenSym 1,1” allowing localization of one α and one β electron on different centers. The summary of the thermochemistry results is presented in Table S2.

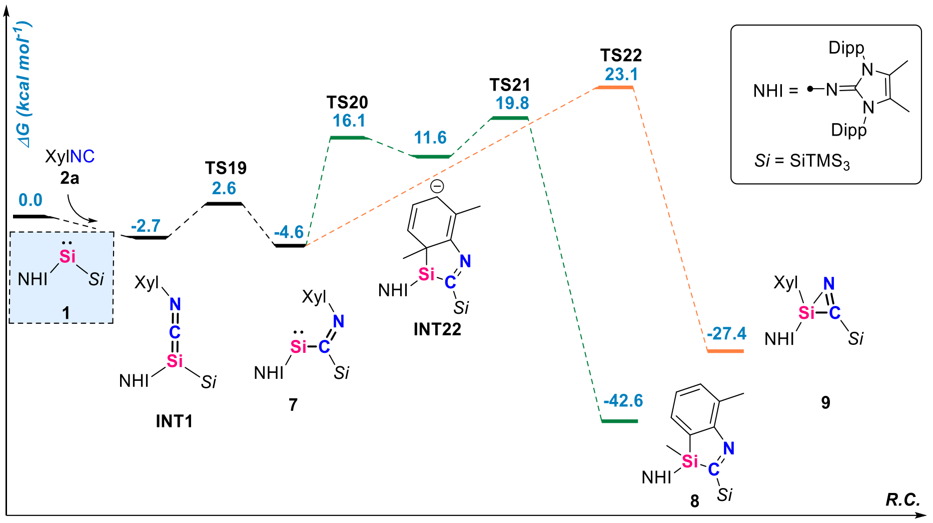

Figure S11. Free energy reaction coordinate diagram of competing reaction to the formation of **3a** at the (SMD=Benzene)PW6B95-D4/def2-QZVPP//r^{2}SCAN-3c level of theory.

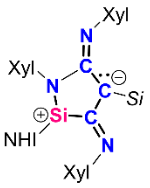

INT10

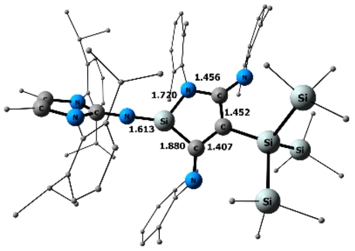

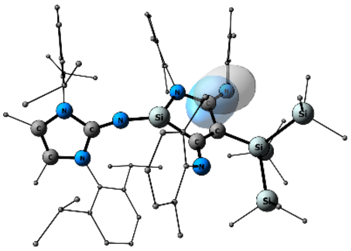

NBO 128. BD(2) N9-C15 occ. 1.90
65.37% N9 sp99.99
34.63% C15 sp99.99

Acceptor	E(2) (kcal mol^{-1})
351. BD*(2) C21-C48	5.6
352. BD*(1) C21-C49	4.2
350. BD*(1) C21-C48	4.0
324. BD*(2) N9-C15	1.4
791. RY (1) C12	1.1

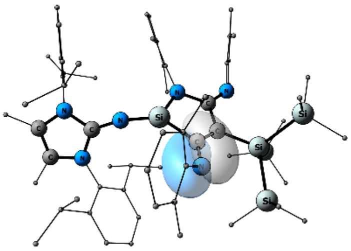

NBO 134. BD(2) N11-C13 occ. 1.85
64.78% N11 sp99.99
35.22% C13 sp99.99

Acceptor	E(2) (kcal mol^{-1})
335. BD*(2) C16-C22	16.7
299. LV (1) Si1	5.6
353. BD*(1) C22-C43	2.9
330. BD*(2) N11-C13	2.4
334. BD*(1) C16-C22	2.3

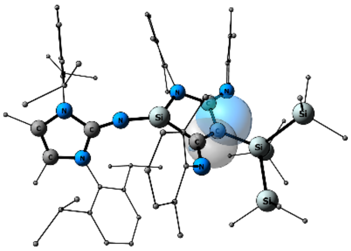

NBO 103. LP(1) C12 occ. 1.22
sp1.00

Acceptor	E(2) (kcal mol^{-1})
330. BD*(2) N11-C13	158.7
324. BD*(2) N9-C15	111.4
305. BD*(1) Si2-Si5	8.4
303. BD*(1) Si2-Si3	4.0
820. RY (4) C13	2.4

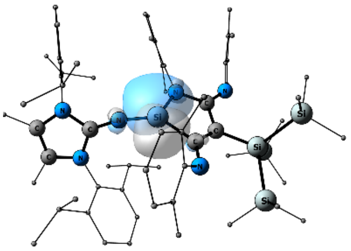

NBO 299. LV(1) Si1 occ. 0.39
sp99.99

Donor	E(2) (kcal mol^{-1})
99. LP(1) N8	35.1
126. BD(2) N8-C14	14.1
134. BD(2) N11-C13	5.6
136. BD(1) C12-C13	2.3
188. BD(1) C44-H47	2.0

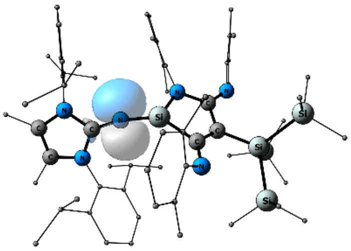

NBO 99. LP(1) N8 occ. 1.74
sp28.71

Acceptor	E(2) (kcal mol^{-1})
299. LV (1) Si1	35.1
318. BD*(1) N7-C14	23.4
326. BD*(1) N10-C14	17.4
300. BD*(1) Si1-N6	6.9
302. BD*(1) Si1-C13	6.1

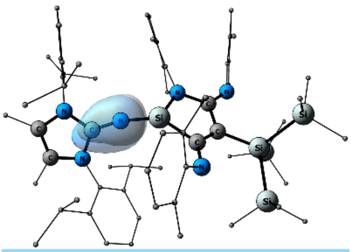

NBO 126. BD(2) N8-C14 occ. 1.90
76.81% N8 sp99.99
23.19% C14 sp99.99

Acceptor	E(2) (kcal mol^{-1})
299. LV (1) Si1	14.1
322. BD*(2) N8-C14	7.4
300. BD*(1) Si1-N6	3.3
496. RY (2) Si1	1.8
302. BD*(1) Si1-C13	1.5

Figure S12. top - Lewis structure of **INT10** derived from the NBO analysis and the optimized geometry with key bond lengths; bottom - selected NBOs of **INT10**, their atomic orbital compositions and five largest donor-acceptor interactions, according to the second-order perturbation theory.

INT10 contains a nearly planar Si center (Σθ = 356.5°) within a five-membered ring, which features two exocyclic C-N double bonds (NBO 128 and 134). The endocyclic carbanion center (NBO 103) is heavily delocalized to the adjacent π*(C-N) orbitals. The Si center exhibits a silylium cation character, having a lone vacancy p orbital (NBO 299) stabilized the lone-pair of the NHI nitrogen (NBO 99) and the π(C-N) of the NHI moiety (NBO 126). The presence of the vacant p orbital allows the electrophilic activity of the silicon center, and the reaction with RNC to form **INT11**, with CO to form **INT12** and intramolecularly with the aryl substituent to form **5a**.

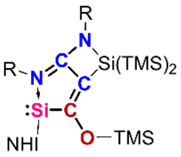

INT17

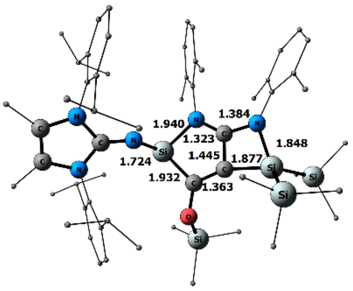

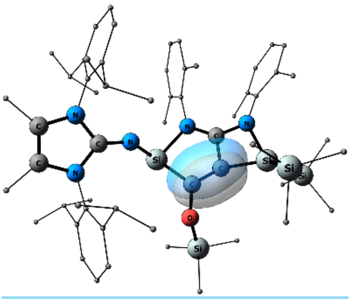

NBO 110. BD(2) N9-C11 occ. 1.94<br>73.40% N9 sp39.31<br>26.60% C11 sp99.99	
Acceptor	E(2) (kcal mol-1)
291. BD*(1) C12-C18	4.8
297. BD*(2) C14-C16	3.7
292. BD*(2) C12-C18	3.3
293. BD*(1) C12-C23	3.2
271. LV (1) Si8	2.5

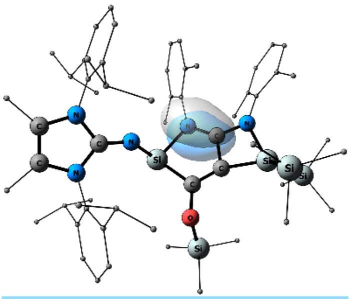

NBO 122. BD(2) C14-C16 occ. 1.82<br>40.21% C14 sp99.99<br>59.79% C16 sp1.00	
Acceptor	E(2) (kcal mol-1)
285. BD*(2) N9-C11	33.9
297. BD*(2) C14-C16	2.4
301. BD*(1) Si15-Si31	2.1
282. BD*(1) Si8-N10	2.0
302. BD*(1) Si15-Si38	1.4

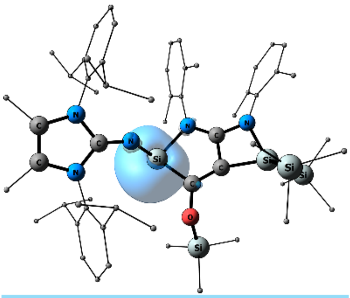

NBO 89. LP(1) Si8 occ. 1.75<br>sp0.63	
Acceptor	E(2) (kcal mol-1)
297. BD*(2) C14-C16	7.9
288. BD*(2) N10-C13	4.7
416. BD*(1) H117-C145	3.7
271. LV (1) Si8	2.1
285. BD*(2) N9-C11	1.8

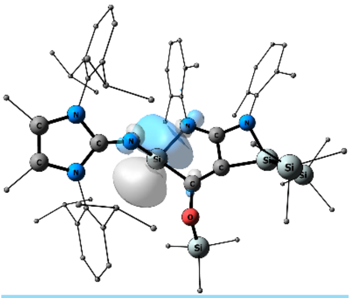

NBO 271. LV(1) Si8 occ. 0.32<br>sp12.24	
Donor	E(2) (kcal mol-1)
90. LP(1) N9	142.8
91. LP(1) N10	25.7
109. BD(1) N9-C11	7.3
111. BD(1) N9-C12	5.6
110. BD(2) N9-C11	2.5

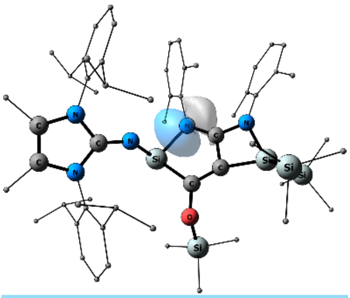

NBO 90. LP(1) N9 occ. 1.67<br>sp3.02	
Acceptor	E(2) (kcal mol-1)
271. LV (1) Si8	142.8
290. BD*(1) C11-N17	17.9
292. BD*(2) C12-C18	11.1
283. BD*(1) Si8-C14	8.5
289. BD*(1) C11-C16	2.8

Figure S13. top - Lewis structure of **INT17** derived from the NBO analysis and the optimized geometry with key bond lengths; bottom - selected NBOs of **INT17**, their atomic orbital compositions and five largest donor-acceptor interactions, according to the second-order perturbation theory.

INT17 contains a pyramidal Si center (Σθ = 295.7°) within a five membered ring of a bicyclic farmwork, which features endocyclic C-C and C-N double bonds (NBO 110 and 122). The Si center exhibits a silylene character, having a σ-type lone pair (NBO 89) and a vacant p orbital (NBO 271) stabilized predominantly by a lone-pair of the adjacent nitrogen atom (NBO 90). The presence of the vacant p orbital allows the electrophilic activity of the silicon center, and the reaction with RNC to form **INT18**.

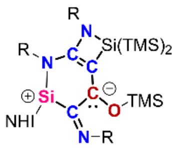

INT19

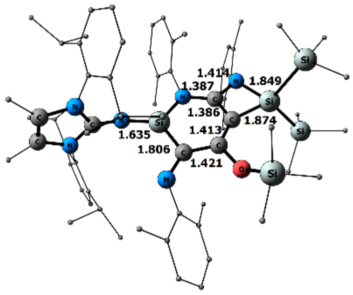

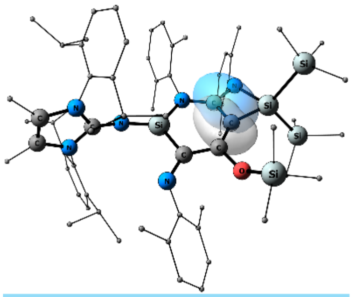

NBO 128. BD(2) C12-C18 occ. 1.79<br>43.14% C12 sp1.00<br>56.86% C18 sp1.00	
Acceptor	E(2) (kcal mol-1)
327. BD*(2) C12-C18	3.6
340. BD*(1) Si17-Si41	3.6
339. BD*(1) Si17-Si34	3.0
341. BD*(1) N19-C51	2.4
610. RY (1) N9	1.4

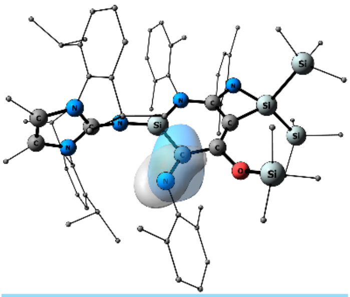

NBO 126. BD(2) C11-N16 occ. 1.83<br>31.33% C11 sp99.99<br>68.67% N16 sp99.99	
Acceptor	E(2) (kcal mol-1)
361. BD*(2) C33-C77	21.5
306. LV (1) Si8	9.7
325. BD*(2) C11-N16	5.6
362. BD*(1) C33-C82	2.2
360. BD*(1) C33-C77	1.9

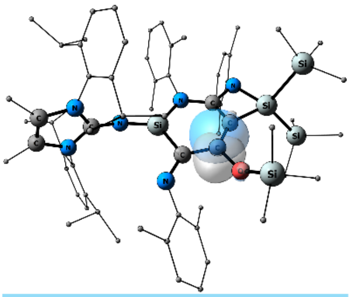

NBO 101. LP(1) C15 occ. 1.02<br>sp1.00	
Acceptor	E(2) (kcal mol-1)
325. BD*(2) C11-N16	160.9
327. BD*(2) C12-C18	93.3
359. BD*(1) O32-Si67	3.3
846. RY (1) C18	2.8
1042. RY (1) O32	2.6

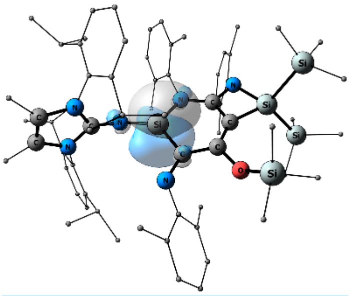

NBO 306. LV(1) Si8 occ. 0.55<br>sp42.34	
Donor	E(2) (kcal mol-1)
100. LP(1) N10	66.1
126. BD(2) C11-N16	9.7
123. BD(2) N10-C14	3.9
118. BD(1) Si8-N10	3.6
153. BD(1) H27-C55	1.1

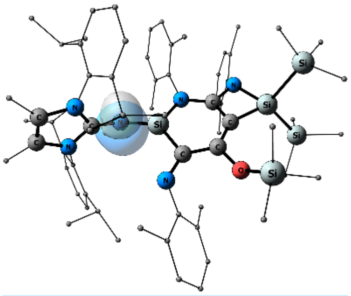

NBO 100. LP(1) N10 occ. 1.75<br>sp3.57	
Acceptor	E(2) (kcal mol-1)
306. LV (1) Si8	66.1
333. BD*(1) C14-N31	19.6
332. BD*(1) C14-N30	7.2
317. BD*(1) Si8-N10	4.1
583. RY (2) Si8	3.0

Figure S14. top - Lewis structure of **INT19** derived from the NBO analysis and the optimized geometry with key bond lengths; bottom - selected NBOs of **INT19**, their atomic orbital compositions and five largest donor-acceptor interactions, according to the second-order perturbation theory.

INT19 contains a nearly planar Si center ($\Sigma\theta$ = 359.1°) within a five membered ring of a bicyclic farmwork, which features an endocyclic C-C and an exocyclic C-N double bonds (NBO 128 and 126). The carbanion center (NBO 101) is strongly delocalized to the π*(C-C) and π*(C-N) orbitals. The Si center exhibits a silylium cation character, having a lone vacancy p orbital (NBO 306) stabilized mainly by the lone-pair of the NHI nitrogen (NBO 100). The presence of the vacant p orbital allows the electrophilic activity of the silicon center, and the reaction with CO to form **INT20**.

Table S2. Calculated energies (E_h). Thermochemistry at 298.15 K.
E_{PW6B95} - electronic energy at the PW6B95-D4/def2-QZVPP//r^{2}SCAN-3c level; G-E_{el} - Gibbs energy minus the electronic energy at the r^{2}SCAN-3c//r^{2}SCAN-3c level; G_{cds} - cavity term and G_{enp} - electrostatic contribution at the r^{2}SCAN-3c(SMD=Beneze)//r^{2}SCAN-3c level; G_{conc} - concentration-induced free-energy shift (G_{conc} = RTln(24.5)); G_{PW6B95} - free energy at the (SMD=Benzene)PW6B95-D4/def2-QZVPP/ //r^{2}SCAN-3c level (G_{PW6B95} = E_{PW6B95} + [G-E_{el}] + G_{cds} + G_{enp} + G_{conc}).

ICN	Compound	E_{PW6B95}	G-E_{el}(298.15K)	G_{cds}(Benzene)	G_{enp}(Benzene)	G_{conc}(298.15K)	G_{PW6B95}
3930516	**1**	-3104.66019	0.87938	-0.01550	-0.01617	0.00302	-3103.80946
3159596	**XyINC**	-403.80785	0.11843	-0.00670	-0.00520	0.00302	-403.69831
3931668	**INT1**	-3508.50762	1.02840	-0.01819	-0.01774	0.00302	-3507.51213
3939328	**TS1**	-3912.30968	1.17334	-0.02256	-0.02357	0.00302	-3911.17944
3955087	**INT2**	-3912.31748	1.17486	-0.02072	-0.02225	0.00302	-3911.18256
3953196	**TS2**	-3912.31363	1.17499	-0.02138	-0.02276	0.00302	-3911.17976
3933095	**INT3**	-3912.32460	1.17583	-0.02107	-0.02275	0.00302	-3911.18957
3931242	**TS3**	-3912.31433	1.17595	-0.02075	-0.02236	0.00302	-3911.17847
3931940	**INT4**	-3912.34445	1.17864	-0.02129	-0.02209	0.00302	-3911.20616
4755847	**TS4**	-3912.32698	1.17913	-0.02445	-0.02261	0.00302	-3911.19189
3930517	**3a**	-3912.37972	1.17909	-0.02047	-0.02116	0.00302	-3911.23925
4075862	**TS5**	-3912.33300	1.17411	-0.02069	-0.02226	0.00302	-3911.19882
3047439_3	**INT5**	-3912.34225	1.17657	-0.02140	-0.02104	0.00302	-3911.20510
4075863	**TS6**	-3912.33600	1.17743	-0.02082	-0.02183	0.00302	-3911.19820
3930518	**4a**	-3912.42628	1.17844	-0.02239	-0.02084	0.00302	-3911.28805
3960970	**INT6**	-3912.41229	1.17541	-0.02322	-0.02182	0.00302	-3911.27891
625494	**TS7**	-3912.39407	1.17672	-0.02356	-0.02184	0.00302	-3911.25973
3978170	**INT7**	-3912.40423	1.17669	-0.02487	-0.02243	0.00302	-3911.27182
3969042	**INT8**	-4316.23996	1.32637	-0.02975	-0.02588	0.00302	-4314.96621
3976803	**TS8**	-4316.22855	1.32830	-0.02611	-0.02469	0.00302	-4314.94802
3978980	**INT9**	-4316.24664	1.33125	-0.02548	-0.02531	0.00302	-4314.96316
4787506	**TS9**	-4316.24269	1.33243	-0.02682	-0.02580	0.00302	-4314.95986
3946070	**INT10**	-4316.25856	1.33095	-0.02666	-0.02628	0.00302	-4314.97753
3938298	**TS10**	-4316.25785	1.33300	-0.02581	-0.02600	0.00302	-4314.97364
3931611	**5a**	-4316.28411	1.33586	-0.02419	-0.02367	0.00302	-4314.99310
613402	**INT11**	-4720.10056	1.47771	-0.03238	-0.02957	0.00302	-4718.68178
3931020	**TS11**	-4720.08727	1.48029	-0.02892	-0.02925	0.00302	-4718.66213
3930519	**6a**	-4720.11839	1.48452	-0.02806	-0.02683	0.00302	-4718.68575
4300111	**INT12**	-4429.76537	1.33574	-0.02570	-0.02409	0.00302	-4428.47641
4302645	**TS12**	-4429.76658	1.33888	-0.02656	-0.02488	0.00302	-4428.47612
4312763	**INT13**	-4429.79147	1.33849	-0.02450	-0.02480	0.00302	-4428.49926
3051225_1	**TS13**	-4429.76937	1.33881	-0.02584	-0.02483	0.00302	-4428.47820
3054299_3	**INT14**	-4429.79552	1.33515	-0.03050	-0.02669	0.00302	-4428.51455
3056451	**TS14**	-4429.76295	1.33726	-0.02957	-0.02621	0.00302	-4428.47846
3059987_1	**INT15**	-4429.77113	1.33779	-0.02696	-0.02628	0.00302	-4428.48357
3099365_2	**INT16 SD**	-4025.93366	1.18709	-0.02160	-0.02227	0.00302	-4024.78742
3090124_1	**TS15 SD**	-4025.92736	1.18686	-0.02258	-0.02213	0.00302	-4024.78219
3099365_1	**INT17**	-4025.96816	1.18707	-0.02323	-0.02259	0.00302	-4024.82389
4329951	**INT18**	-4429.78389	1.33649	-0.02813	-0.02543	0.00302	-4428.49793
4376527	**TS16**	-4429.77637	1.33573	-0.02498	-0.02644	0.00302	-4428.48904
4302631	**INT19**	-4429.82334	1.33711	-0.02764	-0.02511	0.00302	-4428.53596
4306918	**INT20**	-4543.33636	1.34440	-0.02571	-0.02515	0.00302	-4542.03980

4312622	**TS17**	-4543.34090	1.34573	-0.02554	-0.02634	0.00302	-4542.04404
4301213	**INT21**	-4543.37502	1.34682	-0.02565	-0.02599	0.00302	-4542.07683
4300145	**TS18**	-4947.17962	1.49546	-0.02649	-0.02840	0.00302	-4945.73603
3930816	**10a**	-4947.25614	1.50105	-0.02799	-0.02730	0.00302	-4945.80736
4083960	**TS19**	-3508.49830	1.02769	-0.01691	-0.01919	0.00302	-3507.50368
4089702	**7**	-3508.50592	1.02543	-0.01852	-0.01912	0.00302	-3507.51511
663928	**TS20**	-3508.47468	1.02859	-0.01979	-0.01920	0.00302	-3507.48206
4089821	**INT22**	-3508.48130	1.02785	-0.02076	-0.01815	0.00302	-3507.48935
4085363	**TS21**	-3508.46673	1.02635	-0.02090	-0.01796	0.00302	-3507.47622
4089822	**8**	-3508.57077	1.02858	-0.01755	-0.01896	0.00302	-3507.57568
663926	**TS22**	-3508.46304	1.02558	-0.01865	-0.01786	0.00302	-3507.47096
4093431	**9**	-3508.54384	1.02657	-0.01940	-0.01781	0.00302	-3507.55147

4. Appendix: Cartesian coordinates of the optimized geometries

Cartesian coordinates and energies of the optimized geometries at the r^{2}SCAN-3c level of theory

Calculated energies and coordinates of **1**

Electronic energy ... -3099.74830769 Eh
Total Enthalpy ... -3098.71942234 Eh
Final Gibbs free energy ... -3098.86892804 Eh

CARTESIAN COORDINATES (ANGSTROEM)

Si	3.609881	13.881070	5.831003
Si	5.625594	13.799814	4.502697
Si	7.804391	14.533260	5.027172
Si	4.843404	15.057999	2.662876
Si	5.680564	11.540772	3.819304
N	4.068200	13.844627	7.446572
N	3.290764	12.578573	9.327839
N	3.301615	14.760712	9.530478
C	3.577359	13.742675	8.636092
C	2.904310	12.876756	10.647014
C	2.904648	14.225692	10.771031
C	3.421992	11.273265	8.764745
C	4.574418	10.531325	9.059719
C	4.693275	9.259314	8.496526
H	5.579296	8.663845	8.695235
C	3.702007	8.757149	7.667091
H	3.812406	7.769121	7.229064
C	2.573921	9.515977	7.378191
H	1.820654	9.111795	6.710616
C	2.407904	10.792388	7.916353
C	5.704094	11.119212	9.882878
H	5.307999	11.965799	10.455333
C	6.782550	11.673781	8.940450
H	7.242407	10.862180	8.364313
H	7.573124	12.177248	9.508542
H	6.351659	12.388659	8.232683
C	6.304998	10.123376	10.880373
H	6.850737	9.320725	10.373342
H	5.534344	9.662371	11.507502
H	7.018935	10.636244	11.533479
C	1.175393	11.626800	7.607244
H	1.509381	12.664161	7.475745
C	0.175529	11.599997	8.774535
H	-0.730538	12.154699	8.506435
H	0.592725	12.061576	9.673565
H	-0.113978	10.569541	9.011613
C	0.478530	11.227688	6.305434
H	1.181221	11.194185	5.467141
H	-0.290023	11.969229	6.065864
H	-0.019669	10.254874	6.389226
C	2.617132	11.803912	11.632806
H	1.957164	11.038083	11.210646
H	2.135207	12.224015	12.518307
H	3.533375	11.294004	11.956874
C	2.577870	15.101423	11.925525
H	1.874109	15.891276	11.639058
H	3.467693	15.598887	12.331403
H	2.126525	14.512561	12.726860
C	3.379943	16.152709	9.218941
C	2.349921	16.740074	8.464750
C	2.439658	18.109571	8.211988
H	1.667944	18.600276	7.628561
C	3.509023	18.856584	8.689328
H	3.556682	19.922016	8.482331
C	4.524834	18.248553	9.413351
H	5.363287	18.843623	9.761005
C	4.485121	16.880419	9.685516
C	1.175684	15.919970	7.958583
H	1.565538	14.936956	7.668524
C	0.517300	16.510526	6.710424
H	1.257475	16.735693	5.936248
H	-0.044098	17.424687	6.935701
H	-0.189702	15.785716	6.294736
C	0.129118	15.705039	9.063264
H	-0.235644	16.667440	9.440848
H	0.537926	15.135077	9.902758
H	-0.726753	15.146997	8.667359
C	5.644175	16.182935	10.371942
H	5.257125	15.287987	10.873632
C	6.645106	15.706121	9.306675
H	6.149432	15.077904	8.559469
H	7.456479	15.131559	9.768641
H	7.086671	16.563874	8.785647
C	6.340086	17.043507	11.429130
H	6.893148	17.873421	10.976565
H	7.065991	16.437876	11.981424
H	5.626292	17.463240	12.146206
C	8.518958	13.772287	6.601316
H	8.509498	12.678225	6.563419
H	7.960137	14.079743	7.488631
H	9.559592	14.098158	6.726325
C	7.789238	16.413130	5.239723
H	7.512916	16.917039	4.307069
H	8.778898	16.779603	5.539607
H	7.066495	16.712694	6.007033
C	9.006839	14.091826	3.630882
H	9.144935	13.006700	3.564996
H	9.989993	14.540508	3.823213
H	8.657844	14.443804	2.655485
C	4.218865	16.731286	3.292915
H	3.858635	17.354132	2.464537
H	5.005280	17.284738	3.817392
H	3.387864	16.591245	3.994864
C	6.159349	15.388935	1.343084
H	6.593017	14.458504	0.960910
H	6.976433	16.003374	1.736648
H	5.715740	15.927993	0.496471
C	3.382438	14.187774	1.832150
H	2.963056	14.819812	1.039296
H	2.587999	13.982391	2.558561
H	3.679252	13.235144	1.380248
C	6.435491	11.339544	2.095701
H	5.815837	11.820350	1.331053
H	6.526442	10.277318	1.835529
H	7.433182	11.788528	2.044047
C	6.702513	10.525489	5.045288
H	6.665034	9.460258	4.783790
H	6.301296	10.638550	6.058911
H	7.753884	10.832500	5.058330
C	3.944317	10.788730	3.813152
H	3.235721	11.376682	3.220950
H	3.558119	10.725554	4.837688
H	3.972136	9.770770	3.403639

Calculated energies and coordinates of **XylNC**

Electronic energy ... -402.98646605 Eh
Total Enthalpy ... -402.82315204 Eh
Final Gibbs free energy ... -402.86803826 Eh

CARTESIAN COORDINATES (ANGSTROEM)

C	0.428563	-0.135658	-3.371962
N	0.821086	0.961819	-3.222681
C	1.288471	2.247940	-3.037399
C	0.420505	3.203435	-2.483922
C	2.610121	2.541612	-3.411159
C	-0.980733	2.830226	-2.104812
C	0.915938	4.492906	-2.306665

C	3.489749	1.479179	-3.997529
C	3.057287	3.845788	-3.213559
H	-1.537645	2.455664	-2.970995
H	-0.986956	2.028737	-1.357587
H	-1.513402	3.691096	-1.695189
C	2.220421	4.812541	-2.667020
H	0.267392	5.253290	-1.880541
H	3.055863	1.069627	-4.916692
H	4.478839	1.879940	-4.229266
H	3.608111	0.638092	-3.305132
H	4.075200	4.102377	-3.493419
H	2.588021	5.823858	-2.520950

Calculated energies and coordinates of **INT1**

Electronic energy ... -3502.78203876 Eh
Total Enthalpy ... -3501.58731389 Eh
Final Gibbs free energy ... -3501.75364134 Eh

CARTESIAN COORDINATES (ANGSTROEM)

Si	-0.160550	0.277081	0.966668
Si	-1.524779	-1.090259	2.281243
Si	-3.430016	-2.229838	1.456991
Si	-2.067611	0.199499	4.196955
Si	-0.011983	-2.746115	3.054914
N	2.359730	-1.143763	-1.823631
N	0.664769	-0.475332	-0.301094
N	1.370642	0.751916	-2.288840
N	-1.074869	3.065742	1.032087
C	5.120273	-0.154711	-0.611582
H	5.988392	-0.792044	-0.817664
H	5.488682	0.788586	-0.193029
H	4.623759	0.076533	-1.556476
C	4.184292	-0.838128	0.401272
H	3.287704	-0.211574	0.511777
C	3.704995	-2.201743	-0.080091
C	2.796452	-2.321433	-1.147955
C	1.383151	-0.273309	-1.345707
C	4.888019	-0.876775	1.760623
H	4.292542	-1.381057	2.525441
H	5.068398	0.148109	2.100385
H	5.863447	-1.373489	1.692232
C	4.139186	-3.382760	0.523409
H	4.833348	-3.338628	1.355389
C	3.690569	-4.621764	0.081899
H	4.043690	-5.526421	0.569190
C	2.789353	-4.711722	-0.968577
H	2.439860	-5.687266	-1.291721
C	2.322210	-3.560307	-1.604533
C	1.280068	-3.638835	-2.706298
H	1.389148	-2.752478	-3.342153
C	1.435303	-4.870163	-3.603274
H	2.457596	-4.968649	-3.984324
H	0.756074	-4.792678	-4.458631
H	1.180699	-5.793967	-3.072942
C	-0.128582	-3.582522	-2.095637
H	-0.310396	-4.466818	-1.474262
H	-0.890557	-3.560283	-2.884146
H	-0.250714	-2.696495	-1.463486
C	2.868850	-0.701465	-3.053972
C	3.907655	-1.466902	-3.789890
H	4.258851	-0.891133	-4.648571
H	3.529019	-2.429055	-4.156238
H	4.769272	-1.685843	-3.148617
C	2.257204	0.471052	-3.343289
C	2.384140	1.376948	-4.513181
H	3.018989	0.920538	-5.275312
H	2.821104	2.346397	-4.242370
H	1.405892	1.585344	-4.961438
C	0.467169	1.854098	-2.292475
C	0.953866	3.118973	-1.916983
C	0.071098	4.194514	-1.981853
H	0.406685	5.189686	-1.709707
C	-1.246524	4.013767	-2.388712
H	-1.925045	4.862144	-2.409292
C	-1.699775	2.757398	-2.759690
H	-2.728173	2.636332	-3.090330
C	-0.847890	1.650257	-2.730180
C	-1.335314	0.296217	-3.209620
H	-0.532498	-0.431941	-3.051980
C	-1.647273	0.328032	-4.713257
H	-2.460986	1.028261	-4.932505
H	-1.954421	-0.665585	-5.058025
H	-0.773978	0.636620	-5.296937
C	-2.550307	-0.189997	-2.413380
H	-2.327927	-0.206843	-1.340745
H	-2.825917	-1.201685	-2.730305
H	-3.422607	0.453743	-2.577236
C	2.379587	3.283695	-1.419284
H	3.008725	2.568457	-1.963942
C	2.950984	4.681460	-1.667781
H	2.825234	4.993106	-2.710348
H	4.020191	4.690323	-1.432806
H	2.475414	5.433387	-1.028088
C	2.478427	2.919778	0.070899
H	1.841953	3.575141	0.674229
H	3.513666	3.019232	0.417314
H	2.148144	1.893686	0.257607
C	-3.076201	-3.228383	-0.105055
H	-4.009319	-3.664768	-0.483798
H	-2.377004	-4.046166	0.093270
H	-2.645019	-2.609128	-0.895002
C	-4.009172	-3.467662	2.768656
H	-4.916251	-3.977324	2.419336
H	-4.238341	-2.990241	3.725951
H	-3.248015	-4.234327	2.950325
C	-4.862987	-1.058004	1.073874
H	-4.634754	-0.431926	0.205000
H	-5.092864	-0.394140	1.914389
H	-5.766280	-1.636792	0.843821
C	-0.471392	0.651748	5.102068
H	0.213196	1.186547	4.434241
H	0.050172	-0.234993	5.477066
H	-0.690757	1.301303	5.958595
C	-3.178403	-0.794266	5.364429
H	-3.278770	-0.259658	6.317421
H	-2.785799	-1.792856	5.577000
H	-4.183561	-0.907392	4.943161
C	-2.996155	1.807333	3.845809
H	-3.220638	2.301370	4.799947
H	-3.949040	1.630513	3.335647
H	-2.409768	2.503078	3.239179
C	-0.507451	-3.449318	4.741861
H	0.188881	-4.248797	5.024709
H	-1.516554	-3.874541	4.729379
H	-0.475272	-2.686926	5.527101
C	1.712764	-1.999244	3.183182
H	2.428036	-2.751795	3.539357
H	1.755046	-1.132529	3.850422
H	2.027190	-1.675040	2.185275
C	0.118339	-4.187466	1.840691
H	0.397737	-3.825331	0.845534
H	-0.816417	-4.751626	1.755870
H	0.901071	-4.881057	2.173463
C	-2.064728	4.031100	0.816819
C	-3.388226	3.713673	0.445009
C	-4.299960	4.758439	0.296785
H	-5.323386	4.523885	0.014288
C	-3.921793	6.080157	0.496856
H	-4.647777	6.878144	0.370228
C	-2.617867	6.377962	0.877107
H	-2.325055	7.409997	1.052013
C	-1.679544	5.366173	1.058138
C	-0.281546	5.688640	1.499144
H	0.001327	5.095228	2.374537
H	-0.189192	6.750331	1.743505
H	0.445838	5.450020	0.715695
C	-3.811539	2.303918	0.193070
H	-3.327918	1.911486	-0.707339
H	-4.896491	2.240907	0.068097
H	-3.506341	1.636615	1.005455

C	-0.961376	1.869327	0.770097

Calculated energies and coordinates of **TS1**

Electronic energy ... -3905.76513624 Eh
Total Enthalpy ... -3904.40604572 Eh
Final Gibbs free energy ... -3904.59179719 Eh

CARTESIAN COORDINATES (ANGSTROEM)

Si	-0.343204	-0.054811	0.560264
Si	-1.588510	-1.582377	1.876888
Si	-3.476056	-2.464701	0.723754
Si	-2.549746	-0.401552	3.705315
Si	-0.192678	-3.373180	2.599123
N	2.214982	-1.211882	-2.254418
N	0.400505	-0.760017	-0.786072
N	1.322060	1.701693	2.228845
N	0.717069	0.189230	-3.009085
N	-2.967878	2.818871	0.034278
C	4.737131	0.642855	-1.264260
H	5.759905	0.247731	-1.274134
H	4.788577	1.712732	-1.032725
H	4.314941	0.540680	-2.267635
C	3.891735	-0.083118	-0.205034
H	2.862697	0.293097	-0.271767
C	3.833073	-1.578704	-0.465950
C	2.969147	-2.107934	-1.437692
C	1.032611	-0.574142	-1.885780
C	0.860907	0.680694	1.705755
C	2.233737	1.936178	3.268109
C	2.844376	3.210730	3.257673
C	3.696643	3.563729	4.296703
H	4.173634	4.540343	4.282605
C	3.927031	2.690229	5.355421
H	4.577305	2.983826	6.174273
C	2.490601	1.027014	4.316758
C	1.947121	-0.368362	4.342052
H	2.655292	-1.067208	3.879493
H	1.013694	-0.463662	3.782510
H	1.792891	-0.699822	5.373968
C	3.321855	1.442685	5.360018
H	3.506621	0.754379	6.181747
C	4.405630	0.262718	1.192423
H	3.851977	-0.275808	1.967190
H	4.278572	1.333241	1.379003
H	5.472656	0.037303	1.302803
C	4.634692	-2.476705	0.239020
H	5.310418	-2.110356	1.004415
C	4.576597	-3.840692	-0.017256
H	5.213025	-4.521180	0.541710
C	3.696855	-4.341683	-0.965814
H	3.646599	-5.412550	-1.136398
C	2.869882	-3.483948	-1.692371
C	1.832652	-4.029118	-2.656313
H	1.602589	-3.249382	-3.392535
C	2.299412	-5.269004	-3.423568
H	3.266349	-5.105326	-3.911590
H	1.564998	-5.526030	-4.193917
H	2.395692	-6.140427	-2.767142
C	0.533363	-4.318950	-1.887669
H	0.696716	-5.109866	-1.145835
H	-0.255379	-4.650314	-2.573010
H	0.186901	-3.423554	-1.360459
C	2.577062	-0.893869	-3.570525
C	3.781625	-1.478930	-4.213603
H	3.972138	-0.990002	-5.171263
H	3.663041	-2.553653	-4.398537
H	4.669778	-1.360992	-3.582540
C	1.650898	-0.027276	-4.040324
C	1.525068	0.635721	-5.363852
H	2.416321	0.440409	-5.963950
H	1.408467	1.720451	-5.260653
H	0.653350	0.272497	-5.921973
C	-0.425126	1.023780	-3.212128
C	-0.390763	2.351656	-2.758116
C	-1.435612	3.190783	-3.148696
H	-1.445043	4.226172	-2.824186
C	-2.470889	2.719816	-3.945191
H	-3.262487	3.395437	-4.257953
C	-2.506919	1.390881	-4.341374
H	-3.334182	1.035397	-4.946789
C	-1.479764	0.516146	-3.988039
C	-1.515654	-0.944919	-4.395673
H	-0.480588	-1.290840	-4.511777
C	-2.235481	-1.194604	-5.723463
H	-3.314765	-1.028728	-5.636781
H	-2.094250	-2.236314	-6.029459
H	-1.855142	-0.548364	-6.522107
C	-2.147480	-1.778689	-3.271599
H	-1.624058	-1.619024	-2.323863
H	-2.110587	-2.846017	-3.517710
H	-3.196875	-1.496519	-3.129553
C	0.748044	2.858134	-1.887064
H	0.978586	2.063536	-1.162270
C	2.018880	3.141991	-2.705352
H	2.427681	2.238413	-3.164992
H	2.793781	3.564662	-2.055677
H	1.807514	3.871312	-3.496544
C	0.371978	4.106045	-1.087615
H	0.270230	4.985707	-1.733961
H	1.158887	4.325985	-0.361642
H	-0.565325	3.971293	-0.542259
C	-3.008917	-3.834277	-0.497300
H	-3.830805	-3.982624	-1.209289
H	-2.842867	-4.781022	0.025181
H	-2.108082	-3.602648	-1.069271
C	-4.648345	-3.276187	1.974410
H	-5.437308	-3.811134	1.430283
H	-5.134017	-2.544948	2.628360
H	-4.130441	-4.004643	2.607914
C	-4.462424	-1.163735	-0.218685
H	-3.838733	-0.636521	-0.945098
H	-4.884181	-0.405871	0.448230
H	-5.289706	-1.653496	-0.749009
C	-1.377347	0.768452	4.623133
H	-0.756626	1.378898	3.962414
H	-0.707428	0.205712	5.281167
H	-1.967676	1.443980	5.255667
C	-3.206672	-1.591138	5.025038
H	-3.877817	-1.043792	5.699388
H	-2.390693	-2.000054	5.628284
H	-3.767989	-2.429316	4.602714
C	-4.033340	0.601897	3.087453
H	-4.364259	1.300361	3.866570
H	-4.875141	-0.060125	2.857013
H	-3.810161	1.175045	2.182698
C	-0.093438	-3.455190	4.489403
H	0.702656	-4.149515	4.786937
H	-1.033937	-3.826048	4.910554
H	0.119402	-2.484230	4.945719
C	1.529348	-3.136714	1.879669
H	1.520057	-3.317449	0.800133
H	2.248961	-3.828361	2.334965
H	1.885665	-2.112046	2.018026
C	-0.802547	-5.077600	2.044002
H	-0.778143	-5.175903	0.954760
H	-1.822785	-5.287088	2.382625
H	-0.145221	-5.848711	2.466141
C	-3.157076	4.116125	0.463269
C	-4.056699	4.931903	-0.243570
C	-4.201441	6.250198	0.181119
H	-4.887437	6.903910	-0.350679
C	-3.485768	6.730670	1.272665
H	-3.610421	7.762462	1.587834
C	-2.618375	5.895530	1.967703
H	-2.070695	6.272141	2.827282
C	-2.437606	4.568768	1.582818
C	-1.537674	3.638878	2.332811

H	-0.792944	3.163669	1.684782
H	-2.119101	2.822865	2.775483
H	-1.014741	4.159999	3.138408
C	-4.838170	4.378141	-1.395925
H	-4.181573	3.883548	-2.117866
H	-5.396488	5.168329	-1.903340
H	-5.549814	3.617396	-1.053212
C	2.585072	4.159273	2.123881
H	1.514746	4.369249	2.025374
H	2.906433	3.729845	1.167443
H	3.119250	5.101006	2.275888
C	-2.722713	1.737653	-0.366413

Calculated energies and coordinates of **INT2**

Electronic energy ... -3905.78258998 Eh
Total Enthalpy ... -3904.42278346 Eh
Final Gibbs free energy ... -3904.60771890 Eh

CARTESIAN COORDINATES (ANGSTROEM)

Si	-0.414973	0.042977	0.613091
Si	-1.699711	-1.458527	1.929313
Si	-3.613379	-2.272950	0.772279
Si	-2.598195	-0.261183	3.780786
Si	-0.298546	-3.252434	2.631521
N	2.209980	-1.099747	-2.194587
N	0.472129	-0.574789	-0.677058
N	1.290518	1.660265	2.396217
N	0.822431	0.456409	-2.866219
N	-2.632281	1.963005	-0.176849
C	4.880539	0.528642	-1.173767
H	5.870065	0.057438	-1.211716
H	5.018293	1.583055	-0.908352
H	4.440709	0.492215	-2.174268
C	3.995365	-0.166061	-0.126571
H	2.996833	0.289185	-0.157464
C	3.818056	-1.642312	-0.438612
C	2.906095	-2.074148	-1.415890
C	1.082809	-0.388848	-1.782937
C	0.931215	0.653726	1.774853
C	2.360617	1.886269	3.275787
C	3.059547	3.100710	3.111276
C	4.078859	3.420760	3.998711
H	4.627425	4.349383	3.862080
C	4.392408	2.573531	5.058603
H	5.176719	2.841847	5.760073
C	2.689759	1.000887	4.321562
C	2.036937	-0.338838	4.496799
H	2.672287	-1.136734	4.092731
H	1.082853	-0.405422	3.971690
H	1.880125	-0.554718	5.559304
C	3.696470	1.384033	5.212288
H	3.945239	0.713779	6.032330
C	4.554180	0.085606	1.274122
H	3.965889	-0.433914	2.036655
H	4.518014	1.155133	1.503029
H	5.600594	-0.229729	1.361087
C	4.555554	-2.620282	0.229045
H	5.264264	-2.329174	0.996704
C	4.391743	-3.967774	-0.064861
H	4.980552	-4.710721	0.466128
C	3.466081	-4.371455	-1.015415
H	3.331236	-5.429795	-1.215901
C	2.700348	-3.431178	-1.706833
C	1.615341	-3.873073	-2.670372
H	1.417146	-3.050821	-3.368442
C	1.995295	-5.104183	-3.497686
H	2.963421	-4.976784	-3.993966
H	1.236741	-5.284252	-4.266704
H	2.048819	-6.007393	-2.880691
C	0.317255	-4.121206	-1.886022
H	0.453049	-4.942863	-1.172552
H	-0.498827	-4.391830	-2.565986
H	0.026880	-3.226068	-1.325392
C	2.577377	-0.758093	-3.502779
C	3.725679	-1.408046	-4.185055
H	3.938248	-0.903556	-5.129816
H	3.524443	-2.464533	-4.400830
H	4.628696	-1.376537	-3.564767
C	1.718328	0.197249	-3.924032
C	1.623099	0.910885	-5.222669
H	2.480661	0.658628	-5.850104
H	1.604592	1.997725	-5.085138
H	0.711476	0.643338	-5.771153
C	-0.297929	1.325860	-3.044831
C	-0.228455	2.655809	-2.588553
C	-1.290805	3.500541	-2.915986
H	-1.286695	4.531582	-2.582963
C	-2.371087	3.044783	-3.659039
H	-3.182528	3.726861	-3.898208
C	-2.425131	1.726372	-4.083945
H	-3.280699	1.381860	-4.656732
C	-1.385602	0.842987	-3.792371
C	-1.455041	-0.606038	-4.237407
H	-0.430612	-0.991257	-4.307358
C	-2.108593	-0.789370	-5.611018
H	-3.183026	-0.579002	-5.581001
H	-1.992932	-1.827605	-5.939054
H	-1.657059	-0.138451	-6.367399
C	-2.185268	-1.439905	-3.175725
H	-1.715728	-1.320221	-2.193884
H	-2.173033	-2.501849	-3.446048
H	-3.231491	-1.122593	-3.093549
C	0.943414	3.162198	-1.754544
H	1.081124	2.442971	-0.931005
C	2.261573	3.231421	-2.545531
H	2.607832	2.251952	-2.879812
H	3.046522	3.663468	-1.914663
H	2.142216	3.880878	-3.420830
C	0.681553	4.540913	-1.141543
H	0.662249	5.315743	-1.917018
H	1.490114	4.794131	-0.450868
H	-0.261826	4.587809	-0.590483
C	-3.198464	-3.634544	-0.472929
H	-4.050520	-3.781764	-1.148886
H	-3.007572	-4.583502	0.037137
H	-2.323287	-3.397490	-1.081470
C	-4.838038	-3.043880	1.995867
H	-5.641950	-3.536701	1.434201
H	-5.299967	-2.295757	2.648070
H	-4.363896	-3.800367	2.630477
C	-4.536540	-0.907075	-0.147502
H	-3.905948	-0.441174	-0.908908
H	-4.868866	-0.115132	0.530501
H	-5.421607	-1.332466	-0.638346
C	-1.364629	0.801925	4.743095
H	-0.720624	1.416832	4.108474
H	-0.716204	0.183124	5.371292
H	-1.925095	1.471036	5.408642
C	-3.361306	-1.436236	5.055860
H	-3.970124	-0.854306	5.759753
H	-2.587070	-1.949781	5.633842
H	-4.005863	-2.196165	4.605735
C	-3.992376	0.877782	3.189796
H	-4.332592	1.499433	4.027608
H	-4.852867	0.299874	2.836084
H	-3.686824	1.547639	2.379673
C	-0.209515	-3.344520	4.520145
H	0.562126	-4.066727	4.815757
H	-1.162391	-3.684592	4.939266
H	0.037775	-2.382962	4.979776
C	1.428063	-3.035962	1.920300
H	1.419548	-3.185293	0.835904
H	2.123833	-3.762041	2.359377
H	1.812074	-2.025475	2.089594
C	-0.948758	-4.932823	2.054098
H	-0.933367	-5.011819	0.962956
H	-1.971329	-5.124915	2.396339
H	-0.306046	-5.725861	2.457559
C	-3.114390	3.221130	0.165500
C	-4.304598	3.638531	-0.461647

C	-4.777587	4.918763	-0.186833
H	-5.690193	5.259871	-0.668331
C	-4.101708	5.754176	0.695745
H	-4.481621	6.751710	0.896789
C	-2.948728	5.311299	1.332917
H	-2.434952	5.957969	2.039899
C	-2.435742	4.037751	1.091248
C	-1.226260	3.545536	1.818335
H	-0.457214	3.156179	1.144596
H	-1.481486	2.708204	2.478143
H	-0.793339	4.340429	2.430200
C	-5.055046	2.711688	-1.370928
H	-4.379798	2.204341	-2.065450
H	-5.814700	3.256020	-1.938632
H	-5.557454	1.927761	-0.792232
C	2.723179	3.998778	1.958269
H	1.660542	4.262752	1.964725
H	2.911110	3.494950	1.001445
H	3.318276	4.915777	1.986838
C	-1.776429	1.123411	-0.039446

Calculated energies and coordinates of **TS2**

Electronic energy ... -3905.77866229 Eh
Total Enthalpy ... -3904.42007512 Eh
Final Gibbs free energy ... -3904.60366977 Eh

CARTESIAN COORDINATES (ANGSTROEM)

Si	-0.329589	-0.001910	0.615009
Si	-1.619895	-1.476619	1.952018
Si	-3.540214	-2.241550	0.780577
Si	-2.516038	-0.278022	3.805626
Si	-0.251969	-3.294680	2.655511
N	2.134606	-1.160327	-2.249647
N	0.325248	-0.630352	-0.792796
N	1.315147	1.678258	2.327708
N	0.731252	0.349297	-2.979762
N	-2.280849	2.269640	0.343375
C	4.869419	0.512881	-1.302935
H	5.850399	0.022681	-1.300403
H	5.020517	1.573221	-1.071026
H	4.452116	0.450171	-2.311808
C	3.945206	-0.126562	-0.253383
H	2.952899	0.333962	-0.342410
C	3.773063	-1.615764	-0.499886
C	2.851699	-2.096880	-1.444027
C	0.988006	-0.462749	-1.873489
C	1.049032	0.625755	1.706010
C	2.345795	1.897395	3.262277
C	2.997296	3.148824	3.201955
C	3.951884	3.469621	4.158742
H	4.461080	4.428383	4.096814
C	4.248457	2.587469	5.194330
H	4.979777	2.855926	5.950922
C	2.663135	0.980429	4.287295
C	2.062794	-0.391740	4.381552
H	2.723365	-1.141244	3.928523
H	1.110022	-0.462906	3.852852
H	1.921443	-0.675011	5.430589
C	3.599297	1.364062	5.252126
H	3.829916	0.667008	6.055104
C	4.457359	0.190243	1.151587
H	3.840048	-0.291697	1.914946
H	4.412636	1.269354	1.327900
H	5.500598	-0.118179	1.287254
C	4.538193	-2.558286	0.187945
H	5.254084	-2.229058	0.933495
C	4.394994	-3.917018	-0.060812
H	5.005018	-4.631736	0.484803
C	3.465698	-4.368448	-0.986722
H	3.351774	-5.434886	-1.154851
C	2.671639	-3.465479	-1.694829
C	1.592120	-3.954441	-2.641808
H	1.374568	-3.152812	-3.358384
C	1.996504	-5.195893	-3.441546
H	2.957708	-5.057260	-3.948147
H	1.236893	-5.413086	-4.199835
H	2.076527	-6.081959	-2.802911
C	0.302134	-4.211644	-1.847628
H	0.454472	-5.020156	-1.122426
H	-0.513430	-4.505063	-2.518774
H	0.000875	-3.313028	-1.298959
C	2.527803	-0.829607	-3.553306
C	3.689467	-1.483497	-4.209612
H	3.925636	-0.977788	-5.148144
H	3.484036	-2.537499	-4.434256
H	4.578870	-1.461930	-3.570404
C	1.662810	0.104451	-4.008315
C	1.608604	0.827022	-5.304729
H	2.493995	0.591051	-5.898910
H	1.573582	1.912598	-5.155625
H	0.722877	0.557638	-5.893098
C	-0.367579	1.238039	-3.187620
C	-0.303116	2.546226	-2.678722
C	-1.293682	3.443229	-3.085765
H	-1.284400	4.462617	-2.716910
C	-2.293440	3.055472	-3.967521
H	-3.040516	3.778485	-4.283491
C	-2.362811	1.746041	-4.421526
H	-3.167202	1.452197	-5.088013
C	-1.404210	0.809084	-4.035278
C	-1.502282	-0.642373	-4.467071
H	-0.484089	-1.041465	-4.557653
C	-2.197746	-0.841767	-5.815599
H	-3.268337	-0.617056	-5.757149
H	-2.105488	-1.887374	-6.126514
H	-1.759766	-0.212729	-6.598237
C	-2.208968	-1.449725	-3.366635
H	-1.706240	-1.320301	-2.402801
H	-2.218668	-2.516579	-3.617718
H	-3.247497	-1.115307	-3.256187
C	0.811245	2.978335	-1.737810
H	0.939100	2.178815	-0.992583
C	2.154059	3.155940	-2.467057
H	2.515916	2.224624	-2.909630
H	2.913988	3.502834	-1.757796
H	2.062069	3.909417	-3.258435
C	0.479185	4.264595	-0.979408
H	0.466421	5.132581	-1.649036
H	1.245899	4.450143	-0.224668
H	-0.488290	4.207949	-0.472576
C	-3.160255	-3.585418	-0.491473
H	-4.045757	-3.752206	-1.118225
H	-2.907985	-4.533249	-0.007217
H	-2.331273	-3.309152	-1.146741
C	-4.786985	-2.994540	1.992446
H	-5.625650	-3.423251	1.429140
H	-5.196443	-2.252321	2.685061
H	-4.340499	-3.799266	2.586716
C	-4.409508	-0.833608	-0.130993
H	-3.744594	-0.380402	-0.872556
H	-4.726910	-0.042675	0.555292
H	-5.299036	-1.218249	-0.646926
C	-1.278541	0.827682	4.711564
H	-0.666756	1.436044	4.038937
H	-0.597225	0.234392	5.329512
H	-1.827235	1.500779	5.383199
C	-3.214270	-1.469636	5.102056
H	-3.851133	-0.909915	5.798998
H	-2.411925	-1.932689	5.684026
H	-3.819323	-2.269572	4.665177
C	-3.961561	0.820538	3.259407
H	-4.307744	1.409284	4.118636
H	-4.809098	0.224146	2.905884
H	-3.690538	1.519322	2.462563
C	-0.127046	-3.382192	4.541847
H	0.649089	-4.106057	4.821212
H	-1.071998	-3.721572	4.978921
H	0.131378	-2.421860	4.997027
C	1.460831	-3.122226	1.901778
H	1.424661	-3.319890	0.825735

H	2.161704	-3.835395	2.353303
H	1.857176	-2.108936	2.017416
C	-0.955725	-4.962871	2.103692
H	-0.945878	-5.058387	1.013775
H	-1.982417	-5.119852	2.451556
H	-0.336133	-5.769453	2.516517
C	-2.949209	3.453738	0.459545
C	-3.994529	3.744065	-0.444434
C	-4.604756	4.990963	-0.347874
H	-5.406620	5.239943	-1.038078
C	-4.218652	5.903195	0.629143
H	-4.708892	6.870580	0.687974
C	-3.219771	5.578445	1.540589
H	-2.935537	6.289696	2.311425
C	-2.565591	4.350971	1.481373
C	-1.510569	3.975052	2.472733
H	-0.642605	3.498341	2.003200
H	-1.903237	3.243651	3.190012
H	-1.174837	4.848973	3.036556
C	-4.470613	2.712243	-1.418920
H	-3.644158	2.150075	-1.859186
H	-5.048281	3.173305	-2.224202
H	-5.117264	1.986462	-0.910427
C	2.677622	4.101434	2.089080
H	1.607023	4.332005	2.070364
H	2.920254	3.662329	1.113443
H	3.239992	5.033123	2.196516
C	-1.721132	1.231542	0.172072

Calculated energies and coordinates of **INT3**

Electronic energy ... -3905.79281067 Eh
Total Enthalpy ... -3904.43280656 Eh
Final Gibbs free energy ... -3904.61697896 Eh

CARTESIAN COORDINATES (ANGSTROEM)

Si	-0.383579	-0.122426	0.561758
Si	-1.772079	-1.620536	1.755761
Si	-3.586137	-2.331057	0.399798
Si	-2.810956	-0.396541	3.518113
Si	-0.441001	-3.397105	2.608495
N	2.120143	-1.100462	-2.236635
N	0.157789	-0.563255	-0.947262
N	1.239802	1.456366	2.279344
N	0.651759	0.228200	-3.161185
N	-1.267835	2.485540	1.339086
C	4.693818	0.730594	-1.335499
H	5.690952	0.275067	-1.361080
H	4.817026	1.801172	-1.136209
H	4.237685	0.629138	-2.323556
C	3.836920	0.091309	-0.229672
H	2.823505	0.508405	-0.298816
C	3.712348	-1.413354	-0.408568
C	2.837418	-1.969437	-1.356975
C	0.907861	-0.463026	-1.980588
C	1.059807	0.400935	1.675734
C	2.151410	1.962283	3.198458
C	2.637211	3.266673	2.968512
C	3.467438	3.846166	3.920701
H	3.852423	4.847886	3.746150
C	3.798620	3.164837	5.088925
H	4.432812	3.635955	5.833931
C	2.505302	1.242093	4.358278
C	2.071939	-0.178626	4.572645
H	2.770198	-0.875948	4.092251
H	1.088975	-0.377586	4.138854
H	2.046102	-0.418917	5.639958
C	3.317424	1.878239	5.297685
H	3.588147	1.340037	6.203240
C	4.397229	0.481777	1.138264
H	3.844198	-0.001591	1.948717
H	4.314026	1.563418	1.280007
H	5.459105	0.224863	1.229204
C	4.484621	-2.297309	0.346712
H	5.165133	-1.910159	1.096888
C	4.398649	-3.670052	0.155660
H	5.013803	-4.336672	0.753765
C	3.523095	-4.195082	-0.782812
H	3.455249	-5.271153	-0.909249
C	2.722822	-3.354144	-1.557289
C	1.708354	-3.930113	-2.526856
H	1.479576	-3.167348	-3.281321
C	2.207894	-5.176554	-3.264096
H	3.185315	-5.009487	-3.729142
H	1.496715	-5.453291	-4.049399
H	2.296877	-6.037003	-2.592483
C	0.404860	-4.230577	-1.772534
H	0.577964	-4.991756	-1.002418
H	-0.361486	-4.607192	-2.459506
H	0.025797	-3.328265	-1.280849
C	2.548110	-0.856765	-3.552276
C	3.779298	-1.479156	-4.104081
H	4.030754	-1.024505	-5.064675
H	3.652313	-2.557193	-4.262763
H	4.631979	-1.354498	-3.428184
C	1.641290	-0.031939	-4.124172
C	1.590051	0.567076	-5.482727
H	2.520024	0.361446	-6.016918
H	1.452737	1.653197	-5.434314
H	0.761108	0.166472	-6.079002
C	-0.490036	1.038395	-3.444694
C	-0.502553	2.378567	-3.019820
C	-1.571625	3.171361	-3.440718
H	-1.625447	4.209943	-3.133863
C	-2.588046	2.648095	-4.229646
H	-3.411446	3.286078	-4.538780
C	-2.564299	1.316215	-4.615293
H	-3.370794	0.921632	-5.224912
C	-1.507739	0.485333	-4.238777
C	-1.481332	-0.978633	-4.641290
H	-0.433065	-1.295290	-4.704415
C	-2.124499	-1.248016	-6.004709
H	-3.211586	-1.117554	-5.973991
H	-1.933823	-2.283687	-6.304510
H	-1.725487	-0.586987	-6.781726
C	-2.141476	-1.834077	-3.551313
H	-1.660502	-1.670332	-2.582158
H	-2.071378	-2.898412	-3.803905
H	-3.201726	-1.576307	-3.449811
C	0.603166	2.945492	-2.139244
H	0.789609	2.208134	-1.344069
C	1.917158	3.151918	-2.912605
H	2.334033	2.215404	-3.290167
H	2.665792	3.605115	-2.252957
H	1.758375	3.831517	-3.758380
C	0.215185	4.262383	-1.463692
H	0.126049	5.075090	-2.194166
H	0.996224	4.550716	-0.755296
H	-0.724524	4.188159	-0.909656
C	-3.179289	-3.771205	-0.753322
H	-4.001031	-3.903691	-1.468939
H	-3.070799	-4.703832	-0.190628
H	-2.261914	-3.610006	-1.321909
C	-5.018696	-2.939514	1.479427
H	-5.833606	-3.291554	0.833972
H	-5.421479	-2.154620	2.126629
H	-4.714576	-3.778484	2.115135
C	-4.209466	-0.870454	-0.623030
H	-3.398597	-0.374063	-1.166386
H	-4.676175	-0.123044	0.026024
H	-4.962199	-1.205460	-1.347989
C	-1.600184	0.650621	4.516412
H	-0.978574	1.282284	3.875309
H	-0.941327	0.028351	5.130569
H	-2.163217	1.304891	5.194286
C	-3.646882	-1.583624	4.735330
H	-4.321673	-1.013005	5.386144
H	-2.909677	-2.077701	5.374897
H	-4.238557	-2.358232	4.238825
C	-4.153943	0.756435	2.853223
H	-4.578838	1.330394	3.686636

H	-4.972216	0.205924	2.378253
H	-3.753787	1.469173	2.126869
C	-0.431595	-3.425387	4.500246
H	0.321048	-4.144731	4.847265
H	-1.400955	-3.737228	4.901591
H	-0.183412	-2.449632	4.930758
C	1.334562	-3.217604	2.013121
H	1.396897	-3.254829	0.921233
H	1.958474	-4.026415	2.414554
H	1.765159	-2.258327	2.318282
C	-1.098232	-5.075930	2.036314
H	-1.037426	-5.176876	0.948249
H	-2.142355	-5.227182	2.331897
H	-0.502240	-5.881046	2.484491
C	-2.069804	3.635160	1.463552
C	-3.103029	3.924243	0.535653
C	-3.791755	5.128278	0.666578
H	-4.576410	5.361382	-0.048968
C	-3.504540	6.018696	1.694315
H	-4.055555	6.951249	1.776659
C	-2.518604	5.706589	2.617272
H	-2.300288	6.390787	3.433813
C	-1.788922	4.520618	2.525082
C	-0.766759	4.218423	3.583994
H	-0.128278	3.381968	3.306301
H	-1.266870	3.957664	4.524561
H	-0.140669	5.095073	3.780935
C	-3.487798	2.976536	-0.556681
H	-2.621985	2.595527	-1.106400
H	-4.168169	3.463484	-1.261930
H	-3.989444	2.091141	-0.151115
C	2.277241	3.975256	1.697707
H	1.192363	3.964611	1.540945
H	2.714929	3.468964	0.826456
H	2.633294	5.009105	1.708015
C	-1.360674	1.468126	0.650878

Calculated energies and coordinates of **TS3**

Electronic energy ... -3905.77760356 Eh
Total Enthalpy ... -3904.41849697 Eh
Final Gibbs free energy ... -3904.60165408 Eh

CARTESIAN COORDINATES (ANGSTROEM)

Si	-0.239614	-0.049296	0.587207
Si	-1.691079	-1.464848	1.834693
Si	-3.569841	-2.454981	0.757481
Si	-2.540124	-0.161303	3.643824
Si	-0.343241	-3.273163	2.644883
N	2.135231	-1.045458	-2.336113
N	0.266546	-0.500494	-0.935970
N	1.867545	0.429214	2.686004
N	0.727531	0.438381	-3.107470
N	-1.397020	2.651782	1.257189
C	4.763055	0.598239	-1.424623
H	5.730128	0.100960	-1.565163
H	4.958202	1.642856	-1.157644
H	4.226909	0.594737	-2.376582
C	3.967950	-0.086455	-0.299704
H	2.971651	0.376245	-0.253220
C	3.751091	-1.567114	-0.574923
C	2.815797	-2.008091	-1.527875
C	0.978426	-0.354876	-1.992443
C	1.008600	0.306110	1.825521
C	2.209889	1.280791	3.731469
C	2.296496	2.672901	3.526498
C	2.753386	3.462117	4.580767
H	2.824625	4.537486	4.435377
C	3.103491	2.903731	5.804272
H	3.445996	3.539292	6.615233
C	2.609502	0.693043	4.948638
C	2.579788	-0.798034	5.105047
H	3.279850	-1.275065	4.407402
H	1.592303	-1.211371	4.876932
H	2.850293	-1.090872	6.122764
C	3.029497	1.524246	5.980196
H	3.315418	1.081055	6.930842
C	4.662886	0.180746	1.037427
H	4.172539	-0.340755	1.863112
H	4.630429	1.252309	1.260181
H	5.719143	-0.111997	1.003036
C	4.474879	-2.543636	0.110520
H	5.201297	-2.248437	0.859247
C	4.274239	-3.895046	-0.139240
H	4.850741	-4.634926	0.409047
C	3.329780	-4.305220	-1.067782
H	3.169437	-5.365501	-1.236918
C	2.581494	-3.368077	-1.782026
C	1.495605	-3.813903	-2.742134
H	1.302034	-2.997111	-3.448240
C	1.875176	-5.052826	-3.558756
H	2.843225	-4.931170	-4.056468
H	1.116283	-5.238967	-4.325928
H	1.927945	-5.950487	-2.933725
C	0.195952	-4.053558	-1.959463
H	0.325081	-4.883819	-1.254947
H	-0.623809	-4.310297	-2.640157
H	-0.085810	-3.162137	-1.389279
C	2.522794	-0.750341	-3.653112
C	3.686549	-1.416302	-4.292004
H	3.896865	-0.957374	-5.260228
H	3.501962	-2.485083	-4.455193
H	4.584853	-1.338550	-3.669464
C	1.652766	0.168881	-4.131283
C	1.568548	0.834186	-5.456681
H	2.459405	0.608237	-6.046522
H	1.488956	1.922244	-5.354494
H	0.691484	0.501164	-6.025174
C	-0.332082	1.384359	-3.260288
C	-0.179433	2.678606	-2.729332
C	-1.185975	3.606167	-3.002282
H	-1.124854	4.608910	-2.594516
C	-2.284476	3.264294	-3.780475
H	-3.052538	4.006799	-3.977694
C	-2.420511	1.977591	-4.278993
H	-3.297372	1.720163	-4.864970
C	-1.448977	1.008672	-4.022380
C	-1.642006	-0.425791	-4.476782
H	-0.655998	-0.900239	-4.550674
C	-2.321851	-0.548393	-5.843621
H	-3.373415	-0.245416	-5.802296
H	-2.300192	-1.591272	-6.176334
H	-1.823141	0.064409	-6.602172
C	-2.433511	-1.188813	-3.402842
H	-1.918120	-1.145906	-2.437256
H	-2.560389	-2.239273	-3.689465
H	-3.428793	-0.745402	-3.279939
C	1.047113	3.065227	-1.916121
H	1.257151	2.235545	-1.224879
C	2.282745	3.262163	-2.812139
H	2.581295	2.342505	-3.320315
H	3.131912	3.597173	-2.206289
H	2.082843	4.030648	-3.568241
C	0.838535	4.327084	-1.076628
H	0.767447	5.219343	-1.709428
H	1.697355	4.469463	-0.414435
H	-0.063286	4.266883	-0.462490
C	-3.129193	-3.726614	-0.566724
H	-4.058758	-4.126277	-0.992567
H	-2.547927	-4.565285	-0.177562
H	-2.560320	-3.268735	-1.379982
C	-4.542487	-3.328179	2.131434
H	-5.263615	-4.024906	1.685818
H	-5.107245	-2.600694	2.724538
H	-3.905863	-3.895137	2.817916
C	-4.791312	-1.282656	-0.080695
H	-4.374194	-0.855125	-0.996687
H	-5.121112	-0.459653	0.559476
H	-5.677736	-1.867486	-0.359825
C	-1.202161	0.928138	4.401386
H	-0.798600	1.636300	3.671374

H	-0.367245	0.334676	4.789300
H	-1.623004	1.495569	5.241419
C	-3.243713	-1.228712	5.043252
H	-3.807648	-0.575986	5.722115
H	-2.454914	-1.709798	5.627424
H	-3.925532	-2.007683	4.689952
C	-3.970978	0.937382	3.075229
H	-4.280049	1.572718	3.915248
H	-4.840393	0.341430	2.776910
H	-3.704753	1.598523	2.246307
C	-0.437604	-3.389256	4.531105
H	0.310011	-4.107813	4.890316
H	-1.424644	-3.743099	4.848039
H	-0.252992	-2.432984	5.028637
C	1.454319	-3.107729	2.108514
H	1.533334	-3.092786	1.016135
H	2.021064	-3.975101	2.472107
H	1.935592	-2.200877	2.481973
C	-0.907217	-4.963584	2.005374
H	-0.699002	-5.069788	0.936649
H	-1.970759	-5.160984	2.170205
H	-0.337342	-5.738914	2.533575
C	-2.308304	3.573496	0.701726
C	-3.267859	3.203602	-0.265335
C	-4.170261	4.172904	-0.702560
H	-4.916938	3.893741	-1.442283
C	-4.123351	5.474409	-0.219546
H	-4.833095	6.214410	-0.578270
C	-3.179789	5.821497	0.741131
H	-3.152006	6.834407	1.134824
C	-2.275434	4.881457	1.228365
C	-1.276155	5.262566	2.282916
H	-1.230026	4.507015	3.073770
H	-1.534125	6.229575	2.723195
H	-0.265392	5.338856	1.865168
C	-3.341773	1.813778	-0.813182
H	-2.488988	1.591429	-1.461791
H	-4.261808	1.680731	-1.390574
H	-3.319049	1.067598	-0.012275
C	1.917831	3.282943	2.213004
H	0.836859	3.232265	2.041620
H	2.393167	2.755617	1.375889
H	2.222312	4.333057	2.177291
C	-0.962304	1.586241	0.808648

Calculated energies and coordinates of **INT4**

Electronic energy ... -3905.80688088 Eh
Total Enthalpy ... -3904.44550366 Eh
Final Gibbs free energy ... -3904.62823902 Eh

CARTESIAN COORDINATES (ANGSTROEM)

Si	-0.312006	-0.207966	0.579730
Si	-1.816888	-1.603860	1.781926
Si	-3.669515	-2.367451	0.489593
Si	-2.748243	-0.450753	3.660863
Si	-0.406390	-3.372015	2.540809
N	2.086779	-1.013958	-2.285395
N	0.096418	-0.539277	-0.996437
N	1.816835	0.720234	2.492475
N	0.615645	0.346250	-3.158638
N	-0.889447	2.517529	2.086771
C	4.573795	0.746917	-1.327882
H	5.557319	0.309253	-1.536691
H	4.730406	1.788255	-1.024217
H	3.993004	0.758342	-2.253207
C	3.876385	-0.020079	-0.190508
H	2.866520	0.396200	-0.063769
C	3.702624	-1.500686	-0.502369
C	2.783870	-1.958888	-1.465212
C	0.874149	-0.394508	-2.013688
C	0.678337	0.542825	1.942638
C	2.142049	1.725358	3.416952
C	2.456307	3.028432	2.988356
C	2.893940	3.953281	3.936220
H	3.133509	4.963291	3.609842
C	3.029812	3.607917	5.275292
H	3.363743	4.344686	5.999538
C	2.342730	1.342330	4.757336
C	2.154745	-0.092956	5.156087
H	2.917039	-0.723852	4.680394
H	1.186470	-0.481228	4.829072
H	2.232754	-0.213292	6.240146
C	2.762070	2.302553	5.674068
H	2.895357	2.013441	6.714040
C	4.641656	0.227483	1.111752
H	4.202914	-0.318131	1.950040
H	4.602621	1.290873	1.366585
H	5.699140	-0.045252	1.007262
C	4.450845	-2.467173	0.172330
H	5.164592	-2.161491	0.927938
C	4.293776	-3.820529	-0.094643
H	4.890089	-4.548582	0.448310
C	3.371228	-4.246824	-1.036482
H	3.246274	-5.308725	-1.224139
C	2.602529	-3.322842	-1.745186
C	1.554463	-3.798695	-2.732597
H	1.326832	-2.977012	-3.422598
C	2.008761	-4.995528	-3.574498
H	2.977906	-4.814624	-4.051327
H	1.272405	-5.196840	-4.359522
H	2.094957	-5.906019	-2.972337
C	0.262915	-4.130598	-1.972137
H	0.435755	-4.959330	-1.275596
H	-0.530032	-4.428509	-2.667397
H	-0.079088	-3.264399	-1.396149
C	2.501133	-0.733864	-3.600358
C	3.728917	-1.337376	-4.177428
H	3.943854	-0.888510	-5.149345
H	3.621557	-2.419628	-4.318254
H	4.595419	-1.183529	-3.525264
C	1.590660	0.107992	-4.140536
C	1.501681	0.711315	-5.495510
H	2.438169	0.559447	-6.036321
H	1.304351	1.787162	-5.443202
H	0.692844	0.263930	-6.086620
C	-0.519259	1.190518	-3.364661
C	-0.476888	2.520725	-2.906121
C	-1.583541	3.325530	-3.182291
H	-1.604366	4.351313	-2.833053
C	-2.680816	2.825696	-3.872276
H	-3.533240	3.471335	-4.065659
C	-2.697544	1.510880	-4.312181
H	-3.562661	1.136731	-4.850797
C	-1.609097	0.668413	-4.078050
C	-1.626820	-0.773671	-4.552398
H	-0.588873	-1.118273	-4.630675
C	-2.273312	-0.943475	-5.931247
H	-3.354287	-0.771829	-5.895617
H	-2.121041	-1.967513	-6.287871
H	-1.846059	-0.254847	-6.667952
C	-2.319272	-1.664298	-3.512475
H	-1.845253	-1.553835	-2.532334
H	-2.268535	-2.717372	-3.812051
H	-3.376123	-1.390756	-3.415574
C	0.716026	3.062185	-2.127451
H	0.968545	2.309709	-1.364662
C	1.955160	3.264418	-3.017645
H	2.331672	2.329847	-3.437752
H	2.763348	3.706775	-2.424921
H	1.724735	3.951994	-3.840132
C	0.417293	4.379809	-1.408575
H	0.251662	5.192972	-2.125066
H	1.278854	4.662475	-0.798044
H	-0.454919	4.313605	-0.752170
C	-3.220429	-3.773653	-0.688887
H	-4.073291	-3.967801	-1.352208
H	-3.003247	-4.696763	-0.143460
H	-2.356794	-3.539398	-1.313663
C	-5.020784	-3.052292	1.623332
H	-5.810015	-3.494003	1.001378

H	-5.479764	-2.271155	2.237599
H	-4.645726	-3.836050	2.290190
C	-4.468910	-0.995693	-0.534995
H	-3.755048	-0.504113	-1.201865
H	-4.923542	-0.225371	0.095231
H	-5.263431	-1.435648	-1.151733
C	-1.520428	0.418881	4.798585
H	-0.830838	1.088381	4.277947
H	-0.942181	-0.303212	5.382906
H	-2.095311	1.025575	5.510805
C	-3.643847	-1.703851	4.765450
H	-4.238424	-1.151046	5.504246
H	-2.929466	-2.322472	5.316313
H	-4.320441	-2.368042	4.222880
C	-4.031853	0.826226	3.114327
H	-4.582908	1.164429	4.001222
H	-4.762077	0.419563	2.406966
H	-3.569353	1.708691	2.662080
C	-0.424475	-3.528393	4.424668
H	0.360855	-4.229812	4.733833
H	-1.380508	-3.916755	4.789691
H	-0.230758	-2.572652	4.921625
C	1.360576	-3.019251	1.995555
H	1.441767	-2.980704	0.903430
H	2.030780	-3.812780	2.349696
H	1.725395	-2.062519	2.385921
C	-0.921782	-5.054714	1.850924
H	-0.856012	-5.079100	0.759315
H	-1.946402	-5.317334	2.136346
H	-0.254360	-5.830741	2.246910
C	-1.705383	3.493911	1.515667
C	-2.594792	3.312623	0.426070
C	-3.317294	4.414031	-0.032169
H	-4.002813	4.274029	-0.864704
C	-3.179447	5.668252	0.548513
H	-3.748509	6.510928	0.165936
C	-2.334299	5.833224	1.639405
H	-2.244579	6.806254	2.115303
C	-1.608958	4.760421	2.148848
C	-0.728681	4.945303	3.350707
H	-0.953756	4.200561	4.120401
H	-0.860112	5.946557	3.770345
H	0.329186	4.806091	3.108626
C	-2.818440	1.985840	-0.229686
H	-1.963777	1.677030	-0.841611
H	-3.693465	2.033877	-0.883509
H	-2.976741	1.198078	0.511018
C	2.332185	3.413732	1.542515
H	1.282528	3.515284	1.247246
H	2.771742	2.654007	0.885221
H	2.833072	4.367864	1.352615
C	-0.505679	1.409506	1.588897

Calculated energies and coordinates of **TS4**

Electronic energy ... -3905.79002135 Eh
Total Enthalpy ... -3904.43017976 Eh
Final Gibbs free energy ... -3904.61088963 Eh

CARTESIAN COORDINATES (ANGSTROEM)

Si	-0.621518	-0.362791	0.289606
Si	-2.016088	-2.220865	0.561263
Si	-3.463184	-2.220051	-1.323009
Si	-3.363207	-2.198652	2.515592
Si	-0.621241	-4.129033	0.432144
N	3.104281	-0.074718	0.207438
N	0.867468	-0.711135	-0.334563
N	0.041742	1.791320	1.311192
N	2.225888	0.883897	-1.561299
N	-3.563093	2.036223	0.528539
C	3.278104	0.984491	3.969837
H	2.702759	1.837359	4.344432
H	4.141385	1.370429	3.416571
H	3.655672	0.414084	4.826758
C	2.389370	0.113560	3.071898
H	2.005291	0.732075	2.255985
C	3.169534	-1.037079	2.479965
C	3.447019	-1.144696	1.110691
C	1.948590	0.006927	-0.533613
C	-1.137858	1.335098	0.732338
C	0.050642	2.606290	2.428005
C	1.181350	3.466380	2.580521
C	1.281246	4.308921	3.681830
H	2.147050	4.964630	3.758299
C	0.310587	4.328210	4.676862
H	0.402264	4.992115	5.530940
C	-0.905089	2.584025	3.489002
C	-2.015910	1.573145	3.543752
H	-2.891586	1.874613	2.962283
H	-2.337784	1.426761	4.580303
H	-1.691042	0.605998	3.145892
C	-0.759620	3.449204	4.572118
H	-1.499446	3.405691	5.369483
C	1.167060	-0.394390	3.845069
H	0.534390	-1.017763	3.202485
H	0.576034	0.458161	4.194304
H	1.451070	-0.992718	4.718571
C	3.641990	-2.049811	3.319293
H	3.443223	-1.986361	4.384921
C	4.361250	-3.123483	2.820792
H	4.724604	-3.894905	3.493953
C	4.606427	-3.219123	1.458412
H	5.155099	-4.072771	1.070391
C	4.153791	-2.239174	0.576140
C	4.408247	-2.416064	-0.910046
H	3.978764	-1.562264	-1.441630
C	5.904687	-2.452672	-1.246369
H	6.398786	-3.309100	-0.774763
H	6.414602	-1.543758	-0.913051
H	6.043834	-2.541539	-2.329558
C	3.705969	-3.681916	-1.419626
H	2.637404	-3.652615	-1.192240
H	4.123792	-4.581642	-0.954621
H	3.828894	-3.780845	-2.504183
C	4.084288	0.762127	-0.344130
C	5.391089	0.988758	0.322252
H	5.274487	1.661559	1.181541
H	6.096663	1.450343	-0.372560
H	5.823992	0.055995	0.695731
C	3.550407	1.346863	-1.441692
C	4.164951	2.317602	-2.383909
H	3.697019	2.253932	-3.370769
H	5.231426	2.104880	-2.498924
H	4.064536	3.350797	-2.033470
C	1.348454	1.157495	-2.669976
C	0.788613	2.437595	-2.826104
C	-0.050499	2.650414	-3.923951
H	-0.500177	3.629707	-4.060388
C	-0.326820	1.638891	-4.826048
H	-0.996715	1.822087	-5.661550
C	0.266227	0.392083	-4.674235
H	0.059915	-0.383780	-5.403253
C	1.130256	0.129080	-3.612972
C	1.890422	-1.185533	-3.553821
H	1.945078	-1.503109	-2.506620
C	3.321452	-0.976628	-4.081964
H	3.874960	-1.921956	-4.064898
H	3.878788	-0.249291	-3.485063
H	3.290949	-0.618212	-5.117244
C	1.226796	-2.323665	-4.331848
H	1.734479	-3.266029	-4.102134
H	1.297520	-2.171251	-5.414790
H	0.172280	-2.436419	-4.066734
C	1.079052	3.605503	-1.903815
H	1.773565	3.261296	-1.134052
C	1.723898	4.769840	-2.678623
H	2.546543	4.446793	-3.322409
H	2.105657	5.519315	-1.976197
H	0.984826	5.267963	-3.315693
C	-0.179123	4.114114	-1.189320
H	0.083207	4.933149	-0.510619

H	-0.649855	3.326002	-0.600700
H	-0.910768	4.501777	-1.908267
C	-2.530291	-1.753535	-2.900470
H	-1.954136	-2.616637	-3.250282
H	-1.832505	-0.919252	-2.774245
H	-3.238024	-1.479841	-3.692816
C	-4.138617	-3.970281	-1.571689
H	-3.351101	-4.685364	-1.829886
H	-4.866469	-3.964538	-2.393215
H	-4.649937	-4.341050	-0.677455
C	-4.945259	-1.089481	-1.030554
H	-4.682965	-0.120602	-0.596225
H	-5.651718	-1.573539	-0.346586
H	-5.471146	-0.903156	-1.975328
C	-4.475016	-3.727705	2.433388
H	-5.183969	-3.659737	1.601308
H	-5.061572	-3.791632	3.358929
H	-3.913775	-4.660056	2.325894
C	-4.495067	-0.693766	2.619571
H	-4.094044	0.188672	2.114940
H	-4.675777	-0.432928	3.669512
H	-5.461936	-0.923911	2.158881
C	-2.286988	-2.359015	4.057778
H	-2.925789	-2.499788	4.938832
H	-1.670557	-1.471911	4.230753
H	-1.618868	-3.224717	3.986804
C	0.884524	-3.944632	1.543341
H	1.521531	-4.835757	1.477291
H	0.596745	-3.816543	2.592724
H	1.485145	-3.077067	1.254414
C	-0.073071	-4.340004	-1.361509
H	-0.921161	-4.569602	-2.014921
H	0.653544	-5.156504	-1.455954
H	0.392901	-3.418641	-1.725807
C	-1.562833	-5.679700	0.963365
H	-2.510948	-5.799620	0.429316
H	-1.776032	-5.665138	2.037595
H	-0.948083	-6.565865	0.761023
C	-4.387982	2.741375	-0.367418
C	-4.266115	2.637697	-1.766704
C	-5.210660	3.288166	-2.561963
H	-5.132355	3.202839	-3.643505
C	-6.234889	4.038661	-1.999186
H	-6.955919	4.541641	-2.636961
C	-6.340217	4.133534	-0.615057
H	-7.142692	4.716075	-0.169338
C	-5.437102	3.478651	0.216521
C	-5.556285	3.556657	1.711352
H	-6.446448	4.121741	2.000557
H	-4.679022	4.039374	2.156869
H	-5.614401	2.553379	2.149105
C	-3.165735	1.843712	-2.404583
H	-3.017908	0.883745	-1.900862
H	-2.205874	2.365702	-2.356570
H	-3.389611	1.646315	-3.457516
C	2.272879	3.508497	1.546311
H	2.041855	4.236856	0.758645
H	2.407165	2.541752	1.056468
H	3.219336	3.815746	2.004973
C	-2.336095	1.852963	0.470073

Calculated energies and coordinates of **3a**

Electronic energy ... -3905.83535264 Eh
Total Enthalpy ... -3904.47353071 Eh
Final Gibbs free energy ... -3904.65626673 Eh

CARTESIAN COORDINATES (ANGSTROEM)

Si	4.249325	3.373232	2.385049
Si	2.991497	1.809095	3.614758
Si	1.219441	0.609697	2.592772
Si	2.056613	2.985080	5.445751
Si	4.551893	0.242992	4.454876
N	6.835959	2.347611	-0.313285
N	4.851274	2.881139	0.918948
N	7.296596	6.007270	3.564979
N	5.578352	3.929016	-1.138593
N	3.774735	5.012359	2.827557
C	9.350018	3.747455	1.034329
H	10.308800	3.229543	0.911577
H	9.552182	4.749363	1.427300
H	8.888333	3.867501	0.052439
C	8.454295	2.988005	2.029351
H	7.488538	3.511479	2.072816
C	8.176894	1.558075	1.581630
C	7.362448	1.272727	0.470130
C	5.681345	3.070593	-0.055460
C	6.193231	5.431729	3.554747
C	8.093594	6.427106	4.644931
C	9.096716	7.366970	4.318912
C	9.971641	7.798274	5.311783
H	10.737749	8.527113	5.059764
C	9.882236	7.302836	6.606910
H	10.577017	7.639609	7.370733
C	7.997012	5.916947	5.957987
C	6.951333	4.916999	6.344282
H	6.886868	4.093648	5.625780
H	5.958137	5.377191	6.375025
H	7.162628	4.504147	7.334841
C	8.905287	6.366326	6.916642
H	8.836938	5.969488	7.926773
C	9.100260	3.080736	3.414151
H	8.527387	2.556366	4.184204
H	9.171961	4.131346	3.706804
H	10.118631	2.674225	3.403755
C	8.736241	0.469363	2.254267
H	9.359443	0.639396	3.124562
C	8.510825	-0.834033	1.831396
H	8.961896	-1.661037	2.372817
C	7.713125	-1.086138	0.726004
H	7.541477	-2.110715	0.411626
C	7.122443	-0.036538	0.021576
C	6.202424	-0.316608	-1.153313
H	6.186715	0.569300	-1.799007
C	6.656930	-1.502015	-2.010686
H	7.707830	-1.412661	-2.306126
H	6.048269	-1.556033	-2.919293
H	6.534463	-2.454801	-1.484801
C	4.768845	-0.528853	-0.648460
H	4.715784	-1.423276	-0.016770
H	4.079194	-0.665545	-1.489423
H	4.430293	0.327342	-0.056993
C	7.384686	2.708258	-1.554772
C	8.607570	2.058460	-2.092478
H	8.979784	2.619076	-2.952470
H	8.417338	1.028000	-2.416236
H	9.399644	2.017811	-1.337469
C	6.602582	3.685341	-2.067969
C	6.683099	4.423213	-3.354174
H	7.631506	4.208459	-3.851229
H	6.608837	5.505124	-3.202139
H	5.871732	4.137978	-4.035174
C	4.556299	4.903429	-1.371471
C	4.677579	6.179323	-0.790014
C	3.698095	7.119547	-1.113627
H	3.731660	8.106734	-0.668021
C	2.661090	6.809286	-1.983253
H	1.911162	7.561137	-2.213018
C	2.569456	5.546544	-2.548108
H	1.747982	5.318214	-3.220088
C	3.518017	4.566548	-2.254278
C	3.373668	3.161325	-2.807993
H	4.363403	2.688048	-2.810454
C	2.842059	3.124270	-4.243907
H	1.791127	3.427627	-4.296589
H	2.902126	2.103395	-4.635282
H	3.416472	3.780749	-4.906191
C	2.479165	2.335478	-1.871526
H	2.897714	2.317360	-0.860525
H	2.387733	1.304807	-2.232848
H	1.474391	2.771512	-1.817441

C	5.821946	6.528879	0.149452
H	5.921665	5.704555	0.869187
C	7.164106	6.671535	-0.589478
H	7.498417	5.738560	-1.048404
H	7.934961	6.981320	0.123837
H	7.092194	7.441392	-1.367305
C	5.572927	7.808790	0.949433
H	5.662381	8.698362	0.313603
H	6.327111	7.884169	1.737188
H	4.590051	7.819630	1.426144
C	1.614953	-0.135755	0.909797
H	0.741596	-0.692468	0.546724
H	2.457246	-0.831178	0.970724
H	1.862239	0.627103	0.167609
C	0.819919	-0.839617	3.743907
H	-0.053541	-1.379897	3.357152
H	0.590258	-0.516807	4.763678
H	1.653103	-1.548733	3.793928
C	-0.347836	1.651224	2.422405
H	-0.211224	2.497907	1.742224
H	-0.680987	2.046193	3.387564
H	-1.155888	1.026885	2.020552
C	3.479321	3.612650	6.513458
H	4.227832	4.096397	5.877292
H	3.974196	2.791873	7.044804
H	3.132206	4.339717	7.257695
C	0.973131	1.826697	6.480877
H	0.672251	2.344264	7.400702
H	1.488253	0.905189	6.768372
H	0.057281	1.551550	5.946383
C	0.965363	4.437343	4.942218
H	0.437282	4.812041	5.828566
H	0.209975	4.151391	4.202967
H	1.542668	5.263399	4.517939
C	3.948168	-0.598968	6.036413
H	4.691259	-1.338467	6.360759
H	2.996227	-1.120923	5.897876
H	3.822887	0.123025	6.850232
C	6.154365	1.153683	4.841589
H	6.937967	0.453851	5.157901
H	6.015332	1.900296	5.630242
H	6.508311	1.670192	3.943393
C	4.926640	-1.071128	3.156105
H	5.328558	-0.600300	2.253124
H	4.035327	-1.642326	2.875150
H	5.680750	-1.776313	3.527152
C	3.005823	6.157566	2.672624
C	1.965672	6.122665	1.712323
C	1.183998	7.253791	1.513163
H	0.401668	7.218040	0.758430
C	1.386764	8.413912	2.253654
H	0.773988	9.293437	2.082208
C	2.350306	8.409851	3.253761
H	2.475866	9.285770	3.886236
C	3.148882	7.293917	3.503975
C	4.071446	7.305230	4.689395
H	3.981723	6.377531	5.263644
H	3.818563	8.140204	5.348593
H	5.126978	7.409755	4.410999
C	1.700856	4.868060	0.938196
H	2.561435	4.580168	0.324984
H	0.840400	4.993228	0.275336
H	1.490225	4.028263	1.614458
C	9.216581	7.904688	2.921744
H	8.364796	8.545694	2.667323
H	9.225451	7.093796	2.187298
H	10.130353	8.495374	2.813462
C	5.120840	4.765381	3.258692

Calculated energies and coordinates of **TS5**

Electronic energy ... -3905.79776267 Eh
Total Enthalpy ... -3904.43943186 Eh
Final Gibbs free energy ... -3904.62365399 Eh

CARTESIAN COORDINATES (ANGSTROEM)

Si	-0.757023	0.017519	0.684925
Si	-2.018696	-1.582630	1.907788
Si	-3.851369	-2.649396	0.847876
Si	-2.921698	-0.481911	3.795452
Si	-0.522661	-3.250620	2.655865
N	1.823305	-1.054989	-1.975686
N	-0.231892	-0.515190	-0.826889
N	2.499821	2.466311	1.925030
N	0.601491	0.541672	-2.836380
N	-1.600628	1.455659	1.121334
C	4.455685	0.122672	-0.568504
H	5.378369	-0.436706	-0.765729
H	4.730629	1.062943	-0.078267
H	3.984553	0.375127	-1.520968
C	3.525386	-0.673018	0.363807
H	2.584529	-0.113126	0.438924
C	3.166390	-2.048865	-0.177073
C	2.293101	-2.208892	-1.269015
C	0.647094	-0.336071	-1.754848
C	1.537546	1.661662	1.845895
C	3.295245	2.872631	2.967566
C	4.283685	3.839637	2.638025
C	5.156065	4.274572	3.626396
H	5.913365	5.013192	3.377124
C	5.070900	3.777858	4.924280
H	5.762397	4.125558	5.686036
C	3.198814	2.369488	4.293948
C	2.152222	1.368959	4.667192
H	2.232232	0.454086	4.068736
H	1.146248	1.762379	4.484856
H	2.231872	1.103124	5.724297
C	4.098370	2.836704	5.246389
H	4.032195	2.454577	6.261936
C	4.163531	-0.707321	1.755506
H	3.570625	-1.274305	2.478209
H	4.263015	0.316266	2.129701
H	5.171852	-1.137665	1.721445
C	3.693952	-3.209695	0.393087
H	4.358813	-3.133388	1.246034
C	3.385587	-4.465704	-0.112199
H	3.812220	-5.351679	0.349989
C	2.537320	-4.595846	-1.201204
H	2.304862	-5.584589	-1.583921
C	1.977340	-3.469635	-1.804844
C	1.020985	-3.611237	-2.975314
H	1.054527	-2.681865	-3.556078
C	1.384876	-4.754758	-3.926982
H	2.431896	-4.703522	-4.244578
H	0.753655	-4.705066	-4.820594
H	1.217916	-5.735354	-3.468748
C	-0.413150	-3.772441	-2.453162
H	-0.513531	-4.717486	-1.906247
H	-1.131926	-3.778084	-3.281416
H	-0.672856	-2.957160	-1.771275
C	2.442462	-0.650726	-3.170837
C	3.662839	-1.319453	-3.692424
H	3.447998	-2.340262	-4.032133
H	4.443650	-1.395123	-2.929287
H	4.064025	-0.756482	-4.537635
C	1.697779	0.348195	-3.692546
C	1.897278	1.164850	-4.917325
H	2.220708	2.184678	-4.678652
H	0.972887	1.247475	-5.499331
H	2.660739	0.708132	-5.550588
C	-0.452225	1.450549	-3.170657
C	-0.250593	2.823533	-2.962650
C	-1.237726	3.701598	-3.415175
H	-1.123394	4.768076	-3.246444
C	-2.361704	3.230976	-4.073737
H	-3.114847	3.930657	-4.425516
C	-2.536766	1.867435	-4.276576
H	-3.428842	1.516764	-4.785037
C	-1.592756	0.946469	-3.825200
C	-1.787779	-0.547468	-4.039403
H	-1.410404	-1.055409	-3.142779

C	-0.991070	-1.057001	-5.252406
H	-1.303204	-0.529615	-6.161749
H	-1.178880	-2.126639	-5.401605
H	0.086262	-0.922193	-5.126391
C	-3.256592	-0.951220	-4.202017
H	-3.884950	-0.547964	-3.402512
H	-3.338725	-2.042618	-4.173004
H	-3.664654	-0.619573	-5.163758
C	0.978350	3.369160	-2.258898
H	1.725830	2.568736	-2.201832
C	1.607745	4.551147	-3.009966
H	1.767537	4.330279	-4.070450
H	2.574431	4.801825	-2.559882
H	0.978853	5.445590	-2.947056
C	0.629213	3.780546	-0.823937
H	-0.152445	4.548821	-0.820882
H	1.514361	4.173744	-0.312814
H	0.265204	2.925303	-0.250446
C	-3.558021	-3.006348	-0.981076
H	-4.490266	-3.329734	-1.460870
H	-2.816997	-3.800860	-1.110034
H	-3.189650	-2.121225	-1.509485
C	-4.172329	-4.295370	1.728537
H	-5.041591	-4.791600	1.278647
H	-4.388992	-4.141641	2.791309
H	-3.320888	-4.979258	1.655146
C	-5.474562	-1.685030	0.999770
H	-5.456594	-0.715677	0.493445
H	-5.743919	-1.510444	2.046285
H	-6.277705	-2.279971	0.546006
C	-1.508494	0.170499	4.865098
H	-0.943910	-0.646233	5.328721
H	-1.881006	0.823283	5.664024
H	-0.815898	0.743480	4.239771
C	-3.956753	-1.693102	4.823655
H	-3.398147	-2.590148	5.106683
H	-4.851985	-2.015245	4.280360
H	-4.292211	-1.200366	5.745093
C	-4.064685	0.957094	3.358253
H	-4.594878	1.287866	4.260556
H	-4.816469	0.672785	2.615260
H	-3.507699	1.806623	2.955556
C	-1.159690	-4.204867	4.159583
H	-0.453536	-5.008185	4.405428
H	-2.139116	-4.660731	3.984558
H	-1.239578	-3.554162	5.036864
C	1.100921	-2.420929	3.134250
H	1.853688	-3.167282	3.418186
H	0.970633	-1.721093	3.966428
H	1.483058	-1.854697	2.279027
C	-0.141450	-4.470169	1.268452
H	0.286009	-3.938455	0.412728
H	-1.030845	-5.007598	0.922992
H	0.596800	-5.210544	1.601021
C	-2.211581	2.556801	0.678026
C	-3.125273	2.516061	-0.426505
C	-3.790564	3.674071	-0.796540
H	-4.500428	3.631329	-1.619371
C	-3.566709	4.881708	-0.137956
H	-4.082826	5.781427	-0.460114
C	-2.707345	4.923973	0.960088
H	-2.555959	5.861037	1.490905
C	-2.055169	3.785353	1.405212
C	-1.141638	3.821398	2.588630
H	-1.351585	2.982518	3.261710
H	-1.245985	4.762861	3.134419
H	-0.094036	3.709417	2.284562
C	-3.458892	1.198444	-1.047529
H	-2.562877	0.673581	-1.393733
H	-4.137594	1.328595	-1.893371
H	-3.940357	0.546233	-0.308746
C	4.364633	4.390454	1.245028
H	3.480121	4.995865	1.014699
H	4.393393	3.590037	0.499227
H	5.250996	5.019708	1.129705
C	0.636788	0.831286	1.537242

Calculated energies and coordinates of **INT5**

Electronic energy ... -3905.80886042 Eh
Total Enthalpy ... -3904.44941871 Eh
Final Gibbs free energy ... -3904.63228733 Eh

CARTESIAN COORDINATES (ANGSTROEM)

Si	-0.800600	0.215300	0.565970
Si	-1.960532	-1.310318	1.999633
Si	-3.861892	-2.312871	1.019329
Si	-2.813828	-0.321363	3.977600
Si	-0.436825	-3.001957	2.641018
N	1.912134	-1.157445	-2.034772
N	0.041039	-0.568704	-0.687936
N	2.480619	2.638164	1.848436
N	0.725210	0.530271	-2.774834
N	-2.077727	1.214993	0.042706
C	4.464314	0.152994	-0.694783
H	5.410056	-0.374674	-0.868241
H	4.699266	1.133108	-0.264824
H	3.977434	0.326393	-1.656586
C	3.581148	-0.631530	0.291786
H	2.621560	-0.103236	0.374485
C	3.261779	-2.040417	-0.188529
C	2.417248	-2.269461	-1.291378
C	0.795810	-0.382543	-1.709949
C	1.520588	1.815276	1.753774
C	3.402553	2.868845	2.835227
C	4.438186	3.786525	2.498934
C	5.424508	4.065047	3.434481
H	6.218635	4.760616	3.175893
C	5.407815	3.467343	4.692544
H	6.186265	3.695908	5.414397
C	3.377572	2.264775	4.124697
C	2.280392	1.328258	4.518111
H	2.243018	0.449005	3.867179
H	1.301119	1.811283	4.432196
H	2.410001	0.993970	5.550762
C	4.387628	2.581351	5.026206
H	4.371428	2.123899	6.012288
C	4.256489	-0.592517	1.665451
H	3.698833	-1.145866	2.425619
H	4.339059	0.446556	1.998013
H	5.274488	-0.998482	1.620005
C	3.795795	-3.161735	0.448723
H	4.442480	-3.032898	1.309192
C	3.516857	-4.447154	0.000938
H	3.947562	-5.301858	0.515414
C	2.691802	-4.644657	-1.094671
H	2.476376	-5.655058	-1.428216
C	2.126536	-3.558834	-1.765649
C	1.182889	-3.789215	-2.933725
H	1.146070	-2.872230	-3.533812
C	1.636213	-4.926697	-3.856100
H	2.683573	-4.815674	-4.156628
H	1.018483	-4.939371	-4.760250
H	1.526178	-5.905388	-3.377106
C	-0.238710	-4.048191	-2.415205
H	-0.258447	-4.942146	-1.781715
H	-0.930302	-4.202306	-3.250965
H	-0.593528	-3.201785	-1.819845
C	2.473498	-0.761533	-3.252996
C	3.666804	-1.435485	-3.825442
H	4.050509	-0.863536	-4.672788
H	3.432494	-2.447029	-4.178301
H	4.465044	-1.532343	-3.081589
C	1.743367	0.281643	-3.711589
C	1.889683	1.085215	-4.953558
H	2.862258	0.889659	-5.410540
H	1.814176	2.157636	-4.746772
H	1.115137	0.847577	-5.692916
C	-0.326054	1.452024	-3.087939
C	-0.320515	2.730151	-2.505938
C	-1.289639	3.638670	-2.936075

H	-1.332486	4.628010	-2.494248
C	-2.199002	3.301466	-3.928833
H	-2.939424	4.028551	-4.250877
C	-2.171518	2.040159	-4.505688
H	-2.893144	1.789494	-5.276650
C	-1.240414	1.086593	-4.093142
C	-1.267582	-0.318546	-4.668594
H	-0.253151	-0.732897	-4.619542
C	-1.715402	-0.375702	-6.131833
H	-2.781930	-0.150632	-6.239594
H	-1.559102	-1.385300	-6.525815
H	-1.156223	0.327208	-6.758824
C	-2.158541	-1.208912	-3.792753
H	-1.811682	-1.201070	-2.755438
H	-2.150872	-2.243094	-4.153891
H	-3.194385	-0.850420	-3.809931
C	0.723798	3.123466	-1.477198
H	0.847033	2.278870	-0.789257
C	2.092140	3.393658	-2.125980
H	2.520697	2.495875	-2.578696
H	2.792528	3.744597	-1.361052
H	2.008047	4.170457	-2.895865
C	0.316656	4.337701	-0.640636
H	0.384259	5.266133	-1.221006
H	0.996850	4.428763	0.211953
H	-0.705009	4.247867	-0.260272
C	-3.630104	-2.689917	-0.812240
H	-4.538589	-3.143083	-1.228640
H	-2.797861	-3.382204	-0.977213
H	-3.416788	-1.774622	-1.370188
C	-4.256387	-3.948552	1.889199
H	-5.185604	-4.370585	1.485711
H	-4.386352	-3.821838	2.968639
H	-3.460629	-4.684931	1.732235
C	-5.368861	-1.182491	1.208943
H	-5.159609	-0.160183	0.876725
H	-5.692325	-1.124864	2.253691
H	-6.210594	-1.567278	0.619467
C	-1.397183	0.294012	5.068947
H	-0.772640	1.017605	4.536397
H	-0.749419	-0.534955	5.375711
H	-1.784679	0.775215	5.975494
C	-3.775572	-1.612785	4.976906
H	-4.088808	-1.165739	5.929332
H	-3.187918	-2.507021	5.200918
H	-4.681873	-1.927895	4.449007
C	-4.037048	1.101998	3.670039
H	-5.054137	0.775580	3.912795
H	-4.046776	1.457269	2.636661
H	-3.799818	1.958944	4.311165
C	-1.033408	-4.026710	4.116486
H	-0.331687	-4.853877	4.284064
H	-2.026476	-4.457396	3.954075
H	-1.065818	-3.429782	5.033970
C	1.208112	-2.212626	3.111047
H	1.980892	-2.978564	3.253337
H	1.125042	-1.627317	4.032782
H	1.530609	-1.538963	2.310787
C	-0.127473	-4.155421	1.186404
H	0.320033	-3.589420	0.364086
H	-1.048802	-4.619687	0.818615
H	0.572160	-4.953221	1.465212
C	-2.895141	2.235306	0.186061
C	-3.999963	2.353181	-0.751933
C	-4.839728	3.449486	-0.697135
H	-5.652583	3.533370	-1.414571
C	-4.662002	4.445661	0.265948
H	-5.331959	5.300072	0.294139
C	-3.636671	4.334603	1.208049
H	-3.527401	5.101926	1.970918
C	-2.761946	3.261546	1.206501
C	-1.701688	3.160937	2.252209
H	-1.654733	2.152126	2.671407
H	-1.891600	3.868718	3.064026
H	-0.707480	3.362561	1.840820
C	-4.201858	1.275911	-1.765309
H	-3.256858	1.029604	-2.252653
H	-4.934096	1.577814	-2.519514
H	-4.554666	0.353598	-1.288086
C	4.462555	4.427385	1.143400
H	3.527514	4.960389	0.940940
H	4.566888	3.674975	0.353843
H	5.294460	5.132092	1.064324
C	0.547302	1.085160	1.447980

Calculated energies and coordinates of **TS6**

Electronic energy ... -3905.79521412 Eh
Total Enthalpy ... -3904.43567539 Eh
Final Gibbs free energy ... -3904.61778444 Eh

CARTESIAN COORDINATES (ANGSTROEM)

Si	-0.647518	0.365242	0.097020
Si	-1.532268	-0.858224	2.142583
Si	-3.554338	-1.824027	1.349914
Si	-2.025581	0.265255	4.173661
Si	-0.040295	-2.613246	2.671492
N	1.803642	-1.628859	-2.435997
N	0.328516	-0.627942	-0.859814
N	0.813184	2.786229	3.189497
N	0.942701	0.295420	-3.036627
N	-2.051505	0.949394	-0.588925
C	4.606282	-0.567958	-1.511333
H	5.441525	-1.226748	-1.777870
H	5.026271	0.393764	-1.196752
H	4.008316	-0.387412	-2.406120
C	3.795281	-1.178823	-0.353194
H	2.925638	-0.532953	-0.159833
C	3.250132	-2.561380	-0.690885
C	2.266501	-2.751429	-1.681736
C	0.946227	-0.627261	-1.986828
C	0.663327	1.891309	2.327019
C	1.831090	3.141773	4.042393
C	1.508168	4.139979	4.998313
C	2.487172	4.563317	5.887646
H	2.239724	5.323990	6.623537
C	3.771644	4.028701	5.845762
H	4.527971	4.371429	6.545573
C	3.138540	2.592929	3.994439
C	3.507181	1.548876	2.987101
H	3.305962	1.882779	1.962002
H	2.926690	0.630537	3.123098
H	4.567765	1.299491	3.073512
C	4.085905	3.056080	4.902512
H	5.091099	2.643063	4.867532
C	4.683220	-1.161079	0.893658
H	4.188902	-1.580968	1.772799
H	4.953520	-0.126885	1.123351
H	5.616603	-1.710446	0.721837
C	3.721733	-3.699600	-0.034314
H	4.469898	-3.601067	0.743768
C	3.259163	-4.967189	-0.363964
H	3.650157	-5.835717	0.158969
C	2.301812	-5.128970	-1.352508
H	1.945878	-6.124926	-1.595001
C	1.783623	-4.023966	-2.028977
C	0.676822	-4.193995	-3.054135
H	0.755200	-3.378466	-3.782065
C	0.757563	-5.510133	-3.831945
H	1.754289	-5.671607	-4.256931
H	0.032387	-5.497954	-4.652092
H	0.514134	-6.371622	-3.200883
C	-0.686553	-4.048029	-2.364482
H	-0.822377	-4.839267	-1.618300
H	-1.500875	-4.115928	-3.094916
H	-0.762652	-3.084996	-1.852380
C	2.268284	-1.344525	-3.729301
C	3.241857	-2.213664	-4.437184
H	3.554069	-1.730585	-5.366308
H	2.819484	-3.193501	-4.690208
H	4.132433	-2.397827	-3.824728

C	1.738523	-0.157738	-4.102273
C	1.961303	0.630262	-5.350130
H	1.549878	0.126747	-6.232139
H	3.029936	0.790025	-5.531763
H	1.487898	1.613135	-5.273815
C	0.164657	1.490661	-3.043238
C	0.629688	2.597473	-2.310778
C	-0.155672	3.751846	-2.324291
H	0.162756	4.627866	-1.769650
C	-1.348576	3.792057	-3.030218
H	-1.965653	4.685239	-2.998946
C	-1.766175	2.697304	-3.776038
H	-2.700191	2.762116	-4.321412
C	-1.014681	1.523630	-3.811409
C	-1.411953	0.343782	-4.692074
H	-0.597348	0.230598	-5.420043
C	-2.673036	0.602227	-5.521158
H	-3.557901	0.725716	-4.887724
H	-2.852253	-0.254631	-6.178196
H	-2.573421	1.490949	-6.152735
C	-1.518093	-1.000869	-3.926391
H	-1.600031	-0.851814	-2.847227
H	-0.641184	-1.628739	-4.104884
H	-2.398014	-1.562219	-4.257548
C	1.981627	2.568054	-1.608902
H	2.094130	1.585057	-1.133047
C	3.112808	2.738936	-2.638105
H	3.133689	1.920818	-3.360559
H	4.084584	2.765921	-2.133131
H	2.985756	3.680279	-3.185395
C	2.140749	3.623020	-0.512710
H	2.161199	4.637874	-0.925806
H	3.095060	3.468430	0.001816
H	1.349628	3.564308	0.238865
C	-3.297145	-2.563916	-0.365419
H	-4.253616	-2.868161	-0.809241
H	-2.668520	-3.456562	-0.284953
H	-2.805491	-1.864115	-1.045188
C	-4.071998	-3.282331	2.445768
H	-5.027044	-3.677885	2.074598
H	-4.207466	-3.010708	3.495985
H	-3.337341	-4.092819	2.392175
C	-4.939699	-0.541339	1.386581
H	-4.659606	0.384636	0.875566
H	-5.191923	-0.285274	2.421581
H	-5.848285	-0.933818	0.912409
C	-0.526744	0.385760	5.318386
H	0.317782	0.908326	4.863486
H	-0.185952	-0.606992	5.630074
H	-0.809886	0.936259	6.225129
C	-3.303350	-0.779261	5.104138
H	-3.481675	-0.324509	6.087153
H	-2.955385	-1.803870	5.270129
H	-4.263398	-0.825769	4.581007
C	-2.768590	1.982235	3.935618
H	-3.252585	2.300351	4.867892
H	-3.521493	1.999310	3.140005
H	-1.995651	2.716123	3.690257
C	-0.627045	-3.508806	4.235602
H	0.046272	-4.352182	4.436470
H	-1.639535	-3.910291	4.127975
H	-0.614090	-2.857352	5.114788
C	1.679202	-1.920554	2.987193
H	2.405582	-2.731712	3.122147
H	1.694964	-1.289206	3.882107
H	1.984501	-1.308896	2.132657
C	0.043734	-3.868619	1.274691
H	0.353079	-3.382173	0.344744
H	-0.925564	-4.349725	1.105070
H	0.775174	-4.652819	1.505271
C	-2.993765	1.901719	-0.661910
C	-4.171053	1.643511	-1.457753
C	-5.131605	2.628297	-1.632393
H	-6.002122	2.408783	-2.246338
C	-5.009325	3.878268	-1.033135
H	-5.775314	4.634900	-1.175947
C	-3.894278	4.144224	-0.243079
H	-3.792578	5.117739	0.231310
C	-2.895723	3.201911	-0.045820
C	-1.713394	3.562548	0.785985
H	-1.588945	2.874241	1.621974
H	-1.806259	4.578366	1.179915
H	-0.795059	3.496282	0.198078
C	-4.370961	0.310737	-2.101197
H	-3.467056	-0.018783	-2.612948
H	-5.199963	0.344096	-2.814477
H	-4.598200	-0.454910	-1.353172
C	0.121189	4.708047	5.048885
H	-0.610222	3.934999	5.310924
H	-0.181278	5.101047	4.072428
H	0.058512	5.509772	5.789366
C	0.342973	1.122165	1.376058

Calculated energies and coordinates of **4a**

Electronic energy ... -3905.87949811 Eh
Total Enthalpy ... -3904.51741844 Eh
Final Gibbs free energy ... -3904.70105964 Eh

CARTESIAN COORDINATES (ANGSTROEM)

Si	9.561067	5.492977	14.066858
Si	7.851045	2.735055	13.246546
Si	8.113232	0.428206	13.754092
Si	5.807622	3.465385	14.230415
Si	7.631221	2.877961	10.893646
N	13.080785	7.084965	15.179014
N	11.050619	5.985574	14.605918
N	11.124732	2.329176	15.174854
N	12.804070	6.615203	13.061617
N	8.189074	6.318107	13.911532
C	12.184598	6.513635	14.296035
C	14.215128	7.555058	14.496546
C	14.046007	7.263956	13.186108
C	12.807516	7.369183	16.554139
C	12.978793	6.358133	17.517210
C	12.732560	6.701437	18.847763
H	12.847105	5.957978	19.627367
C	12.334810	7.985574	19.200148
H	12.146136	8.222433	20.243526
C	12.174622	8.961224	18.230527
H	11.853720	9.957407	18.518670
C	12.413779	8.675250	16.884971
C	13.395741	4.943841	17.130816
H	12.694141	4.609301	16.350656
C	14.818104	4.873690	16.545667
H	14.919205	5.422676	15.607357
H	15.077899	3.828144	16.345647
H	15.546358	5.267975	17.264353
C	13.307236	3.959161	18.297690
H	13.505666	2.944428	17.941167
H	12.318250	3.952209	18.762759
H	14.053196	4.191281	19.067418
C	12.184037	9.745227	15.831790
H	12.715923	9.455794	14.917724
C	12.714253	11.120166	16.257656
H	12.682000	11.809232	15.407279
H	13.746811	11.067569	16.619542
H	12.101633	11.560520	17.051290
C	10.691064	9.824173	15.488107
H	10.499868	10.609293	14.748591
H	10.098185	10.049386	16.380981
H	10.323421	8.878385	15.082686
C	15.326938	8.233824	15.208231
H	16.180989	8.357201	14.538772
H	15.649846	7.657770	16.082118
H	15.033244	9.227463	15.567691
C	14.915817	7.529980	12.011461
H	14.934121	6.674916	11.327830
H	15.938443	7.730295	12.339181
H	14.571977	8.397174	11.435558
C	12.212647	6.246084	11.815250

C	12.230067	4.892132	11.430873
C	11.706961	4.576545	10.177339
H	11.700213	3.546568	9.839424
C	11.186975	5.563438	9.348137
H	10.777706	5.290396	8.379594
C	11.170306	6.887906	9.755500
H	10.746530	7.645164	9.102729
C	11.689818	7.260933	10.997138
C	12.832756	3.825699	12.331904
H	12.531143	4.053004	13.362641
C	12.329547	2.417745	12.015505
H	11.236812	2.373157	12.031283
H	12.698314	1.721120	12.772895
H	12.685715	2.063001	11.041331
C	14.369452	3.850410	12.277017
H	14.719826	3.694581	11.249848
H	14.776616	3.046060	12.900011
H	14.779518	4.794287	12.645644
C	11.651458	8.715307	11.430573
H	12.303000	8.832464	12.304189
C	12.161215	9.663369	10.336079
H	13.127410	9.344489	9.931687
H	12.272581	10.674489	10.741634
H	11.455848	9.726224	9.500651
C	10.233690	9.113703	11.859770
H	9.542666	9.050845	11.012705
H	10.220632	10.144353	12.229878
H	9.849894	8.465066	12.652161
C	6.025604	3.852957	16.062955
H	6.751046	4.660346	16.198333
H	6.358418	2.981078	16.635958
H	5.069688	4.188222	16.484315
C	5.046084	4.973987	13.400293
H	4.980451	4.865893	12.312708
H	5.641350	5.863980	13.620596
H	4.029678	5.128005	13.784280
C	4.548163	2.053752	14.105542
H	4.359746	1.750704	13.070356
H	3.594506	2.392937	14.529580
H	4.864051	1.168705	14.667504
C	5.889556	2.289786	10.440631
H	5.768562	2.298419	9.350122
H	5.118293	2.939590	10.866480
H	5.698691	1.269713	10.789324
C	7.840037	4.644097	10.282218
H	7.830568	4.678796	9.185387
H	8.779980	5.086847	10.626158
H	7.026193	5.272321	10.653957
C	8.844699	1.755543	9.971089
H	8.735250	1.906161	8.889526
H	8.649810	0.698347	10.180182
H	9.885689	1.962968	10.235418
C	6.811540	-0.608313	12.852404
H	6.969151	-0.582814	11.768742
H	5.786456	-0.285772	13.052843
H	6.903268	-1.653896	13.172671
C	9.790416	-0.243362	13.194198
H	9.894988	-1.287647	13.515382
H	10.617002	0.329228	13.621117
H	9.890867	-0.214264	12.104133
C	7.945702	0.125715	15.612800
H	6.983252	0.481720	15.996158
H	8.739205	0.626671	16.178544
H	8.017158	-0.947318	15.830647
C	9.378049	3.674114	13.933138
C	10.254262	2.991835	14.636539
C	11.265505	1.792894	16.465647
C	12.238018	0.787189	16.615871
C	12.379525	0.178189	17.860836
H	13.122684	-0.605118	17.984122
C	11.589777	0.563003	18.937270
H	11.706837	0.075738	19.900649
C	10.665329	1.588299	18.784245
H	10.069449	1.913211	19.633440
C	10.493807	2.231728	17.559740
C	13.118294	0.391367	15.465425
H	12.542346	-0.064108	14.653136
H	13.875966	-0.325868	15.791127
H	13.624441	1.266658	15.042905
C	9.549882	3.389299	17.437337
H	10.063524	4.277162	17.044325
H	9.126947	3.644845	18.412240
H	8.721196	3.175680	16.755170
C	7.716432	7.568940	14.236545
C	7.087392	8.366213	13.244269
C	6.562926	9.609961	13.586079
H	6.098421	10.213350	12.808542
C	6.610112	10.082813	14.892588
H	6.185220	11.049688	15.145370
C	7.202922	9.294868	15.872266
H	7.240464	9.650409	16.900315
C	7.765942	8.056523	15.571665
C	6.979569	7.863734	11.834961
H	6.741851	8.680480	11.146085
H	6.185002	7.112199	11.751883
H	7.901885	7.375055	11.509484
C	8.430388	7.256180	16.654385
H	8.031265	6.236464	16.708767
H	8.287003	7.730608	17.629727
H	9.512430	7.159299	16.486847

Calculated energies and coordinates of **INT6**

Electronic energy	... -3905.86621871 Eh
Total Enthalpy	... -3904.50488171 Eh
Final Gibbs free energy	... -3904.69081056 Eh

CARTESIAN COORDINATES (ANGSTROEM)

Si	0.865875	0.521204	-0.040676
Si	-1.607455	2.345490	1.601619
Si	-1.519502	4.295989	0.262926
Si	-3.424358	0.871907	1.265336
Si	-1.813961	3.212771	3.808268
N	2.305331	-0.162172	0.193368
N	-0.210849	0.383853	-3.870771
N	0.085821	0.527544	-1.515124
N	1.594885	1.781824	3.304237
N	0.787118	-1.333492	-2.962285
C	0.049476	1.432393	1.334066
C	0.242084	-0.096784	-2.649113
C	0.841481	1.528241	2.394576
C	3.224185	-0.224929	1.213193
C	4.126373	0.845150	1.448956
C	0.350885	2.719650	-4.267913
C	3.342613	-1.401053	1.996438
C	2.063462	1.197118	4.480591
C	-0.629705	1.720730	-4.161438
C	5.089734	0.727634	2.446533
H	5.778219	1.554674	2.611916
C	0.063650	-0.524518	-4.902780
C	1.452809	-2.321141	-2.156989
C	4.304917	-1.466325	3.002493
H	4.367049	-2.366825	3.610628
C	5.182192	-0.414286	3.236258
C	2.857637	-2.350038	-2.182108
C	0.662179	-1.596848	-4.341786
C	4.024761	2.090161	0.619888
H	4.042207	1.850767	-0.449927
H	4.846071	2.778366	0.842295
H	3.079419	2.622432	0.801713
C	2.454596	-2.573452	1.706760
H	1.399683	-2.285990	1.625432
H	2.550414	-3.342936	2.479410
H	2.710114	-3.016236	0.739451
C	3.047272	1.914108	5.183142
C	1.592056	-0.049921	4.932963
C	0.682465	-3.352920	-1.605738
C	-1.977126	1.956182	-4.479173
C	1.815434	2.459113	-3.973502
H	1.937886	1.400706	-3.721738
C	3.677005	-1.144593	-2.617836

H	3.062400	-0.261127	-2.409752
C	-0.277442	-0.232885	-6.317908
H	-1.360040	-0.224524	-6.486270
H	0.161076	-0.989678	-6.970945
H	0.104170	0.748154	-6.622567
C	2.131454	-0.563853	6.110041
H	1.784389	-1.529284	6.469127
C	-0.056332	3.988280	-4.682923
H	0.678858	4.783629	-4.769252
C	3.557344	1.361067	6.353764
H	4.325346	1.900006	6.902131
C	1.352398	-4.503945	-1.185375
H	0.791099	-5.330944	-0.762234
C	-0.825335	-3.220772	-1.462513
H	-1.053968	-2.149777	-1.404460
C	3.478398	-3.523979	-1.757639
H	4.561514	-3.591484	-1.768153
C	3.543506	3.231277	4.664424
H	3.881807	3.134441	3.627721
H	4.373768	3.597067	5.274029
H	2.750778	3.987999	4.673617
C	-2.331424	3.237565	-4.901057
H	-3.367109	3.452902	-5.146980
C	1.111656	-2.877161	-4.948788
H	0.571041	-3.734201	-4.532511
H	2.176103	-3.062040	-4.775313
H	0.935628	-2.854064	-6.026307
C	0.250633	4.946258	0.387317
H	0.379732	5.827512	-0.253355
H	0.970590	4.186666	0.065737
H	0.509764	5.228338	1.412759
C	2.731590	-4.604381	-1.302365
H	3.235505	-5.514311	-0.988431
C	-1.381314	4.243622	-5.004611
H	-1.677453	5.235705	-5.334076
C	-3.042001	0.886223	-4.326674
H	-2.539736	-0.080088	-4.198386
C	2.282828	3.276248	-2.763485
H	2.209675	4.352346	-2.957771
H	3.326490	3.045200	-2.525440
H	1.671163	3.046329	-1.886363
C	0.555900	-0.807563	4.164351
H	-0.394262	-0.263149	4.119616
H	0.372641	-1.783431	4.621217
H	0.880591	-0.965283	3.132691
C	-2.896145	-0.526025	0.113614
H	-2.357638	-0.163065	-0.765637
H	-3.760072	-1.116848	-0.216207
H	-2.218343	-1.194312	0.657257
C	3.105330	0.130827	6.816409
C	-3.584426	3.873756	3.956958
H	-3.846167	4.564354	3.150632
H	-3.693419	4.412590	4.906617
H	-4.320076	3.062603	3.953978
C	4.955925	-1.001254	-1.783980
H	4.750048	-1.091127	-0.714407
H	5.398924	-0.015507	-1.962321
H	5.707619	-1.747843	-2.065626
C	-4.904187	1.819211	0.568606
H	-5.217626	2.614009	1.255287
H	-5.760442	1.150328	0.417825
H	-4.665780	2.284380	-0.393332
C	-0.611762	4.640808	4.111625
H	0.422043	4.318173	3.948022
H	-0.697281	4.984248	5.150152
H	-0.806353	5.496566	3.457858
C	-1.916260	3.942334	-1.536590
H	-1.345232	3.087852	-1.912472
H	-1.675524	4.810966	-2.160384
H	-2.980476	3.722797	-1.671352
C	-3.954415	0.039903	2.880448
H	-3.128476	-0.538742	3.309520
H	-4.780705	-0.655761	2.686629
H	-4.291083	0.755392	3.636497
C	4.034762	-1.148375	-4.113007
H	4.555790	-2.073285	-4.388631
H	4.707262	-0.310652	-4.329899
H	3.154442	-1.038587	-4.752967
C	2.693298	2.734114	-5.202014
H	2.366251	2.143122	-6.064149
H	3.734735	2.473469	-4.984929
H	2.665804	3.791318	-5.487102
C	-1.332675	-3.863273	-0.166496
H	-1.270712	-4.956226	-0.204220
H	-2.384420	-3.602731	-0.012495
H	-0.760366	-3.518800	0.701312
C	-2.708828	5.654632	0.830356
H	-3.750839	5.317296	0.797806
H	-2.618727	6.510019	0.148764
H	-2.498105	6.012729	1.842407
C	-1.591596	-3.793407	-2.664757
H	-1.384217	-3.237118	-3.583612
H	-2.670546	-3.739005	-2.480264
H	-1.328135	-4.845386	-2.827446
C	-3.967637	0.785140	-5.546145
H	-4.607540	1.669210	-5.636207
H	-4.626092	-0.084088	-5.445003
H	-3.407750	0.684851	-6.481284
C	-1.569126	1.993408	5.230496
H	-2.163500	1.081845	5.115452
H	-1.888924	2.481883	6.160111
H	-0.519033	1.711234	5.356723
C	-3.865103	1.154452	-3.058474
H	-3.214736	1.260711	-2.187729
H	-4.569071	0.336336	-2.868911
H	-4.438540	2.082768	-3.165212
H	5.925885	-0.480917	4.024403
H	3.516595	-0.290665	7.729072

Calculated energies and coordinates of **TS7**

Electronic energy ... -3905.84856690 Eh
Total Enthalpy ... -3904.48833096 Eh
Final Gibbs free energy ... -3904.67184453 Eh

CARTESIAN COORDINATES (ANGSTROEM)

Si	0.258596	0.113936	0.288150
Si	-2.351732	1.229457	2.330943
Si	-2.446276	3.373519	3.329798
Si	-3.895656	1.147283	0.556514
Si	-2.934653	-0.403361	3.934826
N	1.749903	0.471359	0.954060
N	0.013660	-0.322504	-3.535416
N	-0.198255	-0.493697	-1.159294
N	0.997760	1.350788	3.439823
N	0.613080	-2.220926	-2.632069
C	-0.665445	0.805705	1.638317
C	0.140591	-0.964580	-2.321373
C	0.507508	1.030032	2.343384
C	2.911829	-0.167128	1.365936
C	4.162534	0.457623	1.146688
C	-0.041417	2.122628	-3.400628
C	2.866316	-1.413431	2.035246
C	2.229638	1.931653	3.735675
C	-0.673861	0.918067	-3.743880
C	5.319846	-0.139303	1.635617
H	6.275352	0.354418	1.475316
C	0.408094	-1.174535	-4.580372
C	1.013202	-3.206988	-1.673588
C	4.050037	-1.985450	2.499382
H	4.005769	-2.939133	3.021317
C	5.273052	-1.351404	2.316865
C	2.386759	-3.445655	-1.502006
C	0.769187	-2.355378	-4.022916
C	4.242629	1.722917	0.349332
H	3.830837	1.570016	-0.656071
H	5.279241	2.058795	0.255240
H	3.660850	2.522521	0.814721
C	1.557458	-2.115384	2.237575
H	0.866889	-1.516117	2.842478
H	1.703415	-3.079516	2.732750

H	1.059124	-2.320775	1.277693
C	2.623929	3.132775	3.111259
C	3.029155	1.337443	4.730283
C	0.020839	-3.912971	-0.969099
C	-1.956900	0.870530	-4.316770
C	1.345330	2.145343	-2.781631
H	1.426396	1.282549	-2.110311
C	3.446627	-2.669485	-2.266113
H	3.003557	-2.293785	-3.193513
C	0.410764	-0.729387	-5.997360
H	-0.599033	-0.535607	-6.375729
H	0.873461	-1.492729	-6.625942
H	0.979828	0.199707	-6.115395
C	4.251961	1.922859	5.042688
H	4.884672	1.453124	5.791815
C	-0.727662	3.307305	-3.671645
H	-0.278150	4.259381	-3.412651
C	3.854652	3.687748	3.459717
H	4.171869	4.607785	2.973639
C	0.450574	-4.869140	-0.049231
H	-0.278526	-5.429576	0.525067
C	-1.458300	-3.658844	-1.215264
H	-1.597566	-2.571525	-1.254930
C	2.759857	-4.406006	-0.559345
H	3.812144	-4.598437	-0.378448
C	1.731801	3.807819	2.107393
H	1.473022	3.138463	1.279482
H	2.218502	4.696962	1.695525
H	0.787581	4.121618	2.566501
C	-2.590921	2.084312	-4.581267
H	-3.582737	2.087360	-5.021817
C	1.206030	-3.631035	-4.649510
H	2.277900	-3.822480	-4.525148
H	0.986515	-3.613101	-5.719061
H	0.674798	-4.480732	-4.207426
C	-2.216994	4.699720	1.996127
H	-2.126167	5.696924	2.443936
H	-3.074728	4.714142	1.313873
H	-1.319594	4.514368	1.395707
C	1.803658	-5.107909	0.157205
H	2.113622	-5.848436	0.889205
C	-1.981323	3.289482	-4.266148
H	-2.494633	4.224694	-4.470715
C	-2.687605	-0.441555	-4.550090
H	-1.949367	-1.240552	-4.682603
C	1.598669	3.392773	-1.932764
H	1.686965	4.295138	-2.548132
H	2.537022	3.278520	-1.383699
H	0.798208	3.547680	-1.200947
C	2.570128	0.078061	5.401297
H	1.580085	0.211097	5.852102
H	3.273360	-0.227198	6.180893
H	2.486125	-0.738264	4.674365
C	-3.157500	2.031190	-0.939373
H	-2.864310	3.053776	-0.676578
H	-3.873003	2.091613	-1.767958
H	-2.262652	1.516579	-1.305699
C	4.673342	3.086944	4.408234
C	-4.788390	-0.365138	4.322649
H	-5.106111	0.616489	4.690110
H	-5.036177	-1.106562	5.092351
H	-5.386378	-0.595342	3.433799
C	3.893049	-1.445482	-1.462605
H	3.045751	-0.788345	-1.241963
H	4.639086	-0.868858	-2.021986
H	4.333790	-1.744556	-0.506863
C	-5.545816	1.971781	0.977408
H	-5.974774	1.583809	1.907675
H	-6.268902	1.786927	0.173099
H	-5.437086	3.056481	1.082895
C	-1.971023	-0.139517	5.537864
H	-0.901051	-0.024185	5.332142
H	-2.105271	-0.987133	6.221214
H	-2.307960	0.765602	6.054873
C	-4.153331	3.606056	4.119094
H	-4.976713	3.411467	3.425937
H	-4.255383	4.635316	4.485496
H	-4.273832	2.939042	4.980223
C	-4.265247	-0.664467	0.142904
H	-3.340723	-1.226829	-0.030303
H	-4.896992	-0.758165	-0.748178
H	-4.791412	-1.141829	0.977374
C	4.654860	-3.532401	-2.647209
H	5.264136	-3.780261	-1.771836
H	5.298336	-2.980519	-3.340170
H	4.354605	-4.470296	-3.127161
C	2.430392	1.992794	-3.858559
H	2.337558	1.039430	-4.388321
H	3.424969	2.026378	-3.399974
H	2.363579	2.804420	-4.592378
C	-2.358360	-4.184839	-0.096154
H	-2.389761	-5.280567	-0.080467
H	-3.382124	-3.831306	-0.256326
H	-2.032752	-3.831941	0.886744
C	-1.207474	3.657020	4.727798
H	-1.398039	2.965206	5.555136
H	-1.322023	4.679183	5.111558
H	-0.167244	3.512592	4.428931
C	-1.911670	-4.245338	-2.562960
H	-1.421660	-3.757538	-3.410343
H	-2.992103	-4.111254	-2.682494
H	-1.696086	-5.319298	-2.606861
C	-3.579890	-0.431369	-5.795558
H	-4.456001	0.211532	-5.660996
H	-3.950628	-1.442541	-5.992648
H	-3.040598	-0.084460	-6.683537
C	-2.513893	-2.118246	3.256257
H	-3.167342	-2.373777	2.415744
H	-2.635818	-2.887736	4.028468
H	-1.478728	-2.152721	2.898891
C	-3.507925	-0.794499	-3.302150
H	-2.875241	-0.833850	-2.411300
H	-4.000086	-1.765859	-3.425298
H	-4.279748	-0.037216	-3.126397
H	6.185371	-1.801135	2.697875
H	5.631143	3.531367	4.662447

Calculated energies and coordinates of **INT7**

Electronic energy ... -3905.85566020 Eh
Total Enthalpy ... -3904.49402287 Eh
Final Gibbs free energy ... -3904.67897058 Eh

CARTESIAN COORDINATES (ANGSTROEM)

Si	0.538302	0.545361	-0.083316
Si	-1.707323	2.234715	1.960273
Si	-1.163853	4.473942	2.519489
Si	-3.408298	2.151524	0.335626
Si	-2.532177	1.057259	3.844320
N	1.829518	0.259843	1.076870
N	0.159647	-0.011174	-4.016457
N	0.339044	0.209994	-1.660659
N	1.296457	1.510166	3.164231
N	1.170045	-1.645451	-2.980204
C	-0.230494	1.367589	1.249414
C	0.557413	-0.431783	-2.764989
C	0.997905	1.096149	1.990657
C	3.250059	0.359743	1.045752
C	3.860371	1.539572	0.575933
C	-0.201618	2.391146	-4.327718
C	4.039303	-0.726210	1.461911
C	2.409118	1.105533	3.911331
C	-0.715569	1.089860	-4.292677
C	5.249506	1.642741	0.586925
H	5.716357	2.561604	0.239354
C	0.516184	-0.957328	-4.987271
C	1.820316	-2.473626	-2.011557
C	5.429831	-0.591841	1.443350
H	6.039923	-1.425975	1.781725
C	6.035611	0.585283	1.027861
C	3.225433	-2.558361	-2.063324

C	1.129162	-1.979735	-4.344438
C	3.024019	2.655890	0.027279
H	2.531840	2.347708	-0.906788
H	3.637866	3.531657	-0.202282
H	2.231436	2.959452	0.716998
C	3.435731	-2.004176	1.957213
H	3.283963	-1.947161	3.039939
H	4.106198	-2.843950	1.756781
H	2.469307	-2.204665	1.490678
C	3.467068	2.004854	4.153214
C	2.379264	-0.143796	4.569006
C	1.041632	-3.266578	-1.158520
C	-2.057991	0.796285	-4.592398
C	1.243076	2.702760	-3.991026
H	1.675598	1.826668	-3.495358
C	4.035285	-1.622378	-2.945658
H	3.483629	-1.468816	-3.881461
C	0.214749	-0.750719	-6.426075
H	-0.860233	-0.798072	-6.634707
H	0.715181	-1.512145	-7.027273
H	0.561719	0.233237	-6.760369
C	3.448166	-0.502731	5.387490
H	3.427356	-1.469904	5.885964
C	-1.068495	3.416447	-4.712767
H	-0.703387	4.437963	-4.754470
C	4.518414	1.604269	4.975978
H	5.343057	2.293555	5.144700
C	1.707669	-4.206654	-0.368356
H	1.136379	-4.846504	0.298229
C	-0.470396	-3.161692	-1.097462
H	-0.783898	-2.309052	-1.707836
C	3.838453	-3.512564	-1.254639
H	4.918006	-3.616039	-1.266971
C	3.477501	3.368770	3.524250
H	3.954716	3.341656	2.537076
H	4.039454	4.075009	4.143001
H	2.461511	3.748933	3.385665
C	-2.879128	1.853170	-4.979885
H	-3.920944	1.664814	-5.218946
C	1.643238	-3.278789	-4.848507
H	1.397735	-3.390128	-5.906309
H	1.189444	-4.113442	-4.301857
H	2.728732	-3.375989	-4.733904
C	0.064308	5.170795	1.258455
H	0.209267	6.245721	1.424468
H	-0.286321	5.029004	0.230023
H	1.039864	4.683008	1.349922
C	3.084709	-4.341534	-0.431665
H	3.582434	-5.086491	0.182705
C	-2.386401	3.150067	-5.048476
H	-3.042070	3.961701	-5.350607
C	-2.628800	-0.598237	-4.403084
H	-1.816248	-1.328453	-4.496809
C	1.351617	3.881113	-3.016055
H	1.022913	4.818939	-3.475874
H	2.392607	4.018628	-2.706829
H	0.744651	3.708830	-2.120940
C	1.194398	-1.054109	4.407539
H	0.289593	-0.590420	4.815114
H	1.358651	-2.001655	4.929580
H	0.986283	-1.272482	3.353904
C	-2.721413	2.683033	-1.343507
H	-2.355172	3.715468	-1.294606
H	-3.491230	2.641618	-2.123721
H	-1.883947	2.053425	-1.664474
C	4.525552	0.354321	5.582202
C	-4.351059	1.485886	4.171328
H	-4.480253	2.559236	4.352883
H	-4.711268	0.954673	5.061296
H	-4.997655	1.208831	3.332147
C	4.171875	-0.248056	-2.274177
H	3.193456	0.181478	-2.036744
H	4.700616	0.448577	-2.935116
H	4.733171	-0.330473	-1.339122
C	-4.896755	3.253911	0.724355
H	-5.259711	3.108985	1.746952
H	-5.721368	3.017747	0.039993
H	-4.657334	4.314591	0.604881
C	-1.599869	1.431335	5.447536
H	-0.519646	1.497582	5.288387
H	-1.798925	0.654191	6.196377
H	-1.931764	2.387463	5.866355
C	-2.690182	5.597996	2.491231
H	-3.046513	5.766522	1.469743
H	-2.438260	6.574949	2.922635
H	-3.521584	5.181805	3.070781
C	-4.065570	0.375687	0.226891
H	-3.254675	-0.347377	0.085356
H	-4.775532	0.264962	-0.601104
H	-4.590249	0.108180	1.150410
C	5.417031	-2.167589	-3.311710
H	6.083000	-2.188385	-2.442138
H	5.882319	-1.515050	-4.057151
H	5.363698	-3.179322	-3.728510
C	2.054837	2.969339	-5.266923
H	2.032119	2.108341	-5.943522
H	3.100771	3.178137	-5.016875
H	1.654201	3.833981	-5.807970
C	-0.955831	-2.899147	0.334491
H	-0.746005	-3.747510	0.994923
H	-2.037825	-2.729832	0.343658
H	-0.475526	-2.013694	0.767767
C	-0.396344	4.622361	4.239858
H	-1.163734	4.561942	5.018996
H	0.111221	5.589605	4.345850
H	0.329303	3.823713	4.420770
C	-1.134768	-4.415629	-1.683062
H	-0.829544	-4.578994	-2.722304
H	-2.225221	-4.313250	-1.659212
H	-0.866863	-5.309461	-1.109044
C	-3.700072	-0.969515	-5.432999
H	-4.621121	-0.396168	-5.284970
H	-3.960706	-2.027901	-5.330503
H	-3.357781	-0.798699	-6.459311
C	-2.428160	-0.795797	3.464246
H	-3.160294	-1.079392	2.700641
H	-2.622266	-1.395153	4.362323
H	-1.436712	-1.059210	3.083247
C	-3.185698	-0.714679	-2.977037
H	-2.428639	-0.451499	-2.232002
H	-3.539233	-1.732930	-2.776556
H	-4.026985	-0.026383	-2.845710
H	7.118073	0.676024	1.037707
H	5.351866	0.059512	6.222339

Calculated energies and coordinates of **INT8**

Electronic energy ... -4308.87657134 Eh
Total Enthalpy ... -4307.34875024 Eh
Final Gibbs free energy ... -4307.55020458 Eh

CARTESIAN COORDINATES (ANGSTROEM)

Si	0.118440	-0.027427	0.205166
Si	-1.789584	2.441585	1.891109
Si	-0.997377	4.643255	2.328418
Si	-3.517423	2.609600	0.296249
Si	-2.781639	1.597787	3.877712
N	1.509171	0.107658	1.293601
N	0.043666	-0.183215	-3.803872
N	0.141091	-0.271326	-1.426437
N	1.152076	1.802776	3.058143
N	1.119251	-1.887487	-2.959392
N	-1.432598	-2.387205	1.447793
C	-0.446306	1.328445	1.244779
C	-0.767846	-1.573620	0.924006
C	0.411969	-0.747794	-2.589631
C	0.786374	1.162116	1.996760
C	-1.589259	-4.172555	3.031495
C	2.924155	0.029350	1.295371
C	3.673045	1.144604	0.870824
C	-0.396608	2.215950	-4.001848

C	3.576456	-1.138201	1.722054
C	2.215505	1.420155	3.880670
C	-2.190620	-3.357794	2.049361
C	-0.147945	-3.999669	3.389008
H	0.497142	-4.157245	2.515610
H	0.146978	-4.708504	4.165607
H	0.056679	-2.987940	3.749272
C	-0.869145	0.899302	-4.020634
C	5.060253	1.117625	0.996858
H	5.635832	1.987283	0.687484
C	0.529524	-0.940574	-4.878339
C	1.781881	-2.810088	-2.091192
C	-2.376566	-5.155690	3.619684
H	-1.935675	-5.789209	4.384146
C	4.969651	-1.142984	1.797163
H	5.473894	-2.040557	2.147725
C	5.711037	-0.014627	1.469793
C	3.181954	-2.731086	-2.000975
C	1.197629	-1.995835	-4.359681
C	2.992013	2.300984	0.205225
H	2.445623	1.953461	-0.679203
H	3.720992	3.051863	-0.113447
H	2.253851	2.782812	0.850719
C	-3.519956	-3.547555	1.618742
C	2.797445	-2.350804	2.133347
H	2.336964	-2.189168	3.113840
H	3.451866	-3.224258	2.204134
H	1.998174	-2.577231	1.421324
C	3.323093	2.270271	4.080002
C	2.102708	0.239271	4.652361
C	1.034160	-3.824080	-1.463830
C	-2.184563	0.573597	-4.405183
C	1.000619	2.578672	-3.540005
H	1.487457	1.669367	-3.168212
C	-3.700759	-5.342528	3.237379
C	3.962895	-1.659936	-2.742422
H	3.422924	-1.416909	-3.663695
C	0.292556	-0.537010	-6.287603
H	-0.768042	-0.591775	-6.560908
H	0.855832	-1.184281	-6.962369
H	0.611239	0.497406	-6.458172
C	3.137744	-0.125556	5.508097
H	3.045246	-1.042550	6.086956
C	-1.267210	3.221620	-4.430766
H	-0.928214	4.253310	-4.437689
C	4.335685	1.872759	4.953087
H	5.197648	2.524493	5.082027
C	-4.256623	-4.550951	2.238191
H	-5.284799	-4.712009	1.926105
C	1.738260	-4.740555	-0.679836
H	1.208502	-5.539791	-0.174376
C	-0.472415	-3.948978	-1.659178
H	-0.907026	-2.957988	-1.463821
C	3.832998	-3.665059	-1.193802
H	4.910838	-3.619366	-1.080476
C	3.447047	3.579629	3.352792
H	4.055923	3.468505	2.447182
H	3.936265	4.325921	3.987133
H	2.469729	3.962342	3.049008
C	-3.017362	1.613760	-4.810786
H	-4.035512	1.400949	-5.119492
C	1.885331	-3.130292	-5.031330
H	1.649355	-3.125914	-6.097464
H	1.562289	-4.090037	-4.614884
H	2.975335	-3.086788	-4.924162
C	0.223204	5.200797	0.994304
H	0.554387	6.227764	1.194357
H	-0.246800	5.183985	0.004733
H	1.109403	4.561773	0.950484
C	3.117718	-4.655565	-0.539602
H	3.639733	-5.376748	0.083175
C	-2.554936	2.924509	-4.842794
H	-3.214173	3.721357	-5.175374
C	-4.108269	-2.703529	0.533683
H	-4.295173	-1.682728	0.880989
H	-5.057968	-3.120660	0.190893
H	-3.435790	-2.622358	-0.327577
C	-2.680584	-0.861010	-4.351858
H	-1.838132	-1.521105	-4.595344
C	0.937116	3.580510	-2.378978
H	0.442785	4.510830	-2.679107
H	1.945610	3.838215	-2.040778
H	0.388671	3.163337	-1.528356
C	0.837293	-0.566788	4.604702
H	-0.003180	0.014475	4.999633
H	0.936531	-1.472720	5.211791
H	0.565576	-0.846856	3.581087
C	-2.825642	3.139975	-1.374036
H	-2.359036	4.130561	-1.327593
H	-3.617790	3.175407	-2.131448
H	-2.065511	2.432629	-1.721876
C	4.266332	0.674457	5.650419
C	-4.559133	2.218575	4.114652
H	-4.609700	3.313179	4.119019
H	-4.949089	1.865161	5.077726
H	-5.229498	1.853623	3.328962
C	4.023476	-0.378385	-1.910060
H	3.020459	-0.038474	-1.637663
H	4.521663	0.423195	-2.468235
H	4.578632	-0.551971	-0.985393
C	-4.931247	3.791253	0.731768
H	-5.335877	3.618396	1.732858
H	-5.746759	3.646512	0.011516
H	-4.620377	4.837828	0.675475
C	-1.851820	2.052866	5.463267
H	-0.767638	2.070447	5.319995
H	-2.088979	1.340030	6.263360
H	-2.153962	3.047849	5.807711
C	-2.438259	5.877900	2.262540
H	-2.783866	6.038741	1.236090
H	-2.108222	6.846566	2.658709
H	-3.296680	5.549042	2.858634
C	-4.299047	0.900978	0.056821
H	-3.529119	0.163877	-0.194822
H	-5.038620	0.911000	-0.753953
H	-4.805494	0.572655	0.971671
C	5.372656	-2.107317	-3.139269
H	6.030556	-2.183880	-2.266908
H	5.816819	-1.365675	-3.811010
H	5.368893	-3.076040	-3.651257
C	1.848167	3.125838	-4.696467
H	1.924434	2.402619	-5.515505
H	2.861466	3.353129	-4.347906
H	1.417436	4.048337	-5.101850
C	-1.119296	-4.952145	-0.701561
H	-0.841343	-5.980036	-0.962864
H	-2.209410	-4.884332	-0.773836
H	-0.844087	-4.780978	0.341111
C	-0.206064	4.861902	4.029384
H	-0.971255	4.893748	4.811905
H	0.343849	5.811257	4.062951
H	0.483105	4.045741	4.259898
C	-0.852348	-4.353082	-3.095208
H	-0.594955	-3.590439	-3.832597
H	-1.933698	-4.519540	-3.155776
H	-0.354257	-5.290183	-3.369806
C	-3.798858	-1.166260	-5.351096
H	-4.735592	-0.668580	-5.077747
H	-3.997643	-2.242913	-5.363121
H	-3.533070	-0.856431	-6.367355
C	-2.891744	-0.290729	3.748896
H	-3.439460	-0.586628	2.847017
H	-3.415968	-0.710982	4.616411
H	-1.897942	-0.744624	3.698372
C	-3.129419	-1.210100	-2.924143
H	-2.330480	-1.016516	-2.201376
H	-3.413861	-2.267400	-2.863597
H	-3.994583	-0.603712	-2.634853
H	-4.297401	-6.116474	3.710156
H	6.793065	-0.025468	1.564213
H	5.068876	0.380055	6.320222

Calculated energies and coordinates of **TS8**

Electronic energy ... -4308.86528647 Eh
Total Enthalpy ... -4307.33792743 Eh
Final Gibbs free energy ... -4307.53698598 Eh

CARTESIAN COORDINATES (ANGSTROEM)

Si	0.137332	-0.095696	0.193221
Si	-1.924204	2.183312	2.022467
Si	-1.120464	4.266518	2.852822
Si	-3.279160	2.807958	0.187536
Si	-3.314927	1.351060	3.767621
N	1.560703	0.159825	1.202303
N	-0.075636	-0.397585	-3.798663
N	0.154500	-0.324458	-1.429009
N	1.211966	1.674724	3.110494
N	1.090699	-2.013612	-2.900962
N	-1.731174	-1.536349	2.132686
C	-0.522982	1.085985	1.433417
C	-0.943811	-1.016876	1.366054
C	0.376420	-0.872086	-2.576041
C	0.828323	1.056328	2.051175
C	-1.932713	-3.707522	3.201331
C	2.976489	0.139709	1.119217
C	3.620182	1.287442	0.617005
C	-0.487218	2.010157	-3.897522
C	3.719263	-0.974941	1.531548
C	2.390925	1.372801	3.814896
C	-2.358200	-2.797233	2.221659
C	-0.751516	-3.398646	4.067861
H	0.163374	-3.287082	3.473682
H	-0.587342	-4.191743	4.801452
H	-0.891597	-2.454302	4.603700
C	-0.975332	0.697273	-3.999029
C	5.010235	1.348721	0.643352
H	5.509904	2.244337	0.281625
C	0.358377	-1.223939	-4.846451
C	1.929105	-2.795265	-2.041698
C	-2.617653	-4.915282	3.319328
H	-2.285523	-5.631556	4.066385
C	5.113877	-0.891320	1.504838
H	5.696083	-1.746249	1.840900
C	5.758011	0.268054	1.096930
C	3.320242	-2.632393	-2.172721
C	1.073169	-2.229113	-4.290830
C	2.816347	2.390767	-0.003963
H	2.292750	2.019637	-0.893518
H	3.459107	3.220850	-0.310749
H	2.043794	2.778820	0.664107
C	-3.457718	-3.071933	1.391554
C	3.064137	-2.223250	2.043110
H	2.931738	-2.158677	3.128872
H	3.689001	-3.094753	1.832467
H	2.081547	-2.387500	1.592200
C	3.426217	2.320643	3.946417
C	2.460055	0.166412	4.546004
C	1.350942	-3.774915	-1.217137
C	-2.297356	0.401917	-4.374650
C	0.968104	2.284335	-3.555720
H	1.250022	1.580338	-2.763982
C	-3.708567	-5.208935	2.510717
C	3.915780	-1.543035	-3.048967
H	3.245478	-1.376986	-3.899966
C	0.069530	-0.915257	-6.272088
H	0.690884	-1.538100	-6.918576
H	0.289761	0.134038	-6.495160
H	-0.979545	-1.082773	-6.542212
C	3.602772	-0.123466	5.287184
H	3.648360	-1.061691	5.836533
C	-1.380700	3.047422	-4.166826
H	-1.052590	4.077347	-4.082137
C	4.554174	1.992195	4.697536
H	5.359991	2.719066	4.771643
C	-4.122009	-4.286061	1.558182
H	-4.974379	-4.504777	0.919963
C	2.221617	-4.610285	-0.514581
H	1.822889	-5.380115	0.135643
C	-0.158299	-3.941250	-1.109453
H	-0.585539	-2.936320	-0.975938
C	4.142528	-3.486941	-1.438346
H	5.220418	-3.385365	-1.504446
C	3.329291	3.670495	3.299071
H	3.490777	3.614640	2.217096
H	4.082750	4.347505	3.711861
H	2.340761	4.110694	3.457204
C	-3.143313	1.476590	-4.649869
H	-4.171114	1.291684	-4.943482
C	1.727049	-3.410649	-4.910774
H	1.449442	-3.477702	-5.964586
H	1.410528	-4.334901	-4.414805
H	2.820282	-3.370167	-4.844113
C	0.218189	4.960755	1.712599
H	0.601704	5.909193	2.109789
H	-0.181126	5.151206	0.711253
H	1.058698	4.272068	1.616878
C	3.597950	-4.472588	-0.629537
H	4.253085	-5.137562	-0.073363
C	-2.690378	2.783689	-4.543554
H	-3.366227	3.608036	-4.753392
C	-3.887663	-2.088899	0.348967
H	-4.140312	-1.125226	0.801437
H	-4.758980	-2.455391	-0.199648
H	-3.086376	-1.896122	-0.369843
C	-2.806302	-1.030210	-4.429123
H	-1.990068	-1.675593	-4.771913
C	1.217121	3.695996	-3.022299
H	1.051530	4.457494	-3.792622
H	2.259146	3.784296	-2.700822
H	0.581743	3.927823	-2.161909
C	1.278556	-0.753820	4.586154
H	0.440680	-0.270850	5.101791
H	1.522619	-1.676086	5.122711
H	0.919435	-1.018962	3.588025
C	-2.461244	4.155358	-0.860468
H	-2.343954	5.099720	-0.321386
H	-3.067287	4.346258	-1.755089
H	-1.472009	3.828551	-1.196796
C	4.663549	0.771368	5.350118
C	-4.265937	2.805667	4.524124
H	-4.889312	3.322620	3.787274
H	-3.601002	3.543183	4.983025
H	-4.929204	2.421547	5.309662
C	3.988035	-0.231174	-2.261489
H	3.001635	0.062513	-1.892676
H	4.380135	0.577736	-2.889078
H	4.642713	-0.345194	-1.393066
C	-4.963029	3.456689	0.760195
H	-5.529362	2.687804	1.296833
H	-5.559538	3.767541	-0.106661
H	-4.862261	4.321596	1.423445
C	-2.283386	0.570179	5.139695
H	-1.829845	-0.357090	4.777823
H	-2.909785	0.332804	6.008522
H	-1.477457	1.236670	5.464557
C	-2.513710	5.558881	2.824484
H	-2.867043	5.765783	1.809449
H	-2.122483	6.499777	3.232979
H	-3.378640	5.272177	3.428883
C	-3.580587	1.360097	-0.982355
H	-2.634538	0.902910	-1.295307
H	-4.089595	1.709315	-1.889290
H	-4.202146	0.586242	-0.522760
C	5.292852	-1.899812	-3.616138
H	6.063070	-1.898451	-2.837487
H	5.586906	-1.151817	-4.359406
H	5.297384	-2.883158	-4.099155
C	1.882835	2.023193	-4.764766
H	1.871454	0.973038	-5.069739
H	2.916774	2.282238	-4.512311
H	1.576683	2.637996	-5.619284
C	-0.576443	-4.783354	0.096149

H	-0.284814	-5.832734	-0.028914
H	-1.663343	-4.757569	0.210150
H	-0.138470	-4.411049	1.027543
C	-0.484448	4.211335	4.630757
H	-1.288378	3.977507	5.337335
H	-0.076122	5.191455	4.909430
H	0.299318	3.457848	4.744559
C	-0.769421	-4.549117	-2.383671
H	-0.656977	-3.898838	-3.255293
H	-1.840990	-4.719109	-2.231524
H	-0.303195	-5.516363	-2.605479
C	-3.976773	-1.228211	-5.395377
H	-4.892816	-0.754101	-5.026785
H	-4.188238	-2.297056	-5.501723
H	-3.758639	-0.821609	-6.388809
C	-4.642251	0.097262	3.263735
H	-5.204233	0.414596	2.378426
H	-5.360464	-0.013027	4.086300
H	-4.213010	-0.889229	3.066440
C	-3.179994	-1.516523	-3.024165
H	-2.329561	-1.424127	-2.341922
H	-3.489607	-2.567441	-3.051829
H	-4.001057	-0.919846	-2.614642
H	-4.236359	-6.150800	2.624838
H	6.842655	0.324175	1.115940
H	5.552514	0.533378	5.926696

Calculated energies and coordinates of **INT9**

Electronic energy ... -4308.88226712 Eh
Total Enthalpy ... -4307.35269012 Eh
Final Gibbs free energy ... -4307.55101685 Eh

CARTESIAN COORDINATES (ANGSTROEM)

Si	0.158952	-0.279992	0.200256
Si	-1.972475	2.102535	2.140535
Si	-1.088535	4.171274	2.926697
Si	-3.262915	2.789752	0.266660
Si	-3.378582	1.362006	3.914025
N	1.562896	0.229371	1.146463
N	-0.071326	-0.484635	-3.799728
N	0.179423	-0.412260	-1.430623
N	1.168677	1.590481	3.132031
N	1.150499	-2.065107	-2.916373
N	-1.972925	-1.209654	2.197506
C	-0.640055	0.825706	1.606876
C	-0.995674	-0.669175	1.601523
C	0.402266	-0.950662	-2.585033
C	0.813022	0.967476	2.074964
C	-1.964259	-3.529388	2.955652
C	2.983600	0.214388	1.085737
C	3.619024	1.355925	0.563474
C	-0.522417	1.917590	-3.903829
C	3.727353	-0.887877	1.525585
C	2.382726	1.358225	3.814864
C	-2.436632	-2.532116	2.079754
C	-0.815904	-3.257548	3.878571
H	0.121256	-3.135977	3.322086
H	-0.682229	-4.078939	4.587707
H	-0.966274	-2.330972	4.440101
C	-0.989805	0.595950	-3.994669
C	5.008220	1.427913	0.601006
H	5.504876	2.319260	0.225359
C	0.378639	-1.296525	-4.853165
C	1.982662	-2.842414	-2.048944
C	-2.573507	-4.783508	2.941073
H	-2.204880	-5.549528	3.619355
C	5.121818	-0.792807	1.512370
H	5.707538	-1.636562	1.869458
C	5.759218	0.363592	1.087264
C	3.374623	-2.675229	-2.161499
C	1.128296	-2.280655	-4.305568
C	2.815680	2.435703	-0.100052
H	2.411002	2.064703	-1.049161
H	3.433993	3.311338	-0.316594
H	1.955953	2.754263	0.492929
C	-3.513553	-2.808364	1.215920
C	3.079363	-2.138824	2.039741
H	2.994116	-2.097523	3.131221
H	3.685255	-3.011296	1.783150
H	2.076520	-2.287960	1.629547
C	3.400834	2.326073	3.905390
C	2.476871	0.174917	4.579723
C	1.393469	-3.815335	-1.225328
C	-2.308782	0.276778	-4.358465
C	0.932666	2.218570	-3.583131
H	1.227129	1.545336	-2.768560
C	-3.629516	-5.067662	2.084637
C	3.976717	-1.587677	-3.035440
H	3.319340	-1.432441	-3.898328
C	0.058704	-0.999394	-6.274242
H	0.687873	-1.605025	-6.929528
H	0.243147	0.055560	-6.503376
H	-0.989198	-1.200472	-6.525002
C	3.632896	-0.076917	5.313680
H	3.698881	-0.997884	5.889089
C	-1.437927	2.938330	-4.162583
H	-1.128290	3.974172	-4.082506
C	4.543883	2.034494	4.650317
H	5.338484	2.775717	4.692918
C	-4.089157	-4.077631	1.224735
H	-4.920007	-4.279965	0.552685
C	2.255013	-4.639196	-0.497959
H	1.846035	-5.402342	0.153951
C	-0.116086	-3.986522	-1.146490
H	-0.555712	-2.983740	-1.042971
C	4.187651	-3.522225	-1.408339
H	5.266359	-3.420981	-1.461318
C	3.269019	3.661387	3.238123
H	3.278530	3.575746	2.147742
H	4.093417	4.318352	3.529175
H	2.327557	4.144123	3.516560
C	-3.176180	1.335634	-4.628579
H	-4.203491	1.131085	-4.910635
C	1.802645	-3.448148	-4.930164
H	1.524648	-3.516701	-5.983720
H	1.502844	-4.379375	-4.436641
H	2.895058	-3.388775	-4.865502
C	0.246718	4.862297	1.779007
H	0.664094	5.784699	2.203140
H	-0.166584	5.102879	0.795034
H	1.066992	4.158728	1.640889
C	3.632606	-4.499555	-0.595319
H	4.281344	-5.157599	-0.023496
C	-2.746254	2.650470	-4.526886
H	-3.439187	3.462386	-4.729577
C	-4.040114	-1.726213	0.329536
H	-4.443827	-0.903429	0.929869
H	-4.828241	-2.099113	-0.330461
H	-3.246578	-1.297322	-0.284678
C	-2.797424	-1.162375	-4.401177
H	-1.965025	-1.805330	-4.707967
C	1.170355	3.652146	-3.105527
H	0.984448	4.380043	-3.903174
H	2.215429	3.767232	-2.802897
H	0.542874	3.910817	-2.247179
C	1.309485	-0.761199	4.653892
H	0.468737	-0.281627	5.169306
H	1.570086	-1.673616	5.199183
H	0.939638	-1.046312	3.666285
C	-2.369764	4.122394	-0.741772
H	-2.218107	5.055141	-0.192599
H	-2.957906	4.347879	-1.640474
H	-1.392060	3.756305	-1.073298
C	4.680395	0.835211	5.335882
C	-4.257536	2.864587	4.670430
H	-4.876125	3.395124	3.938923
H	-3.560610	3.582751	5.111898
H	-4.921159	2.512684	5.470650
C	4.023455	-0.268480	-2.257714
H	3.028142	0.017559	-1.906406

H	4.416097	0.539393	-2.886368
H	4.667121	-0.367674	-1.379229
C	-4.916429	3.498691	0.854939
H	-5.517484	2.733261	1.358020
H	-5.492655	3.865362	-0.003912
H	-4.786065	4.332370	1.551405
C	-2.330157	0.589900	5.279093
H	-1.926191	-0.367218	4.938167
H	-2.937187	0.411163	6.175180
H	-1.490179	1.236663	5.554215
C	-2.449695	5.497703	2.910972
H	-2.819434	5.704773	1.902160
H	-2.017012	6.430000	3.297228
H	-3.308043	5.248503	3.540097
C	-3.650717	1.441271	-0.994054
H	-2.751010	0.894161	-1.298227
H	-4.061338	1.913912	-1.894760
H	-4.385771	0.723759	-0.622965
C	5.365938	-1.937259	-3.575881
H	6.121640	-1.927866	-2.783154
H	5.668971	-1.190813	-4.317063
H	5.385269	-2.922711	-4.054091
C	1.844696	1.927779	-4.787999
H	1.849349	0.868162	-5.056824
H	2.875745	2.211782	-4.550716
H	1.524603	2.508456	-5.660998
C	-0.558710	-4.800395	0.066748
H	-0.277655	-5.855766	-0.032528
H	-1.646118	-4.756623	0.164315
H	-0.132579	-4.411441	0.995898
C	-0.438766	4.115937	4.700448
H	-1.238433	3.877509	5.409868
H	-0.036223	5.099081	4.976901
H	0.349317	3.367721	4.816096
C	-0.696451	-4.621389	-2.422065
H	-0.574629	-3.984791	-3.302823
H	-1.768825	-4.797552	-2.285059
H	-0.218125	-5.588016	-2.619742
C	-3.938702	-1.389264	-5.396307
H	-4.871723	-0.926760	-5.056913
H	-4.130259	-2.462375	-5.496792
H	-3.701732	-0.988535	-6.387857
C	-4.775255	0.162582	3.482504
H	-5.390216	0.521326	2.649660
H	-5.432981	0.068454	4.356189
H	-4.396260	-0.832062	3.234504
C	-3.209391	-1.617950	-2.996824
H	-2.371423	-1.543356	-2.296838
H	-3.550164	-2.659453	-3.012881
H	-4.021273	-0.991423	-2.614494
H	-4.092920	-6.049671	2.090937
H	6.843072	0.429580	1.116314
H	5.580499	0.627691	5.906781

Calculated energies and coordinates of **TS9**

Electronic energy ... -4308.88079759 Eh
Total Enthalpy ... -4307.35230732 Eh
Final Gibbs free energy ... -4307.54861107 Eh

CARTESIAN COORDINATES (ANGSTROEM)

Si	0.181670	-0.427196	0.213535
Si	-1.863043	2.280559	2.272433
Si	-0.836211	4.279998	3.056768
Si	-3.163713	3.067084	0.447667
Si	-3.303949	1.579999	4.030988
N	1.524730	0.066437	1.218037
N	-0.040848	-0.625811	-3.758607
N	0.313552	-0.613142	-1.394240
N	1.247910	1.637148	3.045905
N	1.211211	-2.219070	-2.949736
N	-2.311367	-0.921058	1.808738
C	-0.655605	0.909435	1.679769
C	-1.155806	-0.467121	1.490267
C	0.473800	-1.115961	-2.575829
C	0.802478	0.934868	2.072150
C	-2.075510	-3.258919	2.498066
C	2.939134	0.139752	1.062193
C	3.480915	1.298535	0.480954
C	-0.539806	1.766092	-3.861046
C	3.764313	-0.913979	1.477718
C	2.462584	1.370054	3.713051
C	-2.724956	-2.259028	1.733468
C	-0.828889	-2.956963	3.273566
H	0.030326	-2.770280	2.619758
H	-0.572683	-3.789079	3.935676
H	-0.959365	-2.058118	3.882675
C	-0.990759	0.436990	-3.891939
C	4.865022	1.450587	0.452295
H	5.290964	2.358933	0.032959
C	0.364100	-1.424925	-4.840178
C	2.009255	-3.035273	-2.089148
C	-2.634958	-4.533068	2.577715
H	-2.131833	-5.285005	3.181750
C	5.147618	-0.736537	1.407992
H	5.797255	-1.536087	1.755105
C	5.696780	0.448399	0.936129
C	3.404328	-2.885606	-2.152330
C	1.141247	-2.413374	-4.338991
C	2.581364	2.313993	-0.157300
H	2.097166	1.878682	-1.039170
H	3.144006	3.193759	-0.481424
H	1.774784	2.643591	0.502298
C	-3.937325	-2.578976	1.082098
C	3.196134	-2.195640	2.009694
H	2.863327	-2.072035	3.045122
H	3.952681	-2.984130	1.989325
H	2.338895	-2.537424	1.420372
C	3.519779	2.300327	3.763897
C	2.524594	0.207353	4.514417
C	1.373452	-4.002275	-1.293863
C	-2.328687	0.089477	-4.132056
C	0.931860	2.089977	-3.665474
H	1.314302	1.402961	-2.900181
C	-3.802064	-4.858866	1.900627
C	4.045238	-1.812535	-3.016699
H	3.417574	-1.663937	-3.902700
C	-0.074222	-1.145999	-6.232389
H	0.539487	-1.710998	-6.936895
H	0.019093	-0.082026	-6.472813
H	-1.122804	-1.420921	-6.395793
C	3.681403	-0.067838	5.238585
H	3.718847	-0.972102	5.842117
C	-1.490845	2.767317	-4.059311
H	-1.193453	3.809340	-4.022420
C	4.663568	1.983090	4.496464
H	5.487225	2.693018	4.503962
C	-4.439266	-3.877582	1.152356
H	-5.366961	-4.107042	0.632762
C	2.193937	-4.832049	-0.527269
H	1.749086	-5.589507	0.108057
C	-0.140285	-4.156983	-1.288940
H	-0.576381	-3.150994	-1.209096
C	4.176090	-3.739878	-1.363646
H	5.257435	-3.653999	-1.375530
C	3.428709	3.627030	3.074986
H	3.348453	3.522825	1.989428
H	4.314576	4.231367	3.289231
H	2.545888	4.176934	3.414422
C	-3.235976	1.127999	-4.343734
H	-4.281435	0.900317	-4.525737
C	1.805981	-3.562022	-5.007447
H	1.483967	-3.621709	-6.048945
H	1.546782	-4.505482	-4.514928
H	2.899002	-3.481628	-4.990417
C	0.449191	4.979777	1.855759
H	0.919554	5.869223	2.294862
H	-0.026722	5.283338	0.918858
H	1.238037	4.268068	1.619207
C	3.576238	-4.702660	-0.564531
H	4.193513	-5.360409	0.041126

C	-2.820765	2.450694	-4.305315
H	-3.542617	3.247633	-4.460931
C	-4.713748	-1.517878	0.367581
H	-5.229898	-0.870041	1.086244
H	-5.465291	-1.958980	-0.294041
H	-4.057212	-0.876636	-0.215441
C	-2.792949	-1.355466	-4.093725
H	-1.936741	-2.004676	-4.308909
C	1.182024	3.517058	-3.176192
H	0.921981	4.257202	-3.941017
H	2.245259	3.645054	-2.952212
H	0.616523	3.746001	-2.267663
C	1.324319	-0.680800	4.634185
H	0.476733	-0.124603	5.050407
H	1.529103	-1.538096	5.282622
H	0.996115	-1.057780	3.664057
C	-2.207355	4.312221	-0.614950
H	-2.002473	5.250431	-0.091891
H	-2.793920	4.546753	-1.512432
H	-1.252302	3.887931	-0.943991
C	4.765341	0.799872	5.215076
C	-4.022273	3.094697	4.919751
H	-4.627943	3.713337	4.248854
H	-3.249907	3.729834	5.363404
H	-4.675941	2.749076	5.730743
C	4.078988	-0.478594	-2.262379
H	3.078932	-0.179096	-1.937105
H	4.489859	0.314042	-2.898763
H	4.703051	-0.561457	-1.368481
C	-4.724815	3.927986	1.087648
H	-5.377116	3.225287	1.617384
H	-5.293841	4.337925	0.243514
H	-4.499081	4.751789	1.770787
C	-2.309538	0.604953	5.304258
H	-1.952396	-0.332031	4.866309
H	-2.929122	0.360540	6.175798
H	-1.440581	1.175747	5.649508
C	-2.136451	5.664356	3.138458
H	-2.505089	5.933346	2.143448
H	-1.653436	6.556470	3.558483
H	-2.998021	5.428643	3.768097
C	-3.743485	1.736005	-0.754511
H	-2.924416	1.093353	-1.097482
H	-4.179460	2.215949	-1.638993
H	-4.510786	1.110694	-0.293082
C	5.447417	-2.185201	-3.504898
H	6.174247	-2.177481	-2.685558
H	5.787068	-1.450719	-4.242111
H	5.469749	-3.175936	-3.971911
C	1.737092	1.840089	-4.952022
H	1.728824	0.786419	-5.242951
H	2.781985	2.132348	-4.800248
H	1.333926	2.434569	-5.780152
C	-0.658515	-4.964319	-0.102029
H	-0.400309	-6.026412	-0.193640
H	-1.748196	-4.889737	-0.055449
H	-0.269986	-4.591269	0.849123
C	-0.111915	4.143977	4.797598
H	-0.895920	3.922060	5.530012
H	0.347187	5.097850	5.087830
H	0.642078	3.355896	4.862425
C	-0.662753	-4.790436	-2.590532
H	-0.506757	-4.151696	-3.463890
H	-1.739262	-4.971213	-2.500277
H	-0.173416	-5.754968	-2.770363
C	-3.881171	-1.670851	-5.125102
H	-4.832162	-1.191508	-4.870154
H	-4.062367	-2.750273	-5.152719
H	-3.598780	-1.343310	-6.131417
C	-4.800846	0.548549	3.519216
H	-5.327923	0.990570	2.666865
H	-5.506641	0.494118	4.357661
H	-4.499305	-0.465684	3.247098
C	-3.277128	-1.695877	-2.679296
H	-2.504448	-1.487881	-1.930759
H	-3.555406	-2.753096	-2.600752
H	-4.154394	-1.091573	-2.429609
H	-4.217189	-5.860234	1.966624
H	6.774929	0.579750	0.920397
H	5.666346	0.573356	5.777278

Calculated energies and coordinates of **INT10**

Electronic energy ... -4308.90048945 Eh
Total Enthalpy ... -4307.37099428 Eh
Final Gibbs free energy ... -4307.56954247 Eh

CARTESIAN COORDINATES (ANGSTROEM)

Si	0.002341	-0.522433	0.029786
Si	-1.874342	2.606335	2.629401
Si	-0.903144	4.776504	2.551220
Si	-4.089409	2.791219	1.781607
Si	-2.049214	1.988043	4.914669
N	1.200049	0.185923	1.041381
N	0.222675	-0.911412	-3.719882
N	0.438210	-1.244427	-1.345202
N	1.135686	1.960305	2.741858
N	1.507773	-2.542809	-3.031683
N	-2.729989	0.136088	0.574274
C	-0.857921	1.364869	1.591765
C	-1.461333	0.370297	0.801142
C	0.682883	-1.539070	-2.581770
C	0.565248	1.223765	1.842049
C	-2.703933	-2.279137	1.077016
C	2.569520	-0.193157	1.178330
C	3.582162	0.742019	0.911975
C	-0.751904	1.313495	-3.384597
C	2.888248	-1.506566	1.564889
C	2.319773	1.734322	3.443393
C	-3.188644	-1.140048	0.363141
C	-1.895918	-2.197480	2.354049
H	-1.142759	-2.992047	2.390759
H	-2.566724	-2.358072	3.207679
H	-1.405757	-1.238675	2.516554
C	-0.901861	-0.022308	-3.784610
C	4.897672	0.407385	1.220691
H	5.677967	1.148009	1.066816
C	0.789976	-1.508482	-4.858994
C	2.328581	-3.374643	-2.204489
C	-3.231490	-3.540569	0.772028
H	-2.862327	-4.398607	1.331822
C	4.225222	-1.818341	1.814660
H	4.477418	-2.826145	2.134363
C	5.220029	-0.859013	1.690375
C	3.709605	-3.117144	-2.179896
C	1.572491	-2.529614	-4.434033
C	3.270775	2.029975	0.213367
H	2.938961	1.811103	-0.809584
H	4.160493	2.660890	0.149550
H	2.472727	2.597208	0.695533
C	-4.298152	-1.310179	-0.517513
C	1.838688	-2.573258	1.699177
H	0.957013	-2.217217	2.236684
H	2.235083	-3.440605	2.234814
H	1.512950	-2.927448	0.713068
C	3.292374	2.761169	3.528718
C	2.490675	0.565057	4.229723
C	1.732104	-4.428308	-1.494302
C	-2.107587	-0.533485	-4.296533
C	0.578948	1.849524	-2.887250
H	1.060381	1.059237	-2.297086
C	-4.265924	-3.704276	-0.132809
C	4.305541	-1.917407	-2.896240
H	3.725932	-1.727992	-3.806051
C	0.516241	-1.004611	-6.229301
H	-0.490900	-1.259177	-6.577281
H	1.237721	-1.427224	-6.931415
H	0.601483	0.086465	-6.267540
C	3.663354	0.398084	4.960865
H	3.787259	-0.514515	5.540468
C	-1.864764	2.147959	-3.505047

H	-1.802589	3.180771	-3.182061
C	4.443952	2.551295	4.284336
H	5.189528	3.343197	4.321229
C	-4.816808	-2.574594	-0.740147
H	-5.661864	-2.686650	-1.416096
C	2.575715	-5.247311	-0.742202
H	2.158039	-6.069450	-0.171297
C	0.233385	-4.677251	-1.557089
H	-0.270224	-3.703438	-1.519353
C	4.505813	-3.966046	-1.410966
H	5.575607	-3.796119	-1.354835
C	3.144640	4.081406	2.820035
H	3.884339	4.190731	2.018307
H	3.308010	4.905851	3.522908
H	2.154147	4.200022	2.385408
C	-3.177599	0.351633	-4.425568
H	-4.126027	-0.007118	-4.812365
C	2.346186	-3.543206	-5.196999
H	2.127271	-3.451897	-6.262573
H	2.076221	-4.556497	-4.879310
H	3.428688	-3.443951	-5.058847
C	0.075988	5.003268	0.945729
H	0.666154	5.927818	0.979112
H	-0.622476	5.081244	0.104476
H	0.750229	4.170068	0.737026
C	3.944922	-5.020338	-0.703988
H	4.581813	-5.669907	-0.110021
C	-3.058404	1.676371	-4.031986
H	-3.910477	2.344890	-4.115472
C	-4.875732	-0.112669	-1.206235
H	-5.231265	0.624166	-0.479797
H	-5.710435	-0.400062	-1.853213
H	-4.119077	0.396470	-1.812496
C	-2.293399	-2.005852	-4.620482
H	-1.314105	-2.455331	-4.815290
C	0.420183	3.063925	-1.974546
H	0.050903	3.939951	-2.518592
H	1.388085	3.341772	-1.549598
H	-0.265201	2.856461	-1.145489
C	1.409332	-0.470723	4.384730
H	0.893746	-0.331921	5.343396
H	1.837134	-1.478059	4.388383
H	0.647022	-0.420346	3.607394
C	-3.995810	3.161231	-0.075802
H	-3.539449	4.149672	-0.213859
H	-4.992909	3.190463	-0.533543
H	-3.389444	2.421659	-0.605011
C	4.653004	1.371248	4.982098
C	-3.424430	3.019965	5.714036
H	-3.259805	4.094108	5.570125
H	-3.447195	2.829861	6.794609
H	-4.411134	2.773188	5.308727
C	4.167983	-0.669952	-2.016076
H	3.124826	-0.491223	-1.738474
H	4.545801	0.215023	-2.541735
H	4.734399	-0.792114	-1.089189
C	-5.013149	4.278703	2.529825
H	-4.880363	4.370689	3.611894
H	-6.085785	4.136198	2.344174
H	-4.723165	5.228258	2.074866
C	-0.500223	2.265540	5.965557
H	0.424591	2.018836	5.437743
H	-0.558416	1.645704	6.869812
H	-0.427669	3.309677	6.284303
C	-2.166452	6.189885	2.523359
H	-2.753505	6.202425	1.599799
H	-1.618154	7.139411	2.581629
H	-2.862274	6.155374	3.367313
C	-5.226583	1.346309	2.242024
H	-4.809546	0.366198	2.004735
H	-6.191550	1.451146	1.728722
H	-5.433219	1.384969	3.318943
C	5.763200	-2.119221	-3.316939
H	6.435738	-2.130152	-2.452629
H	6.078515	-1.289795	-3.958020
H	5.903960	-3.053457	-3.871478
C	1.510658	2.173387	-4.065920
H	1.753303	1.277032	-4.645219
H	2.450528	2.599875	-3.697971
H	1.042829	2.903809	-4.735834
C	-0.298472	-5.492832	-0.377797
H	0.048374	-6.532007	-0.410271
H	-1.391171	-5.505554	-0.418401
H	-0.004927	-5.057212	0.583530
C	0.153742	5.143905	4.075942
H	-0.502256	5.325039	4.935389
H	0.742420	6.055910	3.914833
H	0.834201	4.329760	4.333246
C	-0.158563	-5.359668	-2.878243
H	0.048973	-4.724380	-3.744522
H	-1.230738	-5.581816	-2.878309
H	0.387084	-6.302531	-3.000076
C	-3.161932	-2.246413	-5.861047
H	-4.213082	-2.004863	-5.672232
H	-3.120810	-3.303822	-6.142107
H	-2.829771	-1.650158	-6.717782
C	-2.540829	0.168576	5.078686
H	-3.395667	-0.078882	4.440504
H	-2.812656	-0.052396	6.118727
H	-1.712217	-0.491078	4.802460
C	-2.888684	-2.724192	-3.404164
H	-2.274044	-2.590727	-2.507216
H	-2.991740	-3.797300	-3.600339
H	-3.877971	-2.321351	-3.173692
H	-4.676094	-4.690757	-0.328910
H	6.249044	-1.104283	1.936292
H	5.562598	1.220759	5.555659

Calculated energies and coordinates of **TS10**

Electronic energy ... -4308.90003583 Eh
Total Enthalpy ... -4307.37128988 Eh
Final Gibbs free energy ... -4307.56703362 Eh

CARTESIAN COORDINATES (ANGSTROEM)

Si	-0.067979	-0.585716	0.051604
Si	-1.878175	2.646456	2.630222
Si	-0.906946	4.816171	2.547805
Si	-4.109242	2.825391	1.810067
Si	-2.029412	2.044095	4.927133
N	1.136205	0.138062	1.065222
N	0.208882	-0.926401	-3.704903
N	0.445043	-1.261038	-1.329363
N	1.121096	1.933872	2.745388
N	1.502133	-2.557565	-3.026363
N	-2.739653	0.248537	0.441439
C	-0.874521	1.414931	1.567094
C	-1.475018	0.474957	0.732616
C	0.672889	-1.557517	-2.566168
C	0.542872	1.212143	1.838760
C	-2.516244	-2.127936	1.018299
C	2.505524	-0.244176	1.192157
C	3.519572	0.687494	0.915748
C	-0.773239	1.290471	-3.344249
C	2.824794	-1.556266	1.582550
C	2.301998	1.695370	3.449794
C	-3.135167	-1.044688	0.300294
C	-1.917183	-1.955580	2.399868
H	-1.211588	-2.762553	2.624240
H	-2.728742	-2.032620	3.135286
H	-1.427651	-0.997960	2.573123
C	-0.916998	-0.040040	-3.765585
C	4.835811	0.352054	1.221608
H	5.617366	1.089828	1.058925
C	0.780314	-1.513102	-4.847472
C	2.339083	-3.384962	-2.211095
C	-2.893722	-3.443485	0.690196
H	-2.426265	-4.261963	1.234038
C	4.162108	-1.868735	1.828911
H	4.413877	-2.875711	2.151479
C	5.158126	-0.911693	1.698041

C	3.717291	-3.109617	-2.196143
C	1.567066	-2.533066	-4.428716
C	3.210006	1.971188	0.207187
H	2.861133	1.744390	-0.807989
H	4.104815	2.592889	0.122810
H	2.424837	2.553275	0.693231
C	-4.257907	-1.325485	-0.537391
C	1.776103	-2.622876	1.724585
H	0.895120	-2.264302	2.262082
H	2.175545	-3.486702	2.263857
H	1.447396	-2.980904	0.740778
C	3.285462	2.712687	3.526466
C	2.461691	0.530794	4.244075
C	1.764682	-4.454896	-1.505836
C	-2.118442	-0.545438	-4.293518
C	0.559146	1.826119	-2.849597
H	1.029212	1.044144	-2.239556
C	-3.924645	-3.706137	-0.188958
C	4.293222	-1.900665	-2.913610
H	3.705219	-1.716470	-3.818987
C	0.512154	-0.997794	-6.215215
H	-0.492228	-1.250260	-6.572326
H	1.238462	-1.412730	-6.916981
H	0.595585	0.093748	-6.243565
C	3.632995	0.357785	4.976425
H	3.748351	-0.551643	5.562791
C	-1.891770	2.119546	-3.444748
H	-1.835284	3.146361	-3.102962
C	4.435458	2.497596	4.282947
H	5.189350	3.281970	4.313083
C	-4.637340	-2.636096	-0.749090
H	-5.491750	-2.840093	-1.391063
C	2.626908	-5.263055	-0.762660
H	2.228354	-6.096435	-0.195076
C	0.269495	-4.730872	-1.560836
H	-0.252032	-3.771286	-1.446359
C	4.532077	-3.949245	-1.436636
H	5.599611	-3.763619	-1.388112
C	3.148042	4.028355	2.808144
H	3.870917	4.115635	1.988793
H	3.342899	4.856325	3.498587
H	2.150337	4.159631	2.393837
C	-3.194376	0.335487	-4.402568
H	-4.139616	-0.019822	-4.800504
C	2.340659	-3.540506	-5.200015
H	2.128109	-3.434079	-6.265540
H	2.062782	-4.556279	-4.897152
H	3.422833	-3.449642	-5.053987
C	0.072640	5.031016	0.941879
H	0.671788	5.949781	0.973353
H	-0.622573	5.111319	0.098215
H	0.739722	4.190336	0.738845
C	3.992334	-5.013906	-0.729794
H	4.642524	-5.655727	-0.141887
C	-3.084018	1.651265	-3.977941
H	-3.940043	2.316490	-4.046730
C	-4.978946	-0.189165	-1.193505
H	-5.332220	0.533841	-0.451588
H	-5.834312	-0.551926	-1.771842
H	-4.306415	0.360783	-1.860828
C	-2.294458	-2.009513	-4.659641
H	-1.312112	-2.448666	-4.860436
C	0.407622	3.061963	-1.965705
H	0.039916	3.926135	-2.529750
H	1.378063	3.345949	-1.550664
H	-0.274727	2.874752	-1.129798
C	1.369292	-0.491499	4.407934
H	0.852997	-0.336803	5.363987
H	1.786056	-1.503409	4.422969
H	0.610869	-0.441310	3.627275
C	-4.098417	3.199794	-0.047301
H	-3.609687	4.167412	-0.217450
H	-5.122085	3.273515	-0.437138
H	-3.562115	2.432034	-0.610042
C	4.632663	1.320683	4.989511
C	-3.382985	3.102446	5.729601
H	-3.219732	4.173205	5.562342
H	-3.382972	2.931961	6.813671
H	-4.378701	2.849714	5.350924
C	4.144392	-0.658656	-2.028466
H	3.100399	-0.497375	-1.743950
H	4.506530	0.233957	-2.552294
H	4.717470	-0.777479	-1.105247
C	-5.031408	4.296594	2.589128
H	-4.884780	4.382714	3.669367
H	-6.105390	4.149034	2.415445
H	-4.753166	5.250764	2.135827
C	-0.466832	2.315651	5.958619
H	0.450471	2.048279	5.428052
H	-0.527988	1.707464	6.870560
H	-0.377583	3.362154	6.264433
C	-2.174951	6.224787	2.524411
H	-2.774477	6.227467	1.608767
H	-1.632256	7.178137	2.570074
H	-2.859678	6.191180	3.377560
C	-5.204896	1.351926	2.282415
H	-4.768995	0.384008	2.027724
H	-6.181017	1.435791	1.786877
H	-5.391997	1.374234	3.363137
C	5.750510	-2.082454	-3.344845
H	6.429487	-2.086102	-2.485497
H	6.049864	-1.247581	-3.986517
H	5.899852	-3.013567	-3.902471
C	1.501659	2.117765	-4.028534
H	1.738839	1.209370	-4.590604
H	2.444035	2.539547	-3.661427
H	1.047078	2.842233	-4.713928
C	-0.220624	-5.638219	-0.431530
H	0.161977	-6.660185	-0.534628
H	-1.312431	-5.689587	-0.464525
H	0.068229	-5.254962	0.553065
C	0.160834	5.183186	4.064739
H	-0.486651	5.370734	4.929155
H	0.753721	6.091004	3.895455
H	0.838822	4.366040	4.319351
C	-0.140752	-5.334533	-2.914828
H	0.024261	-4.640181	-3.743527
H	-1.206675	-5.584667	-2.900931
H	0.423167	-6.253659	-3.112747
C	-3.156632	-2.221469	-5.909846
H	-4.210239	-1.992733	-5.718378
H	-3.106873	-3.270535	-6.219507
H	-2.826219	-1.599559	-6.748732
C	-2.549956	0.238783	5.154052
H	-3.422524	-0.014041	4.543130
H	-2.808554	0.065634	6.206577
H	-1.741313	-0.450633	4.892357
C	-2.888189	-2.766093	-3.467665
H	-2.276421	-2.652566	-2.566489
H	-2.980668	-3.834446	-3.693380
H	-3.881634	-2.376944	-3.231425
H	-4.225580	-4.729470	-0.395541
H	6.187394	-1.156941	1.943246
H	5.540513	1.165694	5.564772

Calculated energies and coordinates of **5a**

Electronic energy ... -4308.92043030 Eh
Total Enthalpy ... -4307.39031435 Eh
Final Gibbs free energy ... -4307.58456986 Eh

CARTESIAN COORDINATES (ANGSTROEM)

Si	6.627890	13.355209	7.555001
Si	5.154598	17.074622	9.755136
Si	5.958146	19.201290	9.042001
Si	2.837364	17.163444	9.182684
Si	5.196268	16.874993	12.130126
N	7.857287	13.987947	8.661561
N	6.977638	13.379687	3.843968
N	7.265854	12.735314	6.163400
N	7.899330	15.738267	10.385362

N	8.374700	11.758960	4.302572
N	4.227860	14.572825	7.562447
C	6.063359	15.644479	8.865569
C	5.535566	14.823820	7.914119
C	7.499772	12.651577	4.903694
C	7.388433	15.156880	9.350764
C	5.044481	12.351207	8.200465
C	9.203539	13.540046	8.776513
C	10.263858	14.453662	8.620916
C	6.065517	15.567840	4.442858
C	9.480096	12.176956	9.006159
C	9.003919	15.378000	11.165986
C	3.987671	13.279488	7.599648
C	4.938210	12.472504	9.750975
H	5.632341	11.789769	10.248774
H	3.923371	12.194682	10.057756
H	5.132338	13.492061	10.091391
C	5.867989	14.275949	3.926643
C	11.562554	14.033427	8.901727
H	12.372177	14.755441	8.827076
C	7.533583	12.946744	2.625140
C	9.298737	10.924054	5.004544
C	4.858650	10.924625	7.864638
H	5.640878	10.227918	8.157998
C	10.799208	11.789534	9.234711
H	11.008388	10.740596	9.428561
C	11.834030	12.715023	9.233329
C	10.647930	11.315716	5.033126
C	8.393793	11.941626	2.909828
C	10.031996	15.830606	8.077310
H	9.258825	15.808544	7.303054
H	10.953963	16.223368	7.638634
H	9.693347	16.533279	8.841225
C	2.732013	12.732695	7.149967
C	8.406131	11.127014	8.990847
H	7.552439	11.396858	9.617031
H	8.802080	10.173328	9.351486
H	8.041282	10.963478	7.969183
C	9.938111	16.401059	11.473511
C	9.159797	14.114745	11.787447
C	8.837033	9.740612	5.603316
C	4.626033	13.831725	3.440697
C	7.441359	16.033627	4.895399
H	7.856675	15.244155	5.538043
C	3.697284	10.474582	7.348875
C	11.103718	12.634104	4.430814
H	10.451920	12.875047	3.584077
C	7.194708	13.596388	1.331799
H	6.164009	13.401005	1.014193
H	7.865695	13.236751	0.549065
H	7.305305	14.684103	1.401482
C	10.318201	13.850142	12.514246
H	10.439122	12.861477	12.951973
C	4.954893	16.409938	4.497898
H	5.048278	17.405105	4.916625
C	11.078476	16.092842	12.211650
H	11.797595	16.883972	12.413410
C	2.614763	11.377550	7.059064
H	1.677949	10.955891	6.699862
C	9.780610	8.940553	6.249917
H	9.468971	8.019982	6.731022
C	7.368032	9.349166	5.543536
H	6.783840	10.244624	5.796808
C	11.549560	10.479517	5.692071
H	12.597686	10.754239	5.746720
C	9.714806	17.826716	11.052003
H	10.267216	18.093008	10.142444
H	10.054044	18.504394	11.842810
H	8.657339	18.008195	10.857126
C	3.554312	14.723464	3.497969
H	2.579128	14.418072	3.131885
C	9.215463	11.083451	2.017116
H	10.291814	11.224100	2.169229
H	8.985923	11.305292	0.973057
H	9.006040	10.022174	2.193842
C	7.800143	19.260752	8.589036
H	8.372965	19.805897	9.345711
H	7.928347	19.777548	7.631108
H	8.234413	18.262456	8.497596
C	11.119961	9.305620	6.292785
H	11.835663	8.668384	6.804817
C	3.716085	15.994222	4.029392
H	2.867071	16.669154	4.089611
C	1.641568	13.664057	6.722003
H	1.395911	14.378842	7.513213
H	0.740377	13.110020	6.444585
H	1.973532	14.260080	5.863145
C	4.429792	12.414958	2.925318
H	5.384820	12.049448	2.534382
C	7.401123	17.317699	5.720473
H	7.055280	18.171986	5.126662
H	8.407787	17.559940	6.072625
H	6.756367	17.209759	6.597886
C	8.077546	13.073901	11.796568
H	7.514847	13.136486	12.736822
H	8.508695	12.069773	11.741645
H	7.367595	13.191525	10.981157
C	2.409748	17.253348	7.341298
H	2.721447	18.209367	6.909299
H	1.319321	17.180916	7.229960
H	2.874626	16.440832	6.781233
C	11.297758	14.814258	12.702395
C	3.773508	17.845194	12.925345
H	3.831269	18.918231	12.713804
H	3.841807	17.720434	14.013650
H	2.788451	17.486531	12.612981
C	10.937889	13.763367	5.454070
H	9.910793	13.818975	5.826741
H	11.199941	14.728771	5.005457
H	11.584792	13.591972	6.319470
C	2.075180	18.753334	9.893017
H	2.211171	18.871758	10.969775
H	0.995832	18.735800	9.692786
H	2.478705	19.645854	9.403656
C	6.746015	17.473805	13.025217
H	7.608214	16.830561	12.833636
H	6.545707	17.465038	14.104503
H	7.018697	18.497912	12.751190
C	5.024655	19.808800	7.507210
H	5.078909	19.087795	6.685305
H	5.477255	20.747129	7.161706
H	3.968712	20.004598	7.713167
C	1.912588	15.713745	9.981755
H	2.350112	14.750482	9.700696
H	0.857377	15.714665	9.679881
H	1.942390	15.789214	11.074252
C	12.540862	12.595595	3.902797
H	13.270624	12.544656	4.717932
H	12.751033	13.511130	3.340398
H	12.710616	11.739531	3.240566
C	8.403170	16.219267	3.708428
H	8.627319	15.276103	3.203734
H	9.351958	16.635579	4.064193
H	7.979706	16.918211	2.977613
C	6.990212	8.260666	6.550321
H	7.426979	7.291516	6.282176
H	5.902627	8.136032	6.558557
H	7.310779	8.509216	7.567538
C	5.623507	20.464972	10.410559
H	4.562488	20.492927	10.681030
H	5.910644	21.469293	10.074722
H	6.194315	20.237789	11.316947
C	6.950610	8.899087	4.132964
H	7.018411	9.711576	3.404669
H	5.911127	8.553550	4.145987
H	7.580074	8.068634	3.792109
C	3.406393	12.319702	1.789524
H	2.385843	12.496960	2.145598
H	3.426118	11.313368	1.358328
H	3.614540	13.039518	0.990454
C	4.918264	15.060708	12.580912
H	3.974640	14.681829	12.173285

H	4.888647	14.944705	13.671643
H	5.729962	14.437674	12.197947
C	4.041199	11.484151	4.078304
H	4.763344	11.535477	4.899608
H	3.984218	10.445183	3.733057
H	3.065310	11.768255	4.482935
H	3.548075	9.412447	7.170940
H	12.850174	12.402354	9.455257
H	12.198487	14.583456	13.263391

Calculated energies and coordinates of **INT11**

Electronic energy ... -4711.93037134 Eh
Total Enthalpy ... -4710.23544370 Eh
Final Gibbs free energy ... -4710.45265921 Eh

CARTESIAN COORDINATES (ANGSTROEM)

Si	0.390085	-0.274827	-0.106312
Si	0.052289	5.589841	-1.676702
Si	-1.366158	3.872985	-0.794919
Si	-3.138484	3.499000	-2.356250
Si	-2.363044	4.974685	1.083388
N	-1.347925	0.826836	-2.143809
N	0.775659	3.292483	1.356769
N	1.076278	0.910112	0.978930
N	-0.265359	-1.668327	0.469112
N	2.573905	-0.988592	-2.136639
N	0.197115	-3.774672	1.577711
N	-1.789670	-3.501892	0.691784
C	-0.605862	1.047955	-1.078640
C	-0.573939	-2.851369	0.868253
C	-0.938868	0.159291	-3.263195
C	0.296023	0.470375	-3.908730
C	1.823213	-0.813315	-1.255435
C	1.978870	3.555350	2.003082
C	-0.525678	2.220388	-0.319356
C	3.382440	-1.201578	-3.217186
C	1.282275	6.349119	-0.444049
H	2.191005	6.654798	-0.977771
H	1.574018	5.674727	0.363930
H	0.851722	7.243692	0.016713
C	0.465189	2.202605	0.731942
C	-1.850894	-0.701947	-3.931601
C	1.687991	0.678158	2.258507
C	3.176901	3.581470	1.244737
C	-2.583146	3.116191	-4.126376
H	-2.096188	4.002701	-4.552026
H	-3.467545	2.909806	-4.743510
H	-1.901446	2.269728	-4.216287
C	0.633595	-0.178310	-5.093673
H	1.572743	0.077189	-5.582072
C	-1.069258	7.013415	-2.240789
H	-1.785581	7.315202	-1.469280
H	-1.631191	6.753432	-3.143423
H	-0.445164	7.885252	-2.475315
C	-0.224965	-1.098807	-5.689537
H	0.052137	-1.588333	-6.618927
C	-1.472434	-1.329104	-5.112189
H	-2.178403	-1.998849	-5.600197
C	2.679226	-3.822359	0.097193
H	1.878199	-3.251457	-0.392834
C	1.055123	5.135158	-3.222522
H	1.540373	6.049317	-3.589066
H	0.424955	4.745470	-4.028727
H	1.841228	4.400913	-3.020826
C	4.459681	-0.318628	-3.444570
C	1.110810	1.645270	-3.449201
H	0.464675	2.527842	-3.400288
H	1.921410	1.851628	-4.155746
H	1.535959	1.550883	-2.445538
C	4.365783	3.960345	1.861599
H	5.280204	3.982457	1.271525
C	3.065053	0.436370	2.392097
C	1.994707	4.001022	3.342939
C	0.868640	0.738413	3.401747
C	-3.219840	-0.881233	-3.362324
H	-3.180431	-1.109098	-2.293215
H	-3.763910	-1.677092	-3.878545
H	-3.793982	0.048629	-3.457199
C	2.628786	-3.483748	1.578594
C	3.149242	3.261781	-0.222605
H	2.863691	2.221644	-0.413686
H	4.131119	3.437947	-0.673712
H	2.412002	3.883105	-0.742147
C	3.102476	-2.304103	-4.053613
C	3.994386	0.297239	1.223197
H	4.545201	1.230933	1.060660
H	3.459067	0.058403	0.305153
H	4.723495	-0.496540	1.416693
C	-0.536001	-4.951835	1.809217
C	-4.232730	5.036219	-2.619799
H	-4.932051	5.201565	-1.797714
H	-4.825602	4.861297	-3.527230
H	-3.670102	5.961068	-2.771889
C	2.401836	-5.316317	-0.147775
H	3.124792	-5.932812	0.398830
H	2.499735	-5.546156	-1.215153
H	1.395115	-5.604670	0.163093
C	3.209034	4.365998	3.920935
H	3.214846	4.690686	4.959482
C	4.396364	4.334017	3.200768
H	5.330805	4.631057	3.667479
C	-4.330819	2.209211	-1.644730
H	-3.824684	1.268999	-1.415279
H	-5.149768	2.007867	-2.347747
H	-4.776950	2.601149	-0.721874
C	-0.612240	0.903458	3.258951
H	-1.109313	0.872077	4.232823
H	-1.030605	0.115972	2.622707
H	-0.862972	1.850278	2.776299
C	1.411604	-3.486593	2.282379
C	4.730177	0.826592	-2.520236
H	4.915464	0.481032	-1.497019
H	3.876458	1.510840	-2.475500
H	5.603358	1.392254	-2.852127
C	3.612222	0.361887	3.673546
H	4.684483	0.209296	3.772162
C	4.008871	-3.448371	-0.560397
H	4.278432	-2.402552	-0.382936
H	3.951587	-3.606929	-1.641528
H	4.823629	-4.080721	-0.190123
C	-1.765196	-4.773536	1.279918
C	1.455015	0.664542	4.662500
H	0.820801	0.744155	5.542168
C	-2.248745	-4.745182	-1.848302
H	-1.796360	-5.339385	-1.044639
C	2.825489	0.502465	4.804734
H	3.275196	0.468312	5.793231
C	5.251648	-0.558751	-4.561578
H	6.084512	0.107898	-4.765774
C	3.792953	-3.226327	2.301771
H	4.751636	-3.209298	1.796009
C	-3.001971	3.766284	2.392918
H	-3.619680	2.986338	1.935585
H	-3.626271	4.316395	3.108560
H	-2.202679	3.278591	2.953775
C	-2.935672	-3.071896	-0.052568
C	-3.249132	-3.783329	-1.229674
C	1.927462	-3.187725	-3.797782
H	0.998416	-2.659609	-4.055204
H	1.854870	-3.482680	-2.746658
H	1.982569	-4.091482	-4.409374
C	3.934471	-2.494485	-5.151188
H	3.738003	-3.330167	-5.816766
C	0.025500	-6.140928	2.512906
H	1.089300	-6.001853	2.720278
H	-0.083178	-7.041768	1.900006
H	-0.477213	-6.324527	3.468813
C	-3.910454	5.976514	0.630412
H	-3.765327	6.657602	-0.212751
H	-4.183176	6.580768	1.505769

H	-4.761181	5.328006	0.398944
C	-1.104978	-3.969699	-2.515956
H	-1.471136	-3.384711	-3.365359
H	-0.345296	-4.670809	-2.879688
H	-0.629885	-3.267995	-1.822686
C	0.729541	4.076140	4.150002
H	0.762787	4.924980	4.840512
H	0.586913	3.167281	4.745844
H	-0.143284	4.185106	3.503891
C	-1.218152	6.240006	1.903436
H	-1.614893	6.505229	2.892120
H	-1.183725	7.158342	1.306403
H	-0.195619	5.876858	2.024792
C	1.350032	-3.315838	3.677856
C	-2.958290	-5.656234	1.353270
H	-3.273330	-6.042930	0.377646
H	-3.815465	-5.114934	1.770279
H	-2.738715	-6.506126	2.004120
C	-0.443107	-2.148260	5.042234
H	0.290326	-1.694743	5.716139
H	-1.372369	-2.309467	5.601023
H	-0.638433	-1.439449	4.236956
C	4.994579	-1.632341	-5.408686
H	5.628221	-1.799756	-6.274151
C	-4.487320	-3.540445	-1.817516
H	-4.774072	-4.078407	-2.714422
C	-3.791263	-2.110309	0.496069
C	3.748047	-2.988152	3.669097
H	4.664057	-2.767514	4.209266
C	0.071208	-3.477608	4.487156
H	-0.711307	-3.869107	3.829909
C	-4.349844	-1.948533	2.934744
H	-5.415169	-1.789450	2.732864
H	-4.103144	-1.446071	3.876677
H	-4.188955	-3.023939	3.069879
C	-5.005271	-1.875501	-0.157684
H	-5.690752	-1.132803	0.238973
C	-2.867625	-5.727401	-2.843890
H	-3.713250	-6.273605	-2.411830
H	-2.114182	-6.458488	-3.154133
H	-3.214868	-5.217267	-3.749362
C	2.543045	-3.041843	4.348008
H	2.524355	-2.867893	5.419642
C	-5.360158	-2.598693	-1.283540
H	-6.317224	-2.416793	-1.764036
C	-3.483653	-1.392071	1.794510
H	-2.437100	-1.582252	2.052808
C	-3.653586	0.124379	1.662602
H	-2.996823	0.533302	0.887216
H	-3.405687	0.611967	2.610383
H	-4.683964	0.399289	1.413372
C	0.265747	-4.482438	5.635187
H	0.738622	-5.408766	5.296152
H	-0.702602	-4.730034	6.083897
H	0.893045	-4.060528	6.427552

Calculated energies and coordinates of **TS11**

Electronic energy ... -4711.92006110 Eh
Total Enthalpy ... -4710.22562904 Eh
Final Gibbs free energy ... -4710.43977270 Eh

CARTESIAN COORDINATES (ANGSTROEM)

Si	0.403898	-0.231667	-0.233329
Si	-0.259720	5.613925	-1.647135
Si	-1.580026	3.871984	-0.678716
Si	-3.383018	3.467013	-2.191605
Si	-2.508302	4.948044	1.244376
N	-1.491933	0.778441	-2.075495
N	0.756359	3.328083	1.289725
N	1.091500	0.962412	0.865300
N	-0.061641	-1.663602	0.426826
N	2.606052	-1.135242	-2.086643
N	0.219635	-3.776810	1.535058
N	-1.717846	-3.409833	0.571527
C	-0.735762	1.063125	-1.000345
C	-0.478163	-2.821651	0.793231
C	-0.959414	0.190433	-3.148782
C	0.439167	0.340207	-3.530761
C	1.569865	-0.683351	-1.688400
C	1.988196	3.592185	1.889398
C	-0.669699	2.235513	-0.272773
C	3.485171	-1.432567	-3.108184
C	1.033134	6.404400	-0.506631
H	1.848852	6.810276	-1.118526
H	1.464768	5.714243	0.221910
H	0.592761	7.235871	0.051974
C	0.423489	2.229879	0.698830
C	-1.835316	-0.539091	-4.029723
C	1.747028	0.701131	2.119115
C	3.154259	3.624375	1.084182
C	-2.797500	3.182416	-3.972678
H	-2.445939	4.134135	-4.389718
H	-3.638243	2.851437	-4.596223
H	-1.994415	2.448354	-4.061437
C	0.894945	-0.361639	-4.676272
H	1.923647	-0.209926	-4.995765
C	-1.436711	7.014709	-2.148725
H	-2.105389	7.313162	-1.334654
H	-2.051707	6.740830	-3.011765
H	-0.841272	7.892923	-2.429055
C	0.061948	-1.151871	-5.427413
H	0.439069	-1.672805	-6.303119
C	-1.314880	-1.202524	-5.113259
H	-1.985856	-1.772911	-5.752925
C	2.760518	-3.897600	0.148923
H	2.005585	-3.296430	-0.375583
C	0.638931	5.136188	-3.244906
H	1.023573	6.052430	-3.711404
H	-0.027436	4.647468	-3.962642
H	1.490895	4.474182	-3.064892
C	4.609693	-0.603151	-3.300206
C	1.169263	1.634750	-3.244997
H	0.746002	2.418605	-3.883636
H	2.227408	1.541454	-3.512143
H	1.091864	1.991068	-2.217419
C	4.366208	3.998759	1.657440
H	5.257119	4.021563	1.032997
C	3.116469	0.396200	2.187674
C	2.054269	4.030109	3.229407
C	0.986350	0.778790	3.301255
C	-3.296302	-0.550670	-3.725526
H	-3.474914	-0.867524	-2.695360
H	-3.836691	-1.216592	-4.405168
H	-3.714666	0.458481	-3.811796
C	2.661095	-3.549005	1.624380
C	3.076862	3.315393	-0.383492
H	2.788456	2.275691	-0.567093
H	4.041846	3.495875	-0.867396
H	2.323489	3.938297	-0.877749
C	3.279166	-2.599456	-3.875366
C	3.972600	0.240017	0.968090
H	4.387738	1.207665	0.664564
H	3.417998	-0.165407	0.122358
H	4.810499	-0.431641	1.180565
C	-0.580861	-4.910651	1.750188
C	-4.557602	4.951684	-2.378055
H	-5.239223	5.053036	-1.530664
H	-5.167414	4.771903	-3.273285
H	-4.048528	5.908921	-2.516078
C	2.438311	-5.385163	-0.083760
H	3.116455	-6.015388	0.503234
H	2.570411	-5.641203	-1.141251
H	1.411121	-5.631728	0.193889
C	3.290927	4.391842	3.761845
H	3.337013	4.708633	4.801696
C	4.448551	4.363467	2.996407
H	5.401327	4.653076	3.429479
C	-4.502005	2.086773	-1.535263
H	-3.957152	1.151927	-1.387895
H	-5.335217	1.904620	-2.226780

H	-4.932021	2.398898	-0.574936
C	-0.488903	1.020161	3.245391
H	-0.951387	0.864901	4.224248
H	-0.968130	0.360873	2.515067
H	-0.703593	2.041411	2.925642
C	1.418704	-3.526951	2.282597
C	4.879182	0.573041	-2.411838
H	5.372970	0.249262	-1.487593
H	3.965019	1.092346	-2.116130
H	5.538246	1.289166	-2.909093
C	3.718149	0.275061	3.439567
H	4.784015	0.065080	3.485319
C	4.127339	-3.582646	-0.460459
H	4.415873	-2.538163	-0.307904
H	4.107275	-3.769370	-1.537942
H	4.908088	-4.225702	-0.038238
C	-1.778509	-4.678975	1.171383
C	1.627907	0.662074	4.532636
H	1.038466	0.755975	5.441668
C	-2.263051	-4.587038	-1.979283
H	-1.854083	-5.216785	-1.180571
C	2.992836	0.434405	4.609400
H	3.484893	0.358011	5.574969
C	5.486014	-0.928326	-4.331205
H	6.344208	-0.285146	-4.506617
C	3.801511	-3.306285	2.388624
H	4.777031	-3.302208	1.916246
C	-3.115849	3.731006	2.558799
H	-3.926786	3.113583	2.157021
H	-3.512777	4.292704	3.414073
H	-2.339522	3.058806	2.926302
C	-2.835184	-2.897393	-0.163849
C	-3.195396	-3.566656	-1.350177
C	2.087485	-3.470016	-3.650121
H	1.178556	-2.968030	-4.007865
H	1.933264	-3.686700	-2.589258
H	2.190861	-4.416230	-4.187557
C	4.197822	-2.883639	-4.881047
H	4.044831	-3.771016	-5.489313
C	-0.103768	-6.120015	2.480535
H	0.922661	-5.981000	2.828502
H	-0.123381	-7.004259	1.834383
H	-0.726026	-6.333347	3.356867
C	-4.060368	5.961496	0.841648
H	-3.939520	6.632223	-0.013418
H	-4.297698	6.577314	1.719069
H	-4.923711	5.318338	0.645608
C	-1.065321	-3.887699	-2.632873
H	-1.385494	-3.272099	-3.479146
H	-0.353492	-4.636978	-2.994985
H	-0.546773	-3.228498	-1.928684
C	0.828206	4.098365	4.096100
H	0.880244	4.962385	4.766464
H	0.737620	3.201052	4.719050
H	-0.080727	4.178603	3.496879
C	-1.316159	6.196716	2.021778
H	-1.631257	6.419574	3.049200
H	-1.345201	7.137176	1.459640
H	-0.282049	5.847214	2.042512
C	1.310161	-3.346303	3.673033
C	-3.008610	-5.514710	1.189970
H	-3.173816	-6.066546	0.256449
H	-3.897906	-4.897066	1.355012
H	-2.938432	-6.243424	2.002325
C	-0.497089	-2.063480	4.883993
H	0.228493	-1.589812	5.552904
H	-1.450537	-2.157736	5.416395
H	-0.639062	-1.404197	4.026233
C	5.288365	-2.055797	-5.119869
H	5.988934	-2.293497	-5.914347
C	-4.419559	-3.239516	-1.928454
H	-4.740231	-3.741430	-2.834526
C	-3.633606	-1.900945	0.412546
C	3.712509	-3.069276	3.753812
H	4.612471	-2.864227	4.326187
C	-0.007106	-3.438848	4.428323
H	-0.771171	-3.836934	3.753906
C	-3.894786	-2.067880	2.896915
H	-4.987095	-2.094115	2.807920
H	-3.639791	-1.606170	3.857123
H	-3.527020	-3.099696	2.910223
C	-4.834556	-1.584184	-0.228524
H	-5.473810	-0.810298	0.182490
C	-2.945417	-5.513583	-2.986402
H	-3.829669	-6.001854	-2.562788
H	-2.244339	-6.293048	-3.301434
H	-3.251702	-4.971067	-3.887800
C	2.483166	-3.100269	4.388188
H	2.428124	-2.925669	5.458395
C	-5.234203	-2.262219	-1.368793
H	-6.180032	-2.012370	-1.841162
C	-3.275150	-1.264640	1.741917
H	-2.186992	-1.311746	1.852390
C	-3.673122	0.209194	1.829785
H	-3.212810	0.796712	1.028269
H	-3.338657	0.624814	2.784981
H	-4.758482	0.348539	1.783676
C	0.095130	-4.393912	5.627399
H	0.526359	-5.359573	5.346145
H	-0.900047	-4.569365	6.050462
H	0.716966	-3.971181	6.423672

Calculated energies and coordinates of **6a**

Electronic energy ... -4711.94828706 Eh
Total Enthalpy ... -4710.25156333 Eh
Final Gibbs free energy ... -4710.46377008 Eh

CARTESIAN COORDINATES (ANGSTROEM)

Si	5.967322	11.599297	5.157841
Si	5.443071	17.686915	4.076672
Si	4.111487	15.750394	4.467554
Si	2.669628	15.550835	2.578452
Si	2.589735	16.248019	6.225025
N	4.084416	12.487404	3.307277
N	6.502350	15.236033	6.405356
N	6.618950	12.837929	6.236434
N	5.633506	10.197495	5.958157
N	7.893486	10.518423	3.520722
N	6.226672	7.999956	6.721183
N	4.182839	8.269594	5.931403
C	4.769604	12.853254	4.438820
C	5.377957	8.956221	6.157887
C	4.700765	11.955151	2.293363
C	6.244672	11.907530	2.173331
C	6.864523	11.264925	3.470575
C	7.797045	15.384401	6.923450
C	4.940936	14.096817	4.977880
C	8.717102	9.955305	2.537772
C	6.779771	18.150789	5.319638
H	7.660529	17.506417	5.280921
H	6.398696	18.113142	6.344581
H	7.097971	19.179998	5.106561
C	6.056524	14.110213	5.957520
C	3.885901	11.466767	1.187393
C	7.196286	12.635565	7.533059
C	8.902047	15.209872	6.058186
C	3.486100	15.010197	0.956903
H	4.251850	15.737787	0.665637
H	2.729581	14.997306	0.161154
H	3.954673	14.026714	0.993036
C	6.733932	11.260417	0.912101
H	7.806773	11.244961	0.757209
C	4.261879	19.171246	4.095905
H	3.913351	19.370115	5.115824
H	3.385107	19.049487	3.457012
H	4.806155	20.062029	3.757096
C	5.907978	10.827272	-0.050243
H	6.315558	10.416912	-0.970195
C	4.479455	10.928527	0.093034
H	3.851909	10.558155	-0.714689

C	8.908681	7.996997	5.523180
H	8.207113	8.636080	4.973897
C	6.229288	17.593742	2.354371
H	6.875848	18.463640	2.184560
H	5.463646	17.587450	1.571022
H	6.840600	16.692909	2.228041
C	9.971598	10.537459	2.260420
C	6.742109	13.384313	2.070584
H	6.327874	13.831675	1.162528
H	7.832537	13.406323	2.002156
H	6.426665	13.976586	2.929122
C	10.189291	15.434073	6.544296
H	11.034371	15.292542	5.873573
C	8.524417	12.206041	7.680953
C	8.005535	15.892336	8.222490
C	6.397000	12.876050	8.664328
C	2.398338	11.558561	1.303843
H	2.054679	11.133594	2.252385
H	1.914866	11.036796	0.472948
H	2.073679	12.602844	1.297577
C	8.666728	8.259904	7.000653
C	8.700844	14.844474	4.615283
H	8.334411	13.819548	4.492066
H	9.641031	14.934191	4.062535
H	7.959266	15.500614	4.144098
C	8.383537	8.690353	2.010469
C	9.362793	11.833557	6.498670
H	9.621885	12.724037	5.918592
H	8.844298	11.133557	5.834176
H	10.292668	11.362426	6.828923
C	5.550471	6.772281	6.848576
C	1.936502	17.238372	2.109466
H	1.487152	17.779773	2.945220
H	1.148964	17.064838	1.364306
H	2.686917	17.886051	1.644034
C	8.631936	6.534173	5.137993
H	9.206376	5.849856	5.773804
H	8.935542	6.369376	4.098174
H	7.573656	6.278806	5.219326
C	9.308659	16.083581	8.673521
H	9.461767	16.442337	9.688650
C	10.404196	15.840910	7.853741
H	11.413115	15.999325	8.222647
C	1.185127	14.453336	3.006739
H	1.491813	13.486757	3.417259
H	0.563699	14.277344	2.119270
H	0.556638	14.957451	3.749292
C	4.926426	13.142953	8.527260
H	4.427420	13.062706	9.497382
H	4.471642	12.417759	7.842769
H	4.714619	14.137214	8.119206
C	7.370743	8.299488	7.546190
C	10.355230	11.853801	2.868473
H	11.412807	12.067908	2.694091
H	10.170603	11.857727	3.946512
H	9.775596	12.685345	2.450048
C	9.068658	12.147049	8.962056
H	10.104744	11.839118	9.074349
C	10.320905	8.364350	5.062477
H	10.576458	9.401467	5.299911
H	10.391970	8.243183	3.978193
H	11.074227	7.707626	5.514156
C	4.303068	6.934363	6.358662
C	6.984326	12.824399	9.927931
H	6.370430	13.033532	10.801268
C	3.120427	7.524308	3.287179
H	4.001741	7.110602	3.790409
C	8.320173	12.485653	10.080318
H	8.769362	12.456598	11.069051
C	10.852383	9.861086	1.420406
H	11.816014	10.314470	1.201120
C	9.736676	8.460709	7.874739
H	10.750476	8.447415	7.491855
C	1.810266	14.622810	6.793528
H	1.196767	14.169916	6.007708
H	1.182165	14.773202	7.680459
H	2.596246	13.905997	7.047241
C	2.956227	8.861700	5.479545
C	2.446465	8.513206	4.219109
C	7.045945	8.070588	2.279699
H	6.296924	8.509988	1.612504
H	6.703301	8.244412	3.305683
H	7.069884	6.992436	2.096953
C	9.303036	8.046513	1.184659
H	9.042386	7.073307	0.774894
C	6.177040	5.544311	7.412736
H	5.467185	5.013295	8.054765
H	7.059025	5.791825	8.009080
H	6.495489	4.848206	6.627859
C	1.197085	17.424244	5.714972
H	1.583124	18.377318	5.339386
H	0.582422	17.640373	6.598159
H	0.540540	16.998427	4.950529
C	3.597554	8.234063	2.019693
H	2.750125	8.644150	1.460090
H	4.134032	7.540962	1.362303
H	4.260157	9.064578	2.267104
C	6.847459	16.268730	9.097757
H	7.195978	16.568009	10.090256
H	6.139852	15.446070	9.221856
H	6.297152	17.109898	8.660606
C	3.450735	17.046303	7.699571
H	4.267795	16.418639	8.061395
H	2.740633	17.203201	8.521175
H	3.878408	18.018759	7.430867
C	7.143437	8.504842	8.919055
C	3.208475	5.932897	6.257853
H	3.275825	5.334898	5.341760
H	2.227224	6.416825	6.267914
H	3.257183	5.241528	7.104869
C	5.413009	9.802381	10.215195
H	6.093046	10.018106	11.046105
H	4.393278	9.771675	10.615545
H	5.491507	10.629071	9.506868
C	10.530648	8.621018	0.880332
H	11.235820	8.102412	0.238270
C	1.248127	9.102995	3.807160
H	0.839145	8.849403	2.833433
C	2.245321	9.713333	6.353248
C	9.533071	8.684275	9.229575
H	10.384801	8.844900	9.884846
C	5.759788	8.471185	9.543706
H	5.026533	8.299125	8.751619
C	2.282762	8.609922	8.591413
H	1.207636	8.415500	8.502290
H	2.516613	8.737140	9.653974
H	2.821682	7.729895	8.232464
C	1.075782	10.303934	5.877804
H	0.524616	10.988960	6.511950
C	2.180776	6.366540	2.911869
H	1.709273	5.914544	3.788343
H	2.736786	5.589397	2.375955
H	1.380760	6.712841	2.248844
C	8.249174	8.707298	9.745270
H	8.097651	8.884671	10.806216
C	0.585074	10.012707	4.611417
H	-0.329354	10.484664	4.263119
C	2.651527	9.883541	7.807466
H	3.738329	10.022853	7.849438
C	2.002183	11.085251	8.495515
H	2.110373	12.003828	7.914555
H	2.472738	11.244879	9.470747
H	0.933529	10.916792	8.672039
C	5.629719	7.317550	10.549578
H	5.891884	6.355475	10.101047
H	4.600311	7.255467	10.920392
H	6.286598	7.473987	11.412345

Calculated energies and coordinates of **INT12**

Electronic energy ... -4422.21289951 Eh
Total Enthalpy ... -4420.67385076 Eh
Final Gibbs free energy ... -4420.87716411 Eh

CARTESIAN COORDINATES (ANGSTROEM)

C	5.047707	5.417929	24.428318
H	5.714984	4.648420	24.028065
H	4.149591	5.457453	23.797526
H	4.747883	5.095465	25.427385
C	9.840094	4.318779	34.199837
H	8.479089	5.484210	35.378998
H	9.526183	3.378151	34.642490
Si	9.593419	5.880001	29.232728
N	8.633295	4.681597	28.429363
N	11.144109	5.513705	29.602683
C	9.115240	7.282815	27.982805
C	8.116214	5.227919	27.202654
C	8.754611	3.256321	28.561160
C	12.188676	5.468417	30.358849
N	9.548869	8.518838	28.072419
C	8.504798	6.106196	30.811804
Si	8.085939	7.372809	25.226714
C	8.529267	6.593986	26.920831
N	7.304714	4.595173	26.422598
C	9.639107	2.583728	27.697947
H	9.962701	3.936094	26.032975
H	11.163739	2.643641	26.188418
H	11.185715	4.027066	27.294983
H	10.350451	0.662649	27.068989
C	8.012239	2.554119	29.526365
H	7.791610	3.613835	31.383074
H	6.431708	2.559911	30.971602
H	6.633808	4.102527	30.136882
H	7.509334	0.612740	30.276381
N	12.290139	5.576853	31.733128
N	13.490382	5.283167	29.927702
C	9.209523	9.468494	28.994644
O	7.653570	6.547339	31.445208
Si	5.869826	7.114140	24.379321
H	6.432179	8.461676	22.290756
H	4.905019	7.578362	22.145661
H	6.439363	6.719689	21.975849
H	4.684247	8.241440	26.297061
H	3.688994	8.244843	24.831493
H	5.027418	9.393952	25.001365
Si	9.542097	6.378111	23.629576
H	8.786188	7.525102	21.512199
H	10.406585	6.831788	21.356865
H	10.180969	8.363139	22.214268
H	11.816823	7.160103	24.256741
H	11.854282	5.437224	23.846024
H	11.263279	5.959560	25.433376
H	9.644305	4.207924	22.409400
H	7.958343	4.716149	22.629216
H	8.838518	4.010649	23.982218
C	6.450620	3.587955	26.877803
C	10.530566	3.335558	26.750550
C	9.690854	1.190907	27.753195
H	8.957392	-0.605383	28.678088
C	7.169146	3.249732	30.552633
C	8.103247	1.163238	29.551402
C	11.202742	5.558146	32.664451
C	13.628656	5.436452	32.139397
H	13.584940	6.333603	34.086459
H	15.105207	5.521627	33.661684
H	13.677120	4.573416	34.101602
C	14.370893	5.261431	31.021262
H	16.106287	4.043148	30.652004
H	16.360121	5.406310	31.751138
H	16.205183	5.681081	30.006068
C	13.881573	4.941751	28.590470
Si	8.572935	9.704980	25.060047
H	8.328257	9.745497	22.521423
H	8.402917	11.358980	23.241311
H	6.901579	10.433134	23.321448
H	10.884486	9.414485	25.937215
H	10.677768	11.040376	25.244913
H	10.915490	9.645453	24.177501
H	6.643592	10.663682	26.349884
H	7.814774	11.899067	25.873955
H	8.140595	10.862302	27.270944
C	7.876778	9.635009	29.485356
H	6.813067	8.560163	27.951097
H	5.788888	9.254119	29.221960
H	6.714988	7.790068	29.528781
H	6.556020	10.858313	30.636180
C	10.180147	10.460594	29.328945
H	12.018460	9.367613	29.142525
H	12.213181	11.135567	29.197073
H	11.605824	10.330683	27.733451
H	10.575028	12.306236	30.330652
C	5.932769	7.520249	22.526685
C	4.712225	8.365415	25.208649
C	9.737552	7.379208	22.033366
C	11.279487	6.212320	24.368395
C	8.939181	4.665729	23.114708
C	5.446071	3.932689	27.814257
H	6.173966	5.711302	28.844145
H	4.423530	5.455026	28.937296
H	5.186283	6.037587	27.442545
H	3.784131	3.223393	28.963745
C	6.449638	2.303831	26.293806
H	7.116266	2.524178	24.267854
H	7.349218	0.900613	24.943093
H	8.421474	2.229440	25.413294
H	5.572784	0.353854	26.330817
C	8.917754	0.479982	28.659438
C	10.839504	4.322229	33.224413
H	12.348506	3.224101	32.181522
C	10.603117	6.771218	33.049813
H	9.131270	7.619670	34.366918
H	10.962484	7.919077	31.294679
C	14.018223	5.470022	33.571355
C	15.835757	5.086988	30.847813
C	14.188524	3.598665	28.320426
H	14.801492	2.232668	26.777255
H	14.009722	2.960193	30.354083
C	13.986712	5.952057	27.617108
H	14.429570	6.299251	25.541810
H	12.718158	7.443237	28.424874
C	7.992550	10.348459	23.368462
C	10.444803	9.976532	25.110033
C	7.714432	10.878952	26.266769
C	6.744912	8.760685	29.024251
C	7.579767	10.733037	30.290188
H	8.260981	12.561131	31.202117
C	11.581913	10.322087	28.827099
C	9.821366	11.555174	30.102968
C	5.299805	5.351819	28.290889
C	4.547145	2.955170	28.236058
H	3.907300	0.904989	28.083316
C	7.387851	1.969091	25.173587
C	5.543884	1.354107	26.756884
C	11.452700	3.008932	32.776287
H	12.516091	2.657301	34.664435
H	12.435843	1.255459	33.579480
H	11.013276	1.738143	34.502323
H	9.536003	2.040159	32.416207
H	10.886136	1.310382	31.533410
H	10.204317	2.848490	30.990765
C	9.625964	6.711674	34.044310
C	10.965847	8.091864	32.383839
H	8.930295	8.909150	32.407851
H	10.199014	10.079327	32.085131
H	9.971492	9.486349	33.732116
H	12.452097	8.718469	33.847215
H	12.543618	9.568713	32.295005
H	13.163669	7.921226	32.424273
C	14.572545	3.265947	27.019954
H	14.935913	3.960397	25.023169
C	14.061103	2.500706	29.360676
H	12.728581	1.289186	28.143175

H	11.885382	2.393167	29.232300
H	12.648527	0.925193	29.876092
H	15.202831	0.873639	30.223866
H	16.212792	2.071668	29.388244
H	15.260084	0.904589	28.463102
C	14.364843	5.561573	26.331929
C	13.707880	7.410797	27.946661
H	14.721119	7.518498	29.906500
H	14.546418	9.062028	29.072649
H	15.754467	7.896392	28.509929
H	14.626251	8.340532	26.200831
H	13.406102	9.324886	27.000432
H	12.897801	7.968569	25.993286
C	8.528622	11.699017	30.598886
C	4.606785	1.660252	27.737539
C	11.879595	2.121524	33.953128
C	10.463564	2.260172	31.875136
C	9.249295	5.501688	34.613185
C	9.951800	9.196216	32.674946
C	12.370475	8.592540	32.761054
C	14.649693	4.235126	26.034450
C	12.752363	1.733946	29.144074
C	15.256631	1.539049	29.355640
C	14.743894	7.994916	28.923915
C	13.654250	8.302888	26.706698

Calculated energies and coordinates of **TS12**

Electronic energy	...	-4422.20596576 Eh
Total Enthalpy	...	-4420.66691569 Eh
Final Gibbs free energy	...	-4420.86708740 Eh

CARTESIAN COORDINATES (ANGSTROEM)

C	4.320566	4.966792	25.116294
H	4.080779	5.212318	24.076497
H	3.430710	4.493928	25.552458
H	5.133883	4.238627	25.105865
C	8.092725	6.219821	33.079776
H	7.232510	8.075402	33.722844
H	7.164305	5.676329	33.210645
Si	9.812317	5.788295	28.686292
N	9.249696	4.494207	27.669514
N	11.097094	5.436037	29.623340
C	9.226364	7.217061	27.570456
C	8.335653	5.069582	26.724926
C	9.977998	3.294117	27.364656
C	11.857833	5.349973	30.663401
C	8.071238	6.118044	29.541368
N	9.339114	8.503461	27.604156
Si	6.917519	7.440002	25.770449
C	8.190603	6.519380	26.865902
N	7.664868	4.431577	25.826301
C	11.067123	3.389629	26.478055
H	10.744388	5.344979	25.596966
H	11.987251	5.286153	26.850195
H	12.326325	4.623609	25.246193
H	12.533901	2.294583	25.364092
C	9.624712	2.059873	27.928271
H	8.269569	2.897705	29.383257
H	9.081973	1.402317	29.883845
H	7.758713	1.340746	28.714706
H	9.962890	-0.054608	27.865241
N	11.583041	5.659910	31.983166
N	13.170397	4.916889	30.667682
O	7.118295	6.445844	30.084459
C	10.366654	9.270222	28.129351
Si	4.716651	6.558378	26.063858
H	3.382301	8.719572	25.991987
H	2.396619	7.285465	25.663785
H	3.432475	7.948343	24.396547
H	3.395844	5.899658	28.077548
H	4.410297	7.312433	28.419787
H	5.123892	5.698812	28.412369
Si	7.582202	7.228815	23.494162
H	6.358057	9.168896	22.454661
H	7.785129	8.706228	21.514761
H	7.969259	9.666817	22.992203
H	10.036805	7.518973	23.873559
H	9.721761	6.658995	22.354096
H	9.589834	5.806199	23.902351
H	5.538395	6.313593	22.393920
H	6.482200	5.004071	23.117707
H	7.002376	5.738867	21.582390
C	7.515417	3.044507	25.724867
C	11.550236	4.730585	26.009327
C	11.704678	2.223663	26.063386
H	11.763360	0.075854	26.169163
C	8.623790	1.926354	29.037186
C	10.269204	0.910176	27.467053
C	10.397008	6.319377	32.435771
C	12.717753	5.436204	32.782053
H	11.812133	6.119981	34.597534
H	13.581390	6.191747	34.595322
H	12.756186	4.638877	34.776727
C	13.696100	4.976589	31.968080
H	15.181775	3.459518	32.344351
H	15.371439	4.959133	33.266104
H	15.794436	4.903225	31.542926
C	13.812906	4.218427	29.590596
Si	6.644890	9.763512	26.231410
H	4.775989	9.797138	24.470718
H	6.073942	10.934040	24.095327
H	4.906203	11.315740	25.364399
H	8.673518	11.004031	27.108537
H	7.780164	11.931111	25.883810
H	8.855790	10.606839	25.404208
H	6.114947	9.286756	28.664673
H	4.669019	9.727094	27.754474
H	5.803402	10.987659	28.256604
C	10.129994	10.115484	29.236531
H	7.985113	10.125581	29.337223
H	8.788489	10.748500	30.793872
H	8.739302	9.009303	30.467782
H	10.888117	11.728784	30.423504
C	11.565069	9.407278	27.389767
H	11.707060	7.572868	26.231209
H	12.648071	8.933491	25.589654
H	10.875174	8.896230	25.441321
H	13.423416	10.458631	27.211316
C	3.369726	7.757081	25.473305
C	4.389461	6.338411	27.919058
C	7.401780	8.850153	22.533232
C	9.407348	6.758293	23.400749
C	6.555935	5.943829	22.563821
C	6.622588	2.370474	26.587817
H	6.541576	3.823844	28.192644
H	5.505797	2.403608	28.426228
H	5.051309	3.667314	27.278211
H	5.711331	0.493730	27.073197
C	8.109929	2.344844	24.650809
H	8.477556	3.901539	23.211297
H	9.334288	2.378968	22.889302
H	9.887399	3.465674	24.171231
H	8.344603	0.447031	23.692582
C	11.283663	0.983021	26.525905
C	9.226551	5.558456	32.606160
H	9.711529	3.892786	31.368101
C	10.472944	7.691359	32.750251
H	9.301167	9.354894	33.451107
H	12.487609	7.996243	33.327804
C	12.717513	5.606942	34.263465
C	15.081580	4.550708	32.294727
C	13.886525	2.817585	29.678660
H	14.585267	1.052725	28.669144
H	13.178023	2.710198	31.692971
C	14.374169	4.942544	28.526128
H	15.436043	4.727909	26.669247
H	13.325377	6.780733	28.774302
C	5.484927	10.501190	24.909533
C	8.134037	10.930123	26.162462
C	5.732136	9.956377	27.888354

C	8.845358	9.996876	30.000610
C	11.088427	11.063606	29.586599
H	13.018780	11.923845	29.171346
C	11.720687	8.658853	26.098633
C	12.513336	10.344030	27.796798
C	5.897399	3.102817	27.681032
C	6.396071	1.007258	26.400576
H	6.834700	-0.758533	25.250298
C	8.998603	3.059344	23.677131
C	7.864131	0.982457	24.508170
C	9.203195	4.062079	32.327642
H	11.038989	3.533124	33.411384
H	9.881820	2.208878	33.236042
H	9.564938	3.507743	34.401941
H	7.271709	3.503774	33.177936
H	7.850287	2.439311	31.896861
H	7.174837	4.019724	31.483063
C	9.303395	8.298216	33.213696
C	11.777335	8.476067	32.639499
H	13.040581	7.543321	31.107774
H	13.040319	9.300630	31.068898
H	11.651424	8.426197	30.446179
H	10.963364	10.492463	32.463030
H	12.622086	10.412548	33.055444
H	11.289746	9.999133	34.141981
C	14.525308	2.135835	28.643048
H	15.557939	2.277500	26.768758
C	13.243982	2.050712	30.821318
H	11.805028	0.966642	29.607690
H	11.240915	2.548528	30.130209
H	11.301055	1.203011	31.299399
H	13.619811	0.398308	32.166662
H	15.096100	1.051047	31.427491
H	13.999890	0.024267	30.488353
C	14.999655	4.209985	27.515698
C	14.337772	6.461522	28.488438
H	15.110418	6.783484	30.524102
H	15.300603	8.159441	29.435613
H	16.353250	6.738300	29.257301
H	15.678748	6.894056	26.820334
H	14.445496	8.120305	27.126257
H	13.995906	6.608067	26.327291
C	12.283928	11.176354	28.887084
C	7.019420	0.303859	25.379050
C	9.974932	3.286189	33.409899
C	7.790432	3.484456	32.212925
C	8.129558	7.574916	33.369771
C	12.409783	8.422779	31.232454
C	11.643218	9.927000	33.108128
C	15.071103	2.824687	27.571174
C	11.809168	1.668953	30.447042
C	14.042327	0.813889	31.245744
C	15.333804	7.065934	29.492929
C	14.630535	7.043186	27.104484

Calculated energies and coordinates of **INT13**

Electronic energy ... -4422.23128653 Eh
Total Enthalpy ... -4420.69055828 Eh
Final Gibbs free energy ... -4420.89279776 Eh

CARTESIAN COORDINATES (ANGSTROEM)

C	3.194446	6.364514	26.226895
H	3.030444	7.378102	25.844028
H	2.258566	6.029270	26.691373
H	3.394855	5.713590	25.369562
C	7.687771	6.713460	32.243115
H	6.793870	8.658368	32.355451
H	6.730796	6.206516	32.239092
Si	9.947264	5.861486	28.645712
N	9.597196	4.591473	27.399292
N	11.236440	5.461415	29.592995
C	9.502710	7.155751	27.268681
C	8.523126	5.044519	26.623225
C	10.224380	3.322676	27.234515
C	11.766667	5.458387	30.774100
C	8.030974	6.151984	28.732740
N	9.896017	8.090870	26.517305
Si	6.531642	7.238911	26.422637
C	8.131719	6.432481	27.171009
N	7.887251	4.540005	25.635684
C	11.561383	3.253376	26.814399
H	11.815938	5.362958	26.394072
H	12.934445	4.697468	27.572031
H	13.156441	4.328945	25.855495
H	13.144129	1.932737	26.228144
C	9.491623	2.154986	27.513576
H	7.421553	2.757752	27.498090
H	8.183540	2.817039	29.080284
H	7.741479	1.250491	28.366716
H	9.493431	0.015266	27.409715
N	11.292598	5.949211	31.978937
N	13.005615	4.930826	31.081277
O	7.032105	6.166459	29.398603
C	11.137500	8.737474	26.711860
Si	4.604058	6.312158	27.494365
H	4.742358	7.303037	29.787905
H	3.093010	6.792009	29.380179
H	3.713093	8.326633	28.763853
H	4.039647	3.872244	27.664389
H	4.686338	4.504736	29.194658
H	5.780438	4.109398	27.869931
Si	6.437733	7.146024	24.052044
H	4.269088	8.427970	23.866911
H	5.190321	8.458194	22.357432
H	5.655842	9.518127	23.693786
H	8.527515	8.480669	23.761746
H	8.133969	7.577018	22.277637
H	8.860109	6.745706	23.669497
H	4.821581	5.265098	23.617794
H	6.492828	4.724737	23.385067
H	5.705236	5.745178	22.165712
C	8.180300	3.364352	24.926635
C	12.407946	4.479369	26.639945
C	12.114263	1.994406	26.571536
H	11.804959	-0.131236	26.521751
C	8.136516	2.245322	28.144906
C	10.069103	0.920200	27.233509
C	10.077750	6.688992	32.113024
C	12.224265	5.719375	33.005091
H	10.981050	6.529472	34.541657
H	12.711073	6.803579	34.801527
H	12.015670	5.201851	35.078010
C	13.279342	5.080099	32.448236
H	14.479941	3.436400	33.136381
H	14.602497	4.922340	34.096004
H	15.406296	4.801081	32.517490
C	13.812508	4.132635	30.207076
Si	6.433783	9.538496	27.016033
H	4.608528	11.215812	27.100225
H	3.897563	9.599682	27.014493
H	4.551282	10.353435	25.557560
H	8.654851	10.270330	26.042863
H	7.622696	11.660935	26.460110
H	7.304451	10.713581	25.001758
H	7.472708	9.131274	29.304132
H	5.756373	9.502042	29.408641
H	6.920843	10.806676	29.120029
C	11.306409	9.625115	27.792427
H	9.289399	10.220648	28.224958
H	10.469298	10.681284	29.460538
H	9.898848	9.012008	29.342210
H	12.639161	11.016192	28.729071
C	12.131194	8.611841	25.722762
H	12.069669	6.691012	24.725314
H	12.602876	8.034291	23.711893
H	10.885786	7.852538	24.142203
H	14.091664	9.223289	25.116383
C	3.989882	7.294496	28.994314
C	4.800156	4.525955	28.105570
C	5.277357	8.520943	23.449531

C	8.155059	7.526896	23.373527
C	5.805996	5.562691	23.243541
C	7.171867	2.380289	24.810517
H	5.679503	2.098023	26.366864
H	5.045172	2.156077	24.723082
H	5.586544	3.646517	25.527824
H	6.672235	0.443244	24.049971
C	9.377022	3.216356	24.189662
H	10.274948	5.094919	24.811632
H	10.180397	4.853163	23.071362
H	11.384939	3.968262	24.014360
H	10.552882	1.904808	22.977714
C	11.368556	0.837929	26.746917
C	8.866155	5.974769	32.136780
H	9.523071	4.142082	31.271718
C	10.149156	8.091041	32.242204
H	8.925462	9.857555	32.387503
H	11.976027	8.392220	33.264952
C	11.973340	6.083987	34.429812
C	14.506071	4.531901	33.079961
C	13.718298	2.736810	30.323702
H	14.505631	0.881302	29.573444
H	12.433303	2.790047	32.028513
C	14.696786	4.765100	29.319040
H	16.218235	4.396270	27.845603
H	13.701551	6.637085	29.289256
C	4.707256	10.227598	26.632656
C	7.628144	10.644139	26.046482
C	6.682732	9.761050	28.886176
C	10.186188	9.893140	28.757727
C	12.508471	10.325136	27.899450
H	14.446262	10.729089	27.055070
C	11.910812	7.753632	24.510692
C	13.319399	9.325315	25.875421
C	5.799601	2.584314	25.391277
C	7.447030	1.204381	24.113746
H	8.876380	0.074195	22.966953
C	10.357396	4.343310	24.027499
C	9.612770	2.022369	23.512552
C	8.844353	4.452024	32.078849
H	10.409049	4.078784	33.582141
H	9.280816	2.741320	33.342314
H	8.762657	4.181735	34.237598
H	6.773203	4.011615	32.607220
H	7.559846	2.784911	31.615648
H	7.021722	4.305151	30.873204
C	8.934354	8.776305	32.317464
C	11.481144	8.828428	32.385768
H	12.902549	7.660439	31.185300
H	13.267198	9.374524	31.285413
H	11.957463	8.814415	30.242622
H	10.868719	10.858268	31.846954
H	12.293698	10.761388	32.884056
H	10.689201	10.492597	33.575721
C	14.559980	1.964095	29.522729
H	16.110943	1.946153	28.040416
C	12.688596	2.084155	31.228537
H	11.597501	1.093603	29.626162
H	11.028662	2.724480	29.962185
H	10.625339	1.399281	31.083014
H	12.470815	0.462984	32.643607
H	14.158839	0.952051	32.392978
H	13.311903	-0.012102	31.170796
C	15.525241	3.947331	28.549236
C	14.734462	6.278818	29.189988
H	15.160581	6.706767	31.300969
H	15.609000	8.000330	30.182850
H	16.602120	6.531240	30.277822
H	16.316844	6.579376	27.706988
H	15.078711	7.841784	27.747908
H	14.710216	6.286370	27.001656
C	13.519501	10.170668	26.959356
C	8.671994	1.001282	23.494310
C	9.362998	3.832894	33.389321
C	7.464957	3.864957	31.769265
C	7.722864	8.097435	32.309253
C	12.449276	8.650715	31.202358
C	11.311352	10.319372	32.692099
C	15.460144	2.563732	28.653371
C	11.405098	1.810633	30.430745
C	13.194937	0.800896	31.894991
C	15.574760	6.912734	30.309979
C	15.243195	6.763486	27.830023

Calculated energies and coordinates of **TS13**

Electronic energy ... -4422.21076089 Eh
Total Enthalpy ... -4420.67196427 Eh
Final Gibbs free energy ... -4420.87195201 Eh

CARTESIAN COORDINATES (ANGSTROEM)

C	-6.789231	0.593694	-2.376122
H	-6.938884	1.662943	-2.558725
H	-7.738567	0.180454	-2.012931
H	-6.560825	0.118439	-3.336185
C	-1.943941	1.489690	3.966127
H	-2.439638	3.573422	4.040397
H	-2.980698	1.182383	4.029460
Si	0.072435	-0.132152	0.287810
N	-0.182138	-1.286863	-1.024723
N	1.327815	-0.561261	1.267005
C	0.035693	1.392096	-0.845532
C	-1.248739	-0.797474	-1.814268
C	0.126265	-2.685503	-0.995494
C	1.861716	-0.549515	2.444458
C	-1.820475	0.342148	0.292893
N	0.232794	2.492008	-1.303213
Si	-3.412118	1.237966	-1.959483
C	-1.881105	0.389066	-1.181796
N	-1.671829	-1.258224	-2.938778
C	1.347625	-3.172625	-1.476450
H	2.133004	-1.232310	-1.996222
H	3.280789	-2.310945	-1.197602
H	2.849020	-2.586986	-2.892919
H	2.470309	-4.939139	-1.936193
C	-0.838729	-3.564066	-0.465500
H	-2.636550	-2.355168	-0.428651
H	-1.797970	-2.458061	1.113914
H	-2.726819	-3.843257	0.524622
H	-1.379411	-5.617884	-0.173305
N	1.444463	0.045509	3.618734
N	3.038603	-1.186382	2.783143
O	-2.678376	0.702949	1.079834
C	1.239509	3.468159	-1.374133
Si	-5.423086	0.287452	-1.099823
H	-5.216482	0.914091	1.303348
H	-6.891612	0.490777	0.879112
H	-6.253274	2.089735	0.476530
H	-4.894684	-2.129550	-1.618187
H	-6.344126	-1.955949	-0.611723
H	-4.740059	-1.779080	0.113238
Si	-3.400967	1.251024	-4.329428
H	-5.699593	2.258017	-4.650161
H	-4.658753	2.501775	-6.058539
H	-4.460898	3.525641	-4.632515
H	-0.912417	1.380790	-4.352386
H	-1.624667	2.965854	-4.677801
H	-1.563995	1.741385	-5.963394
H	-4.690465	-0.859067	-4.740830
H	-2.990054	-1.075497	-5.174572
H	-4.077204	-0.162640	-6.247036
C	-1.070866	-2.299108	-3.667149
C	2.458519	-2.269550	-1.920335
C	1.536230	-4.556498	-1.531441
H	0.704266	-6.507257	-1.188467
C	-2.065194	-3.029232	0.214875
C	-0.622513	-4.936157	-0.553705
C	0.377213	0.990825	3.720262
C	2.364267	-0.203819	4.653209
H	1.160178	0.125821	6.392100
H	2.299478	1.427150	6.059549

H	2.887472	-0.112967	6.709288
C	3.353885	-0.967170	4.130462
H	4.684285	-1.187742	5.771884
H	5.484215	-1.125790	4.191498
H	4.646314	-2.593777	4.691032
C	3.844862	-1.956101	1.887880
Si	-3.616118	3.517362	-1.316544
H	-6.130470	3.615255	-1.085911
H	-5.645782	4.019455	-2.736702
H	-5.430653	5.199375	-1.437337
H	-1.443074	4.333359	-2.344326
H	-2.521216	5.685678	-1.926376
H	-2.835166	4.706293	-3.360751
H	-2.437801	3.331429	0.917699
H	-4.181742	3.297735	1.086806
H	-3.364567	4.846172	0.783064
C	1.116674	4.626431	-0.588100
H	-0.968132	4.935900	-0.174285
H	0.130125	5.666369	1.007061
H	-0.160669	3.917088	1.005430
H	2.029016	6.495643	-0.091987
C	2.289455	3.303602	-2.293771
H	2.432112	1.169497	-2.583375
H	3.232119	2.139399	-3.828455
H	1.463777	2.006560	-3.793731
H	4.063384	4.196065	-3.086995
C	-5.995556	1.029229	0.544010
C	-5.330567	-1.572383	-0.785206
C	-4.679407	2.505177	-4.961120
C	-1.713243	1.894556	-4.887470
C	-3.824042	-0.370201	-5.199670
C	-1.771780	-3.513110	-3.837564
H	-3.027872	-3.852142	-2.115826
H	-3.618522	-4.582784	-3.615396
H	-3.740505	-2.824663	-3.359288
H	-1.751990	-5.462071	-4.719252
C	0.130874	-2.091056	-4.377434
H	0.669024	-0.208159	-3.458822
H	0.408558	-0.132673	-5.200563
H	1.887014	-0.862305	-4.566764
H	1.597077	-2.964560	-5.669837
C	0.551137	-5.434739	-1.107906
C	-0.941676	0.525985	3.849637
H	-0.712969	-1.408611	3.006309
C	0.718253	2.355432	3.725455
H	-0.106630	4.338261	3.853108
H	2.810807	2.011809	3.950555
C	2.168728	0.338936	6.022610
C	4.603980	-1.498916	4.728686
C	3.743748	-3.356448	1.940694
H	4.506214	-5.175435	1.084745
H	2.688329	-3.417178	3.787748
C	4.745873	-1.290127	1.046917
H	6.249393	-1.589058	-0.462113
H	4.263818	0.623330	1.844197
C	-5.373557	4.131009	-1.683326
C	-2.485832	4.662584	-2.323184
C	-3.361543	3.775025	0.540948
C	-0.027439	4.795328	0.364757
C	2.103613	5.603145	-0.708139
H	3.917318	6.232220	-1.677580
C	2.361132	2.091174	-3.170442
C	3.244864	4.314179	-2.381690
C	-3.113156	-3.707130	-3.198217
C	-1.211604	-4.524126	-4.614193
H	0.439373	-5.152388	-5.846781
C	0.812250	-0.754032	-4.392014
C	0.660175	-3.130462	-5.141343
C	-1.256272	-0.964967	3.854734
H	0.331257	-1.651161	5.222715
H	-1.079153	-2.707310	5.133084
H	-1.186321	-1.178193	6.020940
H	-3.322484	-0.982861	4.551541
H	-2.869485	-2.360226	3.547601
H	-3.156326	-0.780163	2.790574
C	-0.323195	3.274589	3.849712
C	2.161973	2.809709	3.573369
H	2.241671	2.160498	1.479441
H	3.609738	3.170744	1.988606
H	2.031129	3.902894	1.697381
H	1.986017	4.955203	3.966261
H	3.559772	4.265242	4.354209
H	2.176699	3.972965	5.428469
C	4.560826	-4.092024	1.082839
H	6.078328	-4.047510	-0.435322
C	2.759126	-4.030621	2.880075
H	1.371455	-4.666399	1.330921
H	1.012741	-3.071159	1.979960
H	0.640924	-4.519476	2.944506
H	2.529685	-5.802032	4.094859
H	4.220696	-5.444880	3.691957
H	3.133440	-6.145100	2.476274
C	5.546566	-2.071923	0.210903
C	4.851985	0.222065	1.010768
H	6.742236	0.296920	2.107839
H	6.319134	1.798129	1.258830
H	6.929623	0.412637	0.350704
H	4.808152	0.410608	-1.161281
H	4.271392	1.867593	-0.298099
H	3.210382	0.445907	-0.390912
C	3.162886	5.456002	-1.594555
C	0.010846	-4.351808	-5.251144
C	-0.757528	-1.660253	5.134541
C	-2.742551	-1.279497	3.669370
C	-1.638658	2.843709	3.964685
C	2.529543	3.017799	2.097655
C	2.482360	4.071390	4.381328
C	5.451336	-3.455622	0.225640
C	1.360429	-4.071952	2.248965
C	3.194974	-5.434072	3.307272
C	6.295974	0.704806	1.194736
C	4.249473	0.772017	-0.289555

Calculated energies and coordinates of **INT14**

Electronic energy ... -4422.23702070 Eh
Total Enthalpy ... -4420.69775991 Eh
Final Gibbs free energy ... -4420.90187318 Eh

CARTESIAN COORDINATES (ANGSTROEM)

C	4.519088	4.938361	24.605084
H	4.111564	5.715362	23.948492
H	3.867122	4.059454	24.519005
H	5.514063	4.674485	24.238187
C	8.253486	7.706970	32.345588
H	7.983740	9.827272	32.486004
H	7.194568	7.504701	32.459610
Si	10.230483	5.817507	28.556284
N	9.739632	4.733804	27.293849
N	11.271176	5.162550	29.659058
C	11.224642	7.196658	27.651368
C	8.492309	5.190797	26.733373
C	10.138771	3.365064	27.182497
C	11.781282	5.167075	30.844944
C	8.593103	6.835878	28.525463
N	11.503670	8.220982	27.166342
Si	6.285564	7.126276	26.803931
C	7.879095	6.303080	27.435697
N	7.950359	4.686888	25.669807
C	11.357337	3.006443	26.585299
H	12.218952	4.994351	26.516460
H	12.187524	4.145547	24.967620
H	13.355727	3.672141	26.216993
H	12.603321	1.371016	25.986202
C	9.305144	2.381383	27.747745
H	7.419032	3.416479	28.062206
H	8.460505	3.343894	29.465889
H	7.591429	1.889317	28.949096
H	8.964075	0.277100	27.967790
N	11.457480	5.905650	31.966125
N	12.820255	4.355203	31.258467

O	8.345851	7.822473	29.251254
C	11.822780	9.410521	26.566378
Si	4.553583	5.549264	26.394181
H	2.653252	6.482847	27.759856
H	2.082621	5.508293	26.397234
H	2.629648	7.170318	26.126808
H	3.782510	3.489604	27.558977
H	4.880660	4.412220	28.605617
H	5.530933	3.425103	27.287199
Si	6.806428	8.326586	24.827465
H	4.576946	9.387934	24.344922
H	5.900562	10.280709	23.584264
H	5.581616	10.476141	25.316848
H	9.252506	8.380707	25.397556
H	8.448248	9.875681	25.884929
H	8.868570	9.615896	24.179818
H	5.791408	7.189978	22.861071
H	7.199146	6.274690	23.395968
H	7.413646	7.776163	22.466929
C	8.622139	3.884207	24.743973
C	12.330341	4.015140	26.046482
C	11.667135	1.651074	26.463275
H	11.040024	-0.380830	26.776451
C	8.126269	2.773542	28.590059
C	9.628722	1.038898	27.566064
C	10.501449	6.968949	32.023104
C	12.285002	5.550977	33.046921
H	12.701020	5.610601	35.128878
H	11.087828	6.204799	34.694887
H	12.499600	7.214949	34.405098
C	13.134171	4.593066	32.602030
H	15.196509	4.051075	32.752685
H	14.087942	2.793333	33.297196
H	14.355726	4.229952	34.302146
C	13.460129	3.395941	30.415022
Si	5.451667	8.756418	28.328241
H	2.954834	8.737494	27.954768
H	3.747777	9.613396	26.642206
H	3.571930	10.370237	28.228757
H	7.453455	10.048755	29.052363
H	5.976580	11.046072	29.121848
H	6.775679	10.763016	27.569395
H	6.088166	7.771205	30.505547
H	4.509044	7.133046	29.997138
H	4.620151	8.766174	30.681363
C	11.184143	10.583591	27.011297
H	9.503228	11.405429	28.047828
H	10.704089	10.661346	29.103950
H	9.612487	9.640925	28.180978
H	11.038359	12.688126	26.657947
C	12.757352	9.381053	25.511743
H	13.942258	7.626637	25.906177
H	14.070107	8.250498	24.249694
H	12.623529	7.365541	24.769100
H	13.762294	10.604600	24.073314
C	2.823179	6.259376	26.701704
C	4.707024	4.080570	27.575318
C	5.599608	9.750638	24.497131
C	8.508384	9.122345	25.091376
C	6.813067	7.286324	23.244092
C	8.143807	2.587766	24.448712
H	6.123455	2.706179	25.175017
H	7.221527	1.843651	26.247621
H	6.664514	1.065325	24.758616
H	8.416894	0.831087	23.259246
C	9.671753	4.430393	23.967355
H	10.493020	6.036143	25.170320
H	9.272710	6.542292	24.014380
H	10.891661	6.113518	23.438840
H	11.075751	4.076470	22.389008
C	10.797316	0.669303	26.912660
C	9.143134	6.650383	32.158509
H	9.326260	4.664888	31.431692
C	10.982091	8.290569	32.027099
H	10.368856	10.346520	32.195067
H	13.032200	7.800046	32.353242
C	12.138209	6.179619	34.385847
C	14.247652	3.877860	33.274489
C	13.053965	2.054368	30.500325
H	13.339627	0.105335	29.640917
H	12.029447	2.318035	32.346122
C	14.473940	3.826593	29.550445
H	15.815333	3.180095	27.996592
H	14.485020	5.792365	30.371177
C	3.774743	9.420122	27.718262
C	6.527566	10.301817	28.532160
C	5.132737	8.033103	30.045223
C	10.196600	10.565552	28.135519
C	11.519516	11.763661	26.351350
H	12.676802	12.703351	24.808207
C	13.384062	8.089145	25.083555
C	13.050804	10.590028	24.893847
C	6.974190	2.019040	25.195503
C	8.776633	1.837461	23.461291
H	10.322397	1.750268	21.963360
C	10.109692	5.854208	24.160028
C	10.268975	3.647611	22.980589
C	8.662939	5.208938	32.115779
H	9.812168	4.486845	33.839360
H	8.387085	3.514752	33.448279
H	8.189819	5.091432	34.238586
H	6.493694	5.470890	32.261571
H	7.006813	4.009581	31.420328
H	7.128992	5.582670	30.606937
C	10.045394	9.310798	32.187006
C	12.463306	8.589649	31.847086
H	12.611489	7.560765	29.925179
H	13.946164	8.683121	30.256914
H	12.351614	9.307308	29.794604
H	12.491204	10.775224	31.898415
H	13.986650	10.008966	32.429133
H	12.571089	10.019014	33.499643
C	13.643060	1.146920	29.622294
H	15.048180	0.838921	28.029289
C	11.997446	1.620204	31.500261
H	10.494329	1.016582	30.053525
H	10.424634	2.722229	30.473547
H	9.828600	1.505850	31.631206
H	11.541636	0.017512	32.876802
H	13.261446	0.102148	32.443455
H	12.076643	-0.556745	31.299948
C	15.033676	2.880423	28.689643
C	14.989970	5.253398	29.560248
H	16.743937	4.762134	30.770163
H	16.833979	6.334041	29.949145
H	17.071787	4.839532	29.033765
H	15.143263	5.489965	27.396636
H	15.018419	7.018007	28.292810
H	13.584947	5.987164	28.078264
C	12.438648	11.769886	25.309329
C	9.844487	2.348799	22.733184
C	8.774751	4.538606	33.494281
C	7.240063	5.069495	31.567230
C	8.698301	9.019173	32.358819
C	12.866320	8.528265	30.366599
C	12.895418	9.924439	32.458028
C	14.610420	1.559807	28.714063
C	10.599108	1.723542	30.882054
C	12.242078	0.215285	32.058766
C	16.497562	5.296399	29.846850
C	14.664288	5.981250	28.251519

Calculated energies and coordinates of **TS14**

Electronic energy ... -4422.20150629 Eh
Total Enthalpy ... -4420.66360990 Eh
Final Gibbs free energy ... -4420.86424590 Eh

CARTESIAN COORDINATES (ANGSTROEM)

C	-7.085697	0.834165	-0.934525
H	-7.149981	1.927676	-0.920533

H	-7.847553	0.443347	-0.248050
H	-7.340934	0.493591	-1.943440
C	-2.657992	0.642858	3.892549
H	-3.532322	2.560844	4.279023
H	-3.618365	0.141083	3.889946
Si	0.243619	0.084249	0.008682
N	-0.069712	-1.000862	-1.330311
N	1.109537	-0.608744	1.229745
C	1.357643	1.466967	-0.719318
C	-1.280980	-0.606995	-1.988081
C	0.401622	-2.342413	-1.494700
C	1.509302	-0.643647	2.457633
C	-1.425019	0.909088	-0.140446
N	1.887557	2.387692	-1.220037
Si	-3.675361	1.200951	-1.707599
C	-2.072388	0.359051	-1.228939
N	-1.655631	-0.997603	-3.154991
C	1.727995	-2.589913	-1.883394
H	2.840003	-0.908312	-1.146263
H	2.316463	-0.769403	-2.838596
H	3.666042	-1.836901	-2.405158
H	3.156206	-4.091569	-2.418624
C	-0.475189	-3.412071	-1.234354
H	-2.563579	-2.865334	-1.427180
H	-1.829484	-2.400174	0.097309
H	-2.223761	-4.104952	-0.207388
H	-0.719868	-5.539462	-1.302611
N	0.941159	-0.120678	3.601982
N	2.653633	-1.276520	2.908133
O	-1.856915	1.985851	0.519578
C	2.586661	3.402372	-1.818583
Si	-5.372599	0.232734	-0.393627
H	-4.111079	0.826750	1.684522
H	-5.574380	-0.105407	2.083931
H	-5.689587	1.614717	1.677409
H	-6.242405	-2.073108	0.040838
H	-4.472865	-2.086633	-0.076913
H	-5.449034	-1.998767	-1.547260
Si	-4.384876	1.053014	-3.950135
H	-6.482129	2.337670	-3.379527
H	-6.155493	2.384435	-5.120574
H	-5.230712	3.413710	-4.012591
H	-2.389301	0.551123	-5.377858
H	-2.517393	2.302443	-5.088220
H	-3.595968	1.515206	-6.248911
H	-6.075855	-0.796291	-3.734252
H	-4.513288	-1.411197	-4.302506
H	-5.594834	-0.560884	-5.424214
C	-0.773191	-1.553932	-4.084830
C	2.690874	-1.462660	-2.080988
C	2.134372	-3.904718	-2.098829
H	1.573845	-5.981985	-2.132422
C	-1.845152	-3.186237	-0.664904
C	-0.037565	-4.712441	-1.487732
C	-0.280715	0.617399	3.681609
C	1.717555	-0.433412	4.731685
H	1.925860	-0.537139	6.842199
H	0.263794	-0.257711	6.291588
H	1.429566	1.060950	6.261944
C	2.778420	-1.154752	4.297000
H	3.850240	-1.541524	6.090015
H	4.878086	-1.381360	4.656139
H	3.939015	-2.854507	4.899829
C	3.499944	-2.076389	2.081520
Si	-2.694977	3.385949	-0.298980
H	-5.091200	3.440703	0.464960
H	-4.981533	3.892563	-1.235981
H	-4.538728	5.066915	0.044701
H	-1.328787	4.926789	-1.769327
H	-2.472025	3.942362	-2.715228
H	-0.977997	3.219321	-2.129831
H	-0.995443	4.367940	1.363058
H	-2.637226	4.395563	2.001127
H	-2.157054	5.589450	0.789425
C	2.299854	4.741468	-1.465915
H	1.727403	5.118956	0.565630

H	0.456243	4.368867	-0.403774
H	0.850879	6.076258	-0.630174
H	2.810468	6.777539	-1.854563
C	3.583163	3.069595	-2.770132
H	4.014494	1.028424	-2.233161
H	4.807131	1.593449	-3.714417
H	3.082740	1.201146	-3.714509
H	5.035865	3.881260	-4.104916
C	-5.166842	0.682474	1.437638
C	-5.385013	-1.660829	-0.507567
C	-5.688301	2.424321	-4.128451
C	-3.088040	1.387967	-5.284161
C	-5.225345	-0.585188	-4.390982
C	-1.011482	-2.826733	-4.639356
H	-3.005257	-3.016732	-3.857540
H	-1.914586	-4.315504	-3.368569
H	-2.542112	-4.280847	-5.021431
H	-0.305560	-4.305057	-6.017038
C	0.253575	-0.733381	-4.610937
H	0.746485	0.792785	-3.148336
H	-0.594952	1.200477	-4.191790
H	1.054541	1.248546	-4.838069
H	1.888750	-0.623415	-5.991248
C	1.251355	-4.963628	-1.934356
C	-1.493359	-0.087185	3.659456
H	-0.758002	-1.819939	2.673773
C	-0.202672	2.003519	3.888481
H	-1.389156	3.760304	4.255606
H	1.867713	2.136976	4.402606
C	1.315206	-0.017524	6.100858
C	3.918925	-1.765146	5.023811
C	3.189667	-3.436388	1.935054
H	3.740054	-5.230797	0.887821
H	1.734684	-3.389734	3.492543
C	4.613407	-1.482778	1.472792
H	6.232733	-1.843726	0.101653
H	4.389042	0.318055	2.583856
C	-4.501460	4.011430	-0.259706
C	-1.774745	3.929044	-1.870836
C	-2.041475	4.540504	1.091280
C	1.275574	5.088340	-0.433027
C	3.024505	5.742117	-2.104918
H	4.545870	6.242272	-3.537616
C	3.889429	1.648584	-3.124564
C	4.270536	4.116162	-3.370719
C	-2.184751	-3.653692	-4.199055
C	-0.135776	-3.310747	-5.609685
H	1.599639	-2.941796	-6.827923
C	0.377887	0.700260	-4.175290
C	1.099096	-1.254171	-5.587156
C	-1.534076	-1.587244	3.413611
H	-0.204482	-2.174267	5.057655
H	-1.283417	-3.456147	4.491782
H	-1.929076	-2.135509	5.484939
H	-3.676781	-2.007008	3.560872
H	-2.778134	-3.103134	2.510158
H	-3.157355	-1.462381	1.955100
C	-1.398292	2.687626	4.101488
C	1.134482	2.726439	3.836442
H	1.871804	1.812189	1.987524
H	2.548971	3.410741	2.324300
H	0.870029	3.246969	1.750626
H	0.490346	4.812366	3.848805
H	2.109988	4.536929	4.494594
H	0.690610	4.114119	5.468683
C	3.972522	-4.183453	1.055622
H	5.631041	-4.197452	-0.306275
C	2.049588	-4.080521	2.700795
H	1.091753	-5.027593	0.993319
H	0.532923	-3.387369	1.300194
H	-0.001757	-4.716479	2.358844
H	1.675483	-5.756252	4.022589
H	3.376597	-5.246627	3.996272
H	2.699281	-6.168653	2.645317
C	5.371160	-2.272066	0.606474
C	5.002971	-0.042498	1.749541

H	6.709856	-0.594470	3.001129
H	6.693988	1.103865	2.481737
H	7.148621	-0.166336	1.342095
H	5.304133	0.536175	-0.323633
H	4.944421	1.898279	0.757479
H	3.649628	0.784018	0.271090
C	3.997952	5.442304	-3.049572
C	0.930448	-2.547680	-6.068893
C	-1.214137	-2.380634	4.690720
C	-2.865886	-2.058265	2.825473
C	-2.609970	2.011534	4.112867
C	1.639305	2.801758	2.387660
C	1.096147	4.126290	4.451616
C	5.039784	-3.602029	0.383854
C	0.843645	-4.315794	1.786632
C	2.481862	-5.386542	3.380298
C	6.473896	0.077335	2.169494
C	4.707204	0.850422	0.540396

Calculated energies and coordinates of **INT15**

Electronic energy ... -4422.21097638 Eh
Total Enthalpy ... -4420.67164377 Eh
Final Gibbs free energy ... -4420.87318708 Eh

CARTESIAN COORDINATES (ANGSTROEM)

C	-5.392234	-2.843590	-0.831147
H	-5.831259	-3.078903	-1.806042
H	-6.023982	-3.301749	-0.058552
H	-4.403074	-3.313209	-0.790851
C	-0.752293	2.998438	4.411377
H	-0.228290	5.049071	4.759509
H	-1.793142	3.158600	4.670190
Si	0.158239	0.310711	-0.099642
N	0.293180	-0.564775	-1.626193
N	0.830723	-0.288707	1.273086
C	1.029300	1.931051	-0.513561
C	-0.942850	-0.511776	-2.329265
C	1.224197	-1.586098	-2.007126
C	1.392814	-0.550594	2.396694
C	-1.652818	0.567220	-0.329987
N	1.330355	3.029927	-0.832673
Si	-3.810184	-0.069255	-2.165905
C	-2.077967	-0.022653	-1.514437
N	-1.099947	-0.803993	-3.573275
C	2.493795	-1.258099	-2.504226
H	3.224026	0.539712	-1.575196
H	2.153971	0.801147	-2.956944
H	3.820390	0.263048	-3.225412
H	4.297106	-2.024299	-3.371039
C	0.819663	-2.930360	-1.905432
H	-1.340466	-3.057853	-2.021484
H	-0.713977	-2.749704	-0.412162
H	-0.558402	-4.368950	-1.115689
H	1.361705	-4.961653	-2.308852
N	1.555608	0.262653	3.509406
N	1.977891	-1.748189	2.779385
O	-2.465910	1.335939	0.461516
C	1.757555	4.303379	-1.057234
Si	-5.275995	-0.968060	-0.553643
H	-7.103011	0.761628	-0.461592
H	-7.712390	-0.850446	-0.045110
H	-7.417852	-0.418403	-1.737216
H	-5.338421	-1.366846	1.893069
H	-4.797685	0.294686	1.572162
H	-3.671788	-1.041619	1.362204
Si	-4.229144	-1.078659	-4.243477
H	-6.646930	-1.566878	-3.691571
H	-6.406669	-1.366418	-5.431164
H	-6.478382	0.057065	-4.378185
H	-2.427689	-0.264187	-5.799480
H	-3.740272	0.920277	-5.660881
H	-3.964869	-0.531444	-6.648898
H	-4.481465	-3.511903	-3.740855
H	-2.782057	-3.118731	-4.049040
H	-3.916145	-3.266666	-5.403838
C	-0.062704	-0.710569	-4.510524
C	2.950097	0.163659	-2.570498
C	3.326926	-2.281870	-2.952944
H	3.574125	-4.393650	-3.273877
C	-0.518394	-3.299439	-1.336038
C	1.681603	-3.924340	-2.366710
C	1.038982	1.578112	3.712206
C	2.211762	-0.428999	4.544663
H	2.830055	-0.553663	6.570240
H	1.587040	0.675031	6.271870
H	3.256251	0.980334	5.796707
C	2.459785	-1.679010	4.092826
H	3.346398	-2.601965	5.787529
H	3.924758	-3.250036	4.244354
H	2.320342	-3.684068	4.827819
C	1.938285	-2.965277	2.035361
Si	-3.131960	2.844849	0.080628
H	-5.510221	2.315055	0.615732
H	-5.263405	2.365770	-1.142160
H	-5.335079	3.873479	-0.196477
H	-2.618736	4.583970	-1.596384
H	-2.525216	2.958969	-2.303640
H	-1.183026	3.576608	-1.331305
H	-1.908030	3.745830	2.094483
H	-3.652911	3.659572	2.368743
H	-2.990463	4.956266	1.375581
C	1.362997	5.310231	-0.139639
H	0.369625	5.814664	1.691197
H	0.714592	4.087029	1.523365
H	-0.583215	4.815085	0.584826
H	1.538743	7.379503	0.349057
C	2.587306	4.593364	-2.168930
H	3.639306	2.772876	-2.600782
H	3.603070	3.938051	-3.934728
H	2.165919	2.980892	-3.538612
H	3.644462	6.144666	-3.181562
C	-7.040428	-0.300599	-0.716279
C	-4.716985	-0.742435	1.237884
C	-6.118319	-0.975499	-4.447099
C	-3.512346	-0.149598	-5.724690
C	-3.806424	-2.920500	-4.367708
C	0.392376	-1.812094	-5.260589
H	-0.517306	-3.373423	-4.072995
H	0.501026	-3.953021	-5.396864
H	-1.100935	-3.272687	-5.734444
H	1.770030	-2.470084	-6.759067
C	0.385842	0.593465	-4.835495
H	-0.053470	1.871620	-3.122735
H	-1.354715	1.718925	-4.283947
H	0.064170	2.715001	-4.679723
H	1.709626	1.756872	-6.054000
C	2.922210	-3.608372	-2.900924
C	-0.319658	1.722967	4.043863
H	-1.008304	-0.102333	3.200322
C	1.950408	2.647194	3.735220
H	2.139151	4.749269	4.154919
H	3.695431	1.429745	3.589522
C	2.486068	0.202249	5.861323
C	3.045276	-2.863290	4.771392
C	0.787870	-3.759567	2.122374
H	-0.091889	-5.602651	1.444006
H	-0.081622	-2.532176	3.630712
C	3.098022	-3.365278	1.349258
H	3.914290	-4.924551	0.114320
H	4.423920	-2.098574	2.415479
C	-4.984618	2.832397	-0.193569
C	-2.272986	3.558089	-1.421426
C	-2.883404	3.892862	1.623473
C	0.424380	4.989965	0.976836
C	1.833361	6.599633	-0.348209
H	2.996202	7.924071	-1.583972
C	3.019665	3.517236	-3.112612
C	3.008352	5.906687	-2.333261
C	-0.210786	-3.177226	-5.099941
C	1.392872	-1.610042	-6.210143

H	2.678005	-0.210519	-7.222661
C	-0.263592	1.789637	-4.196326
C	1.369572	0.752669	-5.808161
C	-1.264936	0.529687	4.059703
H	-0.096760	-0.740705	5.430132
H	-1.819457	-1.127355	5.345186
H	-1.284342	0.313632	6.227082
H	-3.114672	1.449228	4.781407
H	-3.338023	0.011546	3.781134
H	-2.887094	1.531159	3.012901
C	1.466935	3.898027	4.120729
C	3.400962	2.453089	3.326695
H	2.915934	1.847140	1.284687
H	4.586068	2.408310	1.501841
H	3.255200	3.580729	1.467516
H	4.248549	4.438460	3.681364
H	5.397899	3.112339	3.825714
H	4.220633	3.403297	5.121174
C	0.786409	-4.963661	1.414665
H	1.880391	-6.301210	0.146185
C	-0.387561	-3.380480	3.006544
H	-1.962567	-3.732120	1.535543
H	-1.385648	-2.054522	1.586241
H	-2.434143	-2.673743	2.875064
H	-1.519259	-4.185684	4.672804
H	0.106019	-4.884261	4.519262
H	-1.184596	-5.377873	3.413334
C	3.048801	-4.578743	0.668716
C	4.356925	-2.513743	1.401237
H	5.702147	-4.194285	1.801275
H	6.509845	-2.682528	1.341840
H	5.701653	-3.651313	0.111010
H	4.290183	-1.674707	-0.597076
H	5.148200	-0.662561	0.588408
H	3.371450	-0.738824	0.584035
C	2.647145	6.907236	-1.435043
C	1.901537	-0.344723	-6.475257
C	-1.095215	-0.304725	5.341549
C	-2.735140	0.916603	3.901462
C	0.131621	4.068548	4.459971
C	3.545888	2.577595	1.802344
C	4.365073	3.408360	4.035410
C	1.899021	-5.360928	0.690276
C	-1.611546	-2.932995	2.198767
C	-0.763006	-4.526267	3.957345
C	5.636547	-3.314810	1.151594
C	4.285100	-1.321634	0.437831

Calculated energies and coordinates of **INT16** open-shell singlet

Electronic energy ... -4019.19648880 Eh
Total Enthalpy ... -4017.82356401 Eh
Final Gibbs free energy ... -4018.00940346 Eh

CARTESIAN COORDINATES (ANGSTROEM)

C	-3.239744	0.023446	-5.931335
H	-2.512090	0.684990	-6.414553
H	-3.982025	-0.262341	-6.687810
H	-2.713514	-0.876625	-5.602025
C	-1.227756	2.052135	5.255627
H	-2.098452	4.011330	5.154741
H	-1.850852	1.746916	6.090275
Si	-0.625578	-0.322545	0.545272
N	-0.725676	-1.307681	-0.947048
N	0.939405	-0.117307	1.088857
C	-1.529824	-0.707272	-1.917779
C	-0.580188	-2.736237	-0.908352
C	1.731523	0.037782	2.089831
C	-1.610213	1.038074	-0.286734
Si	-2.707797	1.776486	-2.839003
C	-1.986057	0.645358	-1.538118
N	-1.899401	-1.175501	-3.063045
C	0.624190	-3.340562	-1.291137
H	1.801589	-1.541565	-1.290478
H	1.810706	-2.431730	-2.823714
H	2.731569	-3.041032	-1.444216
H	1.638026	-5.208646	-1.565050
C	-1.657011	-3.504078	-0.427084
H	-3.322636	-2.129895	-0.647959
H	-2.718576	-2.288520	0.997516
H	-3.683037	-3.597156	0.285539
H	-2.364196	-5.493749	-0.048308
N	1.596895	0.794281	3.247749
N	3.000853	-0.510928	2.202007
O	-1.985325	2.245499	0.240617
Si	-4.099127	0.899197	-4.500531
H	-5.568342	2.941035	-4.462451
H	-5.794226	2.014277	-5.957247
H	-4.373295	3.048890	-5.765885
H	-5.943068	-0.787102	-4.382229
H	-6.021278	0.342460	-3.015320
H	-4.825248	-0.967224	-3.013280
Si	-1.336425	3.657768	-3.095065
H	-2.925350	5.463435	-2.393994
H	-1.241704	6.005083	-2.249943
H	-1.933757	4.918387	-1.029765
H	0.818795	2.391332	-3.150969
H	0.381840	2.816202	-1.491670
H	1.076602	4.049817	-2.566619
H	-2.273207	4.461595	-5.275277
H	-0.943458	3.324389	-5.549290
H	-0.599174	4.996481	-5.066605
C	-1.267897	-2.195548	-3.776388
C	1.807469	-2.538112	-1.733747
C	0.710691	-4.733933	-1.252548
H	-0.277694	-6.590984	-0.813510
C	-2.915223	-2.848194	0.072285
C	-1.530522	-4.892142	-0.402462
C	0.491537	1.605028	3.646526
C	2.759161	0.708471	4.036379
H	2.225778	1.066868	6.091015
H	2.663587	2.514146	5.186023
H	3.922467	1.368087	5.683515
C	3.629413	-0.093093	3.379438
H	5.716108	-0.193146	2.913707
H	5.109017	-1.591957	3.796576
H	5.338253	-0.050629	4.642187
C	3.646981	-1.355537	1.248784
Si	-3.477523	2.381050	1.035568
H	-4.816642	0.865169	-0.423175
H	-5.039484	2.552000	-0.911294
H	-5.844344	1.933488	0.547801
H	-2.728726	4.540751	2.027081
H	-4.484496	4.346519	2.137694
H	-3.745753	4.806171	0.597293
H	-2.506936	1.355308	3.078087
H	-3.533480	0.200310	2.213591
H	-4.270748	1.471810	3.205613
C	-5.046736	2.366581	-5.235465
C	-5.340715	-0.239307	-3.646974
C	-1.917228	5.153592	-2.098159
C	0.401307	3.185938	-2.522798
C	-1.284624	4.152679	-4.919047
C	-1.909937	-3.419209	-4.050636
H	-3.297536	-3.673487	-2.415764
H	-3.637288	-4.675490	-3.835165
H	-3.991093	-2.935207	-3.857186
H	-1.748290	-5.305061	-5.050246
C	-0.054091	-1.872837	-4.425481
H	0.797256	-0.263820	-3.239658
H	-0.211569	0.261474	-4.578165
H	1.420797	-0.380828	-4.896832
H	1.489701	-2.578428	-5.731923
C	-0.359033	-5.507671	-0.827678
C	-0.294053	1.161560	4.724807
H	0.848666	-0.606620	5.064318
C	0.337208	2.875252	3.065078
H	-0.747611	4.722049	3.228500
H	1.311348	2.456020	1.228710
C	2.898081	1.451097	5.314846
C	5.019245	-0.503175	3.701611

C	3.675341	-2.737778	1.501721
H	4.483010	-4.605362	0.804563
H	2.876687	-2.587489	3.467623
C	4.334736	-0.764299	0.180124
H	5.608158	-1.184945	-1.502504
H	3.893793	1.215169	0.828451
C	-4.923048	1.888648	-0.048116
C	-3.613555	4.187449	1.491295
C	-3.442542	1.247992	2.522707
C	-3.280919	-3.694390	-3.508880
C	-1.264527	-4.349363	-4.862381
H	0.454872	-4.809767	-6.073972
C	0.526429	-0.494531	-4.277032
C	0.551236	-2.826683	-5.240290
C	-0.173419	-0.257092	5.253905
H	0.165215	0.348873	7.331884
H	-0.173535	-1.377735	7.103390
H	-1.485164	-0.207187	7.005368
H	-2.163427	-0.876173	4.623087
H	-1.013715	-2.218100	4.819436
H	-0.916060	-1.154385	3.398485
C	-0.612431	3.725107	3.632879
C	1.205048	3.320013	1.897908
H	-0.435976	4.193395	0.761686
H	1.178301	4.625888	0.182202
H	0.551212	5.392876	1.639622
H	2.539454	4.563480	3.088935
H	3.197269	4.089952	1.512201
H	3.156665	2.916791	2.835203
C	4.430115	-3.533993	0.640826
H	5.707404	-3.608867	-1.083719
C	2.897780	-3.334046	2.662503
H	1.398875	-4.357247	1.456798
H	0.954006	-2.702563	1.873041
H	0.868317	-3.977339	3.109676
H	3.022984	-4.891873	4.154220
H	4.598537	-4.460531	3.459989
H	3.446686	-5.446649	2.537861
C	5.067246	-1.605690	-0.659922
C	4.296703	0.731766	-0.069985
H	6.390111	1.041866	0.487138
H	5.637045	2.409202	-0.357980
H	6.118208	0.972899	-1.262017
H	3.692335	0.576863	-2.159748
H	3.331259	2.146848	-1.413014
H	2.335995	0.731569	-1.024141
C	-0.031453	-4.072610	-5.442043
C	-0.430393	-0.370563	6.759615
C	-1.121633	-1.183419	4.475209
C	-1.373393	3.326097	4.724257
C	0.577735	4.450466	1.080122
C	2.608699	3.740177	2.367952
C	5.123648	-2.971712	-0.425055
C	1.441103	-3.605816	2.252279
C	3.536763	-4.603758	3.231476
C	5.694173	1.316472	-0.312621
C	3.355155	1.065234	-1.237624

Calculated energies and coordinates of **TS15** open-shell singlet

Electronic energy ... -4019.18584188 Eh
Total Enthalpy ... -4017.81427650 Eh
Final Gibbs free energy ... -4017.99898321 Eh

CARTESIAN COORDINATES (ANGSTROEM)

C	-3.385405	3.616148	1.763899
H	-2.360025	3.347669	1.488738
H	-3.371765	4.054368	2.770156
H	-3.721763	4.387955	1.063817
C	0.509129	3.879857	2.137355
H	0.074612	5.876473	1.488266
H	-0.274410	3.890598	2.886472
Si	0.410004	0.130390	-0.871293
N	-0.624789	-1.275353	-0.353275
N	1.877612	-0.039186	-0.093955
C	-1.920146	-0.856343	-0.196318
C	-0.200166	-2.591775	-0.009478
C	2.977961	0.470213	0.319189
C	-1.007663	1.323936	-0.528199
Si	-3.995540	0.748571	-0.104123
C	-2.139290	0.579636	-0.323423
N	-3.045245	-1.496591	0.062334
C	0.483700	-3.392688	-0.938874
H	1.914563	-2.352158	-2.135919
H	0.262613	-2.154805	-2.714747
H	1.117578	-3.693123	-2.967047
H	1.275181	-5.346465	-1.317450
C	-0.462383	-3.068740	1.289652
H	-0.231145	-1.288062	2.441127
H	-0.932206	-2.651053	3.338705
H	-1.912264	-1.718354	2.191326
H	-0.429834	-4.782049	2.573280
N	3.236463	1.705869	0.898731
N	4.207142	-0.174670	0.337727
O	-0.990936	2.670747	-0.748419
Si	-4.536168	2.112498	1.720559
H	-3.308844	0.735403	3.424259
H	-5.037373	0.364328	3.450585
H	-4.442362	1.858148	4.199992
H	-6.519249	3.269291	0.715556
H	-6.570095	3.330265	2.487628
H	-7.026040	1.845251	1.638706
Si	-5.510610	0.043866	-1.744702
H	-5.202746	-0.585216	-4.140146
H	-3.654744	-0.655587	-3.280456
H	-4.320619	0.907600	-3.774737
H	-6.838040	-1.598008	-0.399143
H	-5.570810	-2.425146	-1.309235
H	-7.042721	-1.874600	-2.137732
H	-7.557678	1.038828	-2.751924
H	-6.495052	2.322807	-2.146489
H	-7.495278	1.394706	-1.018798
C	-3.305497	-2.865324	0.079577
C	0.956615	-2.869475	-2.264591
C	0.765630	-4.716016	-0.592705
H	0.592820	-6.277144	0.875664
C	-0.919951	-2.137843	2.373126
C	-0.193643	-4.403392	1.582003
C	2.309429	2.787813	1.004135
C	4.592104	1.805060	1.262096
H	6.158438	2.822328	2.272174
H	4.534452	3.265279	2.827980
H	5.136169	3.891765	1.296812
C	5.190002	0.640199	0.908355
H	7.217035	0.970153	1.460454
H	7.010443	-0.084662	0.051495
H	6.683029	-0.708408	1.667171
C	4.418863	-1.464032	-0.235780
Si	-1.554088	3.439424	-2.139473
H	-3.770172	4.239088	-1.314208
H	-3.939990	2.701255	-2.166094
H	-3.700674	4.202127	-3.085716
H	-1.304180	2.954063	-4.555749
H	-1.501690	1.456789	-3.627291
H	0.062258	2.276457	-3.641631
H	-1.185462	5.745493	-2.934214
H	0.297310	5.121473	-2.184351
H	-1.046925	5.656443	-1.169153
C	-4.309197	1.175767	3.350714
C	-6.331764	2.692593	1.627359
C	-4.581557	-0.083372	-3.387691
C	-6.311410	-1.620790	-1.359530
C	-6.890430	1.324718	-1.929112
C	-4.051072	-3.358916	1.177340
H	-4.928782	-1.487591	1.770659
H	-3.820845	-2.143883	2.964264
H	-5.411047	-2.887997	2.765391
H	-4.872421	-5.099642	2.116001
C	-2.979799	-3.731975	-0.989158
H	-1.955391	-2.270836	-2.241854
H	-3.331566	-3.079002	-2.989628

H	-1.807076	-3.949258	-2.778793
H	-2.964265	-5.763113	-1.662664
C	0.395802	-5.235326	0.639448
C	1.311790	2.747904	1.989864
H	1.326070	0.644935	2.271914
C	2.486225	3.889378	0.148696
H	1.795174	5.882957	-0.269835
H	4.332375	3.225077	-0.699887
C	5.132916	3.007890	1.946166
C	6.596338	0.181331	1.031397
C	4.418319	-2.584327	0.610346
H	4.561857	-4.729185	0.643696
H	4.530470	-1.426351	2.391927
C	4.611069	-1.563384	-1.620877
H	4.906677	-2.956001	-3.233698
H	4.590055	0.550314	-1.891707
C	-3.414849	3.661597	-2.173688
C	-1.020390	2.441389	-3.629127
C	-0.792227	5.146431	-2.104625
C	-4.581099	-2.422291	2.225560
C	-4.315817	-4.722595	1.261109
H	-4.081184	-6.662539	0.357235
C	-2.479170	-3.223387	-2.312105
C	-3.246689	-5.092805	-0.853837
C	1.137609	1.533845	2.886665
H	2.049867	2.446995	4.641700
H	3.188383	1.503922	3.655293
H	2.010551	0.672194	4.680624
H	-0.530505	2.208691	4.139354
H	-0.380419	0.461525	3.989003
H	-1.021569	1.403445	2.629375
C	1.674577	5.003844	0.354115
C	3.472024	3.831855	-1.007039
H	1.983186	3.700816	-2.588150
H	2.451698	2.125935	-1.935387
H	3.555161	3.001375	-3.019426
H	4.405640	5.762575	-0.574553
H	3.230563	5.813977	-1.900942
H	4.810408	5.078418	-2.160664
C	4.574675	-3.838948	0.022978
H	4.847503	-4.953436	-1.791687
C	4.205042	-2.433864	2.104814
H	2.533933	-2.378715	3.499826
H	2.335685	-3.539408	2.166344
H	2.128837	-1.818135	1.856612
H	4.945214	-3.183478	3.993860
H	6.077074	-3.430059	2.648229
H	4.640270	-4.456040	2.814592
C	4.764536	-2.841432	-2.162405
C	4.681474	-0.343150	-2.520451
H	6.115605	0.661332	-3.808589
H	6.171160	-1.103071	-3.929219
H	6.868532	-0.297071	-2.517209
H	2.559260	-0.268581	-3.034353
H	3.546361	-1.202857	-4.177283
H	3.621090	0.565711	-4.183245
C	-3.885613	-5.597735	0.270944
C	2.161970	1.542511	4.032333
C	-0.283311	1.402994	3.438897
C	0.701581	5.000861	1.343826
C	2.828294	3.118185	-2.207150
C	4.006882	5.202758	-1.427445
C	4.735450	-3.966482	-1.351340
C	2.710247	-2.545069	2.430337
C	5.018621	-3.434867	2.930619
C	6.040167	-0.267725	-3.232868
C	3.531965	-0.313898	-3.536015

Calculated energies and coordinates of **INT17**

Electronic energy ... -4019.21871888 Eh
Total Enthalpy ... -4017.84569519 Eh
Final Gibbs free energy ... -4018.03164798 Eh

CARTESIAN COORDINATES (ANGSTROEM)

C	-3.953193	-0.002146	-5.815583
H	-3.245870	0.570150	-6.426664
H	-4.874942	-0.120611	-6.398757
H	-3.524103	-0.997007	-5.651021
C	-1.425679	2.153502	5.209451
H	-2.141416	4.162243	4.960691
H	-2.141066	1.922340	5.993519
Si	-0.833355	-0.464899	0.962090
N	-0.739995	-1.353440	-0.759666
N	0.849030	-0.271043	1.285368
C	-1.258010	-0.576741	-1.696839
C	-0.167608	-2.634456	-1.011358
C	1.613634	-0.131540	2.298086
C	-1.502639	1.055971	-0.023302
Si	-2.219110	1.025334	-3.115317
C	-1.760096	0.725238	-1.319937
N	-1.498159	-0.675163	-3.056604
C	0.999474	-2.728395	-1.787371
H	1.386018	-0.603097	-1.820342
H	1.494412	-1.406931	-3.402048
H	2.777690	-1.622405	-2.203545
H	2.423790	-4.064103	-2.667076
C	-0.757702	-3.784847	-0.453366
H	-2.585798	-2.875055	0.293320
H	-1.590871	-3.621265	1.520429
H	-2.517020	-4.652938	0.417416
H	-0.673807	-5.922865	-0.359215
N	1.455100	0.600290	3.484495
N	2.895935	-0.677232	2.431534
O	-1.893597	2.237825	0.534165
Si	-4.325425	0.872148	-4.180751
H	-5.270232	3.133077	-3.638733
H	-5.924379	2.504472	-5.157505
H	-4.298128	3.199797	-5.120018
H	-6.414971	-0.435622	-3.894071
H	-6.058770	0.491194	-2.426126
H	-5.182792	-1.010422	-2.759244
Si	-0.898535	2.892706	-3.716291
H	-2.811288	4.437165	-3.135544
H	-1.241233	5.214191	-2.873745
H	-1.838770	4.020674	-1.706264
H	1.437455	1.964186	-3.464757
H	0.740881	2.450580	-1.912169
H	1.358863	3.679514	-3.033990
H	-1.774981	3.309337	-6.034675
H	-0.122216	2.676591	-6.091864
H	-0.407670	4.366840	-5.657573
C	-1.348283	-1.778553	-3.929046
C	1.696645	-1.516052	-2.330641
C	1.523484	-3.992047	-2.061562
H	1.326402	-6.117882	-1.807880
C	-1.933499	-3.728666	0.484366
C	-0.209608	-5.028344	-0.768577
C	0.398445	1.510958	3.786574
C	2.613109	0.509517	4.284973
H	2.084008	0.760774	6.356300
H	2.428880	2.264509	5.502929
H	3.754983	1.174236	5.947154
C	3.499443	-0.272272	3.628310
H	5.597157	-0.363955	3.205640
H	4.973856	-1.778869	4.051522
H	5.182855	-0.252926	4.927629
C	3.557479	-1.426583	1.417525
Si	-3.470540	2.611235	0.983923
H	-4.618453	1.220958	-0.717886
H	-4.354605	2.863001	-1.332288
H	-5.664907	2.548445	-0.176979
H	-2.768027	4.646874	2.217052
H	-4.441155	4.787771	1.651245
H	-3.101527	5.019334	0.515032
H	-3.288205	1.733582	3.292904
H	-3.868167	0.490618	2.181895
H	-4.984916	1.751817	2.756957
C	-5.012087	2.591130	-4.554507
C	-5.611612	-0.115395	-3.219307
C	-1.791086	4.270189	-2.772993

C	0.823944	2.723660	-2.969971
C	-0.794792	3.346610	-5.546863
C	-2.032107	-2.985169	-3.696819
H	-3.071504	-2.323142	-1.902143
H	-2.962907	-4.076058	-2.095653
H	-4.074973	-3.145193	-3.103126
H	-2.299645	-5.006076	-4.352197
C	-0.549821	-1.625555	-5.080968
H	0.482696	0.206586	-4.567496
H	-0.729716	0.376189	-5.833015
H	0.813512	-0.399978	-6.205484
H	0.275610	-2.605306	-6.796738
C	0.916802	-5.139272	-1.573808
C	-0.512583	1.178350	4.802748
H	0.363061	-0.731692	5.144204
C	0.383927	2.768533	3.151135
H	-0.567953	4.690935	3.138601
H	1.532728	2.194611	1.467691
C	2.723861	1.212026	5.588659
C	4.882648	-0.688173	3.972483
C	3.543458	-2.828277	1.495495
H	4.184288	-4.626569	0.504271
H	2.843585	-2.857426	3.499532
C	4.248609	-0.737037	0.410367
H	5.432575	-0.988824	-1.368501
H	3.950393	1.146064	1.349874
C	-4.636191	2.281092	-0.447220
C	-3.438949	4.436848	1.379803
C	-3.953627	1.552427	2.442564
C	-3.083253	-3.131868	-2.632686
C	-1.794451	-4.063687	-4.550573
H	-0.773053	-4.797831	-6.293366
C	0.044720	-0.293684	-5.435035
C	-0.356532	-2.719656	-5.919684
C	-0.558871	-0.206902	5.420948
H	0.119663	0.466535	7.396891
H	-0.526055	-1.186597	7.354296
H	-1.623681	0.191629	7.290227
H	-2.689651	-0.525047	5.100171
H	-1.743811	-2.021680	5.230563
H	-1.677115	-1.046415	3.739426
C	-0.545065	3.707269	3.595851
C	1.375120	3.108906	2.050685
H	-0.132760	3.913457	0.698332
H	1.545405	4.244273	0.224653
H	0.817019	5.166322	1.538099
H	2.596784	4.433657	3.272097
H	3.423389	3.806355	1.832897
H	3.187399	2.757618	3.239495
C	4.191247	-3.541344	0.487271
H	5.352033	-3.452972	-1.316696
C	2.832821	-3.533812	2.635573
H	1.295102	-4.465220	1.415413
H	0.852320	-2.861743	2.009966
H	0.839193	-4.253141	3.122213
H	3.063843	-5.216416	3.975269
H	4.591910	-4.686496	3.242343
H	3.414251	-5.614777	2.295198
C	4.892667	-1.492434	-0.571008
C	4.302095	0.778613	0.378522
H	6.421088	0.877827	0.909973
H	5.743111	2.395175	0.285411
H	6.109163	1.070078	-0.822660
H	3.650144	0.994393	-1.690936
H	3.375803	2.433358	-0.689341
H	2.329850	1.004531	-0.502561
C	-0.951011	-3.945544	-5.644475
C	-0.648295	-0.175047	6.952440
C	-1.737246	-0.999619	4.834008
C	-1.431876	3.410828	4.623817
C	0.860132	4.172559	1.076580
C	2.726359	3.547518	2.639056
C	4.852450	-2.879794	-0.540076
C	1.363820	-3.792115	2.276685
C	3.522406	-4.834874	3.056907
C	5.728393	1.305095	0.177182
C	3.354869	1.336904	-0.691751

Calculated energies and coordinates of **INT18**

Electronic energy ... -4422.22299367 Eh
Total Enthalpy ... -4420.68407548 Eh
Final Gibbs free energy ... -4420.88650477 Eh

CARTESIAN COORDINATES (ANGSTROEM)

C	4.234977	5.496462	24.321769
H	4.512280	4.528903	23.888051
H	3.358394	5.868827	23.776803
H	3.940988	5.316377	25.361194
C	10.264280	2.502299	32.988599
H	8.517361	2.706990	34.215018
H	10.207938	1.418555	32.977349
Si	9.762156	6.392436	28.932900
N	8.471586	5.110490	28.603500
N	11.162241	5.816819	29.597150
C	8.925128	7.556750	30.167997
C	8.104208	5.236797	27.308864
C	8.167470	3.894589	29.310679
C	12.203755	5.765321	30.331496
C	9.831020	6.786262	27.103743
N	8.750240	8.779320	30.331145
Si	7.565219	5.642137	25.102503
C	8.758332	6.177103	26.454524
N	7.135751	4.595150	26.572827
C	8.694552	2.696696	28.790621
H	9.111304	2.893216	26.681718
H	10.164957	1.759622	27.539921
H	10.374688	3.512957	27.727870
H	8.739397	0.561722	28.977561
C	7.362674	3.884706	30.460685
H	7.430337	6.007360	30.901717
H	6.682463	4.991457	32.153445
H	5.794276	5.333580	30.667399
H	6.420366	2.638234	31.921917
N	12.342468	5.228723	31.609174
N	13.498164	6.154598	29.995823
O	10.636775	7.759255	26.627725
C	8.947686	9.913060	29.537367
Si	5.646201	6.745892	24.194715
H	6.794906	7.814383	22.234155
H	5.070282	7.563329	21.935420
H	6.193600	6.207660	21.783912
H	4.278219	8.781280	24.331469
H	5.923794	9.133175	24.877290
H	4.777395	8.337561	25.968822
Si	8.545286	4.359758	23.341237
H	8.567650	6.151958	21.558888
H	9.706065	4.842161	21.219164
H	10.121723	6.085461	22.403599
H	10.574517	3.774293	24.701145
H	10.549129	2.909728	23.151561
H	9.538860	2.356877	24.494531
H	6.462782	3.898728	22.008443
H	6.747551	2.592758	23.164303
H	7.721078	2.698419	21.690838
C	5.963343	3.989141	27.105659
C	9.636139	2.713705	27.623868
C	8.339822	1.487881	29.385558
H	7.238483	0.511162	30.955019
C	6.792580	5.128952	31.073229
C	7.049846	2.650230	31.035505
C	11.299561	4.556036	32.316953
C	13.689351	5.231638	32.009149
H	13.447715	5.009906	34.124501
H	15.128760	5.044799	33.562640
H	14.139406	3.593184	33.341095
C	14.404944	5.792409	31.005782
H	16.283113	5.344415	30.077756
H	16.377831	5.774782	31.793140
H	16.106672	7.027368	30.563996
C	13.923513	6.728210	28.756923

Si	10.840908	8.383720	25.086278
H	8.794981	7.698518	23.799589
H	9.300780	9.341322	23.393356
H	8.455126	9.032734	24.922828
H	12.946432	7.188985	24.657132
H	12.102593	7.474253	23.119389
H	11.570708	6.175291	24.211180
H	11.048879	10.676562	25.990377
H	11.837184	10.548979	24.402214
H	12.637091	9.895443	25.844326
C	8.224930	10.119580	28.346261
H	7.579119	8.289286	27.355138
H	6.465215	9.661940	27.227811
H	6.612619	8.763495	28.749606
H	7.889040	11.471937	26.718395
C	9.792386	10.916779	30.051936
H	11.039692	9.833659	31.458314
H	9.638620	10.617448	32.165168
H	11.034940	11.600488	31.671386
H	10.662323	12.840630	29.689333
C	5.969887	7.106001	22.366097
C	5.113941	8.398764	24.931381
C	9.299269	5.479204	22.015017
C	9.928116	3.247083	23.990717
C	7.240724	3.289880	22.480404
C	5.074748	4.773700	27.868192
H	6.190081	6.411418	28.735171
H	4.461933	6.730278	28.509687
H	5.577754	6.726377	27.130071
H	3.247487	4.778279	28.986633
C	5.679169	2.642532	26.835962
H	7.248157	2.363782	25.377403
H	6.043832	1.057681	25.430524
H	7.262077	1.206224	26.706395
H	4.296593	1.038526	27.171238
C	7.509252	1.458729	30.497624
C	11.284891	3.151035	32.293715
H	13.229678	2.965826	31.436694
C	10.356723	5.314859	33.030002
H	8.606392	5.162378	34.268421
H	10.626995	7.175620	32.052455
C	14.123421	4.690080	33.324657
C	15.867547	5.998742	30.853883
C	14.351242	5.871909	27.732945
H	15.294313	5.798849	25.799702
H	13.718737	4.123838	28.772381
C	13.997332	8.128548	28.641860
H	14.704838	9.720585	27.381054
H	12.420780	8.604977	29.964827
C	9.192406	8.628595	24.219817
C	11.964340	7.197499	24.170857
C	11.667749	10.032829	25.354212
C	7.169643	9.153997	27.891997
C	8.447248	11.300928	27.637184
H	9.498607	13.175375	27.522098
C	10.419309	10.736358	31.404658
C	9.993854	12.073059	29.305704
C	5.335100	6.234542	28.076450
C	3.932845	4.176051	28.395074
H	2.756910	2.382707	28.567110
C	6.607172	1.775202	26.035196
C	4.515535	2.084939	27.369926
C	12.322259	2.357815	31.515621
H	12.981802	1.195267	33.249038
H	13.565081	0.593718	31.683001
H	11.893168	0.313431	32.164858
H	10.951649	1.469762	30.082335
H	12.628322	1.607923	29.496658
H	11.578632	3.043914	29.590845
C	9.360139	4.615723	33.712847
C	10.435803	6.832580	33.077382
H	8.288879	7.157465	32.915085
H	9.212698	8.573809	33.400956
H	8.920612	7.293155	34.584449
H	11.490452	6.920506	34.986422
H	11.575792	8.411597	34.030701
H	12.568666	7.023895	33.576271
C	14.949234	6.440309	26.606120
H	15.587517	8.243230	25.631472
C	14.102354	4.378662	27.778497
H	13.363819	4.220974	25.732377
H	12.121335	4.650233	26.923429
H	12.727638	2.976991	26.828220
H	15.137158	2.479682	27.642108
H	16.158563	3.796663	28.247636
H	15.760467	3.697841	26.525301
C	14.612211	8.647314	27.501739
C	13.393140	9.045653	29.695761
H	14.313752	8.190973	31.503289
H	13.775803	9.864033	31.663506
H	15.247381	9.503362	30.744686
H	14.066637	10.980293	28.940111
H	12.609208	11.038115	29.923203
H	12.502986	10.445648	28.267273
C	9.341386	12.263460	28.090670
C	3.653294	2.835906	28.154131
C	12.709647	1.042750	32.199025
C	11.843893	2.103777	30.082135
C	9.310541	3.228330	33.686151
C	9.131722	7.490748	33.526329
C	11.591670	7.317330	33.969028
C	15.104315	7.813071	26.504842
C	13.010396	4.033263	26.753563
C	15.365741	3.545775	27.535561
C	14.237291	9.145096	30.977254
C	13.130727	10.454530	29.162798

Calculated energies and coordinates of **TS16**

Electronic energy ... -4422.21227157 Eh
Total enthalpy ... -4420.67411546 Eh
Final Gibbs free energy ... -4420.87653697 Eh

CARTESIAN COORDINATES (ANGSTROEM)

C	-5.577857	-0.567266	-4.169527
H	-5.137228	-1.384046	-4.752859
H	-6.512885	-0.271624	-4.661760
H	-5.822433	-0.967092	-3.179486
C	0.132265	-2.790224	5.481857
H	-1.441630	-2.054952	6.741823
H	-0.030431	-3.825167	5.766570
Si	0.157786	0.292144	0.449160
N	-1.205278	-0.837893	0.051577
N	1.288291	-0.664377	1.236274
C	-0.567649	1.861374	0.902608
C	-1.704736	-0.558912	-1.186669
C	-1.464261	-2.105707	0.663816
C	2.336531	-0.605825	1.985624
C	-0.258442	1.282242	-1.153640
N	-0.446177	2.996640	1.423548
Si	-2.337152	0.002842	-3.299123
C	-1.248086	0.566464	-1.886184
N	-2.587110	-1.273951	-1.992030
C	-1.006098	-3.268092	0.015742
H	-0.636288	-2.829586	-2.063656
H	0.359542	-4.136446	-1.394080
H	0.697026	-2.438609	-0.991274
H	-1.051273	-5.411507	0.016195
C	-2.168003	-2.181677	1.871915
H	-2.109470	-0.050954	2.290013
H	-2.291922	-1.093456	3.706608
H	-3.665617	-0.829747	2.625369
H	-3.075401	-3.514702	3.285100
N	2.402564	-0.789470	3.361334
N	3.662009	-0.433043	1.611600
O	0.418240	2.363040	-1.616509
C	-1.069406	4.191430	1.011161
Si	-4.411385	0.915673	-4.054077
H	-3.552821	2.521457	-5.789343
H	-5.166887	1.916214	-6.187869
H	-3.748974	0.906271	-6.491334

H	-6.075991	2.677638	-3.620452
H	-4.552403	3.109911	-2.832905
H	-5.633867	1.922619	-2.084675
Si	-1.190904	-0.871786	-5.197133
H	-1.329441	1.210135	-6.620466
H	-0.030929	0.127919	-7.142123
H	0.196141	1.160536	-5.724797
H	0.870296	-1.410326	-3.879795
H	0.953959	-2.076515	-5.523044
H	-0.006424	-2.893754	-4.281842
H	-3.167008	-1.342110	-6.683540
H	-2.702968	-2.802821	-5.803548
H	-1.739387	-2.257550	-7.182913
C	-3.691646	-2.047260	-1.543106
C	-0.099069	-3.167717	-1.174552
C	-1.386792	-4.508211	0.520671
H	-2.455482	-5.572088	2.055798
C	-2.577753	-0.967016	2.657621
C	-2.511902	-3.446692	2.357152
C	1.307628	-1.125991	4.217458
C	3.736530	-0.790784	3.800906
H	3.495186	-0.382386	5.889928
H	5.146265	-0.799691	5.389866
H	3.905696	-2.056948	5.528540
C	4.517271	-0.568518	2.717665
H	6.423662	-1.257733	1.998016
H	6.458552	-0.448983	3.572679
H	6.265333	0.495136	2.083055
C	4.126568	-0.185028	0.284235
Si	0.263629	3.410612	-2.910319
H	-1.510294	2.376988	-4.356674
H	-1.515373	4.141750	-4.447688
H	-2.210520	3.334535	-3.030741
H	2.622773	3.374236	-3.690557
H	1.520443	3.434648	-5.073635
H	1.778043	1.910071	-4.197458
H	-0.380682	5.419529	-1.602450
H	0.483577	5.860395	-3.087785
H	1.380363	5.268618	-1.680096
C	-2.319608	4.230086	0.349184
H	-2.645458	2.363882	-0.709091
H	-4.122932	3.243926	-0.243884
H	-3.166242	2.354287	0.961493
H	-3.821620	5.504728	-0.489832
C	-0.419157	5.389891	1.369144
H	1.266266	4.382178	2.264820
H	0.681097	5.787887	3.169663
H	1.611590	6.035451	1.693091
H	-0.465767	7.530443	1.246192
C	-4.184706	1.627129	-5.793264
C	-5.235315	2.279718	-3.037816
C	-0.534200	0.548905	-6.262486
C	0.292255	-1.912348	-4.663452
C	-2.312173	-1.916056	-6.311375
C	-4.611700	-1.494231	-0.628217
H	-3.594639	0.069830	0.467841
H	-5.354664	0.256441	0.371528
H	-4.318100	0.589557	-1.030884
H	-6.393697	-1.836170	0.510054
C	-3.898869	-3.336502	-2.059522
H	-2.373990	-3.220315	-3.587260
H	-3.507997	-4.575812	-3.769946
H	-2.247495	-4.626356	-2.531279
H	-5.160224	-5.069042	-2.019026
C	-2.159945	-4.599295	1.672529
C	1.141750	-2.474206	4.571211
H	2.897388	-3.128187	3.546978
C	0.496994	-0.100833	4.735298
H	-1.135278	0.289660	6.079740
H	0.848659	1.361151	3.232781
C	4.089263	-1.018997	5.225298
C	5.991026	-0.439138	2.585868
C	4.686108	-1.256742	-0.429488
H	5.621959	-1.805477	-2.288710
H	4.698780	-2.594036	1.232422
C	4.066323	1.122552	-0.231103
H	4.555496	2.313436	-1.954713
H	2.477092	1.943452	0.920894
C	-1.399387	3.290822	-3.765003
C	1.672165	2.994307	-4.081266
C	0.456742	5.147772	-2.254087
C	-3.105673	2.984948	0.066486
C	-2.856069	5.470809	0.010461
H	-2.619709	7.610590	0.019710
C	0.860866	5.389895	2.162884
C	-0.983043	6.608373	0.990146
C	-4.466437	-0.074405	-0.174160
C	-5.688063	-2.265033	-0.197583
H	-6.734846	-4.139498	-0.349094
C	-2.956977	-3.966542	-3.046373
C	-5.003297	-4.069347	-1.620354
C	1.973768	-3.572629	3.936202
H	2.842850	-4.271500	5.824358
H	3.083657	-5.359391	4.442671
H	1.509952	-5.275276	5.229908
H	0.277627	-4.632694	3.087310
H	1.806361	-4.913473	2.226395
H	0.926012	-3.378294	2.032953
C	-0.487056	-0.468697	5.654734
C	0.683117	1.350257	4.318350
H	-1.453867	1.778498	4.157525
H	-0.408480	3.195973	4.130086
H	-0.709045	2.364233	5.665988
H	1.818484	1.944294	6.082355
H	1.991219	3.037994	4.695384
H	2.842627	1.490747	4.701075
C	5.186716	-0.999399	-1.706572
H	5.531095	0.466881	-3.234973
C	4.700758	-2.665246	0.138369
H	3.330688	-3.455058	-1.356410
H	2.535644	-2.881399	0.120762
H	3.416741	-4.423501	0.132387
H	5.978557	-4.402214	0.300861
H	6.865311	-2.912601	-0.081917
H	5.917697	-3.740049	-1.329985
C	4.582750	1.324693	-1.512408
C	3.465931	2.273976	0.566472
H	4.357037	1.860234	2.542832
H	3.866074	3.533434	2.281239
H	5.328877	2.917724	1.493471
H	4.194375	4.001790	-0.551005
H	2.662793	4.253157	0.297867
H	2.681381	3.301918	-1.184327
C	-2.189161	6.655473	0.306553
C	-5.885841	-3.551494	-0.685191
C	2.373580	-4.676716	4.921294
C	1.203916	-4.159495	2.746191
C	-0.665408	-1.795669	6.026784
C	-0.548597	2.215269	4.591287
C	1.911841	1.985513	4.990357
C	5.135608	0.279008	-2.240523
C	3.415946	-3.401853	-0.264489
C	5.940068	-3.468153	-0.269211
C	4.308062	2.655598	1.796503
C	3.243359	3.527766	-0.277721

Calculated energies and coordinates of **INT19**

Electronic energy ... -4422.26083778 Eh
Total Enthalpy ... -4420.72083229 Eh
Final Gibbs free energy ... -4420.92372455 Eh

CARTESIAN COORDINATES (ANGSTROEM)

C	3.962274	5.518463	24.819505
H	4.183650	4.832165	23.994022
H	2.935268	5.880967	24.686457
H	4.005059	4.937869	25.745172
C	9.508118	4.353156	34.573254
H	7.952503	5.474627	35.534824

H	9.254088	3.429190	35.083945
Si	10.120407	5.862325	28.759361
N	9.229695	4.759640	27.759802
N	10.852854	5.195569	30.060483
C	9.824222	7.582398	28.295828
C	8.525200	5.323725	26.706018
C	9.428365	3.329646	27.801791
C	11.929873	5.386616	30.769794
C	9.110733	7.734991	27.076997
N	10.359955	8.532580	29.091617
Si	7.422192	6.221229	24.912161
C	8.507049	6.668933	26.373629
N	7.764474	4.609615	25.751797
C	10.274614	2.774684	26.827951
H	10.388101	4.310465	25.311767
H	11.650675	3.064991	25.205707
H	11.725009	4.312522	26.459326
H	11.052102	0.947084	26.022945
C	8.818275	2.540176	28.786683
H	6.950635	3.278020	29.549537
H	8.371807	4.058202	30.272053
H	7.932170	2.408462	30.726464
H	8.518642	0.527504	29.457201
N	11.983869	5.600691	32.124064
N	13.243443	5.315168	30.360567
O	8.834375	9.002225	26.673341
C	9.755288	9.738639	29.344249
Si	5.152920	6.984054	24.811589
H	5.435905	8.836148	23.121134
H	3.800981	8.169140	23.124551
H	5.100525	7.297734	22.303853
H	3.831359	8.740511	25.955488
H	5.560596	8.963574	26.277490
H	4.647051	7.747374	27.172649
Si	8.323295	6.079232	22.715265
H	7.368770	8.186698	21.721640
H	8.715585	7.546595	20.769980
H	9.034584	8.449029	22.258372
H	10.755086	5.894082	23.349219
H	10.468805	5.424466	21.666088
H	10.073631	4.302768	22.976867
H	6.176760	5.327890	21.639457
H	7.080633	3.944823	22.269166
H	7.587585	4.721419	20.760860
C	6.876246	3.563730	26.144228
C	11.045411	3.659521	25.894998
C	10.413016	1.389202	26.783524
H	9.875612	-0.498741	27.661717
C	7.976190	3.112258	29.891616
C	8.998553	1.156774	28.712004
C	10.857129	5.582698	33.015628
C	13.319421	5.617834	32.559750
H	13.124099	6.589577	34.459277
H	14.742215	5.955357	34.099434
H	13.433196	4.855965	34.560942
C	14.100954	5.445724	31.466607
H	15.896418	4.288784	31.250711
H	16.066051	5.765110	32.211568
H	15.940292	5.855683	30.445191
C	13.681595	4.900456	29.059275
Si	9.355866	10.029064	25.470855
H	7.443609	9.431819	24.010398
H	8.074861	11.025028	23.565003
H	7.059312	10.845089	25.008029
H	11.662137	9.231637	24.970460
H	10.824412	9.717740	23.490392
H	10.457026	8.156798	24.249635
H	9.213882	11.973038	26.988276
H	10.140529	12.379943	25.523642
H	10.879905	11.431779	26.833415
C	8.343833	9.930752	29.470465
H	7.183776	8.379559	28.445537
H	6.428562	9.071937	29.890478
H	7.782298	7.933579	30.036424
H	6.769399	11.323925	29.854392
C	10.602624	10.839993	29.665239
H	12.374495	9.720106	30.150033
H	12.596345	11.493608	30.136417
H	12.477275	10.579392	28.624066
H	10.735120	12.906049	30.199258
C	4.864555	7.904760	23.183061
C	4.765135	8.215003	26.191376
C	8.358748	7.726926	21.792027
C	10.068919	5.357544	22.685715
C	7.180808	4.909101	21.762361
C	5.953913	3.757825	27.196536
H	6.690104	5.266755	28.583027
H	4.927951	5.111594	28.509017
H	5.829741	5.912626	27.212661
H	4.393370	2.862280	28.360836
C	6.927634	2.332874	25.466315
H	8.627507	2.878995	24.262446
H	7.369007	1.985692	23.386226
H	8.426489	1.132984	24.512188
H	6.112596	0.353759	25.340742
C	9.762663	0.580836	27.706877
C	10.568445	4.372151	33.665681
H	12.259902	3.339601	32.864257
C	10.127021	6.763850	33.246786
H	8.485520	7.567894	34.374436
H	10.550867	7.864881	31.463239
C	13.672085	5.764077	33.994364
C	15.576419	5.334016	31.335466
C	14.010584	3.548532	28.865954
H	14.765679	2.132189	27.436427
H	13.562901	2.989654	30.877977
C	13.821411	5.861254	28.044368
H	14.459392	6.150690	26.013464
H	12.635135	7.389226	28.975993
C	7.845963	10.368448	24.412241
C	10.699758	9.206134	24.451843
C	9.955224	11.600028	26.271828
C	7.384827	8.771364	29.449648
C	7.847661	11.198515	29.766188
H	8.270506	13.262082	30.237187
C	12.091039	10.652761	29.649472
C	10.060797	12.080769	29.976630
C	5.844697	5.074983	27.912965
C	5.110615	2.709015	27.557225
H	4.497948	0.678556	27.190981
C	7.894849	2.075200	24.344554
C	6.062603	1.308925	25.858884
C	11.320516	3.087217	33.369939
H	12.179899	2.934392	35.379700
H	12.350483	1.473596	34.384949
H	10.793514	1.868898	35.111176
H	9.523938	1.977466	32.857689
H	11.015115	1.274830	32.200752
H	10.319755	2.734910	31.464751
C	9.079577	6.685834	34.168345
C	10.443529	8.072817	32.539159
H	8.360114	8.730961	32.365447
H	9.562503	9.981050	32.059014
H	9.246249	9.467575	33.722435
H	11.754103	8.819284	34.117168
H	11.888539	9.679963	32.573070
H	12.637925	8.092707	32.752270
C	14.510915	3.172223	27.617915
H	15.079248	3.796956	25.644903
C	13.793129	2.489803	29.929673
H	12.770223	1.109575	28.596211
H	11.682732	2.222548	29.430310
H	12.403681	0.857801	30.311027
H	14.879478	0.961363	31.018135
H	15.933563	2.212612	30.329501
H	15.228453	0.969561	29.289017
C	14.335942	5.433814	26.818134
C	13.468089	7.318831	28.262919
H	14.881830	7.741505	29.883868
H	14.437885	9.149179	28.915258
H	15.557869	7.941211	28.250945
H	13.826842	8.223358	26.298227

H	12.501156	8.938719	27.239055
H	12.261552	7.383388	26.448337
C	8.683423	12.286350	30.000062
C	5.162352	1.486149	26.897666
C	11.680698	2.302430	34.637859
C	10.496340	2.216133	32.411144
C	8.774236	5.500683	34.824396
C	9.332960	9.116327	32.686413
C	11.765564	8.691590	33.027537
C	14.683165	4.106326	26.608292
C	12.588298	1.620715	29.547342
C	15.034179	1.614393	30.152591
C	14.660888	8.079295	28.866765
C	12.995355	7.999882	26.977740

Calculated energies and coordinates of **INT20**

Electronic energy ... -4535.58069130 Eh
Total Enthalpy ... -4534.03124090 Eh
Final Gibbs free energy ... -4534.23629164 Eh

CARTESIAN COORDINATES (ANGSTROEM)

C	4.100201	5.464661	24.616070
H	4.332456	4.933109	23.685662
H	3.049633	5.777395	24.562721
H	4.205808	4.746238	25.432546
C	9.470069	4.270018	34.350546
H	7.995966	5.439482	35.380723
H	9.114568	3.331069	34.764913
Si	9.700198	5.986828	29.272345
N	9.038962	4.889712	28.030134
N	11.052440	5.262771	29.889552
C	9.565900	7.668237	28.539663
C	8.460185	5.431364	26.920212
C	9.244608	3.465063	28.078950
C	12.056648	5.355158	30.693594
C	9.078449	7.833551	27.222522
N	9.928848	8.603463	29.430428
C	8.578500	6.479875	30.809275
Si	7.474785	6.277605	25.007159
C	8.530674	6.766617	26.488367
N	7.689385	4.731733	25.970311
C	10.172932	2.915013	27.180134
H	10.426570	4.441349	25.661667
H	11.703696	3.207664	25.702804
H	11.626821	4.466917	26.949483
H	11.028350	1.092637	26.448998
C	8.555741	2.668185	29.003333
H	7.130759	4.146785	29.667615
H	8.198864	3.560735	30.931858
H	6.889434	2.528052	30.346068
H	8.203261	0.654583	29.646771
N	12.102951	5.509029	32.069512
N	13.386536	5.242360	30.320650
O	9.072137	9.097658	26.737788
C	9.459670	9.913939	29.413050
O	7.532008	6.330678	31.302767
Si	5.203052	6.995425	24.760924
H	5.483456	8.882603	23.095043
H	3.878733	8.146600	23.034297
H	5.241293	7.335978	22.259241
H	3.580958	8.393023	26.019031
H	5.190715	9.130251	26.019375
H	4.832614	7.814538	27.135457
Si	8.398501	5.960514	22.835304
H	7.523789	8.041300	21.717496
H	8.794775	7.253521	20.771463
H	9.221025	8.245588	22.173528
H	10.828812	5.752019	23.462329
H	10.507422	5.144417	21.830868
H	10.099515	4.156334	23.241312
H	6.275909	5.299750	21.674021
H	6.913825	3.936441	22.600297
H	7.654613	4.380714	21.054729
C	6.822416	3.654509	26.316284
C	11.023495	3.801260	26.319437
C	10.324267	1.530473	27.152778
H	9.731757	-0.362525	27.984775
C	7.641683	3.258768	30.035879
C	8.750603	1.286607	28.951924
C	10.970407	5.503582	32.945537
C	13.429613	5.443316	32.528682
H	13.167660	6.192102	34.523761
H	14.820018	5.689493	34.119486
H	13.570262	4.477824	34.437770
C	14.225528	5.294640	31.442878
H	16.030826	4.168102	31.111193
H	16.173026	5.503456	32.263974
H	16.083886	5.827877	30.522372
C	13.829525	4.931820	28.997549
Si	9.447493	9.930563	25.349092
H	7.513639	9.078328	24.050628
H	8.048336	10.630375	23.397582
H	7.099070	10.579351	24.897198
H	11.759304	9.147157	24.888494
H	10.897186	9.491319	23.382825
H	10.574840	7.987989	24.268892
H	9.258419	12.030560	26.630732
H	10.163474	12.304094	25.122289
H	10.940614	11.520654	26.514970
C	8.087767	10.265426	29.276899
H	6.956594	8.833028	28.103253
H	6.034981	9.662691	29.366459
H	7.180533	8.379668	29.787919
H	6.664641	11.861082	29.244383
C	10.397867	10.942191	29.689970
H	11.977697	9.624434	30.327323
H	12.333684	11.366325	30.525286
H	12.388035	10.612903	28.930200
H	10.722636	13.043338	29.943265
C	4.950162	7.928262	23.132041
C	4.652602	8.181264	26.122738
C	8.488836	7.531123	21.788261
C	10.118572	5.179981	22.856410
C	7.195529	4.782270	21.966578
C	5.822178	3.821777	27.298364
H	6.360587	5.848182	27.938876
H	5.216212	5.004544	28.992860
H	4.682535	5.630038	27.443627
H	4.260309	2.857344	28.403064
C	6.982454	2.411448	25.673786
H	8.767573	2.988623	24.619516
H	7.594055	2.102156	23.629214
H	8.563121	1.240152	24.826244
H	6.288751	0.388241	25.547651
C	9.608326	0.716312	28.021466
C	10.561036	4.271359	33.478413
H	12.199342	3.191310	32.634628
C	10.350705	6.722781	33.281573
H	8.768116	7.577666	34.456295
H	10.986878	7.887441	31.622624
C	13.762422	5.454880	33.977064
C	15.703007	5.191666	31.328658
C	14.252459	3.621051	28.723514
H	15.060395	2.342175	27.195025
H	14.017338	2.948670	30.737151
C	13.846865	5.945855	28.028786
H	14.357925	6.381704	25.986699
H	12.608778	7.301534	29.114242
C	7.884664	10.071741	24.326298
C	10.793950	9.055173	24.383189
C	10.005103	11.604217	25.950658
C	7.010329	9.230263	29.122682
C	7.717976	11.606727	29.346205
H	8.335871	13.653963	29.616595
C	11.851504	10.617637	29.887841
C	9.982738	12.269742	29.747086
C	5.523985	5.145779	27.952129
C	5.023788	2.729597	27.638442
H	4.551821	0.659882	27.294658
C	8.037613	2.179325	24.629251

C	6.150759	1.351203	26.034123
C	11.246868	2.962120	33.127768
H	12.086650	2.707539	35.136196
H	12.191344	1.269041	34.099149
H	10.650695	1.721892	34.829651
H	9.407158	1.949094	32.552234
H	10.878829	1.196882	31.908069
H	10.272760	2.699687	31.196351
C	9.283571	6.665294	34.179779
C	10.812652	8.048273	32.694871
H	8.794749	8.827623	32.383811
H	10.080268	10.020344	32.226135
H	9.605060	9.470756	33.837286
H	12.030001	8.606469	34.421010
H	12.348898	9.550774	32.956290
H	12.973787	7.906293	33.083765
C	14.729682	3.346941	27.440491
H	15.150418	4.107180	25.477592
C	14.122731	2.503208	29.741373
H	12.900059	1.230088	28.473322
H	11.961240	2.354710	29.457485
H	12.700792	0.921191	30.208914
H	15.262381	0.884763	30.621236
H	16.279524	2.132000	29.871368
H	15.408726	0.964016	28.866984
C	14.331864	5.621545	26.760632
C	13.393943	7.356082	28.352104
H	14.917023	7.736789	29.874910
H	14.258133	9.197447	29.127412
H	15.405303	8.183479	28.226528
H	13.545376	8.366370	26.416117
H	12.249198	8.959889	27.470695
H	12.053420	7.418237	26.638663
C	8.650102	12.615679	29.563172
C	5.181309	1.499684	27.014775
C	11.562176	2.122640	34.373592
C	10.400091	2.155466	32.135369
C	8.842004	5.455517	34.699251
C	9.754731	9.148122	32.799481
C	12.122616	8.546146	33.329728
C	14.775894	4.338256	26.471090
C	12.842541	1.706601	29.457895
C	15.342118	1.574160	29.774036
C	14.565169	8.165586	28.930993
C	12.781553	8.062505	27.142199

Calculated energies and coordinates of **TS17**

Electronic energy ... -4535.57692796 Eh
Total Enthalpy ... -4534.02705797 Eh
Final Gibbs free energy ... -4534.23119970 Eh

CARTESIAN COORDINATES (ANGSTROEM)

C	-5.563406	-0.829493	-4.148595
H	-5.108759	-1.639589	-4.729912
H	-6.533457	-0.595680	-4.605874
H	-5.741382	-1.212041	-3.139146
C	-0.134323	-1.720900	6.050917
H	-1.431148	-0.512115	7.258440
H	-0.503801	-2.654032	6.465666
Si	-0.243004	0.085121	0.923020
N	-1.051850	-1.026529	-0.193930
N	1.093108	-0.688133	1.510004
C	-0.440552	1.726131	0.139991
C	-1.558107	-0.514593	-1.368637
C	-1.030370	-2.454768	-0.014606
C	2.174246	-0.628277	2.210002
C	-0.918078	1.890899	-1.154341
N	-0.171163	2.623703	1.140657
C	-1.084620	1.067644	2.376964
Si	-2.279399	0.164420	-3.437087
C	-1.442743	0.783069	-1.871428
N	-2.258620	-1.292961	-2.322606
C	-0.211433	-3.218489	-0.860918
H	0.170747	-2.046229	-2.626283
H	1.391144	-3.289997	-2.288964
H	1.334755	-1.798945	-1.328282
H	0.347854	-5.210068	-1.422490
C	-1.796890	-3.053345	0.996729
H	-3.101545	-1.419359	1.570078
H	-1.853072	-1.805846	2.753006
H	-3.246469	-2.875009	2.565987
H	-2.423729	-4.922643	1.834557
N	2.346126	-0.542355	3.579481
N	3.463040	-0.730064	1.713211
O	-1.041730	3.163859	-1.621497
C	-0.750726	3.901193	1.213648
O	-1.977301	1.258726	3.100846
Si	-4.493368	0.727371	-4.161907
H	-3.894501	2.248894	-6.089933
H	-5.474017	1.479971	-6.302236
H	-3.998907	0.557399	-6.616979
H	-6.076638	1.656393	-2.455107
H	-5.781206	2.823262	-3.754562
H	-4.583910	2.603802	-2.471179
Si	-0.712395	-0.097232	-5.225234
H	-1.724531	1.454270	-6.931969
H	0.006330	1.201207	-7.194999
H	-0.541321	2.323483	-5.944410
H	1.140571	0.208728	-3.536978
H	1.751416	0.292133	-5.200852
H	1.373442	-1.271657	-4.462815
H	-2.115691	-1.659635	-6.615509
H	-0.901721	-2.555897	-5.687168
H	-0.424264	-1.654594	-7.135248
C	-3.317202	-2.154896	-1.909818
C	0.721499	-2.556764	-1.830515
C	-0.265301	-4.607746	-0.756447
H	-1.117341	-6.306114	0.250194
C	-2.543427	-2.242967	2.017072
C	-1.814159	-4.446214	1.071077
C	1.294908	-0.517380	4.550335
C	3.705010	-0.657070	3.915847
H	3.685745	0.047686	5.943485
H	5.240462	-0.602342	5.385991
H	3.902829	-1.693878	5.778679
C	4.399881	-0.752765	2.756485
H	6.164273	-1.858005	2.192970
H	6.415411	-0.595172	3.408169
H	6.168050	-0.164522	1.706147
C	3.771402	-0.941829	0.334374
Si	-0.794704	4.107849	-2.960907
H	-2.361225	2.860470	-4.444185
H	-2.088679	4.494009	-5.058575
H	-3.171880	4.234713	-3.679042
H	1.672229	3.995452	-3.220922
H	0.874482	4.204139	-4.787445
H	0.914281	2.610073	-4.018061
H	-1.656456	6.047002	-1.695641
H	-0.667282	6.593725	-3.069214
H	0.098430	5.964593	-1.592939
C	-2.137327	4.170827	1.061122
H	-2.942404	2.148967	1.100067
H	-3.198469	3.005921	-0.408668
H	-4.148719	3.434196	1.023138
H	-3.662118	5.670215	1.138084
C	0.119558	4.968639	1.552017
H	1.836719	3.718825	1.938704
H	2.034480	5.429125	2.423834
H	2.106323	4.967216	0.724381
H	0.300361	7.061319	1.975731
C	-4.444879	1.312350	-5.960120
C	-5.304395	2.079214	-3.106142
C	-0.758889	1.366455	-6.425588
C	1.050610	-0.228227	-4.536664
C	-1.080793	-1.639139	-6.256238
C	-4.274918	-1.731796	-0.963011
H	-3.469594	-0.002870	0.109485
H	-5.232458	-0.156626	0.135019
H	-4.385501	0.389573	-1.318968
H	-5.974392	-2.318712	0.202000

C	-3.401806	-3.438407	-2.476204
H	-1.436165	-3.555656	-3.345649
H	-2.740911	-3.397329	-4.519697
H	-2.488463	-4.946279	-3.701954
H	-4.446057	-5.309526	-2.459437
C	-1.075694	-5.222395	0.188351
C	0.863239	-1.745116	5.074556
H	2.347722	-2.880342	4.039904
C	0.780589	0.720773	4.981231
H	-0.640035	1.611991	6.324841
H	1.239023	1.918101	3.286092
C	4.156626	-0.725709	5.329248
C	5.860713	-0.850848	2.502755
C	4.160081	-2.226931	-0.074358
H	4.773125	-3.392996	-1.774748
H	4.231591	-3.032046	1.904103
C	3.671485	0.139240	-0.556008
H	3.908241	0.705453	-2.617381
H	2.468677	1.402060	0.668150
C	-2.236191	3.906926	-4.144330
C	0.814566	3.682963	-3.824289
C	-0.749770	5.842112	-2.275938
C	-3.155399	3.128997	0.679822
C	-2.597036	5.475322	1.247901
H	-2.126940	7.522399	1.718872
C	1.604166	4.752845	1.677152
C	-0.385332	6.255157	1.722379
C	-4.339646	-0.309238	-0.478036
C	-5.241561	-2.640879	-0.534315
H	-6.057611	-4.624536	-0.704623
C	-2.461625	-3.861678	-3.565241
C	-4.395174	-4.310767	-2.032812
C	1.414413	-3.072026	4.584077
H	2.367228	-3.561670	6.495637
H	2.267421	-4.911641	5.345869
H	0.829461	-4.395251	6.226007
H	-0.538746	-3.888321	4.073835
H	0.811779	-4.675835	3.234756
H	0.273256	-3.067045	2.728863
C	-0.201960	0.685989	5.971934
C	1.241433	2.043373	4.379084
H	-0.735574	2.995060	4.422463
H	0.611185	4.089732	4.130790
H	0.338205	3.472493	5.760681
H	2.747878	2.476864	5.897395
H	2.899425	3.430642	4.411931
H	3.425122	1.747849	4.420614
C	4.472429	-2.410946	-1.422731
H	4.622609	-1.521863	-3.370260
C	4.152466	-3.408560	0.878515
H	2.677960	-4.555285	-0.234063
H	1.969184	-3.463935	0.960834
H	2.757334	-4.966457	1.490082
H	5.347845	-5.117805	1.456098
H	6.282438	-3.855421	0.627679
H	5.210964	-4.926035	-0.289697
C	3.983319	-0.100053	-1.895067
C	3.267517	1.520520	-0.073131
H	4.769522	1.688990	1.513491
H	4.152684	3.236630	0.921197
H	5.300035	2.308000	-0.066506
H	3.482324	2.732985	-1.880393
H	2.222585	3.276078	-0.755850
H	1.934612	1.859259	-1.757803
C	-1.740075	6.517847	1.573501
C	-5.299348	-3.928775	-1.051669
C	1.738622	-4.034393	5.733489
C	0.432457	-3.713628	3.595531
C	-0.656006	-0.517121	6.497243
C	0.300047	3.208346	4.696670
C	2.667538	2.434675	4.804516
C	4.384722	-1.359413	-2.322592
C	2.807883	-4.140294	0.771458
C	5.319051	-4.376075	0.651096
C	4.446333	2.226245	0.616741
C	2.700933	2.393887	-1.189472

Calculated energies and coordinates of **INT21**

Electronic energy ... -4535.60936616 Eh
Total Enthalpy ... -4534.05700954 Eh
Final Gibbs free energy ... -4534.26254715 Eh

CARTESIAN COORDINATES (ANGSTROEM)

C	4.717564	5.027764	23.734533
H	5.268087	4.328494	23.094440
H	3.761222	5.247549	23.243270
H	4.510503	4.511756	24.677199
C	10.055175	4.530622	34.498910
H	8.970163	5.828439	35.817821
H	9.672051	3.625810	34.961650
Si	9.688504	6.112895	29.303856
N	8.780662	4.994090	28.247947
N	11.082634	5.346257	29.771955
C	9.456426	7.725858	28.444832
C	8.413705	5.435304	26.993410
C	8.691338	3.596561	28.579255
C	12.174900	5.378595	30.454989
C	8.806285	7.875077	27.256715
N	9.045467	8.399415	29.681165
C	8.824864	7.340371	30.585840
Si	7.836942	6.076589	24.850865
C	8.536764	6.725645	26.452265
N	7.849709	4.629948	25.991989
C	9.529090	2.685434	27.915047
H	10.148715	3.663066	26.096663
H	11.196438	2.329624	26.608934
H	11.243677	3.887761	27.457613
H	10.012969	0.610115	27.678401
C	7.817186	3.180093	29.594415
H	6.685261	5.010687	29.886649
H	7.732660	4.582831	31.227652
H	6.240088	3.674806	30.964898
H	7.003475	1.474082	30.605402
N	12.392531	5.530428	31.815454
N	13.447100	5.186878	29.929743
O	8.134941	9.039684	26.981178
C	8.820549	9.784234	29.885957
O	8.342691	7.418479	31.700131
Si	5.689003	6.631873	23.971228
H	6.429716	8.346613	22.272310
H	4.892959	7.588336	21.832848
H	6.409224	6.722882	21.562098
H	3.817187	7.398718	25.464976
H	4.397529	8.714870	24.440926
H	5.318258	8.244910	25.880204
Si	9.438957	5.762554	23.117353
H	9.039504	7.940736	21.909043
H	10.651235	7.287374	21.583267
H	10.328111	8.113131	23.115405
H	11.318600	5.440755	24.754194
H	11.843738	5.155117	23.084538
H	10.927784	3.915661	23.953805
H	7.951179	5.110607	21.192157
H	8.487997	3.674710	22.072645
H	9.599234	4.509100	20.978201
C	6.860985	3.628859	26.220164
C	10.585122	3.163878	26.963864
C	9.381102	1.327676	28.196576
H	8.340147	-0.174419	29.330262
C	7.073786	4.163325	30.453142
C	7.698850	1.811719	29.841066
C	11.397365	5.627117	32.843519
C	13.756216	5.372429	32.117631
H	13.859564	6.205998	34.095728
H	15.341301	5.405785	33.532682
H	13.940266	4.447471	34.037822
C	14.411863	5.180445	30.948877
H	16.113458	3.984881	30.375610
H	16.447681	5.268962	31.547142
H	16.177594	5.661558	29.839505

C	13.706105	4.775575	28.584697
Si	8.426531	10.324805	25.958715
H	8.154129	8.978116	23.890250
H	7.989299	10.699965	23.531145
H	6.649602	9.824924	24.294854
H	10.690045	10.985317	26.760359
H	10.437243	11.452478	25.069541
H	10.799830	9.767705	25.474809
H	6.447808	11.500201	26.857852
H	7.546220	12.640182	26.059970
H	7.921247	12.025734	27.685537
C	7.540085	10.239068	30.243761
H	6.383309	8.640719	29.404022
H	5.428270	9.874093	30.256839
H	6.379326	8.674488	31.167040
H	6.390224	11.964065	30.790546
C	9.898709	10.675509	29.738851
H	11.717688	9.613202	30.190809
H	11.929549	11.002725	29.112377
H	11.208831	9.479126	28.518683
H	10.512025	12.725154	29.879066
C	5.889273	7.395743	22.250876
C	4.718560	7.860718	25.048089
C	9.897949	7.438329	22.366713
C	11.025483	4.994771	23.798318
C	8.800721	4.661077	21.715377
C	5.723463	3.904726	27.008717
H	6.293698	5.808618	27.931159
H	4.654832	5.243595	28.303525
H	5.040017	5.912518	26.718957
H	3.923469	3.098464	27.844174
C	6.997766	2.380360	25.589995
H	8.633254	2.952708	24.298021
H	7.882972	1.388850	23.903315
H	8.953300	1.543550	25.303836
H	6.135077	0.437298	25.316649
C	8.455088	0.887819	29.133748
C	10.943822	4.434323	33.426732
H	12.216672	3.192737	32.242692
C	10.994731	6.895723	33.299462
H	9.768924	7.888797	34.756797
H	11.326547	7.996542	31.530902
C	14.249971	5.362691	33.518348
C	15.860435	5.012070	30.662957
C	14.027549	3.427526	28.353475
H	14.542694	2.002673	26.826857
H	14.092528	2.894807	30.423656
C	13.639541	5.723852	27.549132
H	13.853406	5.974980	25.423922
H	12.460566	7.188909	28.533739
C	7.739245	9.921482	24.261679
C	10.260580	10.661265	25.807605
C	7.502569	11.758320	26.709583
C	6.363575	9.307781	30.273994
C	7.375800	11.599321	30.511647
H	8.283059	13.547099	30.594015
C	11.262617	10.172871	29.364631
C	9.683040	12.029573	29.987495
C	5.425807	5.283675	27.528961
C	4.790931	2.890733	27.221962
H	4.194243	0.867955	26.796753
C	8.178674	2.055205	24.719661
C	6.022354	1.404652	25.800736
C	11.349136	3.068789	32.902957
H	12.517732	2.539536	34.679484
H	12.168120	1.183633	33.587484
H	10.903349	1.815581	34.641538
H	9.294698	2.388656	32.664982
H	10.466768	1.483933	31.688292
H	9.985423	3.117040	31.205091
C	10.122548	6.934235	34.386494
C	11.443152	8.178092	32.609829
H	9.516745	9.193046	32.825087
H	10.858038	10.241729	32.348064
H	10.749486	9.689282	34.017684
H	13.109075	8.621897	33.941834
H	13.144260	9.503623	32.409126
H	13.611767	7.802298	32.444014
C	14.298302	3.038940	27.039896
H	14.435349	3.633544	24.982343
C	13.998005	2.385783	29.458487
H	12.490745	1.129024	28.520939
H	11.814982	2.381009	29.570288
H	12.587804	0.949019	30.283744
H	15.167019	0.749633	30.261220
H	16.115832	1.858657	29.250395
H	15.010290	0.693506	28.507335
C	13.906842	5.279902	26.253780
C	13.306778	7.178776	27.834455
H	14.739977	7.459785	29.471879
H	14.231025	8.953134	28.676757
H	15.375516	7.867525	27.862499
H	13.670916	8.043289	25.856489
H	12.571999	8.963009	26.878545
H	11.997490	7.477032	26.114195
C	8.434578	12.490571	30.392238
C	4.935166	1.643796	26.627449
C	11.759182	2.102501	34.021555
C	10.207597	2.479228	32.064584
C	9.659988	5.768055	34.980718
C	10.580945	9.389480	32.976206
C	12.918284	8.533169	32.865764
C	14.235640	3.953879	26.001155
C	12.640003	1.671781	29.461042
C	15.141888	1.369604	29.358861
C	14.487104	7.901666	28.504219
C	12.863141	7.949413	26.591729

Calculated energies and coordinates of **TS18**

Electronic energy ... -4938.59205778 Eh
Total Enthalpy ... -4936.87518860 Eh
Final Gibbs free energy ... -4937.09659925 Eh

CARTESIAN COORDINATES (ANGSTROEM)

C	-5.138829	-1.464985	-4.712115
H	-4.561716	-1.983364	-5.485909
H	-6.170826	-1.370877	-5.073439
H	-5.140520	-2.108126	-3.827286
C	0.407448	-0.955196	6.055747
H	-0.270637	0.507040	7.471253
H	-0.071980	-1.781261	6.573489
Si	-0.018348	-0.052571	0.667294
N	-0.658496	-1.206149	-0.621378
N	1.459598	-0.715800	1.108402
C	-0.314030	1.559505	-0.190285
C	-2.239017	-0.703799	1.981722
C	-1.266106	-0.666856	-1.744115
C	-0.106417	-2.535415	-0.834637
C	2.508188	-0.672525	1.860935
C	-1.129698	1.769499	-1.256465
N	-0.369777	2.347656	1.019953
C	-0.503126	1.393504	2.034825
N	-3.150076	-0.092156	2.398266
Si	-2.128315	0.117716	-3.748040
C	-1.489750	0.672363	-2.088897
N	-1.670966	-1.420464	-2.870598
C	0.970720	-2.614608	-1.740732
H	0.854902	-0.871403	-2.998404
H	2.465818	-1.607762	-2.896100
H	1.817740	-0.658585	-1.542232
H	2.274617	-3.917037	-2.829376
C	-0.639038	-3.700958	-0.262628
H	-1.942222	-2.742578	1.176928
H	-1.517798	-4.417331	1.550566
H	-2.679096	-4.077261	0.267927
H	-0.571580	-5.840470	-0.266561
N	2.698203	-0.378974	3.215986
N	3.798807	-0.985495	1.425206
O	-1.770206	2.976218	-1.403764
C	-0.217405	3.752930	1.163226

O	-0.748438	1.615805	3.204395
C	-4.073572	0.760624	2.958029
Si	-4.443080	0.253604	-4.352743
H	-4.367698	2.319546	-5.796591
H	-5.676080	1.226030	-6.263536
H	-4.017150	0.870899	-6.759753
H	-5.694549	0.450811	-2.180050
H	-6.439654	1.430951	-3.452716
H	-4.967398	1.996177	-2.636373
Si	-0.859458	0.446829	-5.751830
H	-1.994648	2.528541	-6.620155
H	-0.386959	2.285417	-7.318090
H	-0.549382	2.950126	-5.685642
H	1.457232	0.691030	-4.817826
H	1.473894	0.294081	-6.544583
H	1.179252	-0.976770	-5.344085
H	-2.450640	-1.251480	-6.796390
H	-0.832940	-1.359508	-7.497791
H	-1.916044	-0.043741	-7.967558
C	-2.295296	-2.695230	-2.861441
C	1.562053	-1.372577	-2.330250
C	1.451030	-3.864408	-2.121590
H	1.243642	-5.999710	-1.942033
C	-1.756433	-3.721749	0.739690
C	-0.134042	-4.936192	-0.683558
C	1.744928	-0.109725	4.254460
C	4.036999	-0.598105	3.591958
H	4.142109	0.371785	5.512087
H	5.573186	-0.575140	5.057068
H	4.087311	-1.385454	5.579504
C	4.720075	-0.946436	2.482033
H	6.330015	-2.334330	2.122233
H	6.726972	-0.965026	3.174676
H	6.570851	-0.736692	1.423427
C	4.178862	-1.565095	0.172252
Si	-1.499698	4.183821	-2.526258
H	-2.690188	2.958545	-4.331535
H	-2.586667	4.678167	-4.728070
H	-3.764428	4.112349	-3.528041
H	0.963789	4.392969	-2.379739
H	0.417374	4.677738	-4.039864
H	0.518152	3.020735	-3.406906
H	-2.798598	5.795747	-1.174000
H	-1.803898	6.634882	-2.375936
H	-1.064300	5.999958	-0.890096
C	-1.239766	4.514129	1.755218
H	-2.931399	3.310915	1.252685
H	-3.299813	4.669835	2.336736
H	-2.478645	3.219387	2.954372
H	-1.801410	6.472691	2.418304
C	0.983114	4.351689	0.740794
H	2.547836	2.889089	0.916634
H	2.872269	4.171200	-0.262360
H	1.719338	2.856589	-0.631479
H	2.077381	6.190670	0.610155
C	-4.978751	1.419366	2.110303
H	-3.957116	1.342510	0.205909
H	-5.659148	1.837343	0.120416
H	-5.226427	0.141202	0.402563
H	-6.582647	2.837057	2.067740
C	-4.043147	0.950688	4.350587
H	-4.963144	2.021284	5.960125
H	-3.133305	-0.855949	5.081188
H	-3.165762	0.483402	6.248091
H	-2.020667	0.494906	4.882193
C	-4.627700	1.266310	-5.941749
C	-5.481311	1.110868	-3.025507
C	-0.969166	2.225911	-6.389350
C	0.984852	0.071297	-5.587551
C	-1.588827	-0.660790	-7.123022
C	-3.401050	-2.951961	-2.029284
H	-3.348515	-1.713567	-0.250900
H	-4.972254	-2.148935	-0.812112
H	-4.009134	-0.920319	-1.662981
H	-4.859936	-4.404548	-1.432095
C	-1.848279	-3.672217	-3.769031
H	0.049564	-2.787754	-4.261727
H	-1.101842	-2.822141	-5.597573
H	-0.292718	-4.314738	-5.101886
H	-2.120516	-5.677416	-4.473872
C	0.880513	-5.026879	-1.620915
C	1.158927	-1.204116	4.905515
H	2.114744	-2.628461	3.642528
C	1.603183	1.212034	4.718251
H	0.748229	2.401219	6.288032
H	1.963826	2.182530	2.881479
C	4.482672	-0.536557	5.008526
C	6.159749	-1.264259	2.290781
C	4.408393	-2.951876	0.137874
H	5.110025	-4.559497	-1.106507
H	4.221773	-3.234317	2.240927
C	4.461919	-0.724638	-0.914143
H	5.235980	-0.693535	-2.921212
H	3.223163	0.876156	-0.307231
C	-2.747250	3.967097	-3.909560
C	0.262499	4.056028	-3.150834
C	-1.816464	5.803965	-1.660533
C	-2.561555	3.898676	2.100179
C	-1.016517	5.876290	1.959715
H	0.316665	7.546193	1.713505
C	2.089134	3.528905	0.151086
C	1.150329	5.722633	0.932952
C	-4.954914	1.177778	0.632101
C	-5.876403	2.305151	2.699266
H	-6.576731	3.215438	4.514446
C	-4.964126	1.846715	4.887734
C	-3.038158	0.230840	5.192787
C	-3.963448	-1.880815	-1.141438
C	-4.003560	-4.208702	-2.073939
H	-4.019614	-6.169057	-2.959854
C	-0.733296	-3.384679	-4.731672
C	-2.478478	-4.915948	-3.785249
C	1.327058	-2.625142	4.403987
H	2.655421	-3.238907	6.032763
H	1.983654	-4.576449	5.079928
H	0.972282	-3.737021	6.258124
H	-0.789185	-3.138721	4.455802
H	0.172723	-4.118197	3.327717
H	-0.253669	-2.444950	2.917136
C	0.882899	1.399388	5.896622
C	2.148446	2.405688	3.941059
H	0.332615	3.585271	4.225462
H	1.699552	4.481222	3.549995
H	1.696109	4.077766	5.268601
H	3.916829	2.793795	5.158634
H	3.940770	3.552196	3.561065
H	4.263480	1.821743	3.708604
C	4.929449	-3.491565	-1.038487
H	5.654420	-3.113699	-3.023029
C	4.091664	-3.835014	1.332912
H	2.452563	-4.946082	0.442555
H	1.942171	-3.441403	1.199872
H	2.381108	-4.840286	2.212956
H	4.850126	-5.545859	2.414820
H	6.077751	-4.761211	1.401768
H	4.828943	-5.785790	0.670264
C	5.004247	-1.309576	-2.058864
C	4.178372	0.766277	-0.837944
H	5.283964	1.187160	1.010637
H	5.054859	2.587826	-0.041427
H	6.246573	1.347676	-0.475685
H	4.970144	1.457538	-2.756055
H	3.673728	2.449953	-2.092594
H	3.287860	0.888625	-2.832017
C	0.166909	6.481836	1.556743
C	-5.870052	2.518335	4.073760
C	-3.541591	-5.194022	-2.936932
C	1.760713	-3.594332	5.511399
C	0.036418	-3.110092	3.734277
C	0.295275	0.330227	6.560428
C	1.416545	3.710246	4.273560
C	3.659711	2.642075	4.103421

C	5.235061	-2.677626	-2.120261
C	2.627652	-4.288853	1.297766
C	5.021292	-5.046412	1.455394
C	5.256755	1.511095	-0.033269
C	4.018666	1.418722	-2.213144

Calculated energies and coordinates of **10a**

Electronic energy ... -4938.67277289 Eh
Total Enthalpy ... -4936.95251924 Eh
Final Gibbs free energy ... -4937.17172178 Eh

CARTESIAN COORDINATES (ANGSTROEM)

C	4.327844	4.809136	23.940325
H	4.938959	4.359094	23.149327
H	3.305903	4.913460	23.554547
H	4.310210	4.105995	24.777871
C	9.860470	5.060728	34.797720
H	9.116028	6.418950	36.282538
H	9.479424	4.179689	35.307475
Si	9.252136	5.803896	29.433280
N	8.666168	4.765095	28.094115
N	10.795166	5.360982	29.872779
C	8.853618	7.502635	28.832258
C	7.941181	6.232080	30.754202
C	8.147166	5.369223	26.967262
C	9.103965	3.390287	27.965308
C	11.845461	5.459136	30.612068
C	8.237570	7.770113	27.640471
N	8.705198	8.372569	29.982845
C	8.175090	7.715061	31.082419
N	6.882975	5.604315	31.102958
Si	7.308663	6.282883	25.015138
C	7.991451	6.735607	26.693227
N	7.723425	4.682256	25.822698
C	10.181379	3.140870	27.095264
H	10.266784	4.785156	25.706921
H	11.797154	3.909711	25.919214
H	11.207852	5.012559	27.180004
H	11.400517	1.630480	26.192518
C	8.483994	2.346829	28.667198
H	7.303621	3.569602	30.044212
H	7.529812	1.882771	30.521162
H	6.414085	2.285845	29.219434
H	8.400980	0.220712	28.912317
N	12.018271	5.889632	31.929428
N	13.133763	5.103872	30.211613
O	7.700381	9.011069	27.444427
C	9.114217	9.737987	30.018768
O	8.005576	8.186767	32.194306
C	5.793628	6.268059	31.722652
Si	4.993472	6.507931	24.426203
H	5.059796	8.652512	23.088098
H	3.751653	7.574806	22.585792
H	5.402893	7.257167	22.049267
H	3.596273	6.544615	26.519760
H	3.069997	7.772087	25.359181
H	4.514328	8.043872	26.351934
Si	8.617090	6.565221	23.032307
H	7.501151	8.632847	22.102396
H	9.077664	8.317072	21.363016
H	8.987561	9.053400	22.969846
H	10.865656	6.775083	24.121153
H	10.999966	6.489318	22.378962
H	10.658695	5.141471	23.473796
H	7.810465	4.369803	22.080403
H	8.606544	5.366654	20.854061
H	6.949748	5.729932	21.351966
C	7.064814	3.418745	25.819925
C	10.904341	4.272530	26.432774
C	10.565925	1.823516	26.862560
H	10.202103	-0.255281	27.284515
C	7.380448	2.548547	29.665451
C	8.898894	1.038549	28.396781
C	11.034612	6.073530	32.963931
C	13.356925	5.699364	32.335896
H	13.657036	6.980493	34.041304
H	14.865739	5.700047	33.824030
H	13.247819	5.318893	34.439982
C	14.044797	5.237196	31.271054
H	15.627986	3.797011	31.059221
H	16.048022	5.254034	31.977248
H	15.906697	5.313904	30.211328
C	13.518272	4.432728	29.005465
Si	8.061234	10.163399	26.284050
H	6.884684	8.983255	24.438178
H	6.988461	10.711330	24.094419
H	5.804513	10.110031	25.271975
H	10.529292	10.185565	26.482734
H	10.036406	10.543063	24.821044
H	10.007267	8.869852	25.412012
H	6.861333	11.885351	27.590975
H	7.872686	12.624329	26.337389
H	8.607325	12.019278	27.838264
C	8.212485	10.725733	30.458433
H	6.384674	9.681418	30.044081
H	6.173350	11.337333	30.651024
H	6.633368	10.017058	31.751135
H	7.984037	12.803790	30.918089
C	10.420030	10.079518	29.613159
H	11.503531	8.220104	29.819762
H	12.379959	9.505849	28.973611
H	11.064799	8.605408	28.172808
H	11.832910	11.675689	29.408821
C	4.931966	7.096149	30.983420
H	6.128682	7.361104	29.168423
H	4.581646	8.220583	29.185854
H	4.628911	6.473241	28.955889
H	3.173888	8.316825	31.076846
C	5.529881	5.973249	33.071387
H	4.244282	6.357244	34.738618
H	6.557786	4.101220	33.304196
H	6.101698	4.888805	34.837236
H	7.460793	5.499910	33.869480
C	4.804143	7.606711	22.895526
C	3.949814	7.286185	25.798001
C	8.525467	8.311514	22.311532
C	10.454128	6.198998	23.285619
C	7.926312	5.393327	21.714594
C	5.935062	3.213529	26.639763
H	5.993306	4.457777	28.415505
H	4.371604	4.088939	27.817577
H	5.399986	5.272295	26.986223
H	4.420422	1.823647	27.240637
C	7.501085	2.406386	24.949245
H	9.075310	3.589412	24.061428
H	8.423243	2.314155	23.008885
H	9.494579	1.898787	24.349109
H	7.164037	0.401537	24.270754
C	9.906744	0.771221	27.484887
C	10.558542	4.914453	33.599058
H	11.325203	3.617688	32.088056
C	10.785140	7.367723	33.466996
H	9.863283	8.423853	35.092600
H	10.748860	8.477936	31.693450
C	13.805017	5.942263	33.731260
C	15.480060	4.881411	31.122602
C	13.722250	3.043827	29.059550
H	14.398635	1.345137	27.927895
H	13.360359	2.902036	31.157265
C	13.818418	5.192517	27.864331
H	14.601726	5.083577	25.865165
H	12.650217	6.856450	28.421255
C	6.823888	9.977509	24.891814
C	9.821208	9.913480	25.691991
C	7.833755	11.826874	27.088982
C	6.771444	10.426346	30.745179
C	8.673684	12.039009	30.569648
H	10.302029	13.416771	30.294086
C	11.394758	9.053227	29.116567
C	10.822274	11.411499	29.710770

C	5.090155	7.304861	29.501374
C	3.839748	7.667484	31.641100
H	2.740846	7.881236	33.476879
C	4.433773	6.566308	33.688317
C	6.457225	5.065078	33.817766
C	5.397500	4.311857	27.508833
C	5.292518	1.978131	26.609244
H	5.216715	0.006783	25.749378
C	8.687374	2.571416	24.040583
C	6.819386	1.187191	24.939467
C	10.775457	3.525380	33.028581
H	12.598204	3.069424	34.144911
H	11.747900	1.647027	33.515999
H	11.113261	2.502501	34.929802
H	8.850589	2.668105	33.607740
H	9.583253	1.922333	32.174227
H	8.824495	3.523934	32.059354
C	10.089049	7.451258	34.673535
C	11.152227	8.635924	32.699795
H	9.398265	9.777436	33.331042
H	10.677081	10.729604	32.573354
H	10.893457	10.165508	34.234362
H	13.112496	9.126779	33.521910
H	12.762343	9.845954	31.946669
H	13.209662	8.141280	32.041874
C	14.238638	2.418955	27.922918
H	14.983077	2.652572	25.923433
C	13.365871	2.230090	30.290706
H	11.924302	0.935631	29.314681
H	11.216898	2.430356	29.927125
H	11.658292	1.125807	31.057978
H	14.152032	0.671862	31.568234
H	15.403165	1.475024	30.600103
H	14.306386	0.302691	29.852068
C	14.356717	4.526730	26.763685
C	13.563129	6.688132	27.839150
H	14.780555	7.236285	29.577729
H	14.546363	8.542346	28.409631
H	15.665729	7.219931	28.035399
H	14.227775	7.239539	25.825972
H	12.959904	8.267401	26.492773
H	12.555518	6.646570	25.901612
C	9.966842	12.387159	30.208395
C	3.591220	7.419930	32.983485
C	5.731661	0.962874	25.768063
C	11.608180	2.638437	33.961950
C	9.427156	2.871599	32.698299
C	9.652907	6.316387	35.343560
C	10.480636	9.895648	33.254243
C	12.652846	8.934226	32.545243
C	14.565769	3.153778	26.792668
C	11.954117	1.650146	30.142117
C	14.370508	1.110460	30.588821
C	14.708009	7.463699	28.510255
C	13.313578	7.232850	26.430617

Calculated energies and coordinates of **TS19**

Electronic energy ... -3502.77046767 Eh
Total Enthalpy ... -3501.57739922 Eh
Final Gibbs free energy ... -3501.74277530 Eh

CARTESIAN COORDINATES (ANGSTROEM)

N	0.155328	-0.356971	0.714407
C	0.335703	-0.062797	1.953509
Si	0.737446	0.326474	-0.749250
N	-0.007177	-0.857866	3.042600
N	0.869205	1.083494	2.545348
C	-0.694576	0.823511	-1.776891
C	0.257638	-0.199254	4.252956
C	-0.567784	-2.163723	2.939662
C	0.799647	1.002524	3.949172
C	1.305846	2.239358	1.835514
Si	0.086250	-1.071799	-2.831942
N	-1.465393	1.508248	-2.469929
C	-0.092611	-0.813671	5.557987
C	0.270669	-3.272294	3.147586
C	-1.934936	-2.295222	2.662824
C	1.245451	2.122890	4.817702
C	2.592460	2.241423	1.267324
C	0.440142	3.343474	1.755941
Si	-2.029866	-1.654104	-3.757718
Si	1.080245	-3.056586	-1.977250
Si	1.520713	-0.193854	-4.498920
C	-1.767984	2.873450	-2.260652
H	-1.165567	-1.034863	5.614733
H	0.157874	-0.132730	6.373802
H	0.436924	-1.759533	5.725325
C	1.754780	-3.087019	3.408152
C	-0.295086	-4.541713	3.042316
C	-2.838275	-1.089344	2.496359
C	-2.451637	-3.587257	2.545557
H	1.291701	1.796548	5.858855
H	2.238722	2.483401	4.527070
H	0.566836	2.983160	4.761239
C	3.491264	1.019351	1.365533
C	3.013472	3.410127	0.631346
C	-0.972338	3.274906	2.307317
C	0.904660	4.485392	1.101831
C	-3.351449	-1.727855	-2.414453
C	-2.577700	-0.446329	-5.103786
C	-1.976155	-3.366393	-4.565591
C	0.011904	-3.711035	-0.570189
C	1.217712	-4.440156	-3.266075
C	2.855615	-2.700885	-1.429358
C	0.643443	1.072635	-5.601643
C	2.137292	-1.575770	-5.637591
C	3.046892	0.607855	-3.725424
C	-3.125641	3.201833	-2.094504
C	-0.771459	3.866898	-2.273758
C	2.344837	-4.145874	4.343161
C	2.511931	-3.051918	2.073417
H	1.896133	-2.109593	3.885625
C	-1.641067	-4.697285	2.731822
H	0.321651	-5.422219	3.193213
C	-4.031308	-1.139542	3.460430
C	-3.307936	-0.957988	1.043448
H	-2.253902	-0.193845	2.737462
H	-3.504273	-3.724675	2.313985
C	4.516731	0.932965	0.233459
C	4.194718	0.952553	2.730474
H	2.847189	0.133868	1.284190
C	2.181144	4.520170	0.557457
H	3.997169	3.451151	0.175625
C	-1.940223	2.791580	1.216526
C	-1.453997	4.603534	2.897819
H	-0.989560	2.529030	3.110440
H	0.258817	5.352483	1.007370
H	-3.477012	-0.760551	-1.921159
H	-4.315402	-2.019234	-2.850665
H	-3.089899	-2.463147	-1.646227
H	-2.468066	0.594719	-4.788544
H	-1.993467	-0.589392	-6.018864
H	-3.631640	-0.627781	-5.351012
H	-1.919281	-4.163746	-3.817597
H	-1.128623	-3.482284	-5.249120
H	-2.896442	-3.518771	-5.144046
H	-0.968266	-4.014725	-0.957065
H	0.467667	-4.590159	-0.098508
H	-0.152528	-2.950602	0.199620
H	1.818090	-5.253413	-2.837141
H	1.710160	-4.112977	-4.186395
H	0.242054	-4.852715	-3.534545
H	3.265599	-3.556116	-0.878613
H	2.932644	-1.812331	-0.794983
H	3.489036	-2.539318	-2.309605
H	-0.068702	1.701037	-5.058528
H	0.086246	0.566132	-6.396476
H	1.382556	1.728059	-6.079049
H	2.680291	-1.142147	-6.486910
H	1.312167	-2.174918	-6.037991

H	2.821991	-2.250785	-5.113684
H	3.573330	-0.104674	-3.081383
H	2.786246	1.472724	-3.107396
H	3.737196	0.936194	-4.513003
C	-4.165184	2.120483	-2.077985
C	-3.470399	4.534493	-1.885102
C	0.673564	3.547482	-2.529561
C	-1.167023	5.190270	-2.070343
H	1.769828	-4.231242	5.271455
H	3.375518	-3.880508	4.600649
H	2.374769	-5.133699	3.871017
H	2.438438	-4.022511	1.569986
H	2.087541	-2.299603	1.402456
H	3.572938	-2.825935	2.232716
H	-2.060986	-5.695032	2.639350
H	-4.614617	-0.215439	3.383863
H	-4.700966	-1.973678	3.224971
H	-3.703789	-1.254735	4.499098
H	-3.911486	-0.051374	0.917958
H	-3.925914	-1.814519	0.750734
H	-2.449484	-0.902531	0.368324
H	5.294669	1.699218	0.327912
H	4.036645	1.035819	-0.744917
H	5.015462	-0.041356	0.266323
H	4.791630	1.856187	2.900718
H	3.477675	0.849520	3.549657
H	4.868044	0.088680	2.763848
H	2.526494	5.417883	0.051694
H	-1.617294	1.835500	0.790220
H	-2.947625	2.664430	1.631075
H	-1.995210	3.523512	0.404480
H	-2.405763	4.453188	3.417925
H	-0.733804	5.017389	3.612089
H	-1.629088	5.351875	2.117224
H	-4.066387	1.462331	-2.947954
H	-4.053424	1.485349	-1.190054
H	-5.171511	2.547214	-2.067602
C	-2.498615	5.528831	-1.864680
H	-4.516581	4.790532	-1.737159
H	0.785172	2.829196	-3.346542
H	1.218722	4.457500	-2.796702
H	1.160122	3.111000	-1.650776
H	-0.405726	5.966966	-2.081905
H	-2.780565	6.565921	-1.708542

Calculated energies and coordinates of **7**

Electronic energy ... -3502.77740262 Eh
Total Enthalpy ... -3501.58366266 Eh
Final Gibbs free energy ... -3501.75197051 Eh

CARTESIAN COORDINATES (ANGSTROEM)

N	0.081477	0.020792	0.815394
C	0.309434	0.179435	2.080551
Si	0.921244	0.337392	-0.615463
N	-0.011105	-0.697060	3.100512
N	0.819558	1.291131	2.734847
C	-0.395087	0.315955	-2.025302
C	0.249347	-0.118619	4.352123
C	-0.510779	-2.021925	2.920363
C	0.755196	1.117788	4.128125
C	1.228620	2.491360	2.080997
Si	-0.082251	-1.252372	-3.161658
N	-1.193122	1.174887	-2.557587
C	-0.064882	-0.834723	5.613922
C	0.376393	-3.097920	3.098754
C	-1.867442	-2.202673	2.623458
C	1.154024	2.195728	5.070261
C	2.516613	2.555586	1.521722
C	0.319761	3.560985	2.032039
Si	-2.181330	-1.727875	-4.152497
Si	0.822183	-3.178154	-2.110525
Si	1.482173	-0.506212	-4.773987
C	-1.432984	2.485709	-2.127288
H	-1.129676	-1.091524	5.668961
H	0.179295	-0.206711	6.472900
H	0.495324	-1.773112	5.702524
C	1.859205	-2.856995	3.320401
C	-0.140210	-4.387209	2.986389
C	-2.816351	-1.036915	2.429920
C	-2.332026	-3.514014	2.497792
H	1.263279	1.792461	6.079309
H	2.106307	2.649636	4.776290
H	0.413147	3.004192	5.106215
C	3.480787	1.384001	1.617124
C	2.885956	3.752502	0.904962
C	-1.085951	3.421288	2.586539
C	0.736689	4.732168	1.399850
C	-3.449506	-1.936778	-2.767947
C	-2.725058	-0.348267	-5.321741
C	-2.160052	-3.325250	-5.167532
C	-0.146339	-3.617136	-0.553989
C	0.680727	-4.648080	-3.298580
C	2.666185	-3.011723	-1.717988
C	0.723032	0.889301	-5.799486
C	1.968379	-1.912849	-5.943983
C	3.069311	0.124795	-3.962148
C	-2.786112	2.860611	-1.944734
C	-0.418293	3.468167	-2.074878
C	2.529028	-3.926557	4.187104
C	2.567142	-2.739822	1.960828
H	1.978509	-1.895211	3.834576
C	-1.481852	-4.592609	2.686293
H	0.512143	-5.244095	3.121427
C	-4.015828	-1.118420	3.383862
C	-3.277684	-0.952586	0.968833
H	-2.274321	-0.111835	2.657194
H	-3.376347	-3.689963	2.255638
C	4.503358	1.349408	0.479607
C	4.196854	1.363315	2.977303
H	2.888407	0.463628	1.539363
C	2.006451	4.825751	0.846584
H	3.867136	3.842733	0.450901
C	-2.034502	2.900637	1.495987
C	-1.624300	4.722012	3.189824
H	-1.064473	2.670855	3.385523
H	0.056181	5.573618	1.322244
H	-3.469149	-1.058335	-2.117085
H	-4.455755	-2.081688	-3.180568
H	-3.209191	-2.806048	-2.145801
H	-2.542070	0.639361	-4.890224
H	-2.183227	-0.410141	-6.271573
H	-3.795723	-0.446773	-5.542254
H	-2.097665	-4.215759	-4.535864
H	-1.322988	-3.355730	-5.873054
H	-3.087851	-3.393531	-5.749756
H	-1.192080	-3.832240	-0.801677
H	0.270338	-4.509531	-0.071310
H	-0.143639	-2.804080	0.178976
H	1.297958	-5.474125	-2.923106
H	1.023636	-4.407217	-4.309949
H	-0.348679	-5.009995	-3.371938
H	3.014109	-3.904109	-1.182353
H	2.887607	-2.130891	-1.106733
H	3.247875	-2.934633	-2.643566
H	0.127290	1.572581	-5.184961
H	0.061387	0.486574	-6.573253
H	1.508987	1.470611	-6.297137
H	2.624317	-1.531583	-6.736602
H	1.093372	-2.364841	-6.423336
H	2.510238	-2.705298	-5.416428
H	3.547947	-0.652356	-3.358203
H	2.885162	0.976841	-3.300769
H	3.778421	0.440435	-4.738290
C	-3.863313	1.821336	-2.025948
C	-3.098678	4.187483	-1.678845
C	1.018654	3.154184	-2.373079
C	-0.782301	4.791220	-1.810868
H	1.992231	-4.079929	5.129488
H	3.554674	-3.624055	4.422020
H	2.588007	-4.890163	3.669894

H	2.493698	-3.685729	1.413127
H	2.111510	-1.963346	1.337823
H	3.628533	-2.500896	2.095123
H	-1.864088	-5.605226	2.592214
H	-4.635989	-0.221101	3.283649
H	-4.648131	-1.984267	3.159914
H	-3.693288	-1.198569	4.427392
H	-3.933069	-0.084906	0.832660
H	-3.838068	-1.848606	0.677852
H	-2.417845	-0.846653	0.300443
H	5.242645	2.153755	0.567959
H	4.010091	1.420512	-0.494877
H	5.049541	0.400919	0.510708
H	4.735294	2.303329	3.145031
H	3.495350	1.213328	3.802603
H	4.924851	0.544766	3.004973
H	2.312044	5.744147	0.352562
H	-1.679061	1.958762	1.065961
H	-3.037183	2.739143	1.910768
H	-2.114472	3.627320	0.682405
H	-2.567833	4.525851	3.709621
H	-0.921185	5.160858	3.906334
H	-1.833478	5.468460	2.415959
H	-3.787443	1.251331	-2.958375
H	-3.772219	1.094766	-1.210261
H	-4.854493	2.279390	-1.967346
C	-2.103736	5.159511	-1.606199
H	-4.140446	4.462886	-1.531275
H	1.108959	2.438053	-3.195121
H	1.557551	4.065745	-2.648383
H	1.546762	2.717451	-1.511034
H	-0.000241	5.546762	-1.784843
H	-2.361616	6.197028	-1.414407

Calculated energies and coordinates of **TS20**

Electronic energy ... -3502.75153562 Eh
Total Enthalpy ... -3501.55860304 Eh
Final Gibbs free energy ... -3501.72294623 Eh

CARTESIAN COORDINATES (ANGSTROEM)

N	0.227108	0.058611	0.559724
C	-0.060452	0.194345	1.816806
Si	0.030603	1.173020	-0.744580
N	-0.150989	-0.864669	2.721729
N	-0.328631	1.324330	2.605672
C	0.254454	0.412095	-2.448970
C	-0.441539	-0.397010	4.010947
C	0.257555	-2.216964	2.480512
C	-0.559370	0.945879	3.941952
C	-0.038932	2.685578	2.258145
Si	-0.652510	-1.075111	-3.223939
N	0.803656	1.260824	-3.333138
C	-0.606025	-1.292448	5.183806
C	1.634546	-2.495893	2.402084
C	-0.706762	-3.236735	2.464325
C	-0.911764	1.911596	5.022381
C	1.170879	3.245548	2.707029
C	-1.015883	3.449600	1.593328
Si	-2.908958	-1.234314	-2.526312
Si	0.636532	-2.996579	-2.727196
Si	-0.620500	-0.824531	-5.578640
C	1.469025	2.306013	-2.760986
H	-1.604178	-1.743742	5.220325
H	-0.467107	-0.721707	6.105997
H	0.121987	-2.110250	5.167661
C	2.680192	-1.401619	2.499553
C	2.026937	-3.829766	2.304616
C	-2.198008	-2.961112	2.486355
C	-0.261797	-4.557295	2.373133
H	-0.282519	1.757123	5.905186
H	-0.774156	2.942589	4.687376
H	-1.955327	1.801041	5.340044
C	2.224820	2.452250	3.464165
C	1.393032	4.602733	2.457460
C	-2.358660	2.851202	1.216098
C	-0.735496	4.793512	1.352338
C	-3.211003	-0.407237	-0.850624
C	-4.056218	-0.329501	-3.729509
C	-3.448593	-3.049008	-2.512674
C	0.344609	-3.671431	-0.995299
C	0.278095	-4.382997	-3.965792
C	2.471175	-2.547448	-2.856043
C	-1.397638	0.811132	-6.111910
C	-1.599905	-2.214291	-6.419016
C	1.156929	-0.889330	-6.218621
C	1.498082	3.543344	-3.457411
C	1.922440	2.223942	-1.381019
C	3.155192	-1.256918	3.953860
C	3.865697	-1.625133	1.556008
H	2.213479	-0.459686	2.198299
C	1.089146	-4.853701	2.299240
H	3.082810	-4.074758	2.243155
C	-2.928260	-3.743797	3.587398
C	-2.803614	-3.300740	1.117266
H	-2.346742	-1.889177	2.663857
H	-0.988386	-5.364384	2.351695
C	3.520750	2.316282	2.653906
C	2.529459	3.081291	4.832624
H	1.840169	1.442802	3.644348
C	0.454465	5.366545	1.783751
H	2.318480	5.063513	2.792791
C	-2.982500	3.501402	-0.019210
C	-3.330442	2.917124	2.404903
H	-2.190791	1.793857	0.977358
H	-1.458724	5.405391	0.824405
H	-3.171802	0.678887	-0.991295
H	-4.206275	-0.667841	-0.468015
H	-2.464974	-0.663244	-0.093064
H	-3.791636	0.730887	-3.803317
H	-4.024848	-0.756449	-4.736795
H	-5.091560	-0.392792	-3.370756
H	-2.820598	-3.667925	-1.865005
H	-3.390008	-3.465049	-3.525490
H	-4.487068	-3.145740	-2.172146
H	-0.624722	-4.172451	-0.907350
H	1.119726	-4.403519	-0.737055
H	0.380979	-2.868542	-0.251390
H	0.800526	-5.296780	-3.655588
H	0.622772	-4.127779	-4.973214
H	-0.791710	-4.611887	-4.022344
H	3.078075	-3.457022	-2.947280
H	2.796035	-2.021423	-1.952175
H	2.687907	-1.904629	-3.715246
H	-0.964327	1.635227	-5.540251
H	-2.480406	0.812536	-5.954007
H	-1.211249	0.991363	-7.178161
H	-1.603366	-2.059744	-7.505521
H	-2.643588	-2.233001	-6.086574
H	-1.171721	-3.202082	-6.223919
H	1.618489	-1.865246	-6.031718
H	1.763490	-0.124270	-5.722983
H	1.184674	-0.707719	-7.300347
C	1.057537	3.611957	-4.890756
C	1.846880	4.686431	-2.766060
C	2.839137	1.033378	-1.012780
C	2.282053	3.455254	-0.743967
H	2.324211	-1.001615	4.620484
H	3.911311	-0.468064	4.034689
H	3.600741	-2.193737	4.308173
H	4.491232	-2.468092	1.869268
H	3.522689	-1.812572	0.533598
H	4.504726	-0.735245	1.544490
H	1.415475	-5.887462	2.227456
H	-3.971605	-3.416663	3.652296
H	-2.935691	-4.817009	3.368928
H	-2.462849	-3.611579	4.568312
H	-3.858210	-3.007184	1.073700
H	-2.741503	-4.378006	0.924174
H	-2.268472	-2.786523	0.317681
H	3.937531	3.298851	2.404617

H	3.352829	1.771249	1.721604
H	4.275242	1.774663	3.234791
H	3.042324	4.042908	4.722440
H	1.618262	3.253848	5.411760
H	3.185304	2.420767	5.410511
H	0.648108	6.418050	1.590713
H	-2.277331	3.500920	-0.856871
H	-3.872378	2.938180	-0.319217
H	-3.305720	4.529701	0.178337
H	-4.297897	2.486438	2.123739
H	-2.950093	2.359773	3.264778
H	-3.494596	3.956021	2.714372
H	1.411480	2.746798	-5.456953
H	-0.036281	3.607255	-4.959928
H	1.423337	4.529674	-5.360744
C	2.210599	4.649080	-1.402140
H	1.824973	5.642255	-3.285096
H	2.694467	0.212550	-1.715301
H	3.879487	1.373551	-1.072686
H	2.656290	0.644872	-0.008195
H	2.643132	3.418866	0.278262
H	2.470531	5.571461	-0.890196

Calculated energies and coordinates of **INT18**

Electronic energy ... -3502.75672280 Eh
Total Enthalpy ... -3501.56298290 Eh
Final Gibbs free energy ... -3501.72887752 Eh

CARTESIAN COORDINATES (ANGSTROEM)

N	0.443959	0.018716	0.700139
C	0.038682	0.159411	1.922108
Si	0.460076	0.917943	-0.713063
N	-0.128519	-0.886486	2.822786
N	-0.328114	1.295431	2.639062
C	0.161367	0.404093	-2.393112
C	-0.549689	-0.396167	4.069716
C	0.282323	-2.244051	2.603563
C	-0.689355	0.942753	3.952553
C	-0.064091	2.666675	2.303791
Si	-0.738587	-1.042366	-3.183324
N	0.633547	1.395102	-3.254735
C	-0.768875	-1.272894	5.249414
C	1.658532	-2.531252	2.561708
C	-0.685291	-3.261931	2.531333
C	-1.129928	1.952901	4.948660
C	1.029914	3.295576	2.932137
C	-0.959515	3.373599	1.486121
Si	-2.964039	-1.285770	-2.433952
Si	0.586082	-2.952754	-2.750079
Si	-0.777418	-0.694603	-5.519124
C	1.336384	2.373809	-2.714282
H	-1.780938	-1.691088	5.272502
H	-0.618957	-0.705805	6.171072
H	-0.066041	-2.112072	5.247439
C	2.728403	-1.467061	2.699239
C	2.048100	-3.862704	2.418929
C	-2.180275	-3.002610	2.545880
C	-0.240756	-4.579102	2.406334
H	-0.308608	2.603754	5.270870
H	-1.907752	2.605575	4.537160
H	-1.537157	1.452266	5.829537
C	2.080419	2.507047	3.694362
C	1.174308	4.673104	2.766545
C	-2.135472	2.690465	0.814941
C	-0.756003	4.746789	1.334747
C	-3.174595	-0.547866	-0.699285
C	-4.188580	-0.334402	-3.520991
C	-3.487325	-3.105485	-2.468189
C	0.375677	-3.611792	-0.993658
C	0.228803	-4.367860	-3.956659
C	2.399996	-2.446836	-2.928509
C	-1.577887	0.958620	-5.958954
C	-1.769542	-2.069737	-6.369069
C	0.977276	-0.719763	-6.223022
C	1.726591	3.521740	-3.478659
C	1.696276	2.290140	-1.251874
C	3.541226	-1.672909	3.985398
C	3.635272	-1.431276	1.463562
H	2.240193	-0.492744	2.776402
C	1.109986	-4.880817	2.346928
H	3.106000	-4.105996	2.375991
C	-2.889114	-3.769471	3.674359
C	-2.797021	-3.396269	1.196327
H	-2.345610	-1.928540	2.694422
H	-0.969832	-5.381454	2.342510
C	3.206933	2.111599	2.727672
C	2.652495	3.248327	4.907430
H	1.623784	1.580737	4.063773
C	0.282659	5.394333	1.985486
H	2.003504	5.186237	3.243143
C	-2.463244	3.301674	-0.551096
C	-3.371028	2.696673	1.727096
H	-1.859231	1.641883	0.646893
H	-1.424065	5.319276	0.700799
H	-3.259623	0.538125	-0.801711
H	-4.091367	-0.912959	-0.220722
H	-2.329285	-0.750359	-0.034022
H	-3.928786	0.729192	-3.562846
H	-4.211502	-0.713196	-4.547752
H	-5.202718	-0.417808	-3.109644
H	-2.827619	-3.740681	-1.868816
H	-3.463511	-3.484057	-3.496948
H	-4.510536	-3.227747	-2.091471
H	-0.591079	-4.106724	-0.855589
H	1.157523	-4.346723	-0.764367
H	0.451142	-2.806022	-0.254682
H	0.798614	-5.261291	-3.672219
H	0.512951	-4.103071	-4.980730
H	-0.833561	-4.636112	-3.962490
H	3.056052	-3.319886	-2.822177
H	2.663750	-1.729324	-2.143858
H	2.608392	-1.974560	-3.893759
H	-1.113229	1.758087	-5.374716
H	-2.652532	0.957553	-5.751207
H	-1.440263	1.178027	-7.025276
H	-1.783155	-1.909508	-7.454505
H	-2.809787	-2.088776	-6.025775
H	-1.340784	-3.059792	-6.183048
H	1.436351	-1.710391	-6.131476
H	1.606516	0.000665	-5.690194
H	0.969175	-0.451211	-7.286810
C	1.562593	3.503977	-4.969562
C	2.169218	4.630493	-2.810325
C	3.058701	1.477655	-1.197908
C	1.936118	3.604503	-0.610751
H	2.892766	-1.662456	4.868423
H	4.286648	-0.877544	4.097308
H	4.073848	-2.629877	3.970907
H	4.214212	-2.356268	1.365842
H	3.039733	-1.301661	0.555032
H	4.347170	-0.601091	1.535067
H	1.432888	-5.912498	2.240108
H	-3.932876	-3.446454	3.752853
H	-2.893886	-4.845592	3.470632
H	-2.406238	-3.623318	4.644164
H	-3.857598	-3.124812	1.162415
H	-2.720259	-4.477594	1.036530
H	-2.287784	-2.899145	0.370316
H	3.708485	3.003003	2.333469
H	2.809875	1.547043	1.878762
H	3.954011	1.492478	3.237124
H	3.284904	4.091456	4.610097
H	1.862860	3.632557	5.561432
H	3.278766	2.567109	5.492632
H	0.413002	6.465708	1.863125
H	-1.572121	3.373406	-1.184724
H	-3.196732	2.679597	-1.072919
H	-2.902288	4.300599	-0.455041
H	-4.215645	2.217423	1.220096
H	-3.184372	2.150090	2.656830

H	-3.658698	3.723605	1.981245
H	2.001485	2.599627	-5.403237
H	0.502326	3.494287	-5.245448
H	2.030677	4.383563	-5.420526
C	2.202149	4.685841	-1.370806
H	2.446112	5.517934	-3.374262
H	3.346764	1.236237	-0.170852
H	2.997408	0.554809	-1.782396
H	3.832335	2.118844	-1.633704
H	1.990138	3.658435	0.471589
H	2.445964	5.632543	-0.893305

Calculated energies and coordinates of **TS21**

Electronic energy ... -3502.74103105 Eh
Total Enthalpy ... -3501.54946272 Eh
Final Gibbs free energy ... -3501.71468348 Eh

CARTESIAN COORDINATES (ANGSTROEM)

N	0.478576	0.002688	0.682053
C	0.070633	0.147104	1.902708
Si	0.550542	0.889411	-0.731005
N	-0.093282	-0.894931	2.807001
N	-0.284763	1.285935	2.615380
C	0.076951	0.463140	-2.426466
C	-0.512268	-0.398203	4.053864
C	0.308483	-2.255472	2.590245
C	-0.642913	0.940528	3.932255
C	0.000086	2.654177	2.283689
Si	-0.760424	-1.029856	-3.203857
N	0.440970	1.485256	-3.243440
C	-0.751866	-1.272763	5.231367
C	1.684335	-2.548697	2.547499
C	-0.664534	-3.266592	2.522153
C	-1.079677	1.957942	4.921982
C	1.108297	3.261308	2.907155
C	-0.891739	3.378992	1.480358
Si	-2.983394	-1.291381	-2.451445
Si	0.593221	-2.913623	-2.735266
Si	-0.774296	-0.702077	-5.543865
C	1.100882	2.518307	-2.680247
H	-1.774240	-1.665558	5.253152
H	-0.588248	-0.712214	6.154833
H	-0.070970	-2.129833	5.227980
C	2.744309	-1.478497	2.716358
C	2.066980	-3.882620	2.417450
C	-2.157290	-2.995688	2.534340
C	-0.227264	-4.587646	2.404231
H	-0.259742	2.618778	5.226034
H	-1.867293	2.599846	4.511476
H	-1.472720	1.464241	5.813178
C	2.175250	2.445733	3.616331
C	1.253110	4.642431	2.776615
C	-2.050086	2.705271	0.771494
C	-0.691264	4.755716	1.365679
C	-3.191466	-0.527903	-0.727498
C	-4.215926	-0.372479	-3.555947
C	-3.484830	-3.117155	-2.460051
C	0.357857	-3.606245	-0.995666
C	0.291033	-4.320325	-3.965849
C	2.402518	-2.374334	-2.863746
C	-1.618327	0.917404	-6.021173
C	-1.709751	-2.114483	-6.395249
C	0.996906	-0.672614	-6.204094
C	1.489427	3.655805	-3.441353
C	1.420561	2.434520	-1.250142
C	3.217803	-1.438956	4.177339
C	3.925530	-1.659922	1.757739
H	2.295546	-0.510822	2.484195
C	1.121499	-4.896153	2.350948
H	3.122380	-4.134494	2.380385
C	-2.874848	-3.761817	3.657495
C	-2.773109	-3.376283	1.180848
H	-2.314313	-1.921041	2.687477
H	-0.960653	-5.386278	2.342876
C	3.300028	2.125304	2.617456
C	2.750373	3.126003	4.862927
H	1.733398	1.495003	3.937851
C	0.349618	5.385840	2.030008
H	2.092376	5.140893	3.251590
C	-2.312783	3.312201	-0.611190
C	-3.317883	2.730850	1.637188
H	-1.781285	1.651810	0.621594
H	-1.359642	5.343441	0.745754
H	-3.271807	0.557579	-0.842414
H	-4.109096	-0.882846	-0.242996
H	-2.346904	-0.726134	-0.059798
H	-3.969437	0.693279	-3.616538
H	-4.230143	-0.770691	-4.575693
H	-5.230372	-0.461500	-3.146661
H	-2.815385	-3.737099	-1.855832
H	-3.460577	-3.507747	-3.484346
H	-4.505103	-3.246890	-2.077933
H	-0.599810	-4.124929	-0.885109
H	1.151342	-4.328672	-0.766085
H	0.403725	-2.814619	-0.239747
H	0.864607	-5.208030	-3.671194
H	0.601518	-4.043173	-4.978815
H	-0.766911	-4.603165	-4.004464
H	3.068030	-3.244481	-2.801336
H	2.652382	-1.700128	-2.037140
H	2.614383	-1.845961	-3.798872
H	-1.173715	1.743660	-5.459647
H	-2.692235	0.893399	-5.811123
H	-1.488678	1.110232	-7.093689
H	-1.718655	-1.958599	-7.481342
H	-2.752342	-2.162615	-6.061339
H	-1.253501	-3.090220	-6.201051
H	1.492973	-1.642030	-6.083496
H	1.583983	0.081785	-5.669618
H	1.006207	-0.421828	-7.272148
C	1.231952	3.680619	-4.919375
C	2.057251	4.725679	-2.788023
C	2.781714	1.115701	-1.214400
C	1.896616	3.636270	-0.611509
H	2.383434	-1.237772	4.858368
H	3.971202	-0.655609	4.318586
H	3.665114	-2.397267	4.465354
H	4.543873	-2.523652	2.025158
H	3.578815	-1.795619	0.728051
H	4.572952	-0.776750	1.788506
H	1.439429	-5.930257	2.252711
H	-3.916150	-3.430717	3.734612
H	-2.887984	-4.837025	3.449408
H	-2.393224	-3.622984	4.629050
H	-3.832332	-3.099646	1.146710
H	-2.700331	-4.456641	1.012468
H	-2.259552	-2.874734	0.360129
H	3.780754	3.047984	2.272547
H	2.910043	1.603735	1.737679
H	4.064539	1.495249	3.085768
H	3.357819	4.000499	4.606980
H	1.963558	3.451737	5.550964
H	3.401507	2.426159	5.397076
H	0.478345	6.460299	1.936885
H	-1.394157	3.373507	-1.205419
H	-3.030448	2.695350	-1.160582
H	-2.744439	4.316195	-0.537883
H	-4.147137	2.249063	1.107711
H	-3.165689	2.197479	2.581145
H	-3.608016	3.762879	1.866490
H	1.604149	2.771187	-5.401980
H	0.156719	3.722413	-5.127470
H	1.706326	4.552104	-5.379257
C	2.219593	4.718747	-1.372916
H	2.351351	5.605919	-3.353328
H	2.856711	0.344602	-0.433372
H	2.856427	0.683613	-2.211081
H	3.568006	1.846184	-1.040176
H	2.027263	3.661567	0.464132
H	2.593124	5.616432	-0.884270

Calculated energies and coordinates of **8**

Electronic energy ... -3502.84072910 Eh
Total Enthalpy ... -3501.64700258 Eh
Final Gibbs free energy ... -3501.81214804 Eh

CARTESIAN COORDINATES (ANGSTROEM)

N	0.618929	0.006659	0.685428
C	0.220450	0.194629	1.886336
Si	1.171810	0.615032	-0.785854
N	0.015401	-0.819858	2.826182
N	-0.100798	1.358281	2.586996
C	0.002634	0.350290	-2.300982
C	-0.400630	-0.287184	4.057371
C	0.328390	-2.202563	2.643357
C	-0.469630	1.053675	3.912676
C	0.193564	2.709521	2.219400
Si	-0.671613	-1.178231	-3.234094
N	-0.245495	1.478619	-2.902509
C	-0.694172	-1.146955	5.232646
C	1.677990	-2.599167	2.644571
C	-0.719238	-3.131820	2.562951
C	-0.853890	2.113613	4.879077
C	1.352160	3.294854	2.757386
C	-0.736976	3.438755	1.460881
Si	-3.001320	-1.272530	-2.783305
Si	0.498279	-3.176533	-2.739685
Si	-0.195313	-0.643714	-5.497367
C	0.384633	2.626450	-2.339308
H	-1.643471	-1.683947	5.124900
H	-0.752436	-0.537324	6.136769
H	0.087066	-1.901485	5.377713
C	2.799093	-1.586962	2.792657
C	1.956027	-3.962718	2.560967
C	-2.171645	-2.705403	2.470641
C	-0.389934	-4.487358	2.493990
H	-0.008962	2.764675	5.132018
H	-1.640116	2.759774	4.472133
H	-1.226579	1.661364	5.800355
C	2.410486	2.464086	3.463146
C	1.537880	4.665625	2.570350
C	-1.934637	2.754266	0.820827
C	-0.507166	4.805089	1.304999
C	-3.346297	-0.086464	-1.354152
C	-4.039477	-0.673569	-4.244040
C	-3.601369	-3.021986	-2.387785
C	0.663393	-3.510015	-0.890860
C	-0.404513	-4.641984	-3.527132
C	2.231223	-3.133323	-3.504366
C	-1.367076	0.638099	-6.238581
C	-0.267668	-2.183146	-6.599095
C	1.552997	0.072851	-5.548193
C	0.224531	3.871601	-2.962463
C	1.205988	2.452915	-1.209481
C	3.066914	-1.301254	4.279028
C	4.091176	-2.008068	2.088098
H	2.471227	-0.657407	2.318194
C	0.932932	-4.899804	2.494943
H	2.987399	-4.300055	2.553621
C	-3.094261	-3.504561	3.399683
C	-2.640693	-2.834630	1.015958
H	-2.241833	-1.646874	2.746411
H	-1.181194	-5.228345	2.425930
C	3.514433	2.079694	2.465480
C	3.018799	3.157178	4.687264
H	1.945689	1.534025	3.811209
C	0.605846	5.415059	1.869435
H	2.421036	5.150334	2.976013
C	-2.538979	3.570197	-0.324087
C	-3.032382	2.414075	1.842312
H	-1.568207	1.808845	0.395125
H	-1.194418	5.404458	0.719100
H	-3.090923	0.927066	-1.681206
H	-4.406933	-0.101921	-1.073434
H	-2.747373	-0.302645	-0.464708
H	-3.805644	0.362680	-4.506301
H	-3.883705	-1.291438	-5.134855
H	-5.104146	-0.726383	-3.982388
H	-2.984649	-3.518552	-1.634421
H	-3.591447	-3.641543	-3.291524
H	-4.633048	-2.991960	-2.015274
H	-0.283004	-3.868162	-0.473463
H	1.416425	-4.285924	-0.705071
H	0.945457	-2.619684	-0.318364
H	0.206279	-5.548153	-3.427930
H	-0.592275	-4.482145	-4.594078
H	-1.366293	-4.832120	-3.041177
H	2.773026	-4.048635	-3.233930
H	2.823210	-2.280244	-3.160057
H	2.184404	-3.086736	-4.597347
H	-1.538023	1.451418	-5.527599
H	-2.333934	0.201078	-6.505516
H	-0.925423	1.060722	-7.150008
H	-0.070184	-1.905663	-7.642078
H	-1.256877	-2.653694	-6.563593
H	0.471935	-2.936612	-6.309515
H	2.291199	-0.612624	-5.119635
H	1.590936	1.008703	-4.980495
H	1.849038	0.287580	-6.582519
C	-0.664030	4.037822	-4.162010
C	0.940773	4.949345	-2.434093
C	2.861937	-0.121486	-1.157267
C	1.919220	3.543449	-0.729869
H	2.184261	-0.884802	4.774865
H	3.887390	-0.582813	4.390330
H	3.350846	-2.222806	4.800795
H	4.592889	-2.834451	2.604006
H	3.899940	-2.310827	1.053898
H	4.790677	-1.165544	2.070874
H	1.171157	-5.958143	2.435461
H	-4.099199	-3.069156	3.394475
H	-3.189881	-4.545029	3.071630
H	-2.728784	-3.515311	4.431398
H	-3.659304	-2.449585	0.893430
H	-2.635540	-3.887068	0.708564
H	-1.977497	-2.283537	0.342866
H	4.021066	2.975284	2.086830
H	3.099860	1.535607	1.610727
H	4.266364	1.444405	2.947210
H	3.638948	4.014739	4.405108
H	2.247340	3.512077	5.378028
H	3.662952	2.455092	5.227381
H	0.759498	6.482228	1.735392
H	-1.780531	3.855836	-1.059556
H	-3.304554	2.976790	-0.832152
H	-3.029905	4.478037	0.045891
H	-3.885295	1.958967	1.326420
H	-2.688362	1.707867	2.602633
H	-3.384154	3.323737	2.343700
H	-0.273385	3.479915	-5.020225
H	-1.668826	3.648375	-3.969255
H	-0.742755	5.092089	-4.442821
C	1.786941	4.790190	-1.341454
H	0.839478	5.927747	-2.899033
H	2.840700	-1.211710	-1.061877
H	3.185134	0.139738	-2.171350
H	3.611959	0.261690	-0.454914
H	2.569928	3.443123	0.133842
H	2.338620	5.644083	-0.957208

Calculated energies and coordinates of **TS22**

Electronic energy ... -3502.74108836 Eh
Total Enthalpy ... -3501.54966659 Eh
Final Gibbs free energy ... -3501.71551090 Eh

CARTESIAN COORDINATES (ANGSTROEM)

N	0.020422	0.368699	0.656362
C	-0.145920	0.442461	1.935202

Si	1.236608	0.291334	-0.518917
N	-0.254253	-0.617233	2.834673
N	-0.277975	1.563883	2.743140
C	0.552665	-0.234027	-2.262901
C	-0.449180	-0.150438	4.143881
C	-0.291748	-2.001913	2.491843
C	-0.468998	1.201717	4.085591
C	-0.218725	2.926018	2.316980
Si	-0.141907	-1.617787	-3.383347
N	0.776703	0.894337	-2.754199
C	-0.582103	-1.075532	5.300309
C	0.911142	-2.673436	2.222321
C	-1.536345	-2.653745	2.517227
C	-0.630489	2.220093	5.154815
C	1.038391	3.509512	2.087466
C	-1.421040	3.645147	2.231774
Si	-2.374235	-1.971605	-2.703564
Si	1.385337	-3.404831	-3.137083
Si	-0.063985	-0.712700	-5.570134
C	1.678676	2.050623	-1.575508
H	-1.557894	-1.576318	5.324368
H	-0.465464	-0.524214	6.235978
H	0.178398	-1.862666	5.267481
C	2.244811	-1.946742	2.251012
C	0.838852	-4.044035	1.969232
C	-2.823013	-1.886618	2.772683
C	-1.554402	-4.024308	2.254709
H	-0.589906	1.741602	6.135608
H	0.158653	2.978407	5.104261
H	-1.588406	2.747972	5.074818
C	2.320167	2.696390	2.196334
C	1.062736	4.866439	1.765390
C	-2.768574	2.972614	2.433908
C	-1.340904	4.999831	1.907368
C	-2.962745	-0.326890	-1.992308
C	-3.452366	-2.424551	-4.188556
C	-2.518244	-3.375410	-1.451451
C	1.008074	-4.453811	-1.615632
C	1.354893	-4.516795	-4.664936
C	3.115470	-2.674989	-2.950001
C	-1.145401	0.830733	-5.677269
C	-0.684500	-1.969096	-6.841368
C	1.712984	-0.242896	-6.001011
C	0.877488	3.220001	-1.443438
C	3.042774	2.213108	-1.974709
C	2.783155	-1.830993	3.685661
C	3.295461	-2.580052	1.336733
H	2.073488	-0.929926	1.876713
C	-0.378893	-4.711281	1.984695
H	1.744628	-4.598646	1.747779
C	-3.894879	-2.718618	3.482049
C	-3.365577	-1.306342	1.460250
H	-2.590887	-1.032139	3.418332
H	-2.497469	-4.561340	2.257697
C	3.506453	3.343871	1.481852
C	2.700964	2.408107	3.658853
H	2.133652	1.732006	1.702019
C	-0.111695	5.602680	1.681330
H	2.009049	5.352016	1.556556
C	-3.338858	2.496751	1.088602
C	-3.779693	3.867192	3.159508
H	-2.615641	2.079500	3.051987
H	-2.248359	5.590403	1.826955
H	-2.953066	0.444817	-2.770767
H	-3.981107	-0.395930	-1.590358
H	-2.296080	0.010891	-1.191777
H	-3.464946	-1.624158	-4.935549
H	-3.101890	-3.339080	-4.679344
H	-4.486325	-2.595172	-3.863663
H	-1.889699	-3.211969	-0.570010
H	-2.211976	-4.322059	-1.909877
H	-3.557102	-3.491002	-1.118274
H	0.108930	-5.062104	-1.756849
H	1.845152	-5.131213	-1.403071
H	0.845057	-3.831852	-0.729872
H	2.006803	-5.385243	-4.508863
H	1.715439	-3.985127	-5.551982
H	0.347778	-4.889451	-4.881842
H	3.856793	-3.474406	-2.826202
H	3.172456	-2.017965	-2.075000
H	3.399403	-2.082245	-3.826024
H	-0.802467	1.584544	-4.961581
H	-2.196987	0.608541	-5.465225
H	-1.092426	1.261426	-6.685033
H	-0.622944	-1.536164	-7.847697
H	-1.728799	-2.244069	-6.661712
H	-0.091001	-2.888963	-6.838359
H	2.374484	-1.116344	-6.007877
H	2.105357	0.481863	-5.279951
H	1.753659	0.214123	-6.997592
C	-0.601031	3.108172	-1.225809
C	1.452737	4.475515	-1.619163
C	3.883218	1.002074	-2.199461
C	3.569550	3.483168	-2.159155
H	2.122545	-1.229722	4.316794
H	3.766823	-1.348389	3.680368
H	2.891392	-2.822871	4.140104
H	3.658663	-3.537221	1.728347
H	2.901162	-2.742628	0.328895
H	4.158231	-1.911281	1.253746
H	-0.411508	-5.777092	1.776808
H	-4.727881	-2.073381	3.779865
H	-4.307075	-3.493503	2.826635
H	-3.502055	-3.208086	4.379858
H	-4.240907	-0.675363	1.651987
H	-3.663444	-2.109691	0.779959
H	-2.606302	-0.702884	0.955259
H	3.865738	4.226798	2.024413
H	3.258118	3.634707	0.459021
H	4.335597	2.630059	1.435464
H	2.818322	3.346410	4.214092
H	1.960992	1.789313	4.173085
H	3.658423	1.876155	3.692043
H	-0.067068	6.658086	1.426713
H	-2.670696	1.776728	0.608609
H	-4.313268	2.017340	1.235632
H	-3.471462	3.342837	0.404726
H	-4.666414	3.283990	3.429293
H	-3.358907	4.296659	4.075058
H	-4.118802	4.693939	2.526127
H	-1.089147	2.716955	-2.128971
H	-0.834403	2.414575	-0.414029
H	-1.040075	4.081862	-0.990683
C	2.788914	4.623920	-1.974433
H	0.824271	5.355479	-1.496957
H	3.508674	0.405174	-3.040933
H	4.928574	1.258477	-2.394291
H	3.824741	0.336727	-1.317812
H	4.613698	3.581666	-2.449475
H	3.214146	5.612110	-2.123349

Calculated energies and coordinates of **9**

Electronic energy ... -3502.81185630 Eh
Total Enthalpy ... -3501.61804947 Eh
Final Gibbs free energy ... -3501.78528798 Eh

CARTESIAN COORDINATES (ANGSTROEM)

N	0.423603	0.147264	0.845349
C	0.110949	0.366677	2.067391
Si	0.413742	0.759378	-0.707856
N	-0.020945	-0.630111	3.023893
N	-0.142070	1.540533	2.762085
C	-0.393462	-0.169052	-2.028607
C	-0.384150	-0.085935	4.264125
C	-0.045441	-2.017378	2.683398
C	-0.454224	1.257611	4.106867
C	-0.115279	2.857629	2.209542
Si	-0.649488	-1.442920	-3.414698
N	-1.129301	0.828173	-1.625366
C	-0.640564	-0.944296	5.448605

C	1.171790	-2.691104	2.494456
C	-1.290136	-2.641553	2.514539
C	-0.749621	2.337577	5.081790
C	1.129971	3.476681	1.984854
C	-1.335573	3.509828	1.965613
Si	-2.722189	-2.513824	-3.083214
Si	1.185557	-2.901838	-3.262504
Si	-0.622548	-0.042510	-5.307645
C	1.783865	1.899618	-1.266245
H	-1.549820	-1.545143	5.322622
H	-0.765483	-0.328199	6.341480
H	0.182389	-1.646159	5.624136
C	2.493570	-1.954687	2.631908
C	1.113734	-4.044942	2.165882
C	-2.587306	-1.856830	2.594238
C	-1.296373	-3.998112	2.183298
H	-0.746419	1.936872	6.097741
H	-0.003192	3.137926	5.024114
H	-1.729116	2.795663	4.902798
C	2.433994	2.735673	2.236430
C	1.120199	4.794113	1.527741
C	-2.676355	2.812265	2.109629
C	-1.287839	4.832418	1.519098
C	-3.972877	-1.259796	-2.435901
C	-3.358491	-3.263163	-4.698709
C	-2.503527	-3.916986	-1.831561
C	1.347481	-3.483158	-1.471379
C	0.949342	-4.402714	-4.388121
C	2.763391	-2.001214	-3.787169
C	-2.191037	1.006881	-5.276356
C	-0.546496	-1.043458	-6.909183
C	0.877697	1.099178	-5.213796
C	1.500949	3.173120	-1.805356
C	3.112822	1.422372	-1.273975
C	2.876807	-1.750786	4.106365
C	3.642573	-2.630453	1.880776
H	2.348841	-0.965207	2.180244
C	-0.107026	-4.692754	2.018124
H	2.030430	-4.601045	2.001437
C	-3.715785	-2.626760	3.288152
C	-3.016913	-1.414460	1.186559
H	-2.404006	-0.947635	3.178627
H	-2.241328	-4.512486	2.036475
C	3.637586	3.390706	1.556173
C	2.722889	2.574049	3.739375
H	2.316530	1.732994	1.802751
C	-0.074969	5.469528	1.311661
H	2.056879	5.301647	1.327363
C	-3.230295	2.448768	0.722930
C	-3.698088	3.655517	2.885396
H	-2.521877	1.878115	2.662594
H	-2.213775	5.362287	1.315759
H	-4.233989	-0.534092	-3.213546
H	-4.894483	-1.758254	-2.109947
H	-3.561693	-0.694783	-1.593288
H	-3.575953	-2.483503	-5.437063
H	-2.625552	-3.947488	-5.140470
H	-4.283343	-3.827607	-4.526082
H	-1.993381	-3.576526	-0.923908
H	-1.906330	-4.729636	-2.260911
H	-3.476194	-4.334747	-1.542003
H	0.537841	-4.171206	-1.205096
H	2.298362	-4.008571	-1.315270
H	1.297612	-2.645450	-0.765865
H	1.813393	-5.075975	-4.330633
H	0.832031	-4.097999	-5.434154
H	0.059373	-4.977102	-4.107936
H	3.653726	-2.590038	-3.533573
H	2.851563	-1.023406	-3.301114
H	2.768798	-1.834400	-4.870317
H	-2.306302	1.486412	-4.297193
H	-3.083345	0.397887	-5.458868
H	-2.153410	1.789863	-6.043776
H	-0.585674	-0.380702	-7.782474
H	-1.383385	-1.746451	-6.981834
H	0.382387	-1.621473	-6.971046
H	1.816244	0.541424	-5.296293
H	0.904637	1.646725	-4.265007
H	0.849704	1.834334	-6.027844
C	0.098951	3.717189	-1.828292
C	2.538919	3.932389	-2.350792
C	3.465666	0.098654	-0.649853
C	4.124414	2.197584	-1.841354
H	2.148384	-1.125788	4.630156
H	3.851320	-1.254655	4.177889
H	2.946040	-2.714304	4.624703
H	3.938814	-3.573695	2.354026
H	3.377382	-2.834361	0.838570
H	4.520122	-1.975556	1.886231
H	-0.128910	-5.746483	1.754176
H	-4.578265	-1.968477	3.436663
H	-4.057817	-3.475223	2.686023
H	-3.403741	-3.011160	4.265247
H	-3.899090	-0.766336	1.240617
H	-3.274960	-2.285464	0.574185
H	-2.216072	-0.868607	0.676814
H	3.911062	4.331513	2.048885
H	3.452507	3.587072	0.496867
H	4.503890	2.724617	1.627128
H	2.769575	3.555630	4.225714
H	1.968445	1.968929	4.248945
H	3.692248	2.082698	3.879492
H	-0.056454	6.496831	0.958412
H	-2.548934	1.803097	0.162667
H	-4.193023	1.934288	0.821216
H	-3.390734	3.354323	0.126104
H	-4.590738	3.057865	3.098233
H	-3.294422	4.021564	3.834964
H	-4.022271	4.525881	2.304994
H	-0.512063	3.206633	-2.583776
H	-0.407107	3.567497	-0.869496
H	0.099390	4.788171	-2.050951
C	3.840259	3.446865	-2.378497
H	2.317820	4.912458	-2.767044
H	2.691434	-0.656175	-0.832748
H	4.416994	-0.283107	-1.032611
H	3.555251	0.194523	0.439771
H	5.143763	1.818856	-1.855270
H	4.634929	4.044705	-2.816746

5. References

(S1) Zhu, H.; Kostenko, A.; Franz, D.; Hanusch, F.; Inoue, S. *J. Am. Chem. Soc*. **2023**, *145*, 1011-1021.

(S2) Singh, C.; Prakasham, A. P.; Gangwar, M. K.; Butcher, R. J.; Ghosh, P. *ACS Omega* **2018**, *3*, 1740−1756.

(S3) APEX suite of crystallographic software, APEX 4 version 2021.10-0; Bruker AXS Inc.: Madison, Wisconsin, USA, 2021.

(S4) SAINT, Version 7.56a and SADABS Version 2008/1; Bruker AXS Inc.: Madison, Wisconsin, USA, 2008.

(S5) Sheldrick, G. M. SHELXL-2014, University of Göttingen, Göttingen, Germany, 2014.

(S6) Hübschle, C. B.; Sheldrick, G. M.; Dittrich, B. J. Appl. Cryst. 2011, 44, 1281-1284.

(S7) Sheldrick, G. M. SHELXL-97, University of Göttingen, Göttingen, Germany, 1998.

(S8) Wilson, A. J. C. International Tables for Crystallography, Vol. C, Tables 6.1.1.4 (pp. 500-502), 4.2.6.8 (pp. 219-222), and 4.2.4.2 (pp. 193-199); Kluwer Academic Publishers: Dordrecht, The Netherlands, 1992.

(S9) Macrae, C. F.; Bruno, I. J.; Chisholm, J. A.; Edgington, P. R.; McCabe, P.; Pidcock, E.; Rodriguez-Monge, L.; Taylor, R.; van de Streek, J.; Wood, P. A. J. Appl. Cryst. 2008, 41, 466-470.

(S10) Neese, F. Software update: The ORCA program system—Version 5.0. *WIREs Comput Mol Sci* **2022**, *12* (5).

(S11) Grimme, S.; Hansen, A.; Ehlert, S.; Mewes, J.-M. r2SCAN-3c: A "Swiss army knife" composite electronic-structure method. *J. Chem. Phys.* **2021**, *154* (6), 64103.

(S12) Furness, J. W.; Kaplan, A. D.; Ning, J.; Perdew, J. P.; Sun, J. Accurate and Numerically Efficient r2SCAN Meta-Generalized Gradient Approximation. *J. Phys. Chem. Lett.* **2020**, *11* (19), 8208–8215.

(S13) Furness, J. W.; Kaplan, A. D.; Ning, J.; Perdew, J. P.; Sun, J. Correction to "Accurate and Numerically Efficient r2SCAN Meta-Generalized Gradient Approximation". *J. Phys. Chem. Lett.* **2020**, *11* (21), 9248.

(S14) Kruse, H.; Grimme, S. A geometrical correction for the inter- and intra-molecular basis set superposition error in Hartree-Fock and density functional theory calculations for large systems. *J. Chem. Phys.* **2012**, *136* (15), 154101.

(S15) Caldeweyher, E.; Bannwarth, C.; Grimme, S. Extension of the D3 dispersion coefficient model. *J. Phys. Chem.* **2017**, *147* (3), 34112.

(S16) Caldeweyher, E.; Ehlert, S.; Hansen, A.; Neugebauer, H.; Spicher, S.; Bannwarth, C.; Grimme, S. A generally applicable atomic-charge dependent London dispersion correction. *J. Chem. Phys.* **2019**, *150* (15), 154122.

(S17) Caldeweyher, E.; Mewes, J.-M.; Ehlert, S.; Grimme, S. Extension and evaluation of the D4 London-dispersion model for periodic systems. *Phys. Chem. Chem. Phys.* **2020**, *22* (16), 8499–8512.

(S18) Marenich, A. V.; Cramer, C. J.; Truhlar, D. G. Universal solvation model based on solute electron density and on a continuum model of the solvent defined by the bulk dielectric constant and atomic surface tensions. *J. Phys. Chem. B* **2009**, *113* (18), 6378–6396.

(S19) Zhao, Y.; Truhlar, D. G. Design of density functionals that are broadly accurate for thermochemistry, thermochemical kinetics, and nonbonded interactions. *J. Phys. Chem. A* **2005**, *109* (25), 5656–5667.

(S20) Weigend, F.; Ahlrichs, R. Balanced basis sets of split valence, triple zeta valence and quadruple zeta valence quality for H to Rn: Design and assessment of accuracy. *Phys. Chem. Chem. Phys.* **2005**, *7* (18), 3297–3305.

(S21) Weigend, F. Accurate Coulomb-fitting basis sets for H to Rn. *Phys. Chem. Chem. Phys.* **2006**, *8* (9), 1057–1065.
